# Structural and diffusion imaging in olfactory-related brain regions in Parkinson’s disease: predictors of clinical progression

**DOI:** 10.1038/s41598-025-19551-0

**Published:** 2025-10-13

**Authors:** Seyyed Mohammad Hosseini, Ahmadreza Sohrabi-Ashlaghi, Shahriar Kolahi, Narges Azizi, Hoda Borooghani, Zeinab Gharaylou, Samira Raminfard, Hossein Ghanaati, Hedayat Abbastabar, Amir Hossein Jalali, Madjid Shakiba, Nafiseh Ghavami, Kavous Firouznia

**Affiliations:** Advanced Diagnostic and Interventional Radiology Research Center (ADIR), Tehran University of Medical Sciences (TUMS), Tehran, Iran

**Keywords:** Parkinson’s disease, T1-weighted MRI, Diffusion tensor imaging, Olfactory brain regions, Cognitive impairment, Longitudinal study, Olfactory system, Cognitive neuroscience, Parkinson's disease

## Abstract

Olfactory dysfunction is a prevalent non-motor symptom in Parkinson’s disease (PD). Structural and diffusion MRI studies suggest that olfactory-related brain regions undergo significant neurodegenerative changes in PD. The current study aims to explore the longitudinal structural and diffusion imaging in olfactory-related regions in PD over four years. The relationships between baseline imaging and fluid biomarkers, and subsequent cognitive and clinical changes were also explored. We analyzed 97 newly diagnosed early-stage PD patients from the Parkinson’s Progression Markers Initiative (PPMI) who underwent T1-weighted MRI, diffusion tensor imaging (DTI), and clinical assessments at baseline, two years, and four years. Structural and diffusion measures of olfactory-related regions were extracted using FreeSurfer and ExploreDTI. Baseline fluid biomarkers were also evaluated. Baseline mean diffusivity (MD) displayed significant associations with further cognitive changes across multiple regions, including amygdala, orbitofrontal cortex (OFC), insula, and thalamus (β=–0.214 to − 0.422). Baseline structural measures, including amygdala volume and entorhinal and OFC thickness, were associated with subsequent changes in cognitive and Unified Parkinson’s Disease Rating Scale (UPDRS) scores (β = 0.264 to 0.402). Moreover, baseline serum neurofilament-light chain levels predicted multiple cognitive score changes (β= − 0.603 to − 0.331). Longitudinal analyses revealed a significant MD increase in the thalamus along with gradual reductions in volume and cortical thickness in the amygdala, insula, OFC, and entorhinal cortex (FDR-adjusted *p* < 0.05). Linear mixed-effect models further confirmed thalamic diffusion metrics as a predictor of cognitive deterioration. Olfactory-related regions exhibit progressive neurodegeneration in early PD, contributing to worsening cognition and disease severity. Baseline imaging and fluid biomarkers may serve as prognostic tools in PD.

**Supplementary Information:**

The online version contains supplementary material available at 10.1038/s41598-025-19551-0.

## Introduction

Parkinson’s disease (PD) is the second most common neurodegenerative disease and the cause of a major health burden due to its impact on 12 million cases all over the world. By 2021, the prevalence of PD has increased two-fold since 1990, and the age-standardized rates have increased by 22%, owing to the aging of the population and the availability of better diagnostic tools^1^. This increase in the prevalence of PD denotes the importance of the development of reliable diagnostic, prognostic, and therapeutic measures that could be able to not only mitigate the worsening of patients’ quality of life (QoL) but also improve the healthcare system.

PD morbidity is characterized by a variety of motor and non-motor symptoms. Bradykinesia, rigidity, and tremor are the hallmark motor features of PD, whereas the non-motor symptoms, including cognitive impairment, depression, and sleep disturbances, may also impose substantial disability and poor QoL and play a significant role in the disease course^2^. Cognitive decline in PD cases is a widespread issue, with previous studies reporting that up to 80% of individuals develop dementia within 15–20 years of disease onset. This reduces their QoL significantly by increasing the dependency on caregivers^3^.

Parkinson’s disease is an alpha-synucleinopathy, with pathological aggregation of misfolded alpha-synuclein in Lewy bodies and neurites. Braak’s staging hypothesis contends that this pathology initially appears in the olfactory bulb and dorsal motor nucleus of the vagus and then proceeds to midbrain and cortical areas. The early compromise of olfactory structures offers a biological explanation for the pronounced hyposmia in prodromal and early PD^4^.

Indeed, olfactory dysfunction is among the earliest and most prevalent non-motor symptoms experienced by patients with PD, with about 90% of them being affected even before motor symptoms occur^5^. This early occurrence can allow us to consider the impairment in cortical and subcortical olfactory-related regions as an essential biomarker for predicting the course of PD. Olfactory loss has also been proven to be related to the memory impairment and other non-motor signs, and this association may be helpful for early detection of these debilitating conditions^6^. Consistently, longitudinal research indicates that baseline hyposmia in early PD is associated with accelerated cognitive decline^7,8^. Moreover, a recent study reported that olfactory impairment in PD correlates with executive dysfunction and other specific cognitive deficits, further supporting the close link between olfactory network degeneration and cognitive impairment in PD^7^. The olfactory system encompasses several cortical and subcortical brain regions that include the piriform cortex, entorhinal cortex, amygdala, lateral orbitofrontal cortex (lOFC), medial orbitofrontal cortex (mOFC), insular cortex, and thalamus^9–11^. Not only are these regions involved in olfactory processing, but they also contribute to the cognitive and emotional functions. Structural and functional aberrations in these areas are considered to be mainly responsible for the cognitive impairment in both PD and Alzheimer’s disease^6^. For instance, the amygdala and entorhinal cortex are necessary for memory and are thus the most vulnerable regions affected in neurodegenerative processes^12^.

Advanced brain imaging techniques like structural magnetic resonance imaging (MRI) and diffusion tensor imaging (DTI) have been useful in studying various brain structures and clarifying the neurodegenerative process in Parkinson’s disease^13,14^. The volume and thickness of various regions in the brain are shown by the measures of structural MRI. In addition, the microstructural integrity of the brain can be visualized by the DTI metrics such as the fractional anisotropy (FA) and mean diffusivity (MD). Typically, reduced FA and increased MD in olfactory-related regions, such as the thalamus and amygdala, have been associated with the loss of white and grey matter integrity and the spread of α-synuclein pathology, which are hallmark features of PD^13^.

Besides neuroimaging, biomarkers in serum and cerebrospinal fluid (CSF) have achieved a significant level of interest in recent years in terms of the diagnosis and prognosis of PD. Biomarkers such as α-synuclein, amyloid-beta, and tau proteins can help predict how the disease will progress and how cognitive abilities may decline. They also provide valuable information that complements imaging techniques in understanding PD pathophysiology^6^.

Despite these advancements, there is still a lack of longitudinal studies that combine multimodal imaging and biomarker data to predict the disease course in the future, as well as potential disabilities such as cognitive decline and depression in PD patients. Most of the studies have performed cross-sectional analyses, which deprive us of comprehending the dynamics of neurodegeneration over time and better tailoring the treatment approach. Therefore, the purpose of the current study is to determine the longitudinal changes in olfactory-related brain regions of early PD patients over four years. We also investigated the association between the baseline structural MRI and DTI characteristics and biomarkers of these regions and the future cognitive performance and disease severity of PD patients. A novel strength of our study is the combined use of both structural MRI and diffusion tensor imaging to assess complementary macrostructural and microstructural changes, enabling a more comprehensive evaluation of olfactory-related neurodegeneration in early PD.

## Methods

The current study included patients from the Parkinson’s Progression Markers Initiative (PPMI). The PPMI is a global project aimed at establishing biomarkers for Parkinson’s disease^15^. All methods were performed in accordance with the relevant guidelines and regulations, including the Declaration of Helsinki and Good Clinical Practice, as implemented by the PPMI study protocol^15^. Moreover, the study was authorized by regional ethics boards of the PPMI sites, and all patients who agreed to join the trial submitted written informed consent^15^.

### Patient selection

The PPMI comprised PD patients who were aged 30 or older, less than 2 years of diagnosis, had an initial Hoehn and Yahr stage below III, were not on dopamine replacement therapy, and had at least two symptoms from rigidity, resting tremor, or bradykinesia (bradykinesia or resting tremor should be present) or only asymmetrical bradykinesia or resting tremor^15^.

The PD patients who had both 3-dimensional T1-weighted MRI and DTI at baseline, after two years, and also after four years of enrollment, along with complete motor and non-motor (e.g., cognitive) evaluation at these time points, were deemed eligible to enter our study. Accordingly, 97 PD patients were recruited for this study.

### Demographics and clinical assessments

Demographics of the included patients, such as age, gender, ethnicity, education level, and the clinical data encompassing the duration of disease and presence of symptoms like bradykinesia, resting tremor, rigidity, and postural instability at the diagnosis, were recorded.

The overall cognitive status of enrolled cases was examined by the Montreal Cognitive Assessment test (MoCA)^16^. The evaluation of cognition was also completed by additional tests. The immediate and delayed verbal learning and memory were examined by the Hopkins Verbal Learning Total (HVLTR) and Delayed Recall (HVLDR) tests, respectively^17^. The cognitive processing efficiency was examined by the Symbol Digit Modalities test (SDM)^18^. The Benton Judgment of Line Orientation test (JLO) was applied to assess visuospatial perception^19^. Also, focus and cognitive adaptability were examined by the Letter-Number Sequencing test (LNS)^20^. Semantic verbal fluency was assessed using the Semantic Fluency test (SF)^21^. Lastly, the presence of depressive symptoms was evaluated by applying the short form of the Geriatric Depression Scale (GDS)^22^.

Motor examinations comprised the Movement Disorder Society-sponsored version of the Unified Parkinson’s Disease Rating Scale (UPDRS), consisting of UPDRS-Part-I, UPDRS-Part-II, and UPDRS-Part-III^23^.

Initially, PD cases were off specific PD therapies. Nevertheless, PD treatments may be started after registration if they are clinically required^15^. As a result, UPDRS-Part-III examinations were usually performed twice, once while the patients were not taking their normal medicine (OFF) and once when they were (ON). Participants were instructed to discontinue dopaminergic drugs between 6 and 12 h preceding the UPDRS Part-III OFF exams. Following OFF evaluation, subjects were instructed to consume their regular prescription dosage, and another UPDRS Part-III motor evaluation (ON) was carried out in excess of one hour later^15,24^.

Of the 97 enrolled participants, imaging and baseline demographic/clinical characteristics were complete without missing data. Fluid biomarkers at baseline were also largely complete, with approximately 7% missing values; these cases were excluded from the corresponding analyses. Cognitive and most motor assessments were nearly complete across all three time points. The primary exceptions were UPDRS-III (OFF), UPDRS-III (ON), and UPDRS-IV, which had variable availability due to testing conditions in the PPMI protocol. Therefore, analyses of these outcomes were restricted to the available data at each time point. Our longitudinal mixed effects models are well suited to handle this kind of unbalanced data, allowing us to include all participants who had at least one follow-up assessment.

### MRI and DTI acquisition

Patients underwent structural 3D T1-weighted MRI using Siemens 3.0 T scanners in different sites. The scanning parameters of sagittal 3D T1-weighted MRI were as follows: repetition time (TR) = 2,300 ms, time to echo (TE) = 2.52 ms, inversion time = 900 ms, flip angle = 9°, slice number = 176, acquisition matrix = 256 × 256, and voxel size = 0.976 × 0.976 × 1 mm³. The DTI was obtained with the following parameters: TR = 900 ms, TE = 88 milliseconds, flip angle = 90°, voxel size = 2 × 2 × 2 mm³, slice number = 65, and acquisition matrix = 1044 × 1044. Moreover, DTI images included 64 gradient directions with a b-value of 1,000 s/mm² and one non-gradient volume (b = 0 s/mm²).

### Image processing

T1 image segmentation was performed using FreeSurfer version 6.0 (http://surfer.nmr.mgh.harvard.edu). The pipeline has been previously discussed in detail^25^.

The DICOM images were first converted to NIfTI format using MRIcron’s dcm2nii tool (http://www.nitrc.org/projects/mricron). T1-weighted NIfTI images were aligned to Talairach space through a linear registration process to define seed points. Following this, bias field inhomogeneities were corrected, and skull stripping was performed to isolate brain tissue. An initial white matter surface was generated using volumetric tissue classification techniques. Planar partitions derived from Talairach space were utilized to segment the hemispheres. The preliminary white matter surfaces were iteratively refined by propagating them along the intensity gradient to delineate the interfaces between white matter, gray matter, and the pial surface. Finally, cortical surfaces were automatically parcellated via nonlinear surface-based registration, using the Desikan-Killiany atlas as the reference framework^26^.

The generated surfaces and segmentations were carefully examined and rectified as needed. Moreover, the volume and cortical thickness of olfactory-related regions such as the entorhinal cortex, amygdala, lOFC, mOFC, insular cortex, and thalamus were extracted based on the Desikan-Killiany atlas.

DTI images were analyzed employing ExploreDTI version 4.8.6^27^. Signal drift was first corrected using the b = 0 s/mm2 images as a reference. Gibbs ringing artifacts were mitigated, and any visible Venetian blinds artifacts were addressed. Non-brain regions were cropped from both DTI and T1-weighted images to enhance processing efficiency. Subsequent preprocessing included corrections for subject motion and eddy current-induced distortions, with the b-matrix appropriately rotated to maintain alignment. Finally, the DTI data were non-linearly registered to the participant’s T1-weighted anatomical image that had been registered to the MNI-152 space using ANTs^28^ to compensate for geometric distortions inherent to echo planar imaging. The diffusion metrics (FA and MD) of cortical and subcortical olfactory-related regions, including entorhinal cortex, amygdala, lOFC, mOFC, insular cortex, and thalamus, were extracted based on and Desikan-Killiany atlas. Moreover, the diffusion metrics of the olfactory cortex (corresponding to the anterior olfactory nucleus and parts of the piriform cortex) were achieved using the Automated Anatomical Labeling atlas^29^.

### Serum and CSF biomarkers evaluation

All fluid biomarkers were collected at the baseline visit. CSF α-synuclein concentrations were measured using a BioLegend ELISA kit. The Fujirebio-Innogenetics INNO-BIA AlzBio3 immunoassay was used for the analysis of phosphorylated tau (p-Tau181), total tau (t-Tau), and β-amyloid 1–42 (Aβ42) in CSF. The neurofilament-light chain (NfL) protein levels in serum were determined using the Simoa NF-light singleplex assay. Comprehensive details on the collection and preparation of CSF and blood samples are available in the PPMI biologics manual^15^.

### Statistical analysis

The longitudinal analysis of the changes in olfactory-related regions was carried out using the repeated-measure ANOVA (parametric) and the Friedman test (non-parametric), and the between-timepoint differences were detected using post-hoc tests. Moreover, the association between baseline imaging and fluid biomarkers and the changes in cognition and UPDRS scores was examined with Spearman (categorical variables) and Pearson (continuous variables) correlation tests. The changes of clinical scores between two visits were calculated as a percentage of change = ((new score - previous score) / previous score) × 100. The variables with statistically significant correlations were entered into linear regression models in order to test the relationship while controlling for confounding factors, including age, gender, education level, and disease duration. Linear mixed-effect (LME) models were also applied to assess longitudinal relationships between imaging metrics and clinical scores across three time points, controlling for age, gender, education, and disease duration. Linear mixed-effect (LME) models were used to assess the longitudinal relationships between baseline and follow-up imaging and clinical scores across baseline, 2-year, and 4-year visits. In each model, the clinical score was the dependent variable, and a single imaging metric along with time (categorical: baseline, year 2, year 4) were entered as fixed effects. Age, gender, education, and disease duration were included as covariates. Random intercepts for participants were included to account for within-subject correlation over time. All models were fitted using the statsmodels package (Python version 3.9), with false discovery rate (FDR) correction (*p* < 0.05) applied to account for multiple testing. Also, a false discovery rate (FDR) of 0.05 was used to correct for multiple comparisons type 1 errors. All tests were two-sided, and a *p* < 0.05 was deemed significant. We applied false discovery rate (FDR) correction separately for each type of measurement within each modality group (mean diffusivity (MD), fractional anisotropy (FA), volume, cortical thickness, and fluid biomarkers) across all time intervals combined rather than across all measures together. This was our preferred method since correcting all outcomes at once would have been extremely conservative and might have masked significant findings. We conducted analysis with statsmodels package with Python version 3.9 as was mentioned above.

## Results

### Patients’ characteristics

A total of 97 PD patients (64 men and 33 women) were included. The patients were recently diagnosed and had not yet received dopaminergic therapy at baseline. At baseline, the mean (SD) age was 60.94 (9.29) years, and the mean (SD) disease duration was 8.41 (10.03) months. The baseline characteristics of the patients are detailed in Table 1, with CSF and blood biomarkers presented in Table 2. Table 3 summarizes the clinical and cognitive scores at baseline, 2-year, and 4-year follow-up evaluations. Over the 4-year period, UPDRS scores showed a progressive increase across all subscales, indicating a gradual worsening of motor and non-motor symptoms. Cognitive scores were relatively stable, with minor declines noted in HVLTR and SDM tests (e.g., SDM decreased from 41.19 to 39.37). MoCA scores showed a slight reduction by year 2 but partially recovered by year 4.

**Table 1:** Baseline characteristics of parkinson’s patients included in the Study.

Characteristic	Value
Number of Patients	97
Gender (Male/Female)	64/33
Age (at enrollment)	60.94 ± 9.29 (mean ± SD)
Race (White/Black)	95/2
Education (Years)	15.26 ± 2.87 (mean ± SD)
Disease Duration (at enrollment, in months)	8.41 ± 10.03 (mean ± SD)
Handedness (Right/Left/Both)	83/10/4
Tremor at diagnosis (Yes/No)	75/22
Rigidity at diagnosis (Yes/No/Unknown)	81/15/2
Bradykinesia at diagnosis (Yes/No)	89/8
Postural instability at diagnosis (Yes/No/Unknown)	5/91/1

**Table 2:** Fluid biomarkers at Baseline.

Biomarker	Level (mean ± SD)	Number
Serum NfL	12.32 ± 6.09	92
CSF α-synuclein	1446.65 ± 601.68	94
CSF Aβ1–42	848.12 ± 322.56	93
CSF pTau	13.34 ± 4.56	94
CSF tTau	159.89 ± 46.52	91

NfL: neurofilament light chain, CSF: cerebrospinal fluid, Aβ1–42: Beta-Amyloid (1–42), pTau: phosphorylated Tau, tTau: total Tau.

**Table 3:** Clinical scores over the Follow-up period (Mean ± SD).

Measure	Baseline	Second Year	Fourth Year
UPDRS Scores			
UPDRS-1 A	1.19 ± 1.43	1.81 ± 2.17	2.12 ± 2.44
UPDRS-1B	3.95 ± 2.98	5.21 ± 3.43	6.68 ± 3.89
UPDRS-1	5.13 ± 3.77	7.02 ± 4.58	8.80 ± 5.46
UPDRS-2	5.59 ± 4.11	7.62 ± 4.94	9.78 ± 6.54
UPDRS-3 _(OFF)_	21.30 ± 9.64 (*N* = 90)	26.61 ± 12.27 (*N* = 41)	29.41 ± 13.66 (*N* = 66)
UPDRS-3 _(ON)_		20.71 ± 10.79 (*N* = 76)	21.01 ± 13.21 (*N* = 92)
UPDRS-4	0.75 ± 1.75 (*N* = 8)	0.57 ± 1.28 (*N* = 85)	1.58 ± 2.47 (*N* = 93)
Cognitive Scores			
JLO	12.71 ± 2.23	12.79 ± 1.97	12.78 ± 1.94
HVLTR	25.36 ± 5.32	24.31 ± 5.81	24.49 ± 5.95
HVLDR	8.55 ± 2.66	8.56 ± 3.07	8.33 ± 3.35
MoCA	27.48 ± 2.21	26.87 ± 2.62	27.24 ± 2.99
LNS	10.78 ± 2.75	10.46 ± 2.87	10.27 ± 3.18
SF	50.32 ± 11.12	51.36 ± 12.02	49.96 ± 12.89
SDM	41.19 ± 9.79	40.92 ± 10.37	39.37 ± 11.24
GDS	2.17 ± 2.17	2.41 ± 2.72	2.28 ± 2.30

UPDRS: the Unified Parkinson’s Disease Rating Scale, JLO: the Benton Judgment of Line Orientation test, HVLTR: the Hopkins Verbal Learning Total Recall test, HVLDR: the Hopkins Verbal Learning Delayed Recall test, MoCA: the Montreal Cognitive Assessment test, LNS: the Letter-Number Sequencing test, SF: the Semantic Fluency test, SDM: the Symbol Digit Modalities test, GDS: Geriatric Depression Scale (short form).

### Regression analysis of baseline biomarkers and clinical score changes

Baseline biomarkers showed a range of significant correlations with changes in clinical scores over different time periods (supplementary results). Considering the variables with significant correlations, we then developed linear regression models to test whether the baseline imaging and fluid biomarkers have the potential to predict the clinical score changes during the follow-up period.

In the olfactory cortex, baseline FA showed an association with LNS changes (years 0–4; left: β = 0.323; right: β = 0.245), while right FA was associated with SDM changes (years 2–4; β = 0.285). In the amygdala, right FA demonstrated an association with JLO changes (years 2–4; β = 0.275). In the lOFC, left FA was associated with HVLDR changes (years 0–4; β = 0.261) and LNS changes (years 0–4; β = 0.338), and right FA was associated with LNS (years 0–4; β = 0.277) and JLO changes (years 2–4; β = 0.294). In the mOFC, baseline FA was associated with LNS changes over the first two years (years 0–2; left: β = 0.323; right: β = 0.344), and the right FA was associated with LNS changes over the four years (years 0–4; β = 0.294).

**Fig. 1:**
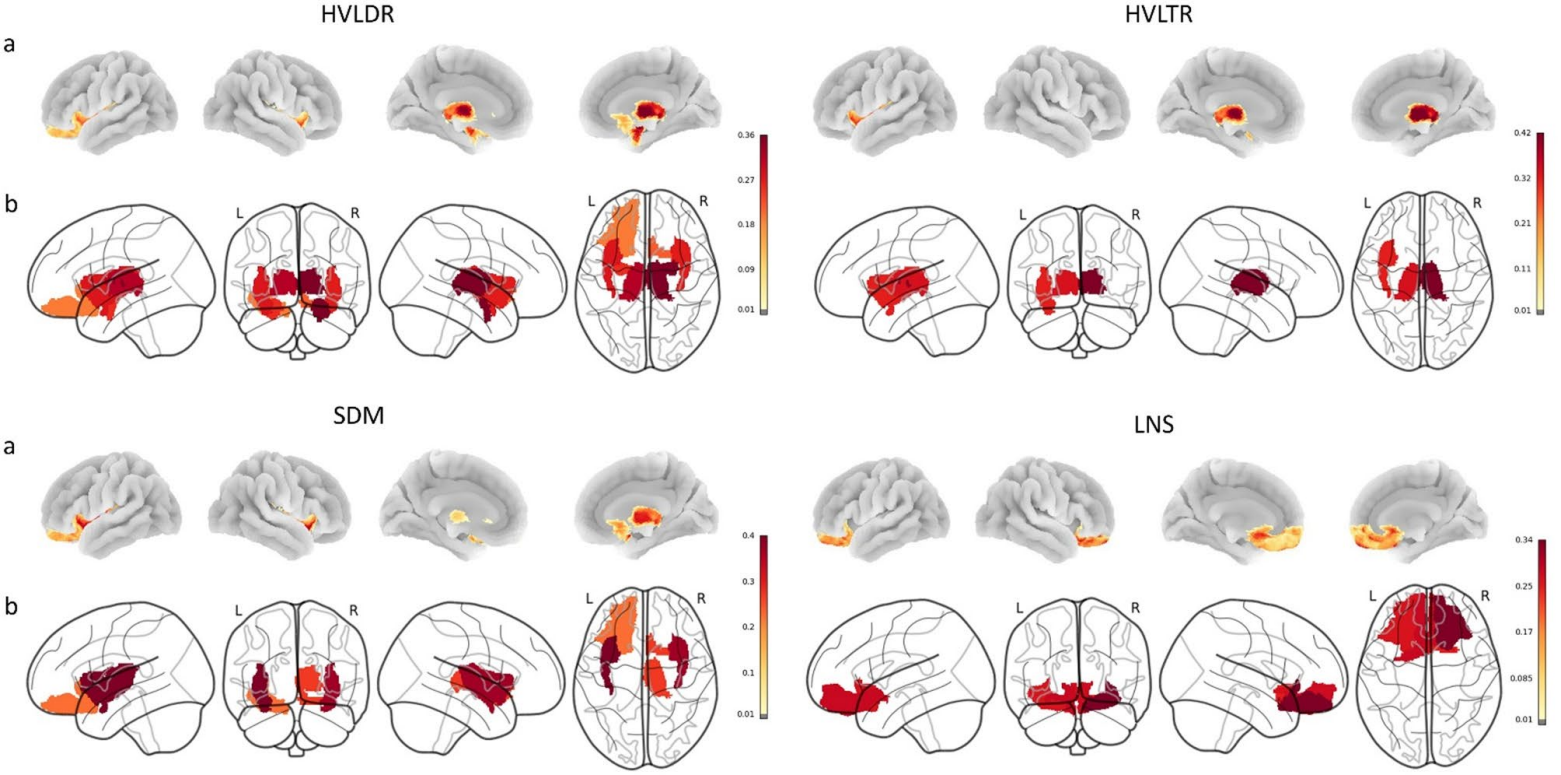
Structural and diffusion imaging changes in olfactory related brain regions over four years in Parkinson’s disease. The figure illustrates significant changes in brain regions (fractional anisotropy [FA] and mean diffusivity [MD]) in key olfactory parts, including the orbitofrontal cortex (OFC), entorhinal cortex, insula, amygdala, and thalamus. Color intensity reflects the magnitude of standardized β coefficients with warmer colors indicate regions showing cortical thinning or volume loss, while cooler colors reflect increased MD or reduced FA, consistent with progressive microstructural degeneration. HVLDR: the Hopkins Verbal Learning Delayed Recall test, HVLTR: the Hopkins Verbal Learning Total Recall test, SDM: the Symbol Digit Modalities test, LNS: the Letter-Number Sequencing test, a: surface view, b: glass brain view, R: right, L: left.

In the amygdala, baseline MD was significantly associated with JLO changes (years 2–4; left: β = − 0.304; right: β = − 0.214), and MD in the right amygdala was associated with HVLDR changes (years 0–4; β = − 0.361). Both hemispheres in the lOFC demonstrated baseline MD association with LNS changes (years 0–4; left: β = − 0.263; right: β = − 0.339). In the mOFC, MD in both hemispheres was significantly associated with LNS changes (years 0–4; left: β = − 0.283; right: β = − 0.276). The insula showed baseline MD significant association with HVLDR changes (years 0–2; right: β = − 0.271 and years 0–4; left: β = − 0.276; right: β = − 0.268), along with significant negative association with SDM changes (years 0–4; left: β = − 0.399; right: β = − 0.370) and HVLTR changes (years 0–2; left: β = − 0.359; right: β = − 0.405). In the thalamus, MD was associated with HVLDR changes (years 0–2; right: β = − 0.337 and years 0–4 left: β = − 0.319; right: β = − 0.361), with additional significant negative association observed for HVLTR (years 0–4; right: β = − 0.422) and SDM changes (years 2–4; left: β = − 0.323; right: β = − 0.337). Figure 1 demonstrates the associations between baseline MD of olfactory-related regions and subsequent changes in different cognitive scores over four years. (Full data are available in the supplementary tables.)

Baseline structural measures also emerged as significant predictors in the regression analyses. In the amygdala, left volume was significantly associated with UPDRS-2 changes (years 2–4; β = 0.382), while right volume was associated with HVLDR changes (years 0–2; β = 0.264). The thickness of the right entorhinal cortex was associated with changes in HVLTR during the second two years (β=-0.348). In the lOFC, left hemisphere thickness showed a significant association with SF score changes (years 0–2; β = 0.402). Moreover, in the mOFC, left thickness was associated with SF changes (years 0–2; β = 0.333).

Table 4 summarizes the cognitive change related to a one SD change in each significant imaging biomarker. For clarity, cognitive changes are expressed in both standardized units and approximate raw test scores (based on the baseline SD of each test). Baseline cognitive score distributions (Mean ± SD) are provided in Table 3.

**Table 4:** Cognitive decline or change per 1 SD increase in baseline imaging biomarkers. Negative values indicate worse performance (decline), whereas positive values indicate better performance (less decline).

Baseline Biomarker (↑1 SD)	Cognitive Outcome (years)	Change in Outcome per 1 SD	Approx. Raw Change
Amygdala MD (right)	HVLDR (0–4 yr)	–0.36 SD (decline)	≈ − 1.0 point (of 12)
Thalamus MD (right)	HVLTR (0–4 yr)	–0.42 SD (decline)	≈ − 2.2 points (of 36)
Insula MD (left)	SDM score (0–4 yr)	–0.40 SD (decline)	≈ − 3.9 points
OFC MD (lat. OFC, right)	LNS score (0–4 yr)	–0.34 SD (decline)	≈ − 0.9 points
Amygdala Volume (right)	HVLDR (0–2 yr)	+ 0.26 SD (less decline)	≈ +0.7 points (of 12)
Entorhinal Thickness (right)	HVLTR (2–4 yr)	+ 0.35 SD (less decline)	≈ +1.9 points (of 36)
OFC Cortical Thickness (left lat. OFC)	Semantic Fluency (0–2 yr)	+ 0.40 SD (improvement)	≈ +4.5 words

SD: standard deviation, MD: Mean diffusivity, OFC: Orbitofrontal cortex.

Baseline analyses showed that serum and CSF NfL levels were strongly associated with cognitive decline, with effects evident across both early (0–2 years) and later (2–4 years) intervals as well as cumulatively over 0–4 years.

Serum NfL: Higher baseline levels predicted poorer memory outcomes on HVLDR (β = − 0.430, over 0–4 years; β = − 0.541, for 0–2 years), HVLTR (β = − 0.425, over 0–4 years; β = − 0.603, for 0–2 years), and JLO (years 2–4; β = − 0.331).

CSF NfL: Similarly associated with declines in HVLDR (β = − 0.216, over 0–4 years) and HVLTR (β = − 0.403, over 0–4 years).

These associations persisted after adjustment for demographic covariates and remained significant after FDR correction. In contrast, CSF α-synuclein, p-tau, total tau, and Aβ42 showed no significant longitudinal associations with either cognitive or motor outcomes at any interval. Additional details are also summarized in eTable 2 (supplementary).

### Longitudinal analysis of brain regions

**Fig. 2:**
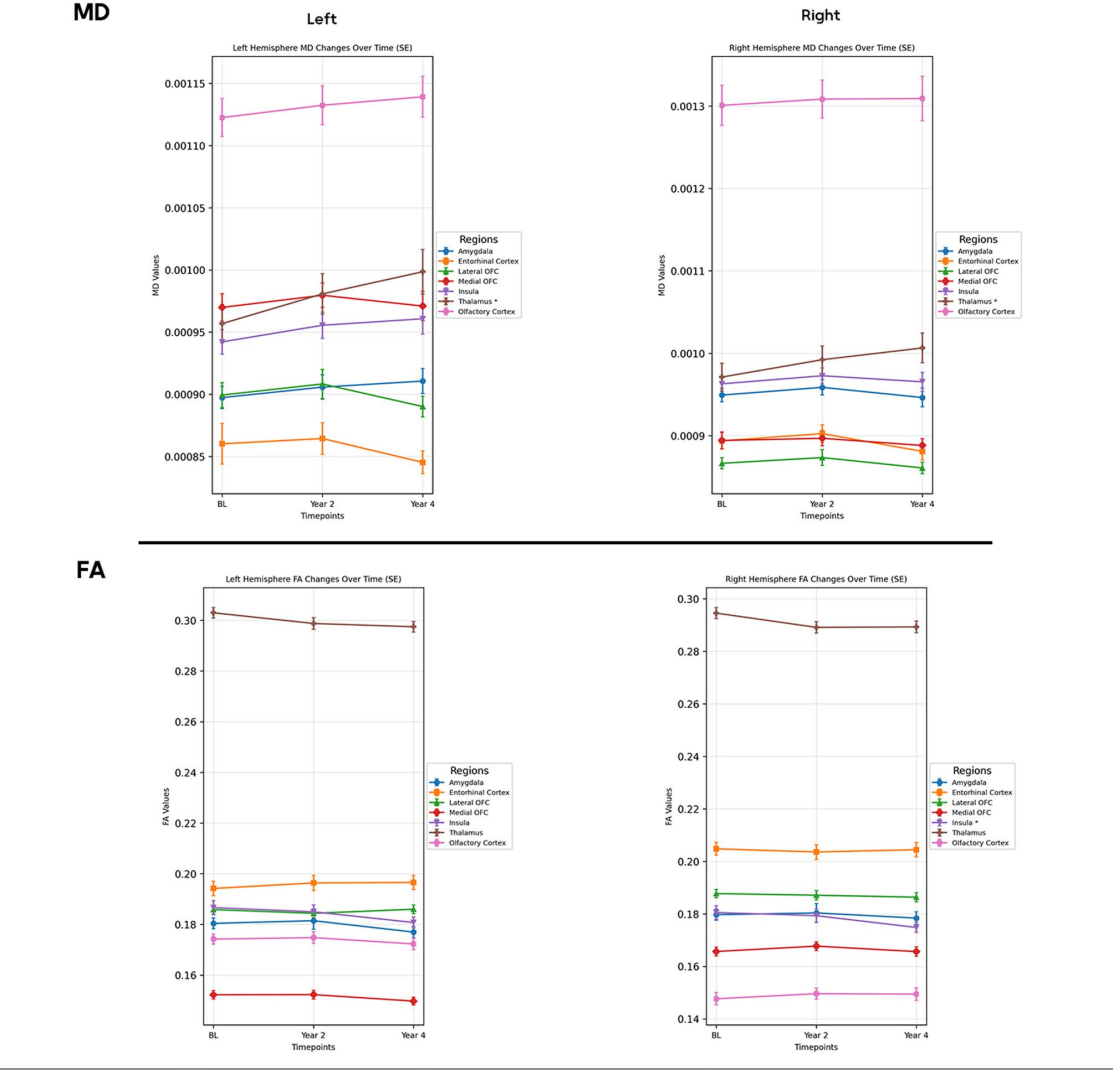
The longitudinal changes of diffusion metrics over four years. MD: mean diffusivity, FA: fractional anisotropy, BL: baseline, OFC: orbitofrontal cortex, *: FDR-adjusted p-value < 0.05. Error bars represent standard error (SE).

Assessment of longitudinal changes revealed a significant FA decrease in the right insular cortex (FDR-adjusted *p* = 0.016), from baseline to the fourth year (post-hoc *p* = 0.0011). The thalamus demonstrated bilateral MD increment (left FDR-adjusted *p* < 0.001, right FDR-adjusted *p* = 0.013), with persistent increase in the left thalamus across all time points (post-hoc *p* < 0.001) and in the right thalamus from baseline to the second year (post-hoc *p* = 0.002) and from baseline to the fourth year (post-hoc *p* < 0.001) (Fig. 2).

**Fig. 3:**
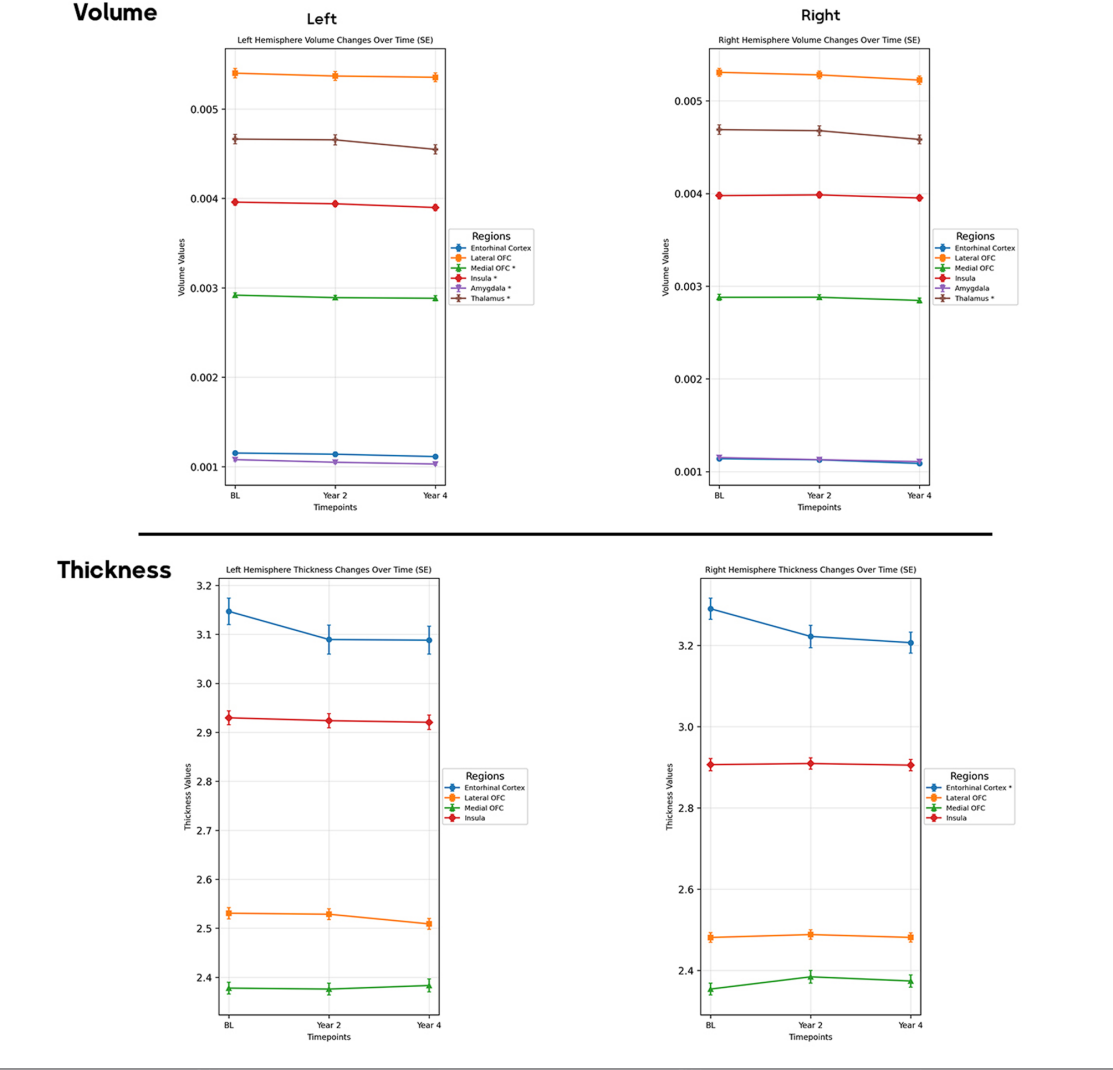
The longitudinal changes of structural metrics over four years. BL: baseline, OFC: orbitofrontal cortex, *: FDR-adjusted p-value < 0.05. Error bars represent standard error (SE).

Volumetric analysis showed significant reductions in the right entorhinal cortical thickness (FDR-adjusted *p* = 0.006), with reductions from baseline to the second year (post-hoc *p* < 0.001) and from baseline to the fourth year (post-hoc *p* < 0.001). Reductions in the left amygdala (FDR-adjusted *p* = 0.013) from baseline to the second year (post-hoc *p* = 0.005), from baseline to the fourth year (post-hoc *p* < 0.001), and between the second and fourth years (post-hoc *p* = 0.021) were significant. The left mOFC exhibited a significant volume decrease (FDR-adjusted *p* = 0.015), particularly from baseline to the fourth year (post-hoc *p* = 0.004). The left insular cortex also showed a significant volume decrease (FDR-adjusted *p* = 0.008), from baseline to the fourth year (post-hoc *p* = 0.002) and from the second to the fourth year (post-hoc *p* = 0.003). Lastly, both thalami demonstrated significant volume loss (FDR-adjusted *p* < 0.001), with reductions from baseline to the fourth year and from the second to the fourth year (post-hoc *p* < 0.001) (Fig. 3). Additional results are also demonstrated in eTable 4 (supplementary).

### Linear Mixed-Effect model

Higher FA in the left thalamus was significantly associated with improved JLO scores across all three time points (β = 25.068). Moreover, reduced cortical thickness in the left entorhinal cortex was associated with poorer SDM scores across all time points (β = 7.92).

## Discussion

In this four-year longitudinal study of PD patients, we found significant neurodegenerative changes in olfactory-related cortical regions associated with cognitive decline. DTI imaging revealed that higher MD in the olfactory cortex, entorhinal cortex, amygdala, lateral and medial OFCs, insula, and thalamus, is associated with declines in at least one of the cognitive domains.

Our use of two-year intervals in addition to the full four-year span was intentional. While the overall trajectory of PD progression is well recognized, shorter interval analyses can show patterns that are for a precise stage, such as periods of relative stability followed by accelerated decline. Identifying such temporal patterns may highlight critical therapeutic windows for early intervention. This approach, to our knowledge, has not been applied in prior longitudinal imaging studies of PD and therefore represents an innovative aspect of our design.

After the follow-up, declines in FA were starkly lateralized in the right insula, and rises in MD in the thalamus were significant bilaterally. All other regions did, however, exhibit crescendo trends in MD values, but not to a level that is statistically significant. Besides the microstructural changes, macrostructural changes demonstrated a significant amount of volume loss in both thalami, and the left medial OFC, insula, and amygdala. Moreover, the right entorhinal cortex indicated cortical thinning. Right amygdala atrophy specifically correlated with cognitive deterioration, while left amygdala atrophy related more strongly to increased motor symptom severity as measured by UPDRS. Thinning of the left medial and lateral OFCs was significantly associated with poorer semantic fluency, and left entorhinal cortex thinning predicted reduced processing speed. Also, baseline fluid biomarker assessments showed that elevated serum and CSF NfL levels corresponded with subsequent cognitive decline. These multimodal findings collectively support the predictive value of combined micro- and macrostructural imaging and fluid biomarkers in monitoring disease progression and managing PD patients.

The adjusted beta values have meaningful information as well. For example, the − 0.422 β value for right thalamus MD predicting HVLTR indicates that a one SD increase in thalamic MD is related to a 0.42 SD reduction in recall performance over 4 years, equal to recalling 2 fewer words out of 36. Similarly, individuals with greater baseline right amygdala MD had about 0.36 SD decline in HVLDR (≈ 1 fewer word out of 12), whereas increased left lateral OFC thickness predicted greater semantic fluency (β = +0.40; ≈4–5 more words in 1 min). These results highlight that 1 SD magnitude structural MRI changes can lead to detectable cognitive differences over time(Full data are available in the supplementary tables).

In quantifying these clinical changes, we used percentage change relative to baseline to normalize across different scales (e.g., UPDRS and MoCA) and make a direct comparison of motor and cognitive parts easier. Although raw score changes are more commonly reported in PPMI studies, prior research has used this approach to compare progression rates across different clinical measures, supporting its importance in our framework^30–34^.

As Braak’s hypothesis states, PD pathology starts in the brainstem and anterior olfactory nucleus, progressing to cortical regions, ultimately leading to cognitive deficits through widespread alpha-synuclein aggregation into Lewy bodies and Lewy neurites^4^. Alpha-synuclein pathology is notably present throughout olfactory cortical regions, including the piriform cortex, entorhinal cortex, amygdala, and OFCs^35,36^. Importantly, these olfactory connected neuropils also represent critical nodes for higher order cognitive operations, including memory, attention, and executive control^37,38^. Accordingly, degeneration in these areas may link olfactory impairment to later cognitive impairment^4^. Emphasizing olfactory-related neuropils thus represents a coherent approach to examining herald sensory markers that can sign subsequent cognitive impairment, rather than implying that olfaction is the exclusive function of these areas.

Another critical mechanism involves disruptions in neurotransmitter systems, particularly dopaminergic, cholinergic, noradrenergic, and serotonergic pathways^39^. Dopaminergic interneurons are integral to olfactory processing, shaping odor discrimination and perception, and reduced dopamine receptor availability in the insula has been linked to poorer executive function, emphasizing its role in integrating olfactory information with higher cognitive processes^40^. Cholinergic neuronal loss, particularly in the basal forebrain, coincides with alpha-synuclein deposition and cognitive impairment^39,41^. Serotonergic and noradrenergic pathways further modulate olfactory cortex function, suggesting their disruption contributes to cognitive decline^39,42^. Additionally, connectivity disruptions between regions commonly involved in olfactory processing and cognition may contribute to cognitive impairment in PD. Specifically, the entorhinal cortex acts as a gateway to the hippocampus, supporting memory encoding related to odor information^43^. Moreover, the amygdala’s connectivity with lateral and medial OFCs integrates sensory input with emotional context, and disruptions in these circuits have been linked to both olfactory dysfunction and cognitive deterioration^44^.

Specific regional changes highlight distinct pathological trajectories. The thalamus showed biphasic degeneration, initially characterized by more pronounced changes in the slope of MD, followed by significant volumetric loss. This progression may reflect an initial stage of compensatory synaptic plasticity and axonal reorganization that eventually gives way to neuronal apoptosis and synaptic pruning, leading to irreversible tissue loss. The thalamus’s vulnerability could be attributed to its role as both a hub for olfaction and cognition. Specifically, the mediodorsal thalamic nucleus is the primary thalamic site for olfactory representation and receives direct input from the piriform cortex and has reciprocal connections with the OFC, involved in higher-order cognitive functions^45^.

The right entorhinal cortex exhibited rapid cortical thinning followed by slower decline, possibly indicating initial compensatory mechanisms preceding irreversible damage. Previous studies also reported that the entorhinal cortex may exhibit early and disproportionate atrophy in PD patients progressing towards dementia^46^. Progressive cortical thinning across regions such as the entorhinal cortex, temporal pole, and prefrontal cortices parallels cognitive deterioration^47^. Moreover, LME modeling revealed that reduced cortical thickness in the left entorhinal cortex was significantly associated with poorer SDM scores. This finding aligns with previous reports showing associations between entorhinal thickness and SDM scores in other neurodegenerative diseases, indicating that entorhinal integrity may buffer against the cognitive effects of underlying pathology^48,49^. The entorhinal cortex, situated at the interface between the neocortex and hippocampus, is essential for memory encoding, attention, and rapid information processing, key functions underlying performance on speeded cognitive tasks such as the SDM^48^.

Among the evaluated regions, only the left insular cortex showed a decrease in FA during longitudinal follow-up. Moreover, our results exhibited that lower baseline FA in the insular cortex, olfactory cortex, amygdala, lateral and medial OFCs may be associated with a subsequent decrease in cognitive scores. However, as previous studies argued, all these findings should be interpreted with caution, as cortical FA measurements are influenced by crossing fibers, which can paradoxically increase FA^50^. Moreover, gray matter typically lacks directional diffusion, limiting the reliability of FA as a marker in cortical regions^50,51^.

DTI studies consistently indicate microstructural degeneration in white-matter tracts and cortical regions related to olfaction, reflected by reduced FA and increased MD, reflecting loss of fiber integrity and neuronal health​^52,53^. Specifically, alterations have been detected in central olfactory regions, such as the left entorhinal cortex and right OFC, highlighting their vulnerability to early degeneration^52^. According to a recent systematic review and meta-analysis, brain regions consistently implicated in olfactory dysfunction among PD patients include significant microstructural alterations in the primary olfactory cortex and gyrus rectus^54^. In early PD cohorts, patients with mild cognitive impairment show considerably lower odor identification scores alongside pronounced DTI changes in olfactory structures compared to cognitively normal PD patients^55^. Also, independent cross-sectional studies have found that olfactory impairment in PD correlates with poorer executive function and memory performance^7,8^. These findings indicate that severe hyposmia in PD patients is likely accompanied by pronounced microstructural degradation in olfactory pathways, serving as a potential early indicator of cognitive decline. Thus, evaluating both DTI metrics in olfactory regions and olfactory function tests could facilitate the identification of PD patients at higher risk for progression to dementia​^55^.

Serum and CSF NfL have been established as biochemical predictors and a significant indicator of cognitive decline. These are likely related to a decline in cognitive function, as easily picked up by HVLTR and HVLDR tests. Based on previous studies, CSF NfL was identified as a potential biomarker in the early phase of the disease, reflecting both the underlying disease severity and an elevated risk of future mortality^56^. Higher baseline CSF NfL levels were significantly correlated with lower cognitive performance, including the mini-mental state examination and MoCA scores^56^. Similarly, elevated plasma NfL levels in PD patients correlate with poorer cognitive scores and an increased risk of cognitive impairment^57^. Increased NfL levels signify an active process of axonal injury rather than isolated neuronal loss. As a critical cytoskeletal element of large-caliber myelinated axons, NfL release into extracellular spaces highlights structural damage and axonal degeneration^56^. Wang et al.^58^ argued that the baseline subcortical MD values may be associated with serum NfL, CSF α-synuclein, β-amyloid, and tau protein levels in PD patients. They also reported that baseline MD values are likely associated with the annual rate of change in the cognitive and UPDRS scores. Although our study investigated the relationship between structural and diffusional imaging along with fluid biomarkers with subsequent clinical changes, in accordance with Wang et al.‘s study^58^, the current study concludes that baseline fluid biomarkers (exclusively NfL levels) and imaging can both potentially predict the clinical and cognitive score changes during the course of PD.

The previous studies argued that tau pathology has also been observed in the anterior olfactory nucleus in PD^59^. Although our study could not find meaningful associations between other fluid biomarker levels (i.e., tau, α-synuclein, and Aβ42) and cognitive and clinical deterioration in PD patients. Future studies should integrate conventional and positron emission tomography (PET) imaging to determine whether such associations exist and if vulnerability in these regions reflects synergistic α-synuclein and Alzheimer’s co-pathology^60^.

This study demonstrates the utility of combining neuroimaging with fluid biomarkers to monitor disease progression and predict cognitive decline. The imaging biomarkers can be applied clinically for early detection of patients at heightened risk of cognitive impairment or rapid disease progression. These markers may guide clinicians in personalized decision-making, aiding in earlier interventions or adjustments to therapeutic strategies, thus potentially delaying or eliminating severe cognitive impairment. Furthermore, shedding light on the connection between elevated serum and CSF NfL levels and the progression of cognitive symptoms emphasizes the clinical significance of NfL as a biomarker in the regular monitoring of axonal degeneration, therapeutic response, and the stage of the disease. Therefore, the inclusion of these biomarkers with multiple modalities in the clinical setting would lead to better prognostic results, targeted medical interventions, and better patient outcomes.

Our study has several limitations that warrant consideration. First, the potential confounding effects of dopaminergic and other symptomatic medications initiated after baseline assessments were not fully controlled during the follow-up period. Second, while the study utilized a comprehensive multimodal biomarker approach, incorporating tau-PET imaging in future research could further elucidate whether the vulnerability of specific cortical regions reflects synergistic contributions from α-synuclein and Alzheimer’s-related pathology. Third, although DTI provides valuable insights into microstructural integrity, it does not directly reflect specific cellular changes such as synaptic density or neuroinflammatory processes, which could be clarified by advanced MRI modalities or PET imaging targeting specific cellular markers. Fourth, although UPSIT is a widely used tool for quantifying olfactory dysfunction in PD, we did not include UPSIT scores in this analysis due to extensive missing data within the longitudinal imaging subset. Finally, the sample size may have limited statistical power to detect subtle longitudinal changes in certain regions, potentially leading to underestimation of smaller yet clinically relevant alterations. Future research incorporating these areas would further clarify neurotransmitter-specific contributions to cognitive and motor symptom progression, thereby providing deeper mechanistic insights and improving therapeutic targeting.

## Conclusion

This longitudinal multimodal study highlights the significant structural and microstructural degeneration of olfactory-related cortical regions in PD, which is associated with cognitive decline over a four-year period. Our findings show the value of MD, volumetric, and cortical thickness measurements, combined with serum and CSF NfL levels, as predictive biomarkers of cognitive deterioration. These neuroimaging and fluid biomarkers could aid in earlier detection, improved monitoring, and tailored interventions for cognitive impairment in PD. Further studies should aim to clarify underlying pathophysiological mechanisms and expand biomarker profiles to enhance early diagnosis, track disease progression, and facilitate the development of targeted therapeutic strategies.

## Supplementary Information

Below is the link to the electronic supplementary material.

Supplementary Material 1

## Data Availability

The current study included patients from PPMI. The PPMI is a global project aimed at establishing biomarkers for Parkinson’s disease. The data used in this research are publicly available at https://www.ppmi-info.org/.

## Abbreviations

PD: Parkinson’s disease
QoL: Quality of life
lOFC: Lateral orbitofrontal cortex
mOFC: Medial orbitofrontal cortex
MRI: Magnetic resonance imaging
DTI: Diffusion tensor imaging
FA: Fractional anisotropy
MD: Mean diffusivity
CSF: Cerebrospinal fluid
PPMI: Parkinson’s progression markers initiative
UPDRS: The unified Parkinson’s disease rating scale
JLO: The Benton judgment of line orientation test
HVLTR: The Hopkins verbal learning total recall test
HVLDR: The Hopkins verbal learning delayed recall test
MoCA: The montreal cognitive assessment test
LNS: The letter-number sequencing test
SF: The semantic fluency test
SDM: The symbol digit modalities test
GDS: Geriatric depression scale (short form)
TR: Repetition time
TE: Time to echo
NfL: Neurofilament light chain
CSF: Cerebrospinal fluid
Aβ1–42: Beta-Amyloid-(1–42)
pTau: Phosphorylated tau
tTau: Total tau
LME: Linear mixed-effect
FDR: False discovery rate
PET: Positron emission tomography
