# Supplementary material for: Structural and diffusion imaging in olfactory-related brain regions in Parkinson’s disease: predictors of clinical progression
Source: Sci Rep. 2025 Oct 13;15:35636. doi: 10.1038/s41598-025-19551-0 (PMC12518524; doi:10.1038/s41598-025-19551-0)
Supplement: Supplementary file 1 — Supplementary Material 1 [file 41598_2025_19551_MOESM1_ESM.docx]

**Supplementary Results**

**Correlations Between Baseline Biomarkers and Changes in Clinical Scores**

In the amygdala, the left FA correlated positively with JLO changes (years 2–4; r = 0.271), and the right FA correlated positively with JLO (years 2–4; r = 0.356) and HVLDR changes (years 2–4; r = 0.243). In the lOFC, FA correlated positively with JLO changes (years 0-4; left: r = 0.266; right: r = 0.379) and with LNS changes (years 0-4; left: r = 0.309; right: r = 0.287); additionally, the left hemisphere FA correlated positively with HVLDR changes (years 0-4; r = 0.315). In the mOFC, FA correlated positively with LNS changes (years 0-2; left: r = 0.299; right: r = 0.308) and showed similar changes for right FA over the four years (years 0-4; r = 0.275). In the insula, FA in both hemispheres correlated positively with JLO changes (years 2-4; left: r = 0.270; right: r = 0.308).

MDs showed significant negative correlations with clinical changes. In the olfactory cortex, MD correlated negatively with HVLDR, LNS, JLO, and SDM changes (years 0–4; r = –0.227 to –0.314). In the amygdala, MD correlated negatively with HVLDR changes over the four years (years 0–4; left: r = –0.300; right: r = –0.406) and also during the first 2 years (years 0–2; left: r = –0.239; right: r = –0.283); additionally, MD in the right amygdala correlated negatively with HVLTR changes (years 0-2 and years 0–4; r = –0.273 to –0.258). In the lOFC, MD correlated negatively with HVLDR, LNS, and SDM changes (years 0–4; r = –0.231 to –0.334). In the mOFC, MD correlated negatively with LNS changes (years 0–4; left: r = –0.311; right: r = –0.283). In the insula, MD correlated negatively with HVLTR changes (years 0–4; r = –0.309 to –0.367), with SDM changes (years 0–4; left: r = –0.391; right: r = –0.362), and with JLO changes (years 0–4; r = –0.312). In the thalamus, MD correlated negatively with HVLDR, HVLTR, JLO, LNS, and SDM (years 0–4; r = –0.227 to –0.419).

Measures of volume and cortical thickness also revealed significant correlations**.** In the amygdala, right volume correlated positively with HVLDR changes (years 0–2; r = 0.297) and with HVLTR changes (years 0–4; r = 0.273), while left volume correlated positively with UPDRS-2 changes (years 2–4; r = 0.301). In the mOFC, left cortical thickness correlated positively with SF changes (years 0–2; r = 0.259). In the insula, right volume correlated positively with HVLTR changes (years 0–4; r = 0.271). In the thalamus, volume correlated positively with HVLDR changes (years 0–4; left: r = 0.312; right: r = 0.289). Significant negative correlations were observed for entorhinal thickness with HVLTR changes between the second and fourth years (r=-0.345).

Among the baseline fluid biomarkers, serum NfL correlated negatively with changes in HVLDR, HVLTR, and JLO scores (years 0–4; r = –0.297 to –0.421). CSF NfL also correlated negatively with changes in HVLDR (years 0–4; r = –0.357) and HVLTR (years 0–2; r = –0.378). Further details and additional findings are provided in eTable 1.

**Supplementary Tables**

**eTable 1.** The correlations between baseline imaging/fluid biomarkers and changes in clinical measures

| **Feature** | **Target** | **Correlation Type** | **Correlation Coefficient** | **Raw P-value** | **P-value (FDR-corrected)** |
| --- | --- | --- | --- | --- | --- |
| Serum - NfL | HVLDR (Years 0-4) | Pearson | -0.421036229 | 3.98E-05 | 0.000278655 |
| Serum - NfL | HVLTR (Years 0-2) | Pearson | -0.397807878 | 8.61E-05 | 0.000602521 |
| Serum - NfL | JLO (Years 2-4) | Pearson | -0.382998539 | 0.000211871 | 0.001483097 |
| Cerebrospinal Fluid - NFL | HVLTR (Years 0-2) | Pearson | -0.377663281 | 0.008136482 | 0.028477687 |
| Cerebrospinal Fluid - NFL | HVLDR (Years 0-4) | Pearson | -0.356546204 | 0.012865827 | 0.045030394 |
| Cerebrospinal Fluid - a-Synuclein | HVLTR (Years 0-2) | Pearson | -0.329584383 | 0.022162802 | 0.051713206 |
| Serum - NfL | HVLTR (Years 0-4) | Pearson | -0.327119787 | 0.001649489 | 0.01154642 |
| Cerebrospinal Fluid - NFL | HVLDR (Years 0-2) | Pearson | -0.317077619 | 0.028100679 | 0.098352377 |
| Serum - NfL | HVLDR (Years 0-2) | Pearson | -0.307429535 | 0.003033795 | 0.021236565 |
| Cerebrospinal Fluid - CSF Alpha-synuclein | UPDRS 1A (Years 0-2) | Pearson | -0.303411823 | 0.025727124 | 0.180089867 |
| Serum - NfL | JLO (Years 0-2) | Pearson | 0.302386499 | 0.003393486 | 0.023754401 |
| Cerebrospinal Fluid - NFL | HVLTR (Years 0-4) | Pearson | -0.287135162 | 0.047840805 | 0.154635178 |
| Serum - NfL | SDM (Years 0-4) | Pearson | -0.275128395 | 0.008678439 | 0.060749075 |
| Cerebrospinal Fluid - NFL | HVLDR (Years 0-4) | Pearson | -0.274887264 | 0.061485309 | 0.215198581 |
| Cerebrospinal Fluid - a-Synuclein | HVLTR (Years 0-4) | Pearson | -0.267286957 | 0.066272219 | 0.154635178 |
| Cerebrospinal Fluid - a-Synuclein | LNS (Years 0-4) | Pearson | -0.26282267 | 0.071111296 | 0.442484915 |
| Cerebrospinal Fluid - NFL | MoCA (Years 0-4) | Pearson | 0.26049043 | 0.073748431 | 0.516239017 |
| Cerebrospinal Fluid - NFL | HVLDR (Years 0-2) | Pearson | -0.255614947 | 0.082889924 | 0.290114734 |
| Cerebrospinal Fluid - a-Synuclein | LNS (Years 0-2) | Pearson | -0.253458588 | 0.082169652 | 0.575187564 |
| Cerebrospinal Fluid - NFL | SDM (Years 2-4) | Pearson | 0.245091976 | 0.093151346 | 0.264715341 |
| Cerebrospinal Fluid - NFL | UPDRS 1 (Years 2-4) | Pearson | 0.244452996 | 0.101572104 | 0.359875324 |
| Serum - NfL | HVLDR (Years 0-2) | Pearson | -0.241163836 | 0.021286988 | 0.098352377 |
| Cerebrospinal Fluid - a-Synuclein | UPDRS 2 (Years 0-2) | Pearson | 0.233991666 | 0.109439031 | 0.766073216 |
| Serum - NfL | SDM (Years 2-4) | Pearson | -0.231540388 | 0.028105412 | 0.196737881 |
| Cerebrospinal Fluid - a-Synuclein | UPDRS 1A (Years 0-2) | Pearson | -0.23091227 | 0.228136577 | 0.725333747 |
| Cerebrospinal Fluid - a-Synuclein | UPDRS 1A (Years 0-4) | Pearson | -0.229566659 | 0.230934511 | 0.633932666 |
| Cerebrospinal Fluid - NFL | UPDRS 1A (Years 2-4) | Pearson | -0.218045897 | 0.247054597 | 0.745941271 |
| Cerebrospinal Fluid - NFL | UPDRS 1 (Years 0-4) | Pearson | 0.214314204 | 0.162423629 | 0.816643519 |
| Serum - NfL | HVLDR (Years 0-4) | Pearson | -0.206556493 | 0.053502386 | 0.215198581 |
| Cerebrospinal Fluid - CSF Alpha-synuclein | HVLDR (Years 0-4) | Pearson | 0.206013534 | 0.048817329 | 0.113907102 |
| Cerebrospinal Fluid - pTau | UPDRS 2 (Years 0-4) | Spearman | 0.202456475 | 0.050354365 | 0.339119138 |
| Cerebrospinal Fluid - NFL | HVLTR (Years 2-4) | Pearson | 0.199979321 | 0.17294944 | 0.322489568 |
| Cerebrospinal Fluid - pTau | JLO (Years 0-2) | Spearman | 0.196393479 | 0.057808316 | 0.202329106 |
| Cerebrospinal Fluid - CSF Alpha-synuclein | SDM (Years 0-4) | Pearson | 0.195926511 | 0.059811789 | 0.20934126 |
| Cerebrospinal Fluid - NFL | UPDRS 1B (Years 0-4) | Pearson | 0.194554655 | 0.211236164 | 0.678526278 |
| Cerebrospinal Fluid - NFL | SDM (Years 0-2) | Pearson | -0.193556658 | 0.187451143 | 0.711532018 |
| Cerebrospinal Fluid - a-Synuclein | HVLDR (Years 0-2) | Pearson | -0.192441039 | 0.190056022 | 0.443464052 |
| Cerebrospinal Fluid - a-Synuclein | UPDRS 1 (Years 0-2) | Pearson | -0.191706467 | 0.212533336 | 0.29754667 |
| Cerebrospinal Fluid - NFL | MoCA (Years 0-2) | Pearson | 0.191423986 | 0.197413229 | 0.971106671 |
| Cerebrospinal Fluid - tTau | UPDRS 1 (Years 0-2) | Spearman | -0.188263635 | 0.077261204 | 0.261753641 |
| Cerebrospinal Fluid - CSF Alpha-synuclein | UPDRS 1A (Years 0-4) | Pearson | -0.183557231 | 0.183972162 | 0.633932666 |
| Cerebrospinal Fluid - NFL | JLO (Years 2-4) | Pearson | -0.182889968 | 0.21341694 | 0.497972861 |
| Cerebrospinal Fluid - pTau | HVLTR (Years 2-4) | Spearman | -0.179775342 | 0.084649843 | 0.322489568 |
| Cerebrospinal Fluid - a-Synuclein | SDM (Years 2-4) | Pearson | 0.179563903 | 0.222003615 | 0.388506327 |
| Cerebrospinal Fluid - CSF Alpha-synuclein | UPDRS 1B (Years 2-4) | Pearson | 0.179106575 | 0.089372997 | 0.515535208 |
| Cerebrospinal Fluid - ABeta 1-42 | UPDRS 1 (Years 0-2) | Pearson | -0.176393175 | 0.102189249 | 0.261753641 |
| Cerebrospinal Fluid - pTau | HVLDR (Years 0-4) | Spearman | -0.175157272 | 0.096781627 | 0.225823796 |
| Cerebrospinal Fluid - tTau | UPDRS 2 (Years 0-4) | Spearman | 0.172250164 | 0.096891182 | 0.339119138 |
| Cerebrospinal Fluid - ABeta 1-42 | HVLTR (Years 2-4) | Pearson | 0.171545373 | 0.103972285 | 0.322489568 |
| Cerebrospinal Fluid - tTau | SF (Years 2-4) | Spearman | -0.171365259 | 0.100505159 | 0.534899782 |
| Cerebrospinal Fluid - pTau | UPDRS 1 (Years 0-2) | Spearman | -0.169552778 | 0.112180132 | 0.261753641 |
| Cerebrospinal Fluid - tTau | UPDRS 1 (Years 2-4) | Spearman | 0.167863103 | 0.111725837 | 0.359875324 |
| Cerebrospinal Fluid - ABeta 1-42 | SDM (Years 2-4) | Pearson | 0.167071665 | 0.113449432 | 0.264715341 |
| Serum - NfL | LNS (Years 0-4) | Pearson | -0.162301368 | 0.126424261 | 0.442484915 |
| Cerebrospinal Fluid - tTau | HVLTR (Years 0-2) | Spearman | -0.1603682 | 0.122579713 | 0.214514498 |
| Cerebrospinal Fluid - pTau | UPDRS 1B (Years 0-2) | Spearman | -0.15716206 | 0.143649405 | 0.453092224 |
| Cerebrospinal Fluid - ABeta 1-42 | UPDRS 1A (Years 0-4) | Pearson | -0.155277988 | 0.271685428 | 0.633932666 |
| Cerebrospinal Fluid - NFL | UPDRS 1B (Years 0-2) | Pearson | 0.15350051 | 0.325730629 | 0.453092224 |
| Cerebrospinal Fluid - ABeta 1-42 | HVLDR (Years 2-4) | Pearson | 0.152262165 | 0.149634066 | 0.501143843 |
| Cerebrospinal Fluid - tTau | HVLTR (Years 0-4) | Spearman | -0.151037169 | 0.148414359 | 0.259725129 |
| Cerebrospinal Fluid - CSF Alpha-synuclein | UPDRS 1 (Years 2-4) | Pearson | 0.150581906 | 0.154232282 | 0.359875324 |
| Serum - NfL | UPDRS 1B (Years 0-2) | Pearson | 0.148510699 | 0.172360169 | 0.453092224 |
| Cerebrospinal Fluid - ABeta 1-42 | UPDRS 1B (Years 2-4) | Pearson | 0.147019651 | 0.169175751 | 0.515535208 |
| Cerebrospinal Fluid - a-Synuclein | HVLDR (Years 2-4) | Pearson | 0.143250058 | 0.331385963 | 0.501143843 |
| Serum - NfL | HVLTR (Years 2-4) | Pearson | 0.141222606 | 0.184279753 | 0.322489568 |
| Cerebrospinal Fluid - ABeta 1-42 | UPDRS 1A (Years 0-2) | Pearson | -0.140519654 | 0.3204126 | 0.725333747 |
| Cerebrospinal Fluid - tTau | JLO (Years 2-4) | Spearman | 0.139968388 | 0.183276284 | 0.497972861 |
| Cerebrospinal Fluid - NFL | JLO (Years 0-2) | Pearson | 0.137981989 | 0.349658327 | 0.81586943 |
| Cerebrospinal Fluid - tTau | JLO (Years 0-4) | Spearman | 0.13744147 | 0.191390235 | 0.743078963 |
| Cerebrospinal Fluid - tTau | UPDRS 1A (Years 2-4) | Spearman | 0.136569218 | 0.281895133 | 0.745941271 |
| Cerebrospinal Fluid - CSF Alpha-synuclein | UPDRS 1 (Years 0-2) | Pearson | -0.136105801 | 0.203445796 | 0.29754667 |
| Cerebrospinal Fluid - a-Synuclein | HVLDR (Years 0-2) | Pearson | -0.13502164 | 0.365524076 | 0.556381762 |
| Cerebrospinal Fluid - a-Synuclein | UPDRS 1B (Years 0-2) | Pearson | -0.134915902 | 0.388364763 | 0.453092224 |
| Cerebrospinal Fluid - a-Synuclein | UPDRS 1B (Years 2-4) | Pearson | 0.133664967 | 0.375836285 | 0.657713498 |
| Cerebrospinal Fluid - NFL | SF (Years 2-4) | Pearson | 0.131568163 | 0.372721679 | 0.534899782 |
| Cerebrospinal Fluid - pTau | JLO (Years 0-4) | Spearman | 0.131265374 | 0.212308275 | 0.743078963 |
| Cerebrospinal Fluid - tTau | UPDRS 1B (Years 2-4) | Spearman | 0.129561874 | 0.22094366 | 0.515535208 |
| Cerebrospinal Fluid - pTau | UPDRS 1A (Years 2-4) | Spearman | 0.126378314 | 0.319689116 | 0.745941271 |
| Cerebrospinal Fluid - CSF Alpha-synuclein | LNS (Years 2-4) | Pearson | -0.12616365 | 0.228185979 | 0.681982625 |
| Serum - NfL | LNS (Years 2-4) | Pearson | -0.125505971 | 0.238526442 | 0.681982625 |
| Cerebrospinal Fluid - a-Synuclein | UPDRS 1 (Years 2-4) | Pearson | 0.124764459 | 0.408724024 | 0.715267042 |
| Cerebrospinal Fluid - NFL | MoCA (Years 2-4) | Pearson | 0.124608067 | 0.403984612 | 0.911109374 |
| Cerebrospinal Fluid - tTau | UPDRS 1B (Years 0-2) | Spearman | -0.124513082 | 0.24774668 | 0.453092224 |
| Cerebrospinal Fluid - a-Synuclein | MoCA (Years 0-4) | Pearson | 0.124403428 | 0.399534636 | 0.699185613 |
| Cerebrospinal Fluid - a-Synuclein | MoCA (Years 0-2) | Pearson | 0.124218254 | 0.405468259 | 0.971106671 |
| Cerebrospinal Fluid - pTau | GDS (Years 0-2) | Spearman | -0.124064501 | 0.233510327 | 0.992597403 |
| Cerebrospinal Fluid - tTau | HVLDR (Years 2-4) | Spearman | 0.124044075 | 0.236159585 | 0.501143843 |
| Cerebrospinal Fluid - pTau | HVLDR (Years 0-2) | Spearman | -0.123322356 | 0.238918318 | 0.556381762 |
| Cerebrospinal Fluid - pTau | SF (Years 0-2) | Spearman | 0.122351939 | 0.240077146 | 0.708688108 |
| Cerebrospinal Fluid - ABeta 1-42 | UPDRS 1B (Years 0-2) | Pearson | -0.122079475 | 0.262821856 | 0.453092224 |
| Cerebrospinal Fluid - CSF Alpha-synuclein | HVLDR (Years 2-4) | Pearson | 0.120868116 | 0.248466481 | 0.501143843 |
| Cerebrospinal Fluid - tTau | UPDRS 2 (Years 2-4) | Spearman | 0.11993676 | 0.257465987 | 0.647513179 |
| Cerebrospinal Fluid - ABeta 1-42 | SDM (Years 0-4) | Pearson | 0.118175377 | 0.264571168 | 0.617332725 |
| Cerebrospinal Fluid - ABeta 1-42 | SF (Years 2-4) | Pearson | -0.118137987 | 0.264723421 | 0.534899782 |
| Cerebrospinal Fluid - pTau | LNS (Years 0-4) | Spearman | 0.117993831 | 0.259978548 | 0.606616611 |
| Serum - NfL | UPDRS 2 (Years 2-4) | Pearson | 0.117776154 | 0.27167133 | 0.647513179 |
| Cerebrospinal Fluid - tTau | GDS (Years 2-4) | Spearman | 0.117656354 | 0.258735166 | 0.917697804 |
| Cerebrospinal Fluid - CSF Alpha-synuclein | SDM (Years 0-2) | Pearson | 0.114747174 | 0.270778308 | 0.711532018 |
| Cerebrospinal Fluid - pTau | UPDRS 1B (Years 0-4) | Spearman | -0.113172328 | 0.29378795 | 0.678526278 |
| Cerebrospinal Fluid - a-Synuclein | HVLDR (Years 0-4) | Pearson | -0.113106248 | 0.444016658 | 0.518019435 |
| Serum - NfL | UPDRS 1 (Years 0-2) | Pearson | 0.111642818 | 0.303246682 | 0.353787796 |
| Cerebrospinal Fluid - ABeta 1-42 | UPDRS 2 (Years 0-2) | Pearson | 0.111028948 | 0.292041661 | 0.995994669 |
| Cerebrospinal Fluid - tTau | UPDRS 1A (Years 0-2) | Spearman | -0.110819607 | 0.425009105 | 0.725333747 |
| Cerebrospinal Fluid - ABeta 1-42 | HVLDR (Years 0-4) | Pearson | 0.110681056 | 0.299023386 | 0.51637419 |
| Cerebrospinal Fluid - NFL | LNS (Years 2-4) | Pearson | -0.108621197 | 0.4624057 | 0.681982625 |
| Cerebrospinal Fluid - CSF Alpha-synuclein | MoCA (Years 0-4) | Pearson | 0.107342214 | 0.305772436 | 0.699185613 |
| Cerebrospinal Fluid - NFL | UPDRS 1A (Years 0-2) | Pearson | -0.106952726 | 0.580803621 | 0.725333747 |
| Cerebrospinal Fluid - tTau | SDM (Years 0-2) | Spearman | -0.106939778 | 0.304942293 | 0.711532018 |
| Cerebrospinal Fluid - ABeta 1-42 | UPDRS 2 (Years 2-4) | Pearson | -0.106301613 | 0.32144962 | 0.647513179 |
| Cerebrospinal Fluid - tTau | HVLDR (Years 0-2) | Spearman | -0.105763785 | 0.312980485 | 0.53551448 |
| Cerebrospinal Fluid - ABeta 1-42 | HVLDR (Years 0-4) | Pearson | 0.103443336 | 0.33192795 | 0.541931558 |
| Cerebrospinal Fluid - ABeta 1-42 | HVLTR (Years 0-4) | Pearson | 0.10019328 | 0.344683937 | 0.482557512 |
| Serum - NfL | UPDRS 1B (Years 0-4) | Pearson | 0.100133498 | 0.358975024 | 0.678526278 |
| Cerebrospinal Fluid - a-Synuclein | UPDRS 1A (Years 2-4) | Pearson | -0.099962882 | 0.599184325 | 0.804773541 |
| Cerebrospinal Fluid - pTau | MoCA (Years 0-4) | Spearman | 0.099806703 | 0.341166549 | 0.699185613 |
| Cerebrospinal Fluid - NFL | UPDRS 1 (Years 0-2) | Pearson | 0.099753309 | 0.519413563 | 0.519413563 |
| Serum - NfL | HVLDR (Years 2-4) | Pearson | 0.099179617 | 0.355114575 | 0.944602604 |
| Serum - NfL | HVLDR (Years 2-4) | Pearson | -0.098597431 | 0.357959888 | 0.501143843 |
| Cerebrospinal Fluid - pTau | SF (Years 2-4) | Spearman | -0.097142623 | 0.354273095 | 0.534899782 |
| Cerebrospinal Fluid - pTau | LNS (Years 2-4) | Spearman | 0.09527694 | 0.363635402 | 0.681982625 |
| Cerebrospinal Fluid - pTau | UPDRS 2 (Years 2-4) | Spearman | 0.095075665 | 0.370007531 | 0.647513179 |
| Cerebrospinal Fluid - pTau | HVLDR (Years 0-4) | Spearman | -0.094775528 | 0.368838707 | 0.51637419 |
| Cerebrospinal Fluid - CSF Alpha-synuclein | SDM (Years 2-4) | Pearson | 0.09394678 | 0.3704024 | 0.51856336 |
| Cerebrospinal Fluid - NFL | HVLDR (Years 2-4) | Pearson | -0.093542044 | 0.527135942 | 0.614991932 |
| Serum - NfL | SF (Years 2-4) | Pearson | -0.093239191 | 0.382071273 | 0.534899782 |
| Cerebrospinal Fluid - a-Synuclein | SF (Years 2-4) | Pearson | 0.092654478 | 0.531080392 | 0.61959379 |
| Cerebrospinal Fluid - pTau | UPDRS 1 (Years 0-4) | Spearman | -0.092383398 | 0.389201667 | 0.816643519 |
| Cerebrospinal Fluid - CSF Alpha-synuclein | GDS (Years 2-4) | Pearson | -0.090080785 | 0.387897112 | 0.917697804 |
| Cerebrospinal Fluid - CSF Alpha-synuclein | HVLDR (Years 0-4) | Pearson | 0.089406249 | 0.399351102 | 0.541931558 |
| Cerebrospinal Fluid - CSF Alpha-synuclein | UPDRS 1B (Years 0-4) | Pearson | 0.089355641 | 0.407725505 | 0.678526278 |
| Cerebrospinal Fluid - ABeta 1-42 | HVLDR (Years 0-2) | Pearson | 0.089271482 | 0.397415544 | 0.556381762 |
| Cerebrospinal Fluid - pTau | GDS (Years 2-4) | Spearman | 0.089063767 | 0.393299059 | 0.917697804 |
| Cerebrospinal Fluid - NFL | UPDRS 1A (Years 0-4) | Pearson | -0.088983604 | 0.64621586 | 0.827609421 |
| Cerebrospinal Fluid - a-Synuclein | SDM (Years 0-4) | Pearson | 0.088240572 | 0.55090878 | 0.960680038 |
| Cerebrospinal Fluid - NFL | SF (Years 0-2) | Pearson | -0.087838315 | 0.552733173 | 0.708688108 |
| Cerebrospinal Fluid - pTau | HVLDR (Years 0-2) | Spearman | -0.086202898 | 0.411305563 | 0.53551448 |
| Serum - NfL | UPDRS 1 (Years 0-4) | Pearson | 0.086069491 | 0.427973764 | 0.816643519 |
| Serum - NfL | SDM (Years 0-2) | Pearson | -0.08589887 | 0.415556584 | 0.727224022 |
| Cerebrospinal Fluid - CSF Alpha-synuclein | MoCA (Years 2-4) | Pearson | 0.083507893 | 0.428703754 | 0.911109374 |
| Serum - NfL | SF (Years 0-4) | Pearson | -0.083478429 | 0.434072103 | 0.861198754 |
| Cerebrospinal Fluid - a-Synuclein | GDS (Years 2-4) | Pearson | -0.082998479 | 0.574905363 | 0.982474676 |
| Cerebrospinal Fluid - pTau | HVLTR (Years 0-2) | Spearman | 0.081812795 | 0.433094795 | 0.588819022 |
| Cerebrospinal Fluid - a-Synuclein | UPDRS 2 (Years 2-4) | Pearson | 0.081740103 | 0.584923271 | 0.682410483 |
| Cerebrospinal Fluid - a-Synuclein | SF (Years 0-2) | Pearson | -0.079828721 | 0.589643398 | 0.708688108 |
| Cerebrospinal Fluid - a-Synuclein | JLO (Years 0-2) | Pearson | -0.07933744 | 0.591942633 | 0.828719686 |
| Cerebrospinal Fluid - a-Synuclein | UPDRS 2 (Years 0-4) | Pearson | 0.078825992 | 0.594340467 | 0.879181794 |
| Cerebrospinal Fluid - pTau | SDM (Years 2-4) | Spearman | -0.078666737 | 0.453533684 | 0.529122631 |
| Cerebrospinal Fluid - ABeta 1-42 | SF (Years 0-4) | Pearson | -0.078066442 | 0.462013585 | 0.861198754 |
| Cerebrospinal Fluid - CSF Alpha-synuclein | HVLDR (Years 0-2) | Pearson | 0.077718456 | 0.459012411 | 0.53551448 |
| Cerebrospinal Fluid - tTau | HVLDR (Years 0-4) | Spearman | -0.077632706 | 0.464512764 | 0.541931558 |
| Cerebrospinal Fluid - NFL | UPDRS 1B (Years 2-4) | Pearson | 0.075867867 | 0.616284438 | 0.862798213 |
| Cerebrospinal Fluid - a-Synuclein | HVLTR (Years 2-4) | Pearson | 0.075694746 | 0.609113411 | 0.710632312 |
| Cerebrospinal Fluid - tTau | UPDRS 1B (Years 0-4) | Spearman | -0.075467679 | 0.484661627 | 0.678526278 |
| Cerebrospinal Fluid - pTau | MoCA (Years 2-4) | Spearman | 0.075282295 | 0.475707996 | 0.911109374 |
| Cerebrospinal Fluid - CSF Alpha-synuclein | UPDRS 2 (Years 2-4) | Pearson | 0.07489819 | 0.48043801 | 0.672613214 |
| Serum - NfL | GDS (Years 0-4) | Pearson | 0.073662581 | 0.485280273 | 0.975169873 |
| Cerebrospinal Fluid - tTau | LNS (Years 2-4) | Spearman | 0.072946207 | 0.487130447 | 0.681982625 |
| Cerebrospinal Fluid - tTau | LNS (Years 0-4) | Spearman | 0.071735212 | 0.494408351 | 0.865214614 |
| Cerebrospinal Fluid - ABeta 1-42 | HVLTR (Years 0-2) | Pearson | -0.070427496 | 0.504702019 | 0.588819022 |
| Cerebrospinal Fluid - a-Synuclein | HVLDR (Years 0-4) | Pearson | -0.070297214 | 0.638685341 | 0.638685341 |
| Cerebrospinal Fluid - tTau | JLO (Years 0-2) | Spearman | 0.070190226 | 0.501428386 | 0.828719686 |
| Cerebrospinal Fluid - CSF Alpha-synuclein | HVLTR (Years 2-4) | Pearson | 0.07006235 | 0.504555286 | 0.706377401 |
| Cerebrospinal Fluid - pTau | UPDRS 1A (Years 0-2) | Spearman | -0.068673714 | 0.62171464 | 0.725333747 |
| Cerebrospinal Fluid - tTau | SF (Years 0-2) | Spearman | 0.068400708 | 0.512426463 | 0.708688108 |
| Cerebrospinal Fluid - tTau | SDM (Years 2-4) | Spearman | 0.065706665 | 0.531473073 | 0.531473073 |
| Cerebrospinal Fluid - NFL | JLO (Years 0-4) | Pearson | -0.064983817 | 0.66079361 | 0.868392676 |
| Cerebrospinal Fluid - CSF Alpha-synuclein | LNS (Years 0-2) | Pearson | 0.064631944 | 0.535986673 | 0.948343701 |
| Cerebrospinal Fluid - CSF Alpha-synuclein | MoCA (Years 0-2) | Pearson | 0.062585029 | 0.551195246 | 0.971106671 |
| Cerebrospinal Fluid - a-Synuclein | JLO (Years 0-4) | Pearson | -0.062533097 | 0.672853104 | 0.868392676 |
| Cerebrospinal Fluid - ABeta 1-42 | JLO (Years 2-4) | Pearson | 0.062516847 | 0.558299305 | 0.957547544 |
| Cerebrospinal Fluid - ABeta 1-42 | GDS (Years 0-2) | Pearson | -0.062074844 | 0.55664802 | 0.992597403 |
| Cerebrospinal Fluid - ABeta 1-42 | SF (Years 0-2) | Pearson | 0.061879914 | 0.55789022 | 0.708688108 |
| Cerebrospinal Fluid - tTau | SF (Years 0-4) | Spearman | -0.061084311 | 0.560800944 | 0.861198754 |
| Serum - NfL | LNS (Years 0-2) | Pearson | -0.057685662 | 0.584934323 | 0.948343701 |
| Cerebrospinal Fluid - ABeta 1-42 | MoCA (Years 2-4) | Pearson | -0.057559523 | 0.589975441 | 0.911109374 |
| Cerebrospinal Fluid - tTau | HVLDR (Years 2-4) | Spearman | 0.05688297 | 0.590177495 | 0.944602604 |
| Cerebrospinal Fluid - CSF Alpha-synuclein | UPDRS 1B (Years 0-2) | Pearson | -0.056249866 | 0.602688531 | 0.602688531 |
| Cerebrospinal Fluid - a-Synuclein | UPDRS 1B (Years 0-4) | Pearson | 0.055918729 | 0.721722682 | 0.842009795 |
| Cerebrospinal Fluid - NFL | GDS (Years 0-2) | Pearson | -0.054931806 | 0.71076641 | 0.992597403 |
| Cerebrospinal Fluid - CSF Alpha-synuclein | SF (Years 0-2) | Pearson | 0.053666134 | 0.60744695 | 0.708688108 |
| Cerebrospinal Fluid - tTau | UPDRS 1A (Years 0-4) | Spearman | -0.053586486 | 0.700354934 | 0.827609421 |
| Cerebrospinal Fluid - NFL | HVLDR (Years 2-4) | Pearson | -0.053106307 | 0.719979742 | 0.944602604 |
| Cerebrospinal Fluid - tTau | UPDRS 1 (Years 0-4) | Spearman | -0.05265404 | 0.624094815 | 0.816643519 |
| Cerebrospinal Fluid - ABeta 1-42 | LNS (Years 0-2) | Pearson | 0.052406425 | 0.619800466 | 0.948343701 |
| Cerebrospinal Fluid - pTau | HVLTR (Years 0-4) | Spearman | -0.052047794 | 0.620262524 | 0.723639612 |
| Serum - NfL | UPDRS 1A (Years 0-4) | Pearson | 0.051400827 | 0.709379503 | 0.827609421 |
| Cerebrospinal Fluid - tTau | HVLDR (Years 0-2) | Spearman | -0.049751792 | 0.635789914 | 0.726198381 |
| Cerebrospinal Fluid - ABeta 1-42 | SDM (Years 0-2) | Pearson | -0.04959693 | 0.638710969 | 0.894195357 |
| Cerebrospinal Fluid - a-Synuclein | UPDRS 1 (Years 0-4) | Pearson | 0.04733767 | 0.760264049 | 0.816643519 |
| Cerebrospinal Fluid - pTau | SF (Years 0-4) | Spearman | 0.047101936 | 0.653907688 | 0.861198754 |
| Cerebrospinal Fluid - tTau | MoCA (Years 0-4) | Spearman | 0.045629136 | 0.664065902 | 0.915492378 |
| Cerebrospinal Fluid - tTau | HVLDR (Years 0-4) | Spearman | 0.044750829 | 0.671873308 | 0.671873308 |
| Cerebrospinal Fluid - ABeta 1-42 | UPDRS 1 (Years 2-4) | Pearson | 0.044195244 | 0.680893568 | 0.930773439 |
| Cerebrospinal Fluid - NFL | LNS (Years 0-4) | Pearson | -0.04371209 | 0.767990576 | 0.880607516 |
| Cerebrospinal Fluid - CSF Alpha-synuclein | UPDRS 1A (Years 2-4) | Pearson | 0.043572835 | 0.732445091 | 0.804773541 |
| Cerebrospinal Fluid - pTau | UPDRS 2 (Years 0-2) | Spearman | 0.043402777 | 0.677870326 | 0.995994669 |
| Cerebrospinal Fluid - a-Synuclein | SDM (Years 0-2) | Pearson | -0.043330486 | 0.769960539 | 0.898287295 |
| Cerebrospinal Fluid - pTau | LNS (Years 0-2) | Spearman | 0.042612929 | 0.683416766 | 0.948343701 |
| Cerebrospinal Fluid - CSF Alpha-synuclein | SF (Years 0-4) | Pearson | 0.042612344 | 0.685062224 | 0.861198754 |
| Cerebrospinal Fluid - a-Synuclein | HVLDR (Years 2-4) | Pearson | 0.040940967 | 0.782328177 | 0.944602604 |
| Cerebrospinal Fluid - tTau | SDM (Years 0-4) | Spearman | -0.040584381 | 0.699313401 | 0.960680038 |
| Cerebrospinal Fluid - ABeta 1-42 | LNS (Years 2-4) | Pearson | -0.040130048 | 0.705670957 | 0.823282783 |
| Cerebrospinal Fluid - CSF Alpha-synuclein | JLO (Years 0-4) | Pearson | -0.039692299 | 0.707171265 | 0.868392676 |
| Serum - NfL | UPDRS 1A (Years 0-2) | Pearson | 0.039642377 | 0.773839789 | 0.773839789 |
| Cerebrospinal Fluid - ABeta 1-42 | GDS (Years 0-4) | Pearson | -0.038742154 | 0.713873687 | 0.975169873 |
| Cerebrospinal Fluid - CSF Alpha-synuclein | HVLTR (Years 0-2) | Pearson | -0.038606794 | 0.71180252 | 0.71180252 |
| Cerebrospinal Fluid - CSF Alpha-synuclein | UPDRS 2 (Years 0-4) | Pearson | -0.038234001 | 0.714464792 | 0.879181794 |
| Cerebrospinal Fluid - ABeta 1-42 | UPDRS 1A (Years 2-4) | Pearson | -0.038020539 | 0.767350328 | 0.804773541 |
| Cerebrospinal Fluid - ABeta 1-42 | MoCA (Years 0-2) | Pearson | 0.037743901 | 0.72243741 | 0.971106671 |
| Cerebrospinal Fluid - CSF Alpha-synuclein | HVLDR (Years 0-2) | Pearson | 0.036798079 | 0.726198381 | 0.726198381 |
| Cerebrospinal Fluid - ABeta 1-42 | UPDRS 1 (Years 0-4) | Pearson | -0.036793079 | 0.73511159 | 0.816643519 |
| Cerebrospinal Fluid - CSF Alpha-synuclein | HVLTR (Years 0-4) | Pearson | 0.034934406 | 0.739556813 | 0.739556813 |
| Cerebrospinal Fluid - ABeta 1-42 | JLO (Years 0-4) | Pearson | 0.034852383 | 0.744336579 | 0.868392676 |
| Cerebrospinal Fluid - pTau | HVLDR (Years 2-4) | Spearman | 0.034793157 | 0.741958375 | 0.944602604 |
| Cerebrospinal Fluid - NFL | GDS (Years 0-4) | Pearson | -0.034400878 | 0.816442921 | 0.975169873 |
| Cerebrospinal Fluid - tTau | MoCA (Years 2-4) | Spearman | 0.033983745 | 0.747758175 | 0.911109374 |
| Cerebrospinal Fluid - CSF Alpha-synuclein | JLO (Years 2-4) | Pearson | -0.033249748 | 0.753029761 | 0.957547544 |
| Serum - NfL | UPDRS 2 (Years 0-4) | Pearson | 0.033206199 | 0.75334289 | 0.879181794 |
| Cerebrospinal Fluid - a-Synuclein | MoCA (Years 2-4) | Pearson | 0.032955334 | 0.825944152 | 0.911109374 |
| Serum - NfL | UPDRS 1A (Years 2-4) | Pearson | 0.032035025 | 0.804773541 | 0.804773541 |
| Cerebrospinal Fluid - NFL | SF (Years 0-4) | Pearson | 0.03141188 | 0.832151114 | 0.861198754 |
| Cerebrospinal Fluid - a-Synuclein | JLO (Years 2-4) | Pearson | 0.031387809 | 0.832277885 | 0.957547544 |
| Cerebrospinal Fluid - tTau | GDS (Years 0-2) | Spearman | -0.029912582 | 0.774727296 | 0.992597403 |
| Cerebrospinal Fluid - CSF Alpha-synuclein | UPDRS 2 (Years 0-2) | Pearson | -0.028052503 | 0.78839694 | 0.995994669 |
| Cerebrospinal Fluid - tTau | MoCA (Years 0-2) | Spearman | -0.027847912 | 0.791028889 | 0.971106671 |
| Cerebrospinal Fluid - NFL | UPDRS 2 (Years 0-4) | Pearson | 0.027321932 | 0.853749448 | 0.879181794 |
| Cerebrospinal Fluid - tTau | HVLTR (Years 2-4) | Spearman | 0.026763524 | 0.798991137 | 0.798991137 |
| Cerebrospinal Fluid - NFL | UPDRS 2 (Years 0-2) | Pearson | 0.026138736 | 0.86001826 | 0.995994669 |
| Cerebrospinal Fluid - a-Synuclein | SF (Years 0-4) | Pearson | 0.025916108 | 0.861198754 | 0.861198754 |
| Cerebrospinal Fluid - CSF Alpha-synuclein | UPDRS 1 (Years 0-4) | Pearson | 0.024926104 | 0.816643519 | 0.816643519 |
| Serum - NfL | GDS (Years 0-2) | Pearson | 0.024554278 | 0.816280747 | 0.992597403 |
| Cerebrospinal Fluid - pTau | UPDRS 1B (Years 2-4) | Spearman | 0.02294233 | 0.829097783 | 0.881072811 |
| Cerebrospinal Fluid - ABeta 1-42 | MoCA (Years 0-4) | Pearson | -0.021085805 | 0.842742141 | 0.915492378 |
| Serum - NfL | GDS (Years 2-4) | Pearson | 0.021006325 | 0.84245623 | 0.982474676 |
| Serum - NfL | SF (Years 0-2) | Pearson | -0.020523589 | 0.846031617 | 0.846031617 |
| Cerebrospinal Fluid - ABeta 1-42 | LNS (Years 0-4) | Pearson | 0.020409762 | 0.847722436 | 0.880607516 |
| Cerebrospinal Fluid - CSF Alpha-synuclein | GDS (Years 0-4) | Pearson | -0.020204217 | 0.846735064 | 0.975169873 |
| Cerebrospinal Fluid - NFL | GDS (Years 2-4) | Pearson | -0.019911055 | 0.893145744 | 0.982474676 |
| Cerebrospinal Fluid - a-Synuclein | GDS (Years 0-2) | Pearson | 0.018531115 | 0.90051268 | 0.992597403 |
| Cerebrospinal Fluid - pTau | HVLDR (Years 2-4) | Spearman | -0.017830018 | 0.865297663 | 0.865297663 |
| Cerebrospinal Fluid - ABeta 1-42 | JLO (Years 0-2) | Pearson | -0.017596748 | 0.867773514 | 0.981697975 |
| Cerebrospinal Fluid - a-Synuclein | GDS (Years 0-4) | Pearson | -0.016449022 | 0.911643633 | 0.975169873 |
| Serum - NfL | UPDRS 1B (Years 2-4) | Pearson | -0.016084924 | 0.881072811 | 0.881072811 |
| Cerebrospinal Fluid - ABeta 1-42 | UPDRS 2 (Years 0-4) | Pearson | 0.016066425 | 0.879181794 | 0.879181794 |
| Cerebrospinal Fluid - a-Synuclein | LNS (Years 2-4) | Pearson | -0.015801213 | 0.915110373 | 0.915110373 |
| Cerebrospinal Fluid - CSF Alpha-synuclein | LNS (Years 0-4) | Pearson | -0.015787476 | 0.880607516 | 0.880607516 |
| Cerebrospinal Fluid - CSF Alpha-synuclein | SF (Years 2-4) | Pearson | 0.01568467 | 0.881379307 | 0.881379307 |
| Cerebrospinal Fluid - pTau | UPDRS 1 (Years 2-4) | Spearman | 0.015475388 | 0.884242114 | 0.930773439 |
| Serum - NfL | MoCA (Years 0-2) | Pearson | 0.014934629 | 0.888260487 | 0.971106671 |
| Cerebrospinal Fluid - pTau | GDS (Years 0-4) | Spearman | -0.014545663 | 0.889334884 | 0.975169873 |
| Cerebrospinal Fluid - NFL | UPDRS 2 (Years 2-4) | Pearson | -0.014447076 | 0.923217113 | 0.923217113 |
| Cerebrospinal Fluid - ABeta 1-42 | UPDRS 1B (Years 0-4) | Pearson | -0.013929369 | 0.898709626 | 0.898709626 |
| Cerebrospinal Fluid - tTau | LNS (Years 0-2) | Spearman | 0.013126558 | 0.900072888 | 0.948343701 |
| Cerebrospinal Fluid - pTau | SDM (Years 0-4) | Spearman | -0.012838798 | 0.902785481 | 0.960680038 |
| Serum - NfL | MoCA (Years 2-4) | Pearson | -0.012003004 | 0.911109374 | 0.911109374 |
| Serum - NfL | MoCA (Years 0-4) | Pearson | 0.011343542 | 0.915492378 | 0.915492378 |
| Cerebrospinal Fluid - pTau | UPDRS 1A (Years 0-4) | Spearman | -0.00963086 | 0.944895965 | 0.944895965 |
| Cerebrospinal Fluid - NFL | LNS (Years 0-2) | Pearson | 0.009604132 | 0.948343701 | 0.948343701 |
| Serum - NfL | UPDRS 1 (Years 2-4) | Pearson | 0.009340229 | 0.930773439 | 0.930773439 |
| Cerebrospinal Fluid - CSF Alpha-synuclein | HVLDR (Years 2-4) | Pearson | -0.008189653 | 0.938241974 | 0.944602604 |
| Cerebrospinal Fluid - ABeta 1-42 | HVLDR (Years 2-4) | Pearson | -0.007428226 | 0.944602604 | 0.944602604 |
| Cerebrospinal Fluid - NFL | SDM (Years 0-4) | Pearson | -0.007308434 | 0.960680038 | 0.960680038 |
| Cerebrospinal Fluid - ABeta 1-42 | HVLDR (Years 0-2) | Pearson | 0.006774832 | 0.949181164 | 0.949181164 |
| Cerebrospinal Fluid - pTau | JLO (Years 2-4) | Spearman | -0.005626644 | 0.957547544 | 0.957547544 |
| Cerebrospinal Fluid - pTau | MoCA (Years 0-2) | Spearman | 0.003807354 | 0.971106671 | 0.971106671 |
| Serum - NfL | JLO (Years 0-4) | Pearson | -0.003610578 | 0.973211509 | 0.973211509 |
| Cerebrospinal Fluid - tTau | GDS (Years 0-4) | Spearman | 0.003253824 | 0.975169873 | 0.975169873 |
| Cerebrospinal Fluid - CSF Alpha-synuclein | JLO (Years 0-2) | Pearson | -0.002398185 | 0.981697975 | 0.981697975 |
| Cerebrospinal Fluid - ABeta 1-42 | GDS (Years 2-4) | Pearson | 0.00232191 | 0.982474676 | 0.982474676 |
| Serum - NfL | UPDRS 2 (Years 0-2) | Pearson | -0.002188204 | 0.983483716 | 0.995994669 |
| Cerebrospinal Fluid - pTau | SDM (Years 0-2) | Spearman | 0.001271865 | 0.990293018 | 0.990293018 |
| Cerebrospinal Fluid - CSF Alpha-synuclein | GDS (Years 0-2) | Pearson | 0.000969921 | 0.992597403 | 0.992597403 |
| Cerebrospinal Fluid - tTau | UPDRS 2 (Years 0-2) | Spearman | -0.000524791 | 0.995994669 | 0.995994669 |
| ctx-rh-lateralorbitofrontal_FA | JLO (Years 2-4) | Pearson | 0.378512204 | 0.000168776 | 0.002362862 |
| Right-Amygdala_FA | JLO (Years 2-4) | Pearson | 0.3558819 | 0.000431182 | 0.003018274 |
| Olfactory_Right_FA | SDM (Years 2-4) | Pearson | 0.33920335 | 0.000771967 | 0.010807532 |
| Right-Amygdala_FA | UPDRS 1A (Years 0-2) | Pearson | 0.314781356 | 0.017085206 | 0.239192878 |
| ctx-lh-lateralorbitofrontal_FA | HVLDR (Years 0-4) | Pearson | 0.31452918 | 0.002018956 | 0.028265388 |
| ctx-lh-lateralorbitofrontal_FA | LNS (Years 0-4) | Pearson | 0.309368932 | 0.002283488 | 0.024137009 |
| ctx-rh-insula_FA | JLO (Years 2-4) | Pearson | 0.30841461 | 0.002492347 | 0.01163095 |
| ctx-rh-medialorbitofrontal_FA | LNS (Years 0-2) | Pearson | 0.308349513 | 0.002120879 | 0.020690506 |
| ctx-lh-medialorbitofrontal_FA | LNS (Years 0-2) | Pearson | 0.298740402 | 0.002955787 | 0.020690506 |
| Olfactory_Left_FA | SDM (Years 2-4) | Pearson | 0.29706761 | 0.003462025 | 0.024234172 |
| Left-Thalamus-Proper_FA | JLO (Years 0-2) | Pearson | -0.293420476 | 0.00353545 | 0.049496297 |
| ctx-rh-lateralorbitofrontal_FA | LNS (Years 0-4) | Pearson | 0.286957742 | 0.004810671 | 0.024137009 |
| Left-Thalamus-Proper_FA | HVLDR (Years 0-4) | Pearson | 0.277211865 | 0.00714271 | 0.07572712 |
| Olfactory_Left_FA | LNS (Years 0-4) | Pearson | 0.276779907 | 0.006622267 | 0.024137009 |
| ctx-rh-medialorbitofrontal_FA | LNS (Years 0-4) | Pearson | 0.275462048 | 0.006896288 | 0.024137009 |
| Olfactory_Right_FA | HVLDR (Years 0-4) | Pearson | 0.273857149 | 0.007565197 | 0.041223285 |
| Olfactory_Right_FA | SDM (Years 0-4) | Pearson | 0.27343365 | 0.007337645 | 0.102727035 |
| Left-Amygdala_FA | JLO (Years 2-4) | Pearson | 0.2713923 | 0.008147513 | 0.022411279 |
| ctx-lh-insula_FA | JLO (Years 2-4) | Pearson | 0.269948242 | 0.008506696 | 0.022411279 |
| ctx-rh-lateralorbitofrontal_FA | JLO (Years 0-2) | Pearson | -0.266725356 | 0.008268345 | 0.057878418 |
| ctx-lh-lateralorbitofrontal_FA | JLO (Years 2-4) | Pearson | 0.26584345 | 0.009604834 | 0.022411279 |
| Olfactory_Right_FA | LNS (Years 0-4) | Pearson | 0.263502757 | 0.009877847 | 0.027657972 |
| Left-Amygdala_FA | HVLDR (Years 0-4) | Pearson | 0.263152751 | 0.01081816 | 0.07572712 |
| Olfactory_Left_FA | HVLDR (Years 0-4) | Pearson | 0.262144625 | 0.010698885 | 0.041223285 |
| ctx-rh-lateralorbitofrontal_FA | HVLDR (Years 0-4) | Pearson | 0.258807049 | 0.011778081 | 0.041223285 |
| Olfactory_Left_FA | SF (Years 0-2) | Pearson | -0.245506207 | 0.015357827 | 0.215009572 |
| Right-Amygdala_FA | HVLDR (Years 0-4) | Pearson | 0.24346818 | 0.0180482 | 0.044266902 |
| Right-Amygdala_FA | HVLDR (Years 0-4) | Pearson | 0.241841439 | 0.019518701 | 0.091087271 |
| Right-Thalamus-Proper_FA | HVLDR (Years 0-4) | Pearson | 0.241616583 | 0.018971529 | 0.044266902 |
| Right-Thalamus-Proper_FA | HVLTR (Years 0-4) | Pearson | 0.241146709 | 0.018565281 | 0.259913937 |
| ctx-rh-lateralorbitofrontal_FA | LNS (Years 0-2) | Pearson | 0.238913554 | 0.018435157 | 0.056036209 |
| ctx-lh-lateralorbitofrontal_FA | LNS (Years 0-2) | Pearson | 0.237448992 | 0.019186697 | 0.056036209 |
| Right-Thalamus-Proper_FA | UPDRS 1A (Years 2-4) | Pearson | -0.237050606 | 0.055311014 | 0.585594122 |
| Olfactory_Right_FA | LNS (Years 0-2) | Pearson | 0.235894752 | 0.020012932 | 0.056036209 |
| Right-Thalamus-Proper_FA | JLO (Years 0-2) | Pearson | -0.227672205 | 0.024910763 | 0.116250229 |
| ctx-lh-medialorbitofrontal_FA | LNS (Years 0-4) | Pearson | 0.226831557 | 0.027069722 | 0.063162685 |
| Right-Amygdala_FA | UPDRS 1A (Years 0-4) | Pearson | 0.226718784 | 0.089899202 | 0.656225455 |
| Right-Amygdala_FA | UPDRS 1B (Years 0-4) | Pearson | -0.224103749 | 0.032720021 | 0.184354486 |
| Left-Amygdala_FA | HVLDR (Years 0-2) | Pearson | 0.223992042 | 0.028244603 | 0.332811615 |
| ctx-rh-insula_FA | HVLDR (Years 0-4) | Pearson | 0.22365506 | 0.030240021 | 0.055538764 |
| Olfactory_Left_FA | HVLTR (Years 0-2) | Pearson | 0.223242762 | 0.027948962 | 0.236754512 |
| ctx-rh-medialorbitofrontal_FA | HVLDR (Years 0-4) | Pearson | 0.221721395 | 0.031736436 | 0.055538764 |
| ctx-rh-lateralorbitofrontal_FA | UPDRS 2 (Years 0-4) | Pearson | -0.221147244 | 0.029492444 | 0.412894222 |
| ctx-lh-lateralorbitofrontal_FA | HVLDR (Years 0-4) | Pearson | 0.219583545 | 0.034444321 | 0.102651617 |
| ctx-lh-medialorbitofrontal_FA | HVLDR (Years 0-4) | Pearson | 0.217018384 | 0.036661292 | 0.102651617 |
| Left-Amygdala_FA | UPDRS 1 (Years 0-2) | Pearson | -0.21510255 | 0.039479644 | 0.552715011 |
| ctx-lh-entorhinal_FA | UPDRS 1B (Years 2-4) | Pearson | -0.213958354 | 0.038387605 | 0.537426474 |
| ctx-lh-lateralorbitofrontal_FA | HVLTR (Years 0-4) | Pearson | 0.213659785 | 0.037617223 | 0.263320561 |
| Olfactory_Left_FA | UPDRS 1B (Years 0-4) | Pearson | -0.212478773 | 0.043171153 | 0.184354486 |
| Right-Thalamus-Proper_FA | HVLTR (Years 0-2) | Pearson | 0.212194772 | 0.03692352 | 0.236754512 |
| ctx-rh-lateralorbitofrontal_FA | UPDRS 1B (Years 0-4) | Pearson | -0.211819866 | 0.043838333 | 0.184354486 |
| ctx-lh-lateralorbitofrontal_FA | UPDRS 1 (Years 0-4) | Pearson | -0.21066048 | 0.043837926 | 0.303186781 |
| Olfactory_Right_FA | HVLDR (Years 2-4) | Pearson | 0.210105463 | 0.042099032 | 0.416129559 |
| Right-Amygdala_FA | JLO (Years 0-4) | Pearson | 0.209330112 | 0.042880908 | 0.428185934 |
| ctx-lh-insula_FA | SDM (Years 0-4) | Pearson | 0.209097099 | 0.041993494 | 0.167704263 |
| Olfactory_Left_FA | UPDRS 1 (Years 0-4) | Pearson | -0.208495197 | 0.046102995 | 0.303186781 |
| ctx-rh-entorhinal_FA | HVLDR (Years 0-2) | Pearson | 0.205120413 | 0.044983824 | 0.172191608 |
| Right-Thalamus-Proper_FA | SDM (Years 0-4) | Pearson | 0.204565436 | 0.046751547 | 0.167704263 |
| Left-Amygdala_FA | UPDRS 1B (Years 0-4) | Pearson | -0.203795265 | 0.05267271 | 0.184354486 |
| ctx-rh-medialorbitofrontal_FA | SDM (Years 0-4) | Pearson | 0.203515391 | 0.047915504 | 0.167704263 |
| Olfactory_Left_FA | HVLDR (Years 0-2) | Pearson | 0.203421114 | 0.04682947 | 0.172191608 |
| Right-Thalamus-Proper_FA | HVLDR (Years 0-2) | Pearson | 0.202777652 | 0.047544516 | 0.332811615 |
| ctx-rh-lateralorbitofrontal_FA | UPDRS 2 (Years 2-4) | Pearson | -0.200663518 | 0.052471201 | 0.397778359 |
| ctx-rh-insula_FA | HVLTR (Years 0-2) | Pearson | 0.198962485 | 0.05073311 | 0.236754512 |
| Right-Thalamus-Proper_FA | MoCA (Years 2-4) | Pearson | 0.19876543 | 0.054791345 | 0.447609035 |
| ctx-lh-entorhinal_FA | SF (Years 0-2) | Pearson | -0.198101391 | 0.051762616 | 0.303611156 |
| ctx-lh-insula_FA | HVLDR (Years 0-4) | Pearson | 0.197951838 | 0.055811287 | 0.086817558 |
| Right-Thalamus-Proper_FA | SDM (Years 2-4) | Pearson | 0.196459856 | 0.05637254 | 0.141347044 |
| ctx-rh-lateralorbitofrontal_FA | HVLDR (Years 0-2) | Pearson | 0.196222979 | 0.055356006 | 0.172191608 |
| ctx-rh-insula_FA | JLO (Years 0-2) | Pearson | -0.19597919 | 0.054373479 | 0.190307175 |
| Right-Amygdala_FA | HVLDR (Years 2-4) | Pearson | 0.195147238 | 0.05944708 | 0.416129559 |
| Left-Amygdala_FA | SDM (Years 2-4) | Pearson | 0.194793564 | 0.058538748 | 0.141347044 |
| Olfactory_Right_FA | JLO (Years 0-4) | Pearson | 0.193867299 | 0.061169419 | 0.428185934 |
| ctx-lh-medialorbitofrontal_FA | SF (Years 0-4) | Pearson | -0.193600831 | 0.06013071 | 0.699015633 |
| ctx-rh-lateralorbitofrontal_FA | UPDRS 1 (Years 0-4) | Pearson | -0.193228357 | 0.064968596 | 0.303186781 |
| ctx-lh-lateralorbitofrontal_FA | MoCA (Years 2-4) | Pearson | 0.191865706 | 0.063944148 | 0.447609035 |
| ctx-rh-lateralorbitofrontal_FA | SDM (Years 2-4) | Pearson | 0.191837946 | 0.062548165 | 0.141347044 |
| ctx-lh-medialorbitofrontal_FA | UPDRS 1A (Years 0-4) | Pearson | -0.191203814 | 0.154227003 | 0.656225455 |
| ctx-lh-lateralorbitofrontal_FA | UPDRS 1B (Years 0-4) | Pearson | -0.190887076 | 0.069912189 | 0.195754129 |
| Left-Amygdala_FA | UPDRS 1B (Years 0-2) | Pearson | -0.190811014 | 0.070025856 | 0.506606253 |
| Olfactory_Left_FA | JLO (Years 2-4) | Pearson | 0.188528362 | 0.068797239 | 0.136825744 |
| ctx-rh-medialorbitofrontal_FA | HVLDR (Years 0-4) | Pearson | 0.188148554 | 0.0709081 | 0.156594781 |
| Olfactory_Left_FA | UPDRS 1A (Years 0-2) | Pearson | 0.187980045 | 0.161425096 | 0.939000138 |
| ctx-rh-lateralorbitofrontal_FA | UPDRS 1A (Years 2-4) | Pearson | -0.187481589 | 0.131706205 | 0.585594122 |
| Right-Amygdala_FA | HVLDR (Years 0-2) | Pearson | 0.187211107 | 0.06778362 | 0.172191608 |
| ctx-lh-insula_FA | HVLTR (Years 0-2) | Pearson | 0.185595938 | 0.068750005 | 0.240625018 |
| Left-Amygdala_FA | HVLDR (Years 0-4) | Pearson | 0.184411021 | 0.075190172 | 0.105266241 |
| ctx-rh-insula_FA | UPDRS 2 (Years 2-4) | Pearson | -0.183564831 | 0.076561362 | 0.397778359 |
| Right-Amygdala_FA | SDM (Years 2-4) | Pearson | 0.183413426 | 0.075214726 | 0.141347044 |
| Olfactory_Right_FA | JLO (Years 2-4) | Pearson | 0.182577737 | 0.07818614 | 0.136825744 |
| ctx-lh-medialorbitofrontal_FA | HVLDR (Years 0-2) | Pearson | 0.182216543 | 0.075589516 | 0.172191608 |
| ctx-rh-medialorbitofrontal_FA | SDM (Years 2-4) | Pearson | 0.180890044 | 0.079385922 | 0.141347044 |
| ctx-lh-insula_FA | SDM (Years 2-4) | Pearson | 0.180076355 | 0.080769739 | 0.141347044 |
| Left-Amygdala_FA | HVLDR (Years 0-2) | Pearson | 0.179792731 | 0.079629585 | 0.172191608 |
| ctx-rh-entorhinal_FA | HVLDR (Years 0-4) | Pearson | 0.179607133 | 0.08494619 | 0.156594781 |
| ctx-rh-entorhinal_FA | JLO (Years 0-2) | Pearson | -0.177457286 | 0.082048081 | 0.229734628 |
| ctx-lh-medialorbitofrontal_FA | HVLDR (Years 0-4) | Pearson | 0.17699039 | 0.087910122 | 0.11188561 |
| Olfactory_Right_FA | SF (Years 0-2) | Pearson | -0.176673746 | 0.083429855 | 0.303611156 |
| ctx-lh-medialorbitofrontal_FA | SF (Years 2-4) | Pearson | -0.175582579 | 0.088762698 | 0.692267795 |
| ctx-rh-medialorbitofrontal_FA | HVLDR (Years 0-2) | Pearson | 0.175122985 | 0.087899037 | 0.172191608 |
| ctx-rh-lateralorbitofrontal_FA | HVLDR (Years 0-4) | Pearson | 0.175034487 | 0.093322948 | 0.156594781 |
| ctx-rh-lateralorbitofrontal_FA | SDM (Years 0-4) | Pearson | 0.17473555 | 0.090337251 | 0.246164529 |
| ctx-lh-entorhinal_FA | UPDRS 1 (Years 2-4) | Pearson | -0.174017751 | 0.09346139 | 0.72897968 |
| Left-Amygdala_FA | SF (Years 0-2) | Pearson | -0.173521136 | 0.089177901 | 0.303611156 |
| ctx-rh-medialorbitofrontal_FA | HVLTR (Years 0-2) | Pearson | 0.173402072 | 0.089400989 | 0.250322768 |
| Olfactory_Left_FA | UPDRS 2 (Years 2-4) | Pearson | -0.172677462 | 0.096053123 | 0.397778359 |
| Olfactory_Left_FA | HVLDR (Years 0-4) | Pearson | 0.171284453 | 0.100668074 | 0.156594781 |
| ctx-lh-lateralorbitofrontal_FA | UPDRS 1A (Years 2-4) | Pearson | -0.169083114 | 0.174721882 | 0.585594122 |
| ctx-lh-insula_FA | MoCA (Years 0-4) | Pearson | 0.167136732 | 0.105470732 | 0.793336395 |
| ctx-rh-insula_FA | SDM (Years 0-4) | Pearson | 0.167123323 | 0.105499084 | 0.246164529 |
| Right-Thalamus-Proper_FA | UPDRS 1A (Years 0-4) | Pearson | -0.166853563 | 0.214780851 | 0.656225455 |
| Olfactory_Left_FA | UPDRS 1B (Years 0-2) | Pearson | -0.166694093 | 0.114278929 | 0.506606253 |
| ctx-rh-entorhinal_FA | GDS (Years 0-2) | Pearson | -0.166188912 | 0.103761068 | 0.925650844 |
| ctx-rh-insula_FA | HVLDR (Years 0-2) | Pearson | 0.165936864 | 0.106144529 | 0.172191608 |
| ctx-rh-lateralorbitofrontal_FA | SF (Years 0-2) | Pearson | -0.164011732 | 0.108432556 | 0.303611156 |
| Left-Thalamus-Proper_FA | HVLDR (Years 0-2) | Pearson | 0.163841131 | 0.110694605 | 0.172191608 |
| ctx-rh-entorhinal_FA | GDS (Years 0-4) | Pearson | -0.163832636 | 0.10882401 | 0.881549631 |
| Left-Thalamus-Proper_FA | JLO (Years 0-4) | Pearson | -0.163768342 | 0.114745597 | 0.535479453 |
| ctx-lh-medialorbitofrontal_FA | UPDRS 1A (Years 2-4) | Pearson | -0.163657421 | 0.18917334 | 0.585594122 |
| Left-Amygdala_FA | UPDRS 1A (Years 0-4) | Pearson | 0.158483715 | 0.239005335 | 0.656225455 |
| ctx-lh-medialorbitofrontal_FA | UPDRS 1B (Years 0-4) | Pearson | -0.158119653 | 0.13440672 | 0.313615681 |
| ctx-rh-medialorbitofrontal_FA | UPDRS 2 (Years 2-4) | Pearson | -0.157967517 | 0.128354084 | 0.397778359 |
| Right-Amygdala_FA | LNS (Years 2-4) | Pearson | -0.157555374 | 0.127291178 | 0.904783342 |
| ctx-lh-insula_FA | JLO (Years 0-2) | Pearson | -0.157209551 | 0.124087271 | 0.289536965 |
| Right-Amygdala_FA | HVLDR (Years 0-2) | Pearson | 0.157116887 | 0.126321259 | 0.44980238 |
| ctx-lh-entorhinal_FA | UPDRS 1B (Years 0-2) | Pearson | 0.156159637 | 0.139365022 | 0.506606253 |
| ctx-rh-insula_FA | SDM (Years 2-4) | Pearson | 0.155409879 | 0.132620345 | 0.206298314 |
| Olfactory_Right_FA | HVLTR (Years 0-4) | Pearson | 0.154683359 | 0.134462944 | 0.413379571 |
| Left-Thalamus-Proper_FA | HVLDR (Years 0-4) | Pearson | 0.154363207 | 0.137411006 | 0.16031284 |
| ctx-rh-lateralorbitofrontal_FA | UPDRS 1B (Years 0-2) | Pearson | -0.154091874 | 0.144744644 | 0.506606253 |
| ctx-lh-medialorbitofrontal_FA | UPDRS 1 (Years 2-4) | Pearson | -0.153195452 | 0.140446961 | 0.72897968 |
| ctx-lh-medialorbitofrontal_FA | UPDRS 1 (Years 0-4) | Pearson | -0.152695562 | 0.14619168 | 0.358133738 |
| ctx-rh-medialorbitofrontal_FA | HVLTR (Years 0-4) | Pearson | 0.152569192 | 0.139935683 | 0.413379571 |
| Olfactory_Left_FA | SDM (Years 0-4) | Pearson | 0.152484821 | 0.140157532 | 0.280315065 |
| ctx-rh-lateralorbitofrontal_FA | HVLTR (Years 0-2) | Pearson | 0.150590577 | 0.140932203 | 0.291022781 |
| ctx-rh-lateralorbitofrontal_FA | UPDRS 1 (Years 0-2) | Pearson | -0.150522275 | 0.152088649 | 0.553261394 |
| Left-Thalamus-Proper_FA | UPDRS 2 (Years 2-4) | Pearson | -0.149160721 | 0.151327652 | 0.397778359 |
| ctx-lh-lateralorbitofrontal_FA | HVLTR (Years 0-2) | Pearson | 0.148895833 | 0.145511391 | 0.291022781 |
| Right-Thalamus-Proper_FA | MoCA (Years 0-4) | Pearson | 0.148436596 | 0.151117834 | 0.793336395 |
| ctx-lh-insula_FA | HVLTR (Years 2-4) | Pearson | -0.147249864 | 0.154449516 | 0.7492054 |
| Left-Thalamus-Proper_FA | UPDRS 1A (Years 0-4) | Pearson | -0.145684112 | 0.279561552 | 0.656225455 |
| Left-Amygdala_FA | UPDRS 1 (Years 0-4) | Pearson | -0.145380449 | 0.16674678 | 0.358133738 |
| ctx-lh-lateralorbitofrontal_FA | HVLDR (Years 0-2) | Pearson | 0.145219151 | 0.158035925 | 0.44980238 |
| Olfactory_Left_FA | UPDRS 1A (Years 0-4) | Pearson | 0.145182165 | 0.281239481 | 0.656225455 |
| Olfactory_Left_FA | LNS (Years 0-2) | Pearson | 0.144918936 | 0.156696016 | 0.365624038 |
| ctx-lh-lateralorbitofrontal_FA | UPDRS 2 (Years 0-4) | Pearson | -0.144016639 | 0.159320638 | 0.547517836 |
| Left-Thalamus-Proper_FA | MoCA (Years 2-4) | Pearson | 0.143908088 | 0.166423712 | 0.752414275 |
| ctx-lh-insula_FA | UPDRS 1B (Years 0-4) | Pearson | -0.142736249 | 0.17710696 | 0.344010487 |
| ctx-rh-entorhinal_FA | SF (Years 0-4) | Pearson | -0.142691017 | 0.16775963 | 0.699015633 |
| ctx-rh-insula_FA | UPDRS 1 (Years 0-4) | Pearson | -0.1426163 | 0.175045796 | 0.358133738 |
| ctx-lh-insula_FA | UPDRS 2 (Years 2-4) | Pearson | -0.142558486 | 0.170476439 | 0.397778359 |
| Left-Amygdala_FA | JLO (Years 0-4) | Pearson | 0.142162797 | 0.171678331 | 0.594056703 |
| Left-Amygdala_FA | UPDRS 2 (Years 0-4) | Pearson | -0.142104208 | 0.164991538 | 0.547517836 |
| ctx-rh-lateralorbitofrontal_FA | UPDRS 1B (Years 2-4) | Pearson | -0.14155706 | 0.173530299 | 0.743141341 |
| ctx-lh-insula_FA | UPDRS 1 (Years 0-4) | Pearson | -0.141311524 | 0.179066869 | 0.358133738 |
| ctx-lh-entorhinal_FA | UPDRS 1A (Years 2-4) | Pearson | -0.141039134 | 0.258656575 | 0.585594122 |
| Olfactory_Left_FA | UPDRS 1 (Years 2-4) | Pearson | -0.138687059 | 0.182504864 | 0.72897968 |
| Left-Amygdala_FA | UPDRS 2 (Years 0-2) | Pearson | -0.137875646 | 0.178058919 | 0.880863766 |
| ctx-lh-medialorbitofrontal_FA | SDM (Years 0-2) | Pearson | 0.137444435 | 0.179432904 | 0.810280936 |
| ctx-rh-insula_FA | UPDRS 2 (Years 0-4) | Pearson | -0.13706437 | 0.180650333 | 0.547517836 |
| ctx-rh-insula_FA | UPDRS 1B (Years 0-4) | Pearson | -0.136624663 | 0.196577421 | 0.344010487 |
| ctx-rh-entorhinal_FA | SDM (Years 0-4) | Pearson | 0.134032083 | 0.19534075 | 0.341846312 |
| ctx-rh-entorhinal_FA | JLO (Years 2-4) | Pearson | 0.133785518 | 0.198606702 | 0.298282127 |
| Right-Amygdala_FA | SF (Years 2-4) | Pearson | 0.133251407 | 0.197979346 | 0.692267795 |
| Right-Amygdala_FA | HVLTR (Years 2-4) | Pearson | 0.132812375 | 0.199474444 | 0.7492054 |
| ctx-lh-insula_FA | UPDRS 2 (Years 0-4) | Pearson | -0.132559698 | 0.195542084 | 0.547517836 |
| ctx-lh-lateralorbitofrontal_FA | JLO (Years 0-2) | Pearson | -0.132200827 | 0.196765451 | 0.384116068 |
| ctx-lh-lateralorbitofrontal_FA | UPDRS 1B (Years 2-4) | Pearson | -0.131861386 | 0.205198916 | 0.743141341 |
| ctx-lh-lateralorbitofrontal_FA | UPDRS 1 (Years 2-4) | Pearson | -0.130977254 | 0.208279909 | 0.72897968 |
| Olfactory_Right_FA | SF (Years 2-4) | Pearson | 0.130735519 | 0.206657077 | 0.692267795 |
| Right-Amygdala_FA | HVLTR (Years 0-4) | Pearson | 0.130112563 | 0.208847092 | 0.413379571 |
| Left-Amygdala_FA | UPDRS 1A (Years 0-2) | Pearson | 0.129689628 | 0.336295005 | 0.939000138 |
| ctx-lh-insula_FA | UPDRS 1 (Years 0-2) | Pearson | -0.129638371 | 0.218079346 | 0.553261394 |
| ctx-rh-medialorbitofrontal_FA | JLO (Years 2-4) | Pearson | 0.129624187 | 0.213058662 | 0.298282127 |
| ctx-lh-lateralorbitofrontal_FA | JLO (Years 0-4) | Pearson | 0.129569267 | 0.21325426 | 0.594056703 |
| ctx-lh-medialorbitofrontal_FA | UPDRS 1 (Years 0-2) | Pearson | -0.129419304 | 0.218864776 | 0.553261394 |
| ctx-lh-insula_FA | MoCA (Years 0-2) | Pearson | 0.128533497 | 0.212011628 | 0.994659941 |
| ctx-rh-insula_FA | MoCA (Years 0-4) | Pearson | 0.127784634 | 0.21717729 | 0.793336395 |
| ctx-rh-lateralorbitofrontal_FA | MoCA (Years 2-4) | Pearson | 0.127634148 | 0.220227713 | 0.752414275 |
| Olfactory_Left_FA | UPDRS 1A (Years 2-4) | Pearson | -0.126949141 | 0.309747906 | 0.585594122 |
| ctx-lh-entorhinal_FA | GDS (Years 0-2) | Pearson | -0.126747888 | 0.216036799 | 0.925650844 |
| Olfactory_Left_FA | UPDRS 1 (Years 0-2) | Pearson | -0.12659796 | 0.229159134 | 0.553261394 |
| ctx-lh-entorhinal_FA | GDS (Years 0-4) | Pearson | -0.126212869 | 0.217997251 | 0.881549631 |
| Olfactory_Left_FA | JLO (Years 0-2) | Pearson | -0.125806442 | 0.219494896 | 0.384116068 |
| ctx-lh-medialorbitofrontal_FA | LNS (Years 2-4) | Pearson | -0.125007135 | 0.227420458 | 0.904783342 |
| Right-Amygdala_FA | UPDRS 1 (Years 0-4) | Pearson | -0.124818063 | 0.235825222 | 0.383838552 |
| ctx-rh-lateralorbitofrontal_FA | HVLTR (Years 0-4) | Pearson | 0.123870007 | 0.2317103 | 0.413379571 |
| Right-Thalamus-Proper_FA | HVLDR (Years 0-4) | Pearson | 0.12372203 | 0.237387838 | 0.332342973 |
| Olfactory_Left_FA | SF (Years 2-4) | Pearson | 0.123676543 | 0.23244574 | 0.692267795 |
| Left-Amygdala_FA | SDM (Years 0-4) | Pearson | 0.123655482 | 0.232525898 | 0.361706953 |
| Olfactory_Left_FA | GDS (Years 0-4) | Pearson | -0.123240891 | 0.229116545 | 0.881549631 |
| ctx-rh-entorhinal_FA | HVLTR (Years 0-4) | Pearson | 0.123191415 | 0.234297073 | 0.413379571 |
| Olfactory_Left_FA | HVLTR (Years 0-4) | Pearson | 0.122691154 | 0.236216898 | 0.413379571 |
| Olfactory_Left_FA | HVLDR (Years 2-4) | Pearson | 0.122473083 | 0.239608458 | 0.749082424 |
| Olfactory_Right_FA | UPDRS 1 (Years 0-4) | Pearson | -0.121974326 | 0.246753355 | 0.383838552 |
| ctx-lh-insula_FA | UPDRS 1B (Years 0-2) | Pearson | -0.12178481 | 0.250152305 | 0.620397815 |
| ctx-lh-entorhinal_FA | UPDRS 2 (Years 2-4) | Pearson | -0.121252775 | 0.244358736 | 0.403391097 |
| ctx-rh-entorhinal_FA | HVLTR (Years 2-4) | Pearson | 0.11988141 | 0.247202991 | 0.7492054 |
| ctx-lh-entorhinal_FA | SF (Years 0-4) | Pearson | -0.119474401 | 0.248823111 | 0.699015633 |
| ctx-rh-entorhinal_FA | SF (Years 0-2) | Pearson | -0.119048979 | 0.24546611 | 0.572754258 |
| ctx-lh-lateralorbitofrontal_FA | MoCA (Years 0-4) | Pearson | 0.118645148 | 0.252146561 | 0.793336395 |
| Left-Thalamus-Proper_FA | JLO (Years 2-4) | Pearson | 0.118294919 | 0.256141343 | 0.323180569 |
| ctx-rh-insula_FA | UPDRS 1B (Years 0-2) | Pearson | -0.11785326 | 0.265884778 | 0.620397815 |
| ctx-rh-insula_FA | HVLTR (Years 2-4) | Pearson | -0.117820271 | 0.255482552 | 0.7492054 |
| Left-Amygdala_FA | HVLTR (Years 0-2) | Pearson | 0.117666645 | 0.251029942 | 0.439302398 |
| ctx-rh-entorhinal_FA | SDM (Years 0-2) | Pearson | 0.117540358 | 0.251542523 | 0.810280936 |
| ctx-lh-lateralorbitofrontal_FA | HVLDR (Years 0-2) | Pearson | 0.117133697 | 0.255724481 | 0.358014274 |
| Olfactory_Right_FA | HVLDR (Years 2-4) | Pearson | -0.116576951 | 0.263160246 | 0.97777147 |
| ctx-lh-insula_FA | JLO (Years 0-4) | Pearson | 0.11630963 | 0.264264036 | 0.594056703 |
| Olfactory_Left_FA | GDS (Years 0-2) | Pearson | -0.116290916 | 0.256652574 | 0.925650844 |
| Left-Amygdala_FA | UPDRS 2 (Years 2-4) | Pearson | -0.11583743 | 0.266221451 | 0.403391097 |
| ctx-lh-lateralorbitofrontal_FA | SDM (Years 0-4) | Pearson | 0.114900747 | 0.26753153 | 0.374264967 |
| ctx-lh-entorhinal_FA | LNS (Years 2-4) | Pearson | -0.114750194 | 0.268163114 | 0.904783342 |
| Right-Thalamus-Proper_FA | UPDRS 2 (Years 0-4) | Pearson | -0.114377832 | 0.264613463 | 0.617431413 |
| ctx-rh-insula_FA | MoCA (Years 0-2) | Pearson | 0.114269081 | 0.267614339 | 0.994659941 |
| ctx-rh-lateralorbitofrontal_FA | UPDRS 1 (Years 2-4) | Pearson | -0.11422811 | 0.27296617 | 0.747148249 |
| Right-Amygdala_FA | LNS (Years 0-2) | Pearson | 0.113490298 | 0.268363033 | 0.469646971 |
| ctx-rh-entorhinal_FA | LNS (Years 0-2) | Pearson | 0.113488727 | 0.268369698 | 0.469646971 |
| ctx-rh-insula_FA | UPDRS 1 (Years 0-2) | Pearson | -0.112976363 | 0.283606692 | 0.553261394 |
| Left-Thalamus-Proper_FA | UPDRS 1A (Years 2-4) | Pearson | -0.112857696 | 0.366935121 | 0.585594122 |
| ctx-lh-entorhinal_FA | UPDRS 2 (Years 0-2) | Pearson | 0.112696093 | 0.271748621 | 0.880863766 |
| ctx-rh-lateralorbitofrontal_FA | SF (Years 2-4) | Pearson | 0.11212885 | 0.279321528 | 0.692267795 |
| ctx-lh-entorhinal_FA | HVLDR (Years 0-4) | Pearson | 0.112067914 | 0.284844254 | 0.362529051 |
| Olfactory_Right_FA | UPDRS 1A (Years 2-4) | Pearson | -0.111877538 | 0.371138082 | 0.585594122 |
| ctx-rh-insula_FA | HVLDR (Years 0-2) | Pearson | 0.111648528 | 0.278813775 | 0.44980238 |
| ctx-rh-lateralorbitofrontal_FA | HVLDR (Years 0-2) | Pearson | 0.111161215 | 0.280930496 | 0.44980238 |
| Right-Thalamus-Proper_FA | UPDRS 2 (Years 2-4) | Pearson | -0.111081354 | 0.286483987 | 0.403391097 |
| ctx-rh-insula_FA | UPDRS 1A (Years 2-4) | Pearson | -0.110647558 | 0.376453364 | 0.585594122 |
| Right-Thalamus-Proper_FA | LNS (Years 0-4) | Pearson | 0.110441607 | 0.286665711 | 0.573331422 |
| ctx-lh-entorhinal_FA | HVLDR (Years 2-4) | Pearson | -0.110051007 | 0.291005162 | 0.749082424 |
| ctx-lh-entorhinal_FA | JLO (Years 2-4) | Pearson | -0.109966807 | 0.291376708 | 0.323180569 |
| Right-Amygdala_FA | SDM (Years 0-4) | Pearson | 0.108770426 | 0.294065331 | 0.374264967 |
| Right-Amygdala_FA | UPDRS 2 (Years 2-4) | Pearson | -0.108627185 | 0.297330181 | 0.403391097 |
| Olfactory_Right_FA | HVLTR (Years 0-2) | Pearson | 0.108576979 | 0.289768388 | 0.450750826 |
| Left-Thalamus-Proper_FA | HVLDR (Years 0-2) | Pearson | 0.108274152 | 0.293690717 | 0.44980238 |
| Right-Thalamus-Proper_FA | JLO (Years 0-4) | Pearson | -0.108188137 | 0.29929865 | 0.594056703 |
| Left-Thalamus-Proper_FA | SF (Years 2-4) | Pearson | -0.108185179 | 0.296686198 | 0.692267795 |
| Olfactory_Left_FA | UPDRS 1B (Years 2-4) | Pearson | -0.107778331 | 0.301143715 | 0.743141341 |
| ctx-lh-lateralorbitofrontal_FA | HVLDR (Years 2-4) | Pearson | 0.107670937 | 0.301628461 | 0.749082424 |
| Olfactory_Left_FA | HVLTR (Years 2-4) | Pearson | -0.106676898 | 0.30351127 | 0.7492054 |
| ctx-lh-insula_FA | HVLDR (Years 0-2) | Pearson | 0.106536502 | 0.301552355 | 0.383793906 |
| ctx-lh-medialorbitofrontal_FA | JLO (Years 2-4) | Pearson | 0.104608219 | 0.315667736 | 0.323180569 |
| Olfactory_Right_FA | GDS (Years 0-4) | Pearson | -0.104587469 | 0.307959521 | 0.881549631 |
| ctx-rh-lateralorbitofrontal_FA | GDS (Years 2-4) | Pearson | -0.10450584 | 0.308339326 | 0.981253447 |
| ctx-lh-lateralorbitofrontal_FA | UPDRS 2 (Years 2-4) | Pearson | -0.104332896 | 0.316950148 | 0.403391097 |
| ctx-rh-medialorbitofrontal_FA | SF (Years 0-2) | Pearson | -0.104309574 | 0.309253762 | 0.618507524 |
| ctx-rh-medialorbitofrontal_FA | SDM (Years 0-2) | Pearson | 0.104108276 | 0.310193473 | 0.810280936 |
| ctx-rh-entorhinal_FA | UPDRS 1 (Years 2-4) | Pearson | -0.103637019 | 0.320206392 | 0.747148249 |
| Olfactory_Right_FA | UPDRS 1 (Years 0-2) | Pearson | -0.10337337 | 0.326792038 | 0.553261394 |
| ctx-rh-entorhinal_FA | HVLDR (Years 0-2) | Pearson | 0.10308758 | 0.317560953 | 0.44980238 |
| Right-Thalamus-Proper_FA | JLO (Years 2-4) | Pearson | 0.103005387 | 0.323180569 | 0.323180569 |
| ctx-lh-insula_FA | HVLDR (Years 0-2) | Pearson | 0.102958753 | 0.318169348 | 0.44980238 |
| ctx-lh-medialorbitofrontal_FA | SDM (Years 0-4) | Pearson | 0.102552991 | 0.322692076 | 0.376474089 |
| ctx-rh-medialorbitofrontal_FA | HVLDR (Years 0-2) | Pearson | 0.102300984 | 0.321287414 | 0.44980238 |
| ctx-lh-entorhinal_FA | UPDRS 1 (Years 0-2) | Pearson | 0.101877303 | 0.333879387 | 0.553261394 |
| Right-Thalamus-Proper_FA | SF (Years 0-4) | Pearson | -0.101795523 | 0.326297894 | 0.699015633 |
| Left-Amygdala_FA | LNS (Years 0-2) | Pearson | 0.101326546 | 0.323369062 | 0.503018541 |
| Olfactory_Right_FA | UPDRS 1B (Years 0-4) | Pearson | -0.101194134 | 0.339861576 | 0.528673563 |
| ctx-rh-insula_FA | HVLDR (Years 2-4) | Pearson | -0.100086367 | 0.33715461 | 0.97777147 |
| ctx-lh-lateralorbitofrontal_FA | SDM (Years 0-2) | Pearson | 0.099892115 | 0.330301426 | 0.810280936 |
| ctx-lh-entorhinal_FA | HVLDR (Years 2-4) | Pearson | 0.099802771 | 0.338532314 | 0.97777147 |
| ctx-lh-lateralorbitofrontal_FA | HVLDR (Years 2-4) | Pearson | 0.09971393 | 0.338964633 | 0.97777147 |
| ctx-rh-entorhinal_FA | JLO (Years 0-4) | Pearson | -0.099612021 | 0.339460973 | 0.594056703 |
| Left-Amygdala_FA | GDS (Years 2-4) | Pearson | -0.09956047 | 0.331917587 | 0.981253447 |
| ctx-rh-insula_FA | HVLDR (Years 0-4) | Pearson | 0.099252534 | 0.343867443 | 0.401178684 |
| ctx-lh-lateralorbitofrontal_FA | SDM (Years 2-4) | Pearson | 0.098831093 | 0.340656178 | 0.476918649 |
| Right-Amygdala_FA | UPDRS 1 (Years 0-2) | Pearson | -0.097399773 | 0.355668039 | 0.553261394 |
| Olfactory_Right_FA | UPDRS 2 (Years 2-4) | Pearson | -0.096867617 | 0.352999487 | 0.411832735 |
| ctx-lh-lateralorbitofrontal_FA | UPDRS 2 (Years 0-2) | Pearson | -0.096439746 | 0.347371011 | 0.880863766 |
| Left-Thalamus-Proper_FA | SF (Years 0-4) | Pearson | -0.09640549 | 0.35269617 | 0.699015633 |
| Left-Thalamus-Proper_FA | UPDRS 1A (Years 0-2) | Pearson | -0.096257022 | 0.476290908 | 0.939000138 |
| Olfactory_Left_FA | UPDRS 2 (Years 0-4) | Pearson | -0.096209763 | 0.348527405 | 0.620178686 |
| ctx-lh-entorhinal_FA | HVLTR (Years 0-4) | Pearson | -0.096096391 | 0.354249248 | 0.528550048 |
| Left-Amygdala_FA | LNS (Years 0-4) | Pearson | 0.095921692 | 0.355128905 | 0.581213353 |
| Left-Amygdala_FA | GDS (Years 0-4) | Pearson | -0.095079287 | 0.354246551 | 0.881549631 |
| Olfactory_Right_FA | HVLDR (Years 0-2) | Pearson | 0.094037397 | 0.362123315 | 0.422477201 |
| Olfactory_Left_FA | SF (Years 0-4) | Pearson | -0.093822294 | 0.365805795 | 0.699015633 |
| Left-Thalamus-Proper_FA | UPDRS 2 (Years 0-4) | Pearson | -0.093662256 | 0.361497272 | 0.620178686 |
| Olfactory_Left_FA | HVLDR (Years 0-2) | Pearson | 0.093584848 | 0.364448318 | 0.463843314 |
| ctx-rh-entorhinal_FA | HVLDR (Years 0-4) | Pearson | 0.093472013 | 0.370208331 | 0.398685895 |
| ctx-rh-insula_FA | LNS (Years 0-2) | Pearson | 0.093310664 | 0.363310395 | 0.508634554 |
| Left-Amygdala_FA | HVLTR (Years 2-4) | Pearson | -0.093126265 | 0.369388637 | 0.7492054 |
| ctx-rh-insula_FA | LNS (Years 0-4) | Pearson | 0.092306245 | 0.373637156 | 0.581213353 |
| Right-Amygdala_FA | SF (Years 0-4) | Pearson | 0.091880244 | 0.375855958 | 0.699015633 |
| ctx-lh-entorhinal_FA | UPDRS 1A (Years 0-2) | Pearson | -0.09073433 | 0.502068136 | 0.939000138 |
| Right-Thalamus-Proper_FA | SF (Years 2-4) | Pearson | -0.090703809 | 0.382024801 | 0.758345587 |
| ctx-lh-medialorbitofrontal_FA | MoCA (Years 2-4) | Pearson | 0.090349101 | 0.386479386 | 0.752414275 |
| ctx-rh-lateralorbitofrontal_FA | GDS (Years 0-2) | Pearson | 0.090065443 | 0.380308904 | 0.925650844 |
| ctx-lh-entorhinal_FA | HVLTR (Years 2-4) | Pearson | -0.088566331 | 0.393388205 | 0.7492054 |
| ctx-lh-insula_FA | MoCA (Years 2-4) | Pearson | 0.088172905 | 0.398067547 | 0.752414275 |
| Olfactory_Right_FA | UPDRS 2 (Years 0-2) | Pearson | -0.088126737 | 0.390689487 | 0.880863766 |
| ctx-lh-insula_FA | GDS (Years 2-4) | Pearson | -0.087916187 | 0.391826962 | 0.981253447 |
| ctx-rh-medialorbitofrontal_FA | HVLDR (Years 2-4) | Pearson | 0.087905765 | 0.39950411 | 0.749082424 |
| ctx-lh-medialorbitofrontal_FA | GDS (Years 2-4) | Pearson | -0.087856027 | 0.392152331 | 0.981253447 |
| Right-Thalamus-Proper_FA | SDM (Years 0-2) | Pearson | 0.087684885 | 0.393078821 | 0.810280936 |
| Left-Thalamus-Proper_FA | UPDRS 1 (Years 2-4) | Pearson | -0.087604318 | 0.401128839 | 0.802257679 |
| ctx-rh-lateralorbitofrontal_FA | HVLDR (Years 2-4) | Pearson | 0.0875291 | 0.401534855 | 0.749082424 |
| ctx-lh-medialorbitofrontal_FA | UPDRS 1B (Years 0-2) | Pearson | -0.087332272 | 0.410420127 | 0.647247659 |
| Olfactory_Right_FA | UPDRS 2 (Years 0-4) | Pearson | -0.086654155 | 0.398686298 | 0.620178686 |
| ctx-rh-insula_FA | JLO (Years 0-4) | Pearson | 0.0863871 | 0.407728942 | 0.632137121 |
| ctx-rh-insula_FA | HVLTR (Years 0-4) | Pearson | 0.086363504 | 0.405307252 | 0.528550048 |
| ctx-rh-entorhinal_FA | UPDRS 1B (Years 0-4) | Pearson | -0.085633371 | 0.419619396 | 0.587467155 |
| ctx-rh-insula_FA | UPDRS 1A (Years 0-2) | Pearson | 0.085408648 | 0.527588636 | 0.939000138 |
| ctx-rh-entorhinal_FA | MoCA (Years 0-4) | Pearson | -0.085388571 | 0.410649468 | 0.793336395 |
| Left-Thalamus-Proper_FA | HVLTR (Years 0-2) | Pearson | 0.084888043 | 0.408403929 | 0.571765501 |
| Left-Thalamus-Proper_FA | HVLTR (Years 0-4) | Pearson | 0.084547814 | 0.415289323 | 0.528550048 |
| Olfactory_Right_FA | UPDRS 1B (Years 0-2) | Pearson | -0.08407197 | 0.428178251 | 0.647247659 |
| ctx-lh-entorhinal_FA | MoCA (Years 2-4) | Pearson | 0.084038936 | 0.420639725 | 0.752414275 |
| Left-Thalamus-Proper_FA | GDS (Years 0-2) | Pearson | -0.083147742 | 0.418113983 | 0.925650844 |
| Olfactory_Left_FA | MoCA (Years 2-4) | Pearson | 0.082371198 | 0.429951014 | 0.752414275 |
| ctx-rh-lateralorbitofrontal_FA | HVLTR (Years 2-4) | Pearson | -0.082251279 | 0.428117371 | 0.7492054 |
| Right-Amygdala_FA | UPDRS 1B (Years 0-2) | Pearson | -0.081471441 | 0.442652511 | 0.647247659 |
| Left-Amygdala_FA | SF (Years 2-4) | Pearson | 0.08132757 | 0.433340335 | 0.758345587 |
| Olfactory_Left_FA | SDM (Years 0-2) | Pearson | -0.080323369 | 0.434154128 | 0.810280936 |
| ctx-lh-entorhinal_FA | HVLDR (Years 0-2) | Pearson | 0.0801007 | 0.437872821 | 0.482903022 |
| ctx-lh-insula_FA | HVLDR (Years 0-4) | Pearson | 0.080074159 | 0.445469554 | 0.479736442 |
| ctx-rh-entorhinal_FA | UPDRS 1B (Years 2-4) | Pearson | -0.079646086 | 0.445416369 | 0.743141341 |
| ctx-lh-medialorbitofrontal_FA | GDS (Years 0-4) | Pearson | 0.079518333 | 0.438789344 | 0.881549631 |
| ctx-lh-insula_FA | SDM (Years 0-2) | Pearson | 0.079476307 | 0.439032087 | 0.810280936 |
| ctx-rh-insula_FA | HVLDR (Years 2-4) | Pearson | 0.079135131 | 0.448350421 | 0.749082424 |
| Left-Thalamus-Proper_FA | UPDRS 1 (Years 0-4) | Pearson | -0.078875897 | 0.454838769 | 0.636774277 |
| ctx-rh-lateralorbitofrontal_FA | JLO (Years 0-4) | Pearson | 0.078584126 | 0.451526515 | 0.632137121 |
| ctx-lh-medialorbitofrontal_FA | HVLDR (Years 0-2) | Pearson | 0.078276611 | 0.448409949 | 0.482903022 |
| ctx-rh-entorhinal_FA | UPDRS 1B (Years 0-2) | Pearson | -0.078013237 | 0.462319757 | 0.647247659 |
| Left-Amygdala_FA | SF (Years 0-4) | Pearson | -0.077786153 | 0.453697407 | 0.699015633 |
| ctx-rh-medialorbitofrontal_FA | JLO (Years 0-2) | Pearson | -0.077275938 | 0.451847013 | 0.628478692 |
| Olfactory_Left_FA | MoCA (Years 0-4) | Pearson | 0.076429253 | 0.461635614 | 0.793336395 |
| Right-Thalamus-Proper_FA | LNS (Years 0-2) | Pearson | 0.076106295 | 0.458742815 | 0.583854492 |
| ctx-rh-insula_FA | UPDRS 1B (Years 2-4) | Pearson | -0.075923336 | 0.46703869 | 0.743141341 |
| Olfactory_Left_FA | LNS (Years 2-4) | Pearson | 0.075888451 | 0.464820585 | 0.904783342 |
| ctx-rh-lateralorbitofrontal_FA | UPDRS 1A (Years 0-4) | Pearson | -0.075734844 | 0.575531947 | 0.938670598 |
| Right-Amygdala_FA | UPDRS 1B (Years 2-4) | Pearson | -0.075331061 | 0.470530727 | 0.743141341 |
| Right-Amygdala_FA | GDS (Years 2-4) | Pearson | 0.075257726 | 0.463781795 | 0.981253447 |
| ctx-lh-entorhinal_FA | HVLDR (Years 0-2) | Pearson | 0.074870221 | 0.468461656 | 0.504497168 |
| ctx-lh-medialorbitofrontal_FA | GDS (Years 0-2) | Pearson | 0.074690748 | 0.467165459 | 0.925650844 |
| Left-Thalamus-Proper_FA | HVLDR (Years 2-4) | Pearson | 0.07406173 | 0.478062195 | 0.97777147 |
| ctx-lh-insula_FA | UPDRS 1B (Years 2-4) | Pearson | -0.073684083 | 0.480315377 | 0.743141341 |
| ctx-lh-medialorbitofrontal_FA | HVLDR (Years 2-4) | Pearson | 0.073477056 | 0.481552987 | 0.749082424 |
| ctx-rh-medialorbitofrontal_FA | SF (Years 0-4) | Pearson | -0.07345132 | 0.479321782 | 0.699015633 |
| ctx-rh-entorhinal_FA | MoCA (Years 0-2) | Pearson | -0.072925179 | 0.480126551 | 0.994659941 |
| Olfactory_Right_FA | GDS (Years 0-2) | Pearson | -0.072473736 | 0.480524793 | 0.925650844 |
| ctx-rh-entorhinal_FA | SDM (Years 2-4) | Pearson | 0.072215281 | 0.486768127 | 0.61952307 |
| ctx-lh-lateralorbitofrontal_FA | GDS (Years 0-4) | Pearson | 0.072030386 | 0.483220704 | 0.881549631 |
| Olfactory_Right_FA | HVLDR (Years 0-2) | Pearson | 0.071862528 | 0.486564739 | 0.486564739 |
| Left-Thalamus-Proper_FA | UPDRS 1B (Years 0-4) | Pearson | -0.071417856 | 0.501122431 | 0.637792185 |
| ctx-lh-medialorbitofrontal_FA | JLO (Years 0-2) | Pearson | -0.070873678 | 0.490292271 | 0.628478692 |
| Right-Thalamus-Proper_FA | HVLTR (Years 2-4) | Pearson | 0.070373142 | 0.497978955 | 0.77463393 |
| Left-Amygdala_FA | JLO (Years 0-2) | Pearson | -0.070302433 | 0.493804687 | 0.628478692 |
| ctx-lh-lateralorbitofrontal_FA | SF (Years 0-4) | Pearson | -0.070158022 | 0.499296881 | 0.699015633 |
| Right-Amygdala_FA | UPDRS 1A (Years 2-4) | Pearson | -0.070129039 | 0.575796652 | 0.74773025 |
| ctx-rh-lateralorbitofrontal_FA | UPDRS 2 (Years 0-2) | Pearson | -0.068836926 | 0.502875922 | 0.880863766 |
| ctx-rh-lateralorbitofrontal_FA | MoCA (Years 0-4) | Pearson | 0.068575273 | 0.509049308 | 0.793336395 |
| ctx-lh-medialorbitofrontal_FA | UPDRS 2 (Years 0-4) | Pearson | -0.068491443 | 0.505026958 | 0.707037741 |
| ctx-rh-entorhinal_FA | LNS (Years 0-4) | Pearson | 0.06819815 | 0.511387421 | 0.715583688 |
| ctx-lh-insula_FA | UPDRS 1A (Years 2-4) | Pearson | -0.067993868 | 0.58750234 | 0.74773025 |
| ctx-rh-insula_FA | GDS (Years 2-4) | Pearson | -0.066837692 | 0.515389178 | 0.981253447 |
| Olfactory_Right_FA | GDS (Years 2-4) | Pearson | -0.066627162 | 0.516716085 | 0.981253447 |
| ctx-lh-insula_FA | HVLDR (Years 2-4) | Pearson | -0.066397354 | 0.524883871 | 0.97777147 |
| Right-Thalamus-Proper_FA | UPDRS 1B (Years 2-4) | Pearson | 0.066378655 | 0.52500086 | 0.743141341 |
| ctx-lh-medialorbitofrontal_FA | UPDRS 1B (Years 2-4) | Pearson | -0.065451937 | 0.530815244 | 0.743141341 |
| ctx-rh-insula_FA | UPDRS 1 (Years 2-4) | Pearson | -0.065267203 | 0.531978123 | 0.848410466 |
| Olfactory_Right_FA | UPDRS 1A (Years 0-4) | Pearson | 0.064691012 | 0.632588077 | 0.938670598 |
| Olfactory_Left_FA | UPDRS 2 (Years 0-2) | Pearson | -0.064283224 | 0.531605985 | 0.880863766 |
| Left-Thalamus-Proper_FA | SDM (Years 0-4) | Pearson | 0.064128446 | 0.536965479 | 0.578270515 |
| ctx-rh-medialorbitofrontal_FA | LNS (Years 2-4) | Pearson | -0.064030589 | 0.537588208 | 0.904783342 |
| Left-Thalamus-Proper_FA | GDS (Years 0-4) | Pearson | -0.064010774 | 0.533350507 | 0.881549631 |
| ctx-lh-insula_FA | HVLTR (Years 0-4) | Pearson | 0.062988327 | 0.544242873 | 0.623131576 |
| Olfactory_Left_FA | HVLDR (Years 2-4) | Pearson | -0.062450189 | 0.549866692 | 0.97777147 |
| ctx-lh-insula_FA | LNS (Years 0-2) | Pearson | 0.062311877 | 0.544292544 | 0.590265471 |
| ctx-rh-medialorbitofrontal_FA | UPDRS 1 (Years 0-4) | Pearson | -0.061987232 | 0.557206167 | 0.709171485 |
| ctx-lh-lateralorbitofrontal_FA | UPDRS 1B (Years 0-2) | Pearson | -0.060515972 | 0.56879443 | 0.723920184 |
| ctx-rh-entorhinal_FA | UPDRS 2 (Years 0-2) | Pearson | -0.060317177 | 0.557278316 | 0.880863766 |
| ctx-lh-insula_FA | HVLDR (Years 2-4) | Pearson | 0.060246578 | 0.564060245 | 0.789684343 |
| ctx-lh-insula_FA | LNS (Years 0-4) | Pearson | 0.060199563 | 0.562244326 | 0.715583688 |
| Left-Thalamus-Proper_FA | SDM (Years 0-2) | Pearson | 0.059802674 | 0.560651718 | 0.810280936 |
| Left-Thalamus-Proper_FA | LNS (Years 0-2) | Pearson | 0.059271329 | 0.564145736 | 0.590265471 |
| ctx-lh-medialorbitofrontal_FA | UPDRS 2 (Years 2-4) | Pearson | -0.059212019 | 0.570783213 | 0.614689614 |
| ctx-lh-entorhinal_FA | UPDRS 2 (Years 0-4) | Pearson | 0.058892532 | 0.566642911 | 0.721181887 |
| ctx-lh-lateralorbitofrontal_FA | UPDRS 1 (Years 0-2) | Pearson | -0.05741918 | 0.58667262 | 0.821341668 |
| ctx-lh-entorhinal_FA | SF (Years 2-4) | Pearson | 0.057344039 | 0.580964828 | 0.875625543 |
| ctx-lh-insula_FA | UPDRS 1A (Years 0-4) | Pearson | -0.057196961 | 0.672582103 | 0.938670598 |
| ctx-rh-medialorbitofrontal_FA | UPDRS 1B (Years 0-4) | Pearson | -0.057122887 | 0.590698421 | 0.689148158 |
| ctx-rh-insula_FA | MoCA (Years 2-4) | Pearson | 0.056925897 | 0.585770945 | 0.839138642 |
| Left-Thalamus-Proper_FA | MoCA (Years 0-2) | Pearson | -0.056925471 | 0.581706233 | 0.994659941 |
| ctx-rh-medialorbitofrontal_FA | UPDRS 1A (Years 2-4) | Pearson | -0.056544424 | 0.652023308 | 0.760693859 |
| Left-Thalamus-Proper_FA | MoCA (Years 0-4) | Pearson | 0.055934508 | 0.590310125 | 0.793336395 |
| ctx-lh-entorhinal_FA | HVLTR (Years 0-2) | Pearson | -0.055852284 | 0.586871929 | 0.746927909 |
| Left-Thalamus-Proper_FA | LNS (Years 2-4) | Pearson | -0.055669242 | 0.59207644 | 0.904783342 |
| ctx-lh-entorhinal_FA | LNS (Years 0-2) | Pearson | 0.055347018 | 0.590265471 | 0.590265471 |
| ctx-rh-medialorbitofrontal_FA | UPDRS 1A (Years 0-4) | Pearson | 0.05501838 | 0.684386114 | 0.938670598 |
| ctx-lh-lateralorbitofrontal_FA | HVLTR (Years 2-4) | Pearson | 0.054933828 | 0.596985747 | 0.789701549 |
| ctx-rh-medialorbitofrontal_FA | MoCA (Years 2-4) | Pearson | 0.054872404 | 0.599384745 | 0.839138642 |
| Right-Amygdala_FA | SDM (Years 0-2) | Pearson | -0.054640045 | 0.595028624 | 0.810280936 |
| ctx-rh-medialorbitofrontal_FA | UPDRS 1B (Years 2-4) | Pearson | -0.054116188 | 0.60443341 | 0.769278886 |
| Olfactory_Right_FA | UPDRS 1 (Years 2-4) | Pearson | 0.053809232 | 0.606488059 | 0.848410466 |
| Right-Thalamus-Proper_FA | UPDRS 2 (Years 0-2) | Pearson | -0.053760791 | 0.600976462 | 0.880863766 |
| Olfactory_Right_FA | MoCA (Years 0-4) | Pearson | 0.052900397 | 0.610653919 | 0.793336395 |
| ctx-rh-insula_FA | SDM (Years 0-2) | Pearson | 0.052754312 | 0.607817176 | 0.810280936 |
| Right-Thalamus-Proper_FA | UPDRS 1A (Years 0-2) | Pearson | -0.052356832 | 0.69890909 | 0.939000138 |
| ctx-rh-medialorbitofrontal_FA | JLO (Years 0-4) | Pearson | 0.051769613 | 0.620217731 | 0.701972355 |
| Left-Amygdala_FA | HVLTR (Years 0-4) | Pearson | 0.051556191 | 0.619763565 | 0.623131576 |
| Right-Amygdala_FA | LNS (Years 0-4) | Pearson | 0.051546855 | 0.619827042 | 0.723131549 |
| Olfactory_Right_FA | HVLTR (Years 2-4) | Pearson | 0.051450869 | 0.620479789 | 0.789701549 |
| ctx-lh-medialorbitofrontal_FA | UPDRS 2 (Years 0-2) | Pearson | -0.051218253 | 0.618322577 | 0.880863766 |
| ctx-lh-medialorbitofrontal_FA | HVLTR (Years 0-4) | Pearson | 0.051061369 | 0.623131576 | 0.623131576 |
| Right-Thalamus-Proper_FA | HVLDR (Years 0-2) | Pearson | 0.050899018 | 0.6223708 | 0.6223708 |
| ctx-rh-lateralorbitofrontal_FA | SF (Years 0-4) | Pearson | -0.05077496 | 0.625084574 | 0.730999461 |
| ctx-rh-entorhinal_FA | SF (Years 2-4) | Pearson | -0.050721879 | 0.625446817 | 0.875625543 |
| ctx-lh-entorhinal_FA | SDM (Years 2-4) | Pearson | 0.050696625 | 0.625619186 | 0.72988905 |
| Olfactory_Right_FA | SF (Years 0-4) | Pearson | -0.050557234 | 0.626570967 | 0.730999461 |
| ctx-lh-entorhinal_FA | JLO (Years 0-4) | Pearson | -0.04989094 | 0.632980382 | 0.701972355 |
| Left-Amygdala_FA | LNS (Years 2-4) | Pearson | -0.048780828 | 0.638753647 | 0.904783342 |
| ctx-rh-lateralorbitofrontal_FA | SDM (Years 0-2) | Pearson | 0.048565077 | 0.636649307 | 0.810280936 |
| Right-Amygdala_FA | MoCA (Years 0-4) | Pearson | 0.048454732 | 0.641000615 | 0.793336395 |
| ctx-lh-insula_FA | UPDRS 1 (Years 2-4) | Pearson | -0.048412184 | 0.643102622 | 0.848410466 |
| ctx-rh-insula_FA | LNS (Years 2-4) | Pearson | -0.047997959 | 0.644153456 | 0.904783342 |
| ctx-lh-medialorbitofrontal_FA | JLO (Years 0-4) | Pearson | 0.047144622 | 0.651831472 | 0.701972355 |
| ctx-lh-lateralorbitofrontal_FA | GDS (Years 0-2) | Pearson | 0.047124225 | 0.646695341 | 0.925650844 |
| ctx-lh-medialorbitofrontal_FA | HVLTR (Years 0-2) | Pearson | 0.046353331 | 0.65209653 | 0.748318166 |
| ctx-rh-insula_FA | GDS (Years 0-4) | Pearson | -0.045261404 | 0.659777772 | 0.881549631 |
| Right-Amygdala_FA | UPDRS 1 (Years 2-4) | Pearson | -0.04501417 | 0.666608223 | 0.848410466 |
| Right-Amygdala_FA | GDS (Years 0-4) | Pearson | 0.044358041 | 0.666159384 | 0.881549631 |
| ctx-lh-medialorbitofrontal_FA | MoCA (Years 0-2) | Pearson | -0.044098504 | 0.669653943 | 0.994659941 |
| ctx-rh-lateralorbitofrontal_FA | MoCA (Years 0-2) | Pearson | -0.043625373 | 0.672994805 | 0.994659941 |
| ctx-lh-insula_FA | UPDRS 2 (Years 0-2) | Pearson | -0.042703658 | 0.677908012 | 0.880863766 |
| Left-Thalamus-Proper_FA | UPDRS 1 (Years 0-2) | Pearson | -0.04224919 | 0.689246438 | 0.824619944 |
| Olfactory_Right_FA | MoCA (Years 0-2) | Pearson | 0.041854529 | 0.685555062 | 0.994659941 |
| ctx-lh-entorhinal_FA | MoCA (Years 0-2) | Pearson | -0.041617625 | 0.687241973 | 0.994659941 |
| ctx-rh-insula_FA | SF (Years 0-4) | Pearson | -0.041211086 | 0.691716279 | 0.744925224 |
| Right-Thalamus-Proper_FA | GDS (Years 2-4) | Pearson | -0.041195072 | 0.688688965 | 0.981253447 |
| ctx-rh-medialorbitofrontal_FA | MoCA (Years 0-4) | Pearson | 0.041048238 | 0.692873296 | 0.793336395 |
| ctx-rh-entorhinal_FA | UPDRS 1A (Years 0-2) | Pearson | 0.040958694 | 0.762265276 | 0.939000138 |
| ctx-rh-lateralorbitofrontal_FA | GDS (Years 0-4) | Pearson | 0.040643542 | 0.692646139 | 0.881549631 |
| ctx-rh-lateralorbitofrontal_FA | LNS (Years 2-4) | Pearson | 0.040547538 | 0.696435128 | 0.904783342 |
| ctx-lh-lateralorbitofrontal_FA | MoCA (Years 0-2) | Pearson | -0.040519963 | 0.695077938 | 0.994659941 |
| ctx-rh-entorhinal_FA | HVLTR (Years 0-2) | Pearson | 0.040334534 | 0.694866869 | 0.748318166 |
| Left-Thalamus-Proper_FA | UPDRS 1B (Years 2-4) | Pearson | -0.039673335 | 0.704205077 | 0.805634302 |
| Right-Amygdala_FA | MoCA (Years 2-4) | Pearson | 0.039213568 | 0.707476694 | 0.861503952 |
| ctx-rh-entorhinal_FA | UPDRS 1 (Years 0-4) | Pearson | -0.038629289 | 0.7146713 | 0.833783184 |
| Olfactory_Right_FA | SDM (Years 0-2) | Pearson | 0.038360253 | 0.709115468 | 0.827301379 |
| ctx-lh-lateralorbitofrontal_FA | SF (Years 0-2) | Pearson | -0.03796461 | 0.711983144 | 0.950807192 |
| Right-Amygdala_FA | SF (Years 0-2) | Pearson | -0.037795332 | 0.713211327 | 0.950807192 |
| ctx-rh-insula_FA | UPDRS 2 (Years 0-2) | Pearson | -0.037765557 | 0.713427429 | 0.880863766 |
| Left-Thalamus-Proper_FA | LNS (Years 0-4) | Pearson | 0.037436357 | 0.71871227 | 0.773997829 |
| ctx-lh-medialorbitofrontal_FA | MoCA (Years 0-4) | Pearson | 0.036800662 | 0.723293884 | 0.793336395 |
| Right-Thalamus-Proper_FA | UPDRS 1 (Years 2-4) | Pearson | 0.036092167 | 0.729825929 | 0.851463584 |
| ctx-rh-entorhinal_FA | LNS (Years 2-4) | Pearson | -0.035937177 | 0.729532857 | 0.904783342 |
| ctx-rh-medialorbitofrontal_FA | UPDRS 1 (Years 0-2) | Pearson | 0.035628392 | 0.735988619 | 0.824619944 |
| Left-Thalamus-Proper_FA | SDM (Years 2-4) | Pearson | 0.035281786 | 0.734280097 | 0.790763182 |
| ctx-lh-entorhinal_FA | MoCA (Years 0-4) | Pearson | 0.034952434 | 0.73666951 | 0.793336395 |
| ctx-rh-lateralorbitofrontal_FA | HVLDR (Years 2-4) | Pearson | -0.034115384 | 0.744098739 | 0.97777147 |
| Olfactory_Right_FA | UPDRS 1B (Years 2-4) | Pearson | -0.033564906 | 0.748088994 | 0.805634302 |
| Left-Amygdala_FA | GDS (Years 0-2) | Pearson | -0.032412984 | 0.752628559 | 0.925650844 |
| Left-Amygdala_FA | HVLDR (Years 2-4) | Pearson | -0.032297621 | 0.757300282 | 0.97777147 |
| Olfactory_Right_FA | HVLDR (Years 0-4) | Pearson | 0.032209652 | 0.759226155 | 0.759226155 |
| ctx-rh-medialorbitofrontal_FA | UPDRS 2 (Years 0-2) | Pearson | 0.032088554 | 0.755026085 | 0.880863766 |
| ctx-rh-entorhinal_FA | UPDRS 1 (Years 0-2) | Pearson | -0.031489389 | 0.765718519 | 0.824619944 |
| Right-Amygdala_FA | MoCA (Years 0-2) | Pearson | 0.031180111 | 0.762977698 | 0.994659941 |
| Right-Thalamus-Proper_FA | HVLDR (Years 2-4) | Pearson | 0.029700813 | 0.776280204 | 0.97777147 |
| ctx-rh-entorhinal_FA | UPDRS 1A (Years 2-4) | Pearson | 0.029660899 | 0.813111265 | 0.827903013 |
| ctx-lh-insula_FA | LNS (Years 2-4) | Pearson | -0.029206527 | 0.778739993 | 0.904783342 |
| ctx-rh-medialorbitofrontal_FA | HVLTR (Years 2-4) | Pearson | -0.028964609 | 0.780526306 | 0.824916096 |
| ctx-lh-insula_FA | GDS (Years 0-4) | Pearson | -0.028205598 | 0.783895367 | 0.91384007 |
| ctx-rh-entorhinal_FA | MoCA (Years 2-4) | Pearson | -0.028091451 | 0.788110033 | 0.861503952 |
| ctx-lh-medialorbitofrontal_FA | HVLTR (Years 2-4) | Pearson | -0.027966386 | 0.787909129 | 0.824916096 |
| Right-Amygdala_FA | HVLTR (Years 0-2) | Pearson | 0.027906802 | 0.786129618 | 0.786129618 |
| ctx-rh-medialorbitofrontal_FA | UPDRS 2 (Years 0-4) | Pearson | -0.027623013 | 0.788253252 | 0.858537035 |
| Left-Amygdala_FA | UPDRS 1A (Years 2-4) | Pearson | -0.027275246 | 0.827903013 | 0.827903013 |
| Left-Thalamus-Proper_FA | UPDRS 1B (Years 0-2) | Pearson | -0.027138634 | 0.798449473 | 0.931524385 |
| ctx-lh-medialorbitofrontal_FA | SDM (Years 2-4) | Pearson | 0.026936224 | 0.795547863 | 0.795547863 |
| ctx-rh-lateralorbitofrontal_FA | UPDRS 1A (Years 0-2) | Pearson | 0.026888548 | 0.842620674 | 0.939000138 |
| Olfactory_Right_FA | MoCA (Years 2-4) | Pearson | 0.026484818 | 0.799967955 | 0.861503952 |
| Olfactory_Left_FA | GDS (Years 2-4) | Pearson | -0.025821827 | 0.801767338 | 0.981253447 |
| ctx-lh-medialorbitofrontal_FA | UPDRS 1A (Years 0-2) | Pearson | -0.025810503 | 0.848855004 | 0.939000138 |
| ctx-rh-medialorbitofrontal_FA | SF (Years 2-4) | Pearson | 0.025357956 | 0.807288095 | 0.904431371 |
| ctx-rh-entorhinal_FA | UPDRS 2 (Years 0-4) | Pearson | -0.024482415 | 0.811854994 | 0.858537035 |
| ctx-lh-lateralorbitofrontal_FA | UPDRS 1A (Years 0-2) | Pearson | 0.024134438 | 0.858565834 | 0.939000138 |
| Left-Thalamus-Proper_FA | GDS (Years 2-4) | Pearson | 0.023838289 | 0.816717252 | 0.981253447 |
| ctx-rh-insula_FA | UPDRS 1A (Years 0-4) | Pearson | -0.023749127 | 0.860801257 | 0.938670598 |
| ctx-rh-medialorbitofrontal_FA | UPDRS 1A (Years 0-2) | Pearson | 0.023514301 | 0.862164159 | 0.939000138 |
| ctx-lh-lateralorbitofrontal_FA | LNS (Years 2-4) | Pearson | 0.023335577 | 0.822394233 | 0.904783342 |
| ctx-lh-lateralorbitofrontal_FA | UPDRS 1A (Years 0-4) | Pearson | -0.023157934 | 0.864233235 | 0.938670598 |
| Left-Thalamus-Proper_FA | HVLTR (Years 2-4) | Pearson | -0.022998821 | 0.824916096 | 0.824916096 |
| ctx-lh-medialorbitofrontal_FA | SF (Years 0-2) | Pearson | -0.022911788 | 0.823723188 | 0.950807192 |
| ctx-lh-lateralorbitofrontal_FA | SF (Years 2-4) | Pearson | -0.022734989 | 0.826893091 | 0.904431371 |
| Right-Thalamus-Proper_FA | GDS (Years 0-2) | Pearson | 0.022002684 | 0.830611047 | 0.925650844 |
| ctx-rh-entorhinal_FA | UPDRS 1A (Years 0-4) | Pearson | 0.021886463 | 0.871622698 | 0.938670598 |
| ctx-rh-insula_FA | SF (Years 0-2) | Pearson | -0.021792184 | 0.83220775 | 0.950807192 |
| ctx-rh-entorhinal_FA | HVLDR (Years 2-4) | Pearson | -0.02168307 | 0.8356688 | 0.97777147 |
| ctx-rh-insula_FA | SF (Years 2-4) | Pearson | -0.021012135 | 0.83982913 | 0.904431371 |
| Olfactory_Right_FA | LNS (Years 2-4) | Pearson | -0.020968682 | 0.84015596 | 0.904783342 |
| Right-Thalamus-Proper_FA | SF (Years 0-2) | Pearson | -0.020369049 | 0.843020237 | 0.950807192 |
| Right-Thalamus-Proper_FA | GDS (Years 0-4) | Pearson | 0.01964065 | 0.848565779 | 0.91384007 |
| ctx-rh-medialorbitofrontal_FA | GDS (Years 0-2) | Pearson | -0.018750035 | 0.855356335 | 0.925650844 |
| Right-Amygdala_FA | GDS (Years 0-2) | Pearson | -0.018688323 | 0.855827259 | 0.925650844 |
| Right-Amygdala_FA | UPDRS 2 (Years 0-4) | Pearson | -0.01833335 | 0.858537035 | 0.858537035 |
| Olfactory_Left_FA | MoCA (Years 0-2) | Pearson | 0.018140204 | 0.860747174 | 0.994659941 |
| Left-Thalamus-Proper_FA | HVLDR (Years 2-4) | Pearson | 0.017516219 | 0.866924239 | 0.960142181 |
| ctx-rh-entorhinal_FA | GDS (Years 2-4) | Pearson | -0.017180322 | 0.867350125 | 0.981253447 |
| ctx-rh-insula_FA | GDS (Years 0-2) | Pearson | -0.016089621 | 0.875701802 | 0.925650844 |
| ctx-lh-entorhinal_FA | SDM (Years 0-2) | Pearson | -0.015816381 | 0.877796213 | 0.945318999 |
| ctx-lh-insula_FA | UPDRS 1A (Years 0-2) | Pearson | 0.015117742 | 0.911129372 | 0.939000138 |
| Right-Amygdala_FA | HVLDR (Years 2-4) | Pearson | 0.01491479 | 0.886544986 | 0.97777147 |
| Left-Amygdala_FA | UPDRS 1 (Years 2-4) | Pearson | 0.014762808 | 0.88769352 | 0.938609027 |
| Right-Amygdala_FA | UPDRS 2 (Years 0-2) | Pearson | 0.013491273 | 0.895651071 | 0.964547307 |
| ctx-rh-medialorbitofrontal_FA | GDS (Years 2-4) | Pearson | 0.01345606 | 0.8959219 | 0.981253447 |
| Olfactory_Right_FA | JLO (Years 0-2) | Pearson | 0.011670745 | 0.909668039 | 0.949521093 |
| ctx-rh-medialorbitofrontal_FA | UPDRS 1B (Years 0-2) | Pearson | 0.011517388 | 0.91371517 | 0.964298316 |
| ctx-lh-entorhinal_FA | HVLDR (Years 0-4) | Pearson | -0.011401683 | 0.913148646 | 0.913148646 |
| Olfactory_Right_FA | UPDRS 1A (Years 0-2) | Pearson | 0.010365576 | 0.939000138 | 0.939000138 |
| ctx-lh-entorhinal_FA | UPDRS 1 (Years 0-4) | Pearson | 0.010345687 | 0.92202903 | 0.936078401 |
| Right-Thalamus-Proper_FA | HVLDR (Years 2-4) | Pearson | 0.010064609 | 0.923300728 | 0.960142181 |
| Right-Thalamus-Proper_FA | LNS (Years 2-4) | Pearson | 0.010015545 | 0.923258844 | 0.923258844 |
| ctx-rh-entorhinal_FA | UPDRS 2 (Years 2-4) | Pearson | -0.010003688 | 0.923763592 | 0.923763592 |
| Right-Thalamus-Proper_FA | UPDRS 1B (Years 0-4) | Pearson | 0.009760831 | 0.92683518 | 0.991426786 |
| ctx-lh-insula_FA | GDS (Years 0-2) | Pearson | 0.009599243 | 0.925650844 | 0.925650844 |
| Left-Amygdala_FA | MoCA (Years 2-4) | Pearson | 0.009046064 | 0.93104263 | 0.93104263 |
| Right-Thalamus-Proper_FA | UPDRS 1 (Years 0-4) | Pearson | -0.008477157 | 0.936078401 | 0.936078401 |
| Left-Amygdala_FA | MoCA (Years 0-4) | Pearson | 0.008340217 | 0.93606577 | 0.93606577 |
| ctx-rh-medialorbitofrontal_FA | UPDRS 1 (Years 2-4) | Pearson | -0.008051426 | 0.938609027 | 0.938609027 |
| Right-Thalamus-Proper_FA | UPDRS 1 (Years 0-2) | Pearson | 0.007841199 | 0.94086482 | 0.94086482 |
| Left-Amygdala_FA | HVLDR (Years 2-4) | Pearson | -0.007473925 | 0.943004728 | 0.960142181 |
| Olfactory_Left_FA | JLO (Years 0-4) | Pearson | -0.007218023 | 0.944953109 | 0.944953109 |
| ctx-lh-entorhinal_FA | JLO (Years 0-2) | Pearson | -0.007064688 | 0.945245966 | 0.949521093 |
| ctx-lh-insula_FA | SF (Years 2-4) | Pearson | 0.006946282 | 0.946734161 | 0.946734161 |
| Right-Amygdala_FA | JLO (Years 0-2) | Pearson | 0.006512329 | 0.949521093 | 0.949521093 |
| Left-Thalamus-Proper_FA | SF (Years 0-2) | Pearson | -0.006498253 | 0.949630059 | 0.950807192 |
| ctx-lh-medialorbitofrontal_FA | HVLDR (Years 2-4) | Pearson | 0.006474933 | 0.950612613 | 0.97777147 |
| ctx-lh-entorhinal_FA | UPDRS 1A (Years 0-4) | Pearson | -0.006423161 | 0.962178566 | 0.962178566 |
| ctx-lh-insula_FA | SF (Years 0-2) | Pearson | -0.006346199 | 0.950807192 | 0.950807192 |
| Left-Amygdala_FA | SDM (Years 0-2) | Pearson | -0.005851124 | 0.954640541 | 0.954640541 |
| ctx-rh-entorhinal_FA | HVLDR (Years 2-4) | Pearson | 0.005224413 | 0.960142181 | 0.960142181 |
| Right-Thalamus-Proper_FA | UPDRS 1B (Years 0-2) | Pearson | -0.004757908 | 0.964298316 | 0.964298316 |
| ctx-lh-lateralorbitofrontal_FA | GDS (Years 2-4) | Pearson | -0.004297569 | 0.966675966 | 0.981253447 |
| Left-Thalamus-Proper_FA | UPDRS 2 (Years 0-2) | Pearson | 0.003514783 | 0.972743235 | 0.972743235 |
| ctx-rh-medialorbitofrontal_FA | GDS (Years 0-4) | Pearson | 0.003133324 | 0.97570045 | 0.97570045 |
| ctx-rh-medialorbitofrontal_FA | HVLDR (Years 2-4) | Pearson | -0.00291281 | 0.97777147 | 0.97777147 |
| ctx-lh-insula_FA | SF (Years 0-4) | Pearson | -0.002650758 | 0.979660613 | 0.979660613 |
| ctx-lh-entorhinal_FA | GDS (Years 2-4) | Pearson | -0.00241714 | 0.981253447 | 0.981253447 |
| Left-Amygdala_FA | UPDRS 1B (Years 2-4) | Pearson | 0.001663981 | 0.987300569 | 0.987300569 |
| Left-Amygdala_FA | MoCA (Years 0-2) | Pearson | 0.001655533 | 0.987227719 | 0.994659941 |
| ctx-rh-medialorbitofrontal_FA | MoCA (Years 0-2) | Pearson | 0.001344771 | 0.989625071 | 0.994659941 |
| ctx-lh-entorhinal_FA | UPDRS 1B (Years 0-4) | Pearson | 0.001142186 | 0.991426786 | 0.991426786 |
| ctx-lh-entorhinal_FA | LNS (Years 0-4) | Pearson | -0.001037297 | 0.992040053 | 0.992040053 |
| Right-Thalamus-Proper_FA | MoCA (Years 0-2) | Pearson | 0.00069215 | 0.994659941 | 0.994659941 |
| ctx-lh-entorhinal_FA | SDM (Years 0-4) | Pearson | -0.000290542 | 0.997770423 | 0.997770423 |
| Right-Thalamus-Proper_MD | HVLDR (Years 0-4) | Pearson | -0.418657675 | 2.68E-05 | 0.000344159 |
| Right-Amygdala_MD | HVLDR (Years 0-4) | Pearson | -0.405963841 | 4.92E-05 | 0.000344159 |
| Left-Thalamus-Proper_MD | HVLDR (Years 0-4) | Pearson | -0.392588296 | 9.09E-05 | 0.000424161 |
| ctx-lh-insula_MD | SDM (Years 0-4) | Pearson | -0.391133195 | 8.89E-05 | 0.001244022 |
| Right-Thalamus-Proper_MD | HVLTR (Years 0-4) | Pearson | -0.38247294 | 0.000130752 | 0.001830525 |
| ctx-rh-insula_MD | HVLTR (Years 0-2) | Pearson | -0.367487207 | 0.000213188 | 0.002984635 |
| ctx-lh-insula_MD | HVLDR (Years 0-4) | Pearson | -0.364623519 | 0.000302582 | 0.001006766 |
| ctx-rh-insula_MD | SDM (Years 0-4) | Pearson | -0.362196051 | 0.000310008 | 0.002170056 |
| ctx-rh-insula_MD | HVLDR (Years 0-4) | Pearson | -0.360396403 | 0.000359559 | 0.001006766 |
| Left-Amygdala_MD | JLO (Years 2-4) | Pearson | -0.351678452 | 0.00050942 | 0.002920738 |
| ctx-lh-insula_MD | HVLTR (Years 0-2) | Pearson | -0.34938113 | 0.000452139 | 0.003164973 |
| Left-Thalamus-Proper_MD | JLO (Years 2-4) | Pearson | -0.347113813 | 0.000608965 | 0.002920738 |
| ctx-rh-insula_MD | JLO (Years 2-4) | Pearson | -0.346407364 | 0.000625872 | 0.002920738 |
| Right-Thalamus-Proper_MD | HVLDR (Years 0-2) | Pearson | -0.341335899 | 0.000665515 | 0.009317212 |
| Right-Thalamus-Proper_MD | SDM (Years 2-4) | Pearson | -0.340785243 | 0.000726589 | 0.005706657 |
| Left-Thalamus-Proper_MD | SDM (Years 2-4) | Pearson | -0.337772136 | 0.000815237 | 0.005706657 |
| ctx-rh-lateralorbitofrontal_MD | LNS (Years 0-4) | Pearson | -0.334150879 | 0.000934785 | 0.013086986 |
| Right-Thalamus-Proper_MD | HVLTR (Years 0-2) | Pearson | -0.331628415 | 0.000905842 | 0.004227262 |
| ctx-lh-insula_MD | HVLTR (Years 0-4) | Pearson | -0.322006691 | 0.001461646 | 0.007080428 |
| Left-Thalamus-Proper_MD | HVLTR (Years 0-4) | Pearson | -0.320969844 | 0.001517235 | 0.007080428 |
| Olfactory_Left_MD | HVLDR (Years 0-4) | Pearson | -0.313814431 | 0.002069755 | 0.004829428 |
| ctx-lh-insula_MD | JLO (Years 2-4) | Pearson | -0.312256651 | 0.002184486 | 0.006849754 |
| ctx-lh-medialorbitofrontal_MD | LNS (Years 0-4) | Pearson | -0.311179265 | 0.002144626 | 0.015012381 |
| Right-Thalamus-Proper_MD | JLO (Years 2-4) | Pearson | -0.308960606 | 0.002446341 | 0.006849754 |
| ctx-rh-insula_MD | HVLTR (Years 0-4) | Pearson | -0.308866496 | 0.002323438 | 0.008132032 |
| Olfactory_Right_MD | HVLDR (Years 0-4) | Pearson | -0.300894178 | 0.003210203 | 0.005720807 |
| Left-Amygdala_MD | HVLDR (Years 0-4) | Pearson | -0.300347059 | 0.003269032 | 0.005720807 |
| Right-Thalamus-Proper_MD | MoCA (Years 0-4) | Pearson | -0.289338363 | 0.004456715 | 0.062394011 |
| Right-Amygdala_MD | JLO (Years 2-4) | Pearson | -0.288122672 | 0.004861723 | 0.011344021 |
| Olfactory_Right_MD | SDM (Years 0-4) | Pearson | -0.284749275 | 0.005161182 | 0.024085515 |
| Right-Amygdala_MD | HVLDR (Years 0-2) | Pearson | -0.283162647 | 0.005181467 | 0.025228015 |
| ctx-rh-medialorbitofrontal_MD | LNS (Years 0-4) | Pearson | -0.28274412 | 0.005498899 | 0.025661527 |
| Left-Thalamus-Proper_MD | HVLDR (Years 0-2) | Pearson | -0.280983542 | 0.005552459 | 0.025228015 |
| Right-Thalamus-Proper_MD | SDM (Years 0-4) | Pearson | -0.274092558 | 0.007191616 | 0.025170655 |
| Right-Amygdala_MD | HVLTR (Years 0-2) | Pearson | -0.273399074 | 0.006736628 | 0.018978648 |
| Left-Thalamus-Proper_MD | HVLTR (Years 0-2) | Pearson | -0.27320152 | 0.006778089 | 0.018978648 |
| ctx-rh-insula_MD | HVLDR (Years 0-2) | Pearson | -0.272605039 | 0.007208004 | 0.025228015 |
| ctx-lh-lateralorbitofrontal_MD | LNS (Years 0-4) | Pearson | -0.264961869 | 0.009461985 | 0.03013798 |
| ctx-lh-lateralorbitofrontal_MD | SDM (Years 0-4) | Pearson | -0.260264433 | 0.010858457 | 0.03040368 |
| Olfactory_Left_MD | HVLDR (Years 0-2) | Pearson | -0.258368636 | 0.011033528 | 0.030893878 |
| Olfactory_Right_MD | LNS (Years 0-4) | Pearson | -0.258059336 | 0.011573959 | 0.03013798 |
| Right-Amygdala_MD | HVLTR (Years 0-4) | Pearson | -0.257806818 | 0.011658464 | 0.0326437 |
| Olfactory_Left_MD | LNS (Years 0-4) | Pearson | -0.254225355 | 0.012916277 | 0.03013798 |
| Left-Thalamus-Proper_MD | HVLDR (Years 0-4) | Pearson | -0.253333296 | 0.014280636 | 0.159839922 |
| Olfactory_Left_MD | JLO (Years 2-4) | Pearson | -0.252613914 | 0.014033998 | 0.025025594 |
| ctx-lh-insula_MD | SDM (Years 2-4) | Pearson | -0.252359378 | 0.013617228 | 0.063547064 |
| Olfactory_Right_MD | JLO (Years 2-4) | Pearson | -0.251941038 | 0.014300339 | 0.025025594 |
| ctx-rh-lateralorbitofrontal_MD | HVLDR (Years 0-4) | Pearson | -0.240304414 | 0.019650205 | 0.030566985 |
| Left-Amygdala_MD | HVLDR (Years 0-2) | Pearson | -0.238648403 | 0.019202399 | 0.044805599 |
| ctx-rh-insula_MD | SDM (Years 2-4) | Pearson | -0.236564902 | 0.020994138 | 0.073479484 |
| ctx-lh-lateralorbitofrontal_MD | HVLDR (Years 0-4) | Pearson | -0.236201714 | 0.021908362 | 0.030671707 |
| Left-Thalamus-Proper_MD | SDM (Years 0-4) | Pearson | -0.231249582 | 0.024148445 | 0.04282046 |
| ctx-rh-lateralorbitofrontal_MD | SDM (Years 0-4) | Pearson | -0.230952898 | 0.024335824 | 0.04282046 |
| Olfactory_Left_MD | HVLTR (Years 0-2) | Pearson | -0.229634555 | 0.023657522 | 0.055200885 |
| Right-Thalamus-Proper_MD | LNS (Years 0-4) | Pearson | -0.227374538 | 0.02669525 | 0.049180034 |
| Right-Amygdala_MD | HVLDR (Years 0-4) | Pearson | -0.226953007 | 0.028692451 | 0.159839922 |
| Right-Amygdala_MD | SDM (Years 0-4) | Pearson | -0.226625972 | 0.027212666 | 0.04282046 |
| Olfactory_Left_MD | SDM (Years 0-4) | Pearson | -0.22617647 | 0.027527439 | 0.04282046 |
| Olfactory_Right_MD | HVLTR (Years 0-4) | Pearson | -0.226168686 | 0.027532916 | 0.064243471 |
| Right-Amygdala_MD | UPDRS 1B (Years 0-2) | Pearson | 0.2261277 | 0.03113824 | 0.435935357 |
| ctx-rh-insula_MD | LNS (Years 0-4) | Pearson | -0.225365887 | 0.028102877 | 0.049180034 |
| Olfactory_Right_MD | SDM (Years 2-4) | Pearson | -0.225144293 | 0.028261951 | 0.079133463 |
| ctx-lh-insula_MD | HVLDR (Years 0-2) | Pearson | -0.221283046 | 0.030261037 | 0.060522075 |
| Left-Amygdala_MD | HVLDR (Years 0-4) | Pearson | -0.21981325 | 0.034251412 | 0.159839922 |
| ctx-lh-insula_MD | MoCA (Years 0-4) | Pearson | -0.214666167 | 0.036705183 | 0.256936281 |
| Left-Amygdala_MD | JLO (Years 0-4) | Pearson | -0.214167199 | 0.038194505 | 0.251636787 |
| ctx-lh-medialorbitofrontal_MD | LNS (Years 0-2) | Pearson | -0.214103637 | 0.035219548 | 0.249096667 |
| ctx-lh-lateralorbitofrontal_MD | HVLTR (Years 0-4) | Pearson | -0.213692241 | 0.037587516 | 0.067704053 |
| Olfactory_Left_MD | HVLTR (Years 0-4) | Pearson | -0.212503952 | 0.03868803 | 0.067704053 |
| ctx-lh-insula_MD | LNS (Years 0-4) | Pearson | -0.211745592 | 0.039404354 | 0.05747438 |
| ctx-rh-lateralorbitofrontal_MD | JLO (Years 2-4) | Pearson | -0.211308731 | 0.040909156 | 0.063636465 |
| Left-Amygdala_MD | LNS (Years 0-4) | Pearson | -0.21004286 | 0.041053129 | 0.05747438 |
| Right-Amygdala_MD | UPDRS 1A (Years 0-2) | Pearson | -0.207541749 | 0.12135465 | 0.992597828 |
| ctx-lh-insula_MD | SDM (Years 0-2) | Pearson | -0.206838297 | 0.042077735 | 0.451647985 |
| Left-Thalamus-Proper_MD | HVLDR (Years 0-2) | Pearson | -0.206639461 | 0.043385432 | 0.529305237 |
| Right-Amygdala_MD | UPDRS 1B (Years 0-4) | Pearson | 0.206157624 | 0.049931874 | 0.69624045 |
| ctx-rh-lateralorbitofrontal_MD | LNS (Years 0-2) | Pearson | -0.204181015 | 0.04484909 | 0.249096667 |
| ctx-lh-lateralorbitofrontal_MD | SDM (Years 2-4) | Pearson | -0.203394456 | 0.048051084 | 0.101128971 |
| Left-Amygdala_MD | SDM (Years 0-4) | Pearson | -0.202438004 | 0.049134567 | 0.068788394 |
| ctx-rh-lateralorbitofrontal_MD | SDM (Years 2-4) | Pearson | -0.201202034 | 0.050564486 | 0.101128971 |
| ctx-lh-insula_MD | MoCA (Years 2-4) | Pearson | -0.200956656 | 0.052120189 | 0.349599365 |
| Right-Thalamus-Proper_MD | MoCA (Years 2-4) | Pearson | -0.198826588 | 0.054715298 | 0.349599365 |
| Left-Amygdala_MD | LNS (Years 2-4) | Pearson | -0.197974453 | 0.054460916 | 0.374224326 |
| ctx-rh-medialorbitofrontal_MD | LNS (Years 0-2) | Pearson | -0.196778376 | 0.053377857 | 0.249096667 |
| Right-Thalamus-Proper_MD | HVLDR (Years 0-4) | Pearson | -0.195623123 | 0.060216338 | 0.210757182 |
| ctx-lh-medialorbitofrontal_MD | HVLDR (Years 0-4) | Pearson | -0.195069517 | 0.059550523 | 0.075791575 |
| Left-Amygdala_MD | HVLTR (Years 0-4) | Pearson | -0.192038475 | 0.062269255 | 0.096863286 |
| Olfactory_Left_MD | GDS (Years 0-4) | Pearson | 0.187875512 | 0.065355558 | 0.91497781 |
| Left-Thalamus-Proper_MD | LNS (Years 0-4) | Pearson | -0.186342307 | 0.07059594 | 0.089849378 |
| ctx-rh-insula_MD | SDM (Years 0-2) | Pearson | -0.186132151 | 0.067938992 | 0.451647985 |
| ctx-rh-insula_MD | UPDRS 1A (Years 2-4) | Pearson | 0.18484679 | 0.137319531 | 0.565713221 |
| Right-Amygdala_MD | JLO (Years 0-4) | Pearson | -0.184530089 | 0.074998822 | 0.251636787 |
| Left-Thalamus-Proper_MD | JLO (Years 0-4) | Pearson | -0.182878077 | 0.077688878 | 0.251636787 |
| ctx-lh-insula_MD | JLO (Years 0-4) | Pearson | -0.182703 | 0.077978438 | 0.251636787 |
| Right-Thalamus-Proper_MD | MoCA (Years 0-2) | Pearson | -0.182633282 | 0.074911766 | 0.810613862 |
| Olfactory_Right_MD | HVLTR (Years 0-2) | Pearson | -0.181890718 | 0.074570293 | 0.149140586 |
| ctx-rh-insula_MD | MoCA (Years 0-4) | Pearson | -0.180911758 | 0.079349254 | 0.369738183 |
| ctx-rh-insula_MD | MoCA (Years 2-4) | Pearson | -0.179643963 | 0.083178654 | 0.349599365 |
| ctx-lh-medialorbitofrontal_MD | SDM (Years 0-4) | Pearson | -0.178153868 | 0.084115847 | 0.107056533 |
| Olfactory_Right_MD | HVLDR (Years 0-2) | Pearson | -0.176369084 | 0.08562846 | 0.149849805 |
| Olfactory_Left_MD | HVLDR (Years 0-4) | Pearson | -0.173631699 | 0.096019416 | 0.268854365 |
| ctx-lh-lateralorbitofrontal_MD | UPDRS 1 (Years 0-4) | Pearson | 0.171721072 | 0.101682045 | 0.606939698 |
| Left-Amygdala_MD | HVLTR (Years 0-2) | Pearson | -0.171687071 | 0.092663651 | 0.162161389 |
| Olfactory_Right_MD | JLO (Years 0-4) | Pearson | -0.171072907 | 0.099229908 | 0.251636787 |
| ctx-lh-insula_MD | UPDRS 1 (Years 0-4) | Pearson | 0.16919332 | 0.106895873 | 0.606939698 |
| ctx-lh-insula_MD | UPDRS 2 (Years 2-4) | Pearson | 0.168138449 | 0.10525242 | 0.913899161 |
| Left-Amygdala_MD | MoCA (Years 2-4) | Pearson | -0.164832156 | 0.112375812 | 0.349599365 |
| ctx-rh-insula_MD | JLO (Years 0-4) | Pearson | -0.164139056 | 0.113915431 | 0.251636787 |
| Olfactory_Right_MD | LNS (Years 0-2) | Pearson | -0.163803339 | 0.108888149 | 0.34315935 |
| Left-Amygdala_MD | UPDRS 1 (Years 0-2) | Pearson | 0.163470941 | 0.119467241 | 0.787207997 |
| ctx-lh-insula_MD | LNS (Years 2-4) | Pearson | -0.162698352 | 0.115184888 | 0.374224326 |
| ctx-lh-insula_MD | UPDRS 1B (Years 0-4) | Pearson | 0.161867542 | 0.12530044 | 0.69624045 |
| ctx-rh-lateralorbitofrontal_MD | HVLDR (Years 0-4) | Pearson | -0.161161706 | 0.12276427 | 0.286449962 |
| ctx-lh-medialorbitofrontal_MD | UPDRS 1A (Years 0-4) | Pearson | 0.159581717 | 0.235724887 | 0.995151246 |
| ctx-rh-lateralorbitofrontal_MD | HVLTR (Years 0-4) | Pearson | -0.159370946 | 0.122910881 | 0.172075233 |
| Right-Thalamus-Proper_MD | JLO (Years 0-4) | Pearson | -0.159011313 | 0.125818394 | 0.251636787 |
| Left-Thalamus-Proper_MD | MoCA (Years 0-4) | Pearson | -0.157551602 | 0.127300401 | 0.369738183 |
| Right-Amygdala_MD | SF (Years 0-2) | Pearson | -0.156075 | 0.126859262 | 0.957648714 |
| ctx-rh-lateralorbitofrontal_MD | MoCA (Years 2-4) | Pearson | -0.156071363 | 0.133060111 | 0.349599365 |
| ctx-rh-lateralorbitofrontal_MD | LNS (Years 2-4) | Pearson | -0.155217066 | 0.133107473 | 0.374224326 |
| ctx-lh-lateralorbitofrontal_MD | HVLTR (Years 0-2) | Pearson | -0.154692 | 0.130302139 | 0.188630126 |
| Left-Amygdala_MD | SDM (Years 2-4) | Pearson | -0.154550365 | 0.13480234 | 0.235904096 |
| Right-Amygdala_MD | UPDRS 1A (Years 2-4) | Pearson | 0.154532089 | 0.215384234 | 0.565713221 |
| ctx-lh-lateralorbitofrontal_MD | LNS (Years 0-2) | Pearson | -0.154511465 | 0.130756784 | 0.34315935 |
| Olfactory_Left_MD | GDS (Years 0-2) | Pearson | 0.153391033 | 0.133605501 | 0.98559754 |
| ctx-rh-lateralorbitofrontal_MD | HVLTR (Years 0-2) | Pearson | -0.152951514 | 0.134735804 | 0.188630126 |
| Right-Amygdala_MD | MoCA (Years 0-4) | Pearson | -0.152804144 | 0.139319287 | 0.369738183 |
| ctx-rh-lateralorbitofrontal_MD | HVLDR (Years 0-2) | Pearson | -0.152177894 | 0.138836874 | 0.21596847 |
| Olfactory_Left_MD | SDM (Years 0-2) | Pearson | -0.150613225 | 0.140871758 | 0.451647985 |
| Right-Amygdala_MD | SDM (Years 0-2) | Pearson | -0.149810446 | 0.143026383 | 0.451647985 |
| ctx-lh-lateralorbitofrontal_MD | MoCA (Years 2-4) | Pearson | -0.149703466 | 0.149828299 | 0.349599365 |
| Right-Thalamus-Proper_MD | JLO (Years 0-2) | Pearson | 0.149421289 | 0.144079777 | 0.535331873 |
| Right-Amygdala_MD | HVLDR (Years 0-2) | Pearson | -0.148993387 | 0.147391149 | 0.529305237 |
| ctx-lh-medialorbitofrontal_MD | UPDRS 1 (Years 0-4) | Pearson | 0.148651798 | 0.157305027 | 0.606939698 |
| Right-Thalamus-Proper_MD | LNS (Years 2-4) | Pearson | -0.148421393 | 0.151160169 | 0.374224326 |
| ctx-lh-entorhinal_MD | LNS (Years 0-2) | Pearson | -0.148328793 | 0.147068293 | 0.34315935 |
| ctx-lh-lateralorbitofrontal_MD | UPDRS 1A (Years 2-4) | Pearson | 0.147788919 | 0.23632652 | 0.565713221 |
| ctx-rh-lateralorbitofrontal_MD | MoCA (Years 0-4) | Pearson | -0.145846724 | 0.158459221 | 0.369738183 |
| Olfactory_Left_MD | UPDRS 1A (Years 0-4) | Pearson | -0.145782022 | 0.279235025 | 0.995151246 |
| Right-Amygdala_MD | LNS (Years 0-4) | Pearson | -0.144699592 | 0.161794535 | 0.188760291 |
| ctx-rh-medialorbitofrontal_MD | HVLDR (Years 0-4) | Pearson | -0.144058388 | 0.165976823 | 0.193639627 |
| ctx-lh-entorhinal_MD | HVLDR (Years 0-4) | Pearson | -0.142669526 | 0.172492985 | 0.318889491 |
| Left-Amygdala_MD | UPDRS 1B (Years 0-2) | Pearson | 0.142478459 | 0.177898586 | 0.69043972 |
| ctx-rh-lateralorbitofrontal_MD | SF (Years 2-4) | Pearson | -0.1423302 | 0.168848184 | 0.845399689 |
| ctx-rh-medialorbitofrontal_MD | HVLDR (Years 2-4) | Pearson | -0.141782647 | 0.172838887 | 0.585178219 |
| ctx-lh-medialorbitofrontal_MD | LNS (Years 2-4) | Pearson | -0.141107635 | 0.172575326 | 0.374224326 |
| Right-Amygdala_MD | LNS (Years 2-4) | Pearson | -0.140307868 | 0.175046047 | 0.374224326 |
| ctx-lh-medialorbitofrontal_MD | HVLTR (Years 0-4) | Pearson | -0.14011024 | 0.175660564 | 0.22356799 |
| ctx-rh-medialorbitofrontal_MD | HVLDR (Years 0-4) | Pearson | -0.139532848 | 0.182222566 | 0.318889491 |
| ctx-rh-insula_MD | UPDRS 1B (Years 0-2) | Pearson | 0.139428038 | 0.187463213 | 0.69043972 |
| ctx-rh-insula_MD | JLO (Years 0-2) | Pearson | 0.13921904 | 0.173827837 | 0.535331873 |
| Left-Thalamus-Proper_MD | MoCA (Years 0-2) | Pearson | -0.137881506 | 0.180353037 | 0.810613862 |
| ctx-lh-entorhinal_MD | HVLDR (Years 2-4) | Pearson | -0.137352463 | 0.186791591 | 0.585178219 |
| ctx-rh-medialorbitofrontal_MD | SDM (Years 0-4) | Pearson | -0.1373102 | 0.184537895 | 0.215294211 |
| Right-Thalamus-Proper_MD | HVLDR (Years 0-2) | Pearson | -0.136627962 | 0.184385274 | 0.529305237 |
| ctx-lh-insula_MD | UPDRS 1B (Years 0-2) | Pearson | 0.136415959 | 0.197268491 | 0.69043972 |
| ctx-lh-medialorbitofrontal_MD | UPDRS 1A (Years 2-4) | Pearson | 0.136177082 | 0.275599351 | 0.565713221 |
| Right-Amygdala_MD | UPDRS 1 (Years 0-4) | Pearson | 0.13609032 | 0.195833925 | 0.606939698 |
| Left-Amygdala_MD | UPDRS 1B (Years 0-4) | Pearson | 0.135068449 | 0.201772532 | 0.69624045 |
| Olfactory_Left_MD | UPDRS 1 (Years 2-4) | Pearson | 0.134911742 | 0.194819566 | 0.984258963 |
| ctx-rh-medialorbitofrontal_MD | UPDRS 1A (Years 0-2) | Pearson | 0.134856953 | 0.317228641 | 0.992597828 |
| Left-Thalamus-Proper_MD | JLO (Years 0-2) | Pearson | 0.1337469 | 0.191534106 | 0.535331873 |
| ctx-lh-lateralorbitofrontal_MD | JLO (Years 2-4) | Pearson | -0.132635189 | 0.202529249 | 0.283540948 |
| Left-Amygdala_MD | MoCA (Years 0-4) | Pearson | -0.132533391 | 0.200428716 | 0.400857431 |
| ctx-lh-insula_MD | UPDRS 1A (Years 2-4) | Pearson | 0.131696441 | 0.291853627 | 0.565713221 |
| ctx-rh-medialorbitofrontal_MD | HVLTR (Years 0-4) | Pearson | -0.131445799 | 0.204180141 | 0.237176892 |
| ctx-lh-medialorbitofrontal_MD | SDM (Years 0-2) | Pearson | -0.1308988 | 0.201250272 | 0.451647985 |
| ctx-rh-medialorbitofrontal_MD | LNS (Years 2-4) | Pearson | -0.130545187 | 0.207324447 | 0.374224326 |
| ctx-lh-medialorbitofrontal_MD | HVLDR (Years 0-4) | Pearson | -0.130427037 | 0.212723212 | 0.326430336 |
| ctx-lh-medialorbitofrontal_MD | UPDRS 1A (Years 0-2) | Pearson | 0.130155471 | 0.334547327 | 0.992597828 |
| ctx-rh-insula_MD | UPDRS 1 (Years 0-4) | Pearson | 0.130006441 | 0.216764178 | 0.606939698 |
| ctx-lh-lateralorbitofrontal_MD | SDM (Years 0-2) | Pearson | -0.129802793 | 0.205081961 | 0.451647985 |
| ctx-rh-lateralorbitofrontal_MD | UPDRS 1A (Years 2-4) | Pearson | 0.129799832 | 0.298919541 | 0.565713221 |
| Right-Amygdala_MD | UPDRS 1 (Years 0-2) | Pearson | 0.129730695 | 0.217748931 | 0.787207997 |
| Right-Amygdala_MD | MoCA (Years 2-4) | Pearson | -0.129629896 | 0.213038337 | 0.426076675 |
| ctx-lh-lateralorbitofrontal_MD | UPDRS 1B (Years 0-4) | Pearson | 0.128862646 | 0.223466893 | 0.69624045 |
| ctx-rh-insula_MD | LNS (Years 2-4) | Pearson | -0.128395904 | 0.21496752 | 0.374224326 |
| ctx-rh-entorhinal_MD | JLO (Years 0-4) | Pearson | 0.128046808 | 0.218727306 | 0.382772786 |
| ctx-lh-medialorbitofrontal_MD | JLO (Years 0-2) | Pearson | 0.12748571 | 0.213353765 | 0.535331873 |
| Left-Amygdala_MD | HVLDR (Years 2-4) | Pearson | -0.127171274 | 0.221919317 | 0.585178219 |
| ctx-rh-entorhinal_MD | HVLTR (Years 0-4) | Pearson | -0.126945833 | 0.220235686 | 0.237176892 |
| ctx-lh-insula_MD | JLO (Years 0-2) | Pearson | 0.126495097 | 0.21696153 | 0.535331873 |
| ctx-lh-insula_MD | HVLDR (Years 0-2) | Pearson | -0.125936232 | 0.221469943 | 0.529305237 |
| Olfactory_Right_MD | SF (Years 2-4) | Pearson | -0.125830583 | 0.224348953 | 0.845399689 |
| Right-Thalamus-Proper_MD | UPDRS 1A (Years 2-4) | Pearson | 0.125796847 | 0.314195791 | 0.565713221 |
| ctx-rh-insula_MD | UPDRS 2 (Years 2-4) | Pearson | 0.125640042 | 0.227580472 | 0.913899161 |
| Right-Amygdala_MD | UPDRS 2 (Years 2-4) | Pearson | -0.125343297 | 0.228689184 | 0.913899161 |
| ctx-lh-insula_MD | HVLDR (Years 0-4) | Pearson | -0.124834291 | 0.233164526 | 0.326430336 |
| ctx-rh-medialorbitofrontal_MD | SDM (Years 2-4) | Pearson | -0.124383602 | 0.229765802 | 0.344833487 |
| Olfactory_Right_MD | SDM (Years 0-2) | Pearson | -0.124110153 | 0.225823993 | 0.451647985 |
| ctx-rh-medialorbitofrontal_MD | JLO (Years 0-2) | Pearson | 0.123159133 | 0.229427946 | 0.535331873 |
| Olfactory_Left_MD | SF (Years 2-4) | Pearson | -0.123019407 | 0.234955949 | 0.845399689 |
| ctx-lh-medialorbitofrontal_MD | UPDRS 1B (Years 0-4) | Pearson | 0.122167118 | 0.248657304 | 0.69624045 |
| Right-Amygdala_MD | HVLDR (Years 2-4) | Pearson | -0.122133145 | 0.240925246 | 0.585178219 |
| ctx-lh-insula_MD | UPDRS 1 (Years 0-2) | Pearson | 0.12185748 | 0.247209717 | 0.787207997 |
| ctx-lh-entorhinal_MD | LNS (Years 0-4) | Pearson | -0.121622399 | 0.240354992 | 0.258843837 |
| ctx-lh-lateralorbitofrontal_MD | LNS (Years 2-4) | Pearson | -0.121566505 | 0.240572781 | 0.374224326 |
| Left-Amygdala_MD | HVLDR (Years 0-2) | Pearson | -0.1203538 | 0.242796399 | 0.529305237 |
| ctx-lh-medialorbitofrontal_MD | SDM (Years 2-4) | Pearson | -0.120106627 | 0.246309634 | 0.344833487 |
| ctx-rh-entorhinal_MD | LNS (Years 0-2) | Pearson | -0.118932577 | 0.245931316 | 0.447339515 |
| Olfactory_Right_MD | HVLDR (Years 2-4) | Pearson | -0.117326071 | 0.260083761 | 0.756929623 |
| Left-Thalamus-Proper_MD | HVLDR (Years 2-4) | Pearson | -0.117187687 | 0.260650226 | 0.756929623 |
| ctx-rh-entorhinal_MD | UPDRS 1A (Years 2-4) | Pearson | 0.117053837 | 0.349271458 | 0.565713221 |
| Olfactory_Left_MD | LNS (Years 0-2) | Pearson | -0.116541377 | 0.25562258 | 0.447339515 |
| ctx-lh-medialorbitofrontal_MD | UPDRS 1 (Years 0-2) | Pearson | 0.11646175 | 0.268918891 | 0.787207997 |
| Olfactory_Left_MD | HVLDR (Years 0-2) | Pearson | -0.11561863 | 0.261967125 | 0.529305237 |
| ctx-rh-entorhinal_MD | MoCA (Years 0-2) | Pearson | 0.115344243 | 0.26310869 | 0.810613862 |
| ctx-rh-entorhinal_MD | HVLDR (Years 0-2) | Pearson | -0.114878483 | 0.265054171 | 0.529305237 |
| ctx-rh-medialorbitofrontal_MD | UPDRS 2 (Years 0-2) | Pearson | -0.114178936 | 0.265450633 | 0.960629925 |
| Right-Thalamus-Proper_MD | HVLTR (Years 2-4) | Pearson | -0.114101337 | 0.270896664 | 0.984628911 |
| ctx-rh-medialorbitofrontal_MD | SF (Years 2-4) | Pearson | -0.113886405 | 0.271806267 | 0.845399689 |
| Left-Thalamus-Proper_MD | UPDRS 1A (Years 2-4) | Pearson | 0.113623213 | 0.363672785 | 0.565713221 |
| Olfactory_Left_MD | LNS (Years 2-4) | Pearson | -0.113253515 | 0.274496642 | 0.384295298 |
| ctx-rh-lateralorbitofrontal_MD | UPDRS 1 (Years 0-4) | Pearson | 0.112434674 | 0.285936489 | 0.66718514 |
| ctx-lh-medialorbitofrontal_MD | HVLDR (Years 2-4) | Pearson | -0.112231641 | 0.281491931 | 0.585178219 |
| Left-Amygdala_MD | SF (Years 0-4) | Pearson | -0.112103789 | 0.279429685 | 0.922234768 |
| ctx-lh-lateralorbitofrontal_MD | HVLDR (Years 2-4) | Pearson | -0.111189019 | 0.286014262 | 0.585178219 |
| Left-Thalamus-Proper_MD | UPDRS 1 (Years 0-2) | Pearson | 0.111033475 | 0.292021867 | 0.787207997 |
| Left-Amygdala_MD | SDM (Years 0-2) | Pearson | -0.110363272 | 0.281858992 | 0.493253236 |
| ctx-rh-lateralorbitofrontal_MD | HVLDR (Years 2-4) | Pearson | -0.109692544 | 0.29258911 | 0.585178219 |
| Olfactory_Left_MD | SF (Years 0-2) | Pearson | 0.107023833 | 0.296763845 | 0.957648714 |
| Olfactory_Right_MD | UPDRS 1A (Years 0-4) | Pearson | -0.10692968 | 0.428541683 | 0.995151246 |
| ctx-rh-insula_MD | HVLDR (Years 0-2) | Pearson | -0.106337779 | 0.302460135 | 0.529305237 |
| ctx-lh-entorhinal_MD | UPDRS 2 (Years 0-2) | Pearson | -0.105687502 | 0.302870996 | 0.960629925 |
| Right-Amygdala_MD | UPDRS 1A (Years 0-4) | Pearson | -0.105016345 | 0.436896918 | 0.995151246 |
| Left-Thalamus-Proper_MD | LNS (Years 2-4) | Pearson | -0.104623759 | 0.312965512 | 0.398319743 |
| Right-Amygdala_MD | SDM (Years 2-4) | Pearson | -0.104551438 | 0.313301976 | 0.370998569 |
| ctx-rh-lateralorbitofrontal_MD | UPDRS 1A (Years 0-4) | Pearson | 0.104439768 | 0.439432506 | 0.995151246 |
| Right-Thalamus-Proper_MD | HVLDR (Years 2-4) | Pearson | -0.10404099 | 0.318313464 | 0.756929623 |
| ctx-lh-medialorbitofrontal_MD | UPDRS 1 (Years 2-4) | Pearson | 0.10392715 | 0.318846163 | 0.984258963 |
| Olfactory_Left_MD | SDM (Years 2-4) | Pearson | -0.103547088 | 0.317998774 | 0.370998569 |
| ctx-lh-lateralorbitofrontal_MD | JLO (Years 0-2) | Pearson | 0.103261577 | 0.31416638 | 0.572661372 |
| Right-Amygdala_MD | HVLDR (Years 2-4) | Pearson | -0.102340854 | 0.326328725 | 0.756929623 |
| Right-Amygdala_MD | SF (Years 0-4) | Pearson | -0.101799969 | 0.326276654 | 0.922234768 |
| ctx-rh-lateralorbitofrontal_MD | UPDRS 1B (Years 0-4) | Pearson | 0.10163513 | 0.337750308 | 0.699542686 |
| ctx-rh-insula_MD | UPDRS 1 (Years 0-2) | Pearson | 0.101146863 | 0.337374856 | 0.787207997 |
| ctx-rh-entorhinal_MD | JLO (Years 0-2) | Pearson | 0.100524203 | 0.32723507 | 0.572661372 |
| ctx-lh-lateralorbitofrontal_MD | HVLDR (Years 0-2) | Pearson | -0.099920402 | 0.332735747 | 0.438232938 |
| ctx-rh-insula_MD | UPDRS 1B (Years 0-4) | Pearson | 0.09914689 | 0.349771343 | 0.699542686 |
| ctx-rh-lateralorbitofrontal_MD | SDM (Years 0-2) | Pearson | -0.098908605 | 0.33510886 | 0.521280448 |
| ctx-lh-lateralorbitofrontal_MD | HVLDR (Years 0-4) | Pearson | -0.09860317 | 0.347049332 | 0.419121423 |
| ctx-lh-entorhinal_MD | HVLDR (Years 0-2) | Pearson | -0.097562774 | 0.34432588 | 0.438232938 |
| Olfactory_Left_MD | UPDRS 1B (Years 2-4) | Pearson | 0.097248734 | 0.351099562 | 0.956068694 |
| Right-Thalamus-Proper_MD | LNS (Years 0-2) | Pearson | -0.092443209 | 0.367807656 | 0.525159931 |
| Left-Amygdala_MD | UPDRS 1 (Years 0-4) | Pearson | 0.092019071 | 0.382989585 | 0.765979171 |
| ctx-lh-insula_MD | UPDRS 2 (Years 0-4) | Pearson | 0.091464025 | 0.372924957 | 0.940662961 |
| Right-Thalamus-Proper_MD | SF (Years 0-4) | Pearson | -0.091226088 | 0.379278634 | 0.922234768 |
| ctx-rh-insula_MD | LNS (Years 0-2) | Pearson | -0.091047616 | 0.375114236 | 0.525159931 |
| ctx-rh-insula_MD | HVLDR (Years 0-4) | Pearson | -0.090585596 | 0.387841618 | 0.419121423 |
| Olfactory_Right_MD | HVLDR (Years 0-4) | Pearson | -0.090330645 | 0.389184178 | 0.419121423 |
| Left-Amygdala_MD | SF (Years 0-2) | Pearson | -0.089244989 | 0.384681412 | 0.957648714 |
| ctx-rh-lateralorbitofrontal_MD | UPDRS 1 (Years 0-2) | Pearson | 0.089228866 | 0.397641798 | 0.795283597 |
| ctx-lh-medialorbitofrontal_MD | HVLDR (Years 0-2) | Pearson | -0.089049911 | 0.388250862 | 0.441765598 |
| ctx-lh-entorhinal_MD | MoCA (Years 0-2) | Pearson | 0.088113965 | 0.393276856 | 0.810613862 |
| ctx-rh-medialorbitofrontal_MD | UPDRS 2 (Years 0-4) | Pearson | -0.087926487 | 0.39177127 | 0.940662961 |
| ctx-lh-medialorbitofrontal_MD | MoCA (Years 0-4) | Pearson | -0.087108287 | 0.401253828 | 0.702194198 |
| ctx-rh-medialorbitofrontal_MD | HVLTR (Years 0-2) | Pearson | -0.086714339 | 0.398357583 | 0.50700056 |
| ctx-lh-entorhinal_MD | JLO (Years 0-4) | Pearson | 0.08664318 | 0.406335139 | 0.550582962 |
| ctx-lh-entorhinal_MD | HVLTR (Years 2-4) | Pearson | -0.08642723 | 0.404959491 | 0.984628911 |
| ctx-lh-lateralorbitofrontal_MD | HVLTR (Years 2-4) | Pearson | -0.085698276 | 0.408947975 | 0.984628911 |
| ctx-rh-medialorbitofrontal_MD | HVLDR (Years 0-2) | Pearson | -0.085011727 | 0.410210913 | 0.441765598 |
| ctx-lh-medialorbitofrontal_MD | UPDRS 1B (Years 0-2) | Pearson | 0.083866563 | 0.429311575 | 0.846522392 |
| Olfactory_Left_MD | HVLDR (Years 2-4) | Pearson | -0.083657556 | 0.422758709 | 0.73982774 |
| ctx-lh-entorhinal_MD | HVLDR (Years 0-4) | Pearson | -0.083510285 | 0.423578599 | 0.430365692 |
| Right-Amygdala_MD | GDS (Years 0-2) | Pearson | -0.083483316 | 0.416231298 | 0.98559754 |
| Olfactory_Right_MD | GDS (Years 0-4) | Pearson | 0.083412029 | 0.41663083 | 0.987569874 |
| ctx-lh-entorhinal_MD | HVLTR (Years 0-4) | Pearson | -0.083362519 | 0.421882064 | 0.421882064 |
| ctx-rh-entorhinal_MD | HVLDR (Years 0-4) | Pearson | -0.08229741 | 0.430365692 | 0.430365692 |
| Olfactory_Left_MD | JLO (Years 0-2) | Pearson | 0.081521623 | 0.427306694 | 0.664699302 |
| ctx-rh-lateralorbitofrontal_MD | SF (Years 0-4) | Pearson | -0.081476359 | 0.432496592 | 0.922234768 |
| Left-Thalamus-Proper_MD | SF (Years 2-4) | Pearson | -0.081209762 | 0.434009058 | 0.845399689 |
| ctx-rh-entorhinal_MD | UPDRS 2 (Years 2-4) | Pearson | -0.081182406 | 0.436659517 | 0.913899161 |
| ctx-rh-insula_MD | GDS (Years 2-4) | Pearson | 0.080048221 | 0.435735228 | 0.96312321 |
| Olfactory_Left_MD | JLO (Years 0-4) | Pearson | -0.079406251 | 0.446792228 | 0.550582962 |
| Left-Thalamus-Proper_MD | UPDRS 1B (Years 0-2) | Pearson | 0.078450594 | 0.459806285 | 0.846522392 |
| ctx-rh-lateralorbitofrontal_MD | HVLDR (Years 2-4) | Pearson | -0.078417268 | 0.452490781 | 0.756929623 |
| ctx-rh-entorhinal_MD | SDM (Years 0-2) | Pearson | -0.078243043 | 0.446189136 | 0.604954895 |
| Right-Thalamus-Proper_MD | UPDRS 1A (Years 0-4) | Pearson | 0.077828955 | 0.564985803 | 0.995151246 |
| ctx-rh-lateralorbitofrontal_MD | JLO (Years 0-4) | Pearson | -0.077501251 | 0.457804727 | 0.550582962 |
| ctx-lh-lateralorbitofrontal_MD | UPDRS 1B (Years 0-2) | Pearson | 0.077438667 | 0.465633199 | 0.846522392 |
| ctx-rh-lateralorbitofrontal_MD | HVLDR (Years 0-2) | Pearson | -0.076991837 | 0.455915871 | 0.709202465 |
| ctx-rh-entorhinal_MD | UPDRS 1A (Years 0-2) | Pearson | -0.076966088 | 0.569320326 | 0.992597828 |
| Left-Amygdala_MD | HVLDR (Years 2-4) | Pearson | -0.07642432 | 0.464095971 | 0.756929623 |
| ctx-lh-medialorbitofrontal_MD | UPDRS 2 (Years 2-4) | Pearson | -0.075807846 | 0.467718506 | 0.913899161 |
| ctx-lh-medialorbitofrontal_MD | HVLTR (Years 2-4) | Pearson | -0.075799051 | 0.465348247 | 0.984628911 |
| ctx-rh-medialorbitofrontal_MD | JLO (Years 0-4) | Pearson | 0.0750947 | 0.471928253 | 0.550582962 |
| ctx-rh-lateralorbitofrontal_MD | UPDRS 1B (Years 0-2) | Pearson | 0.074339538 | 0.483727081 | 0.846522392 |
| ctx-lh-lateralorbitofrontal_MD | MoCA (Years 0-4) | Pearson | -0.073969178 | 0.476220294 | 0.740787124 |
| ctx-rh-entorhinal_MD | HVLTR (Years 2-4) | Pearson | -0.073909964 | 0.476574383 | 0.984628911 |
| ctx-rh-medialorbitofrontal_MD | SDM (Years 0-2) | Pearson | -0.073333189 | 0.475321703 | 0.604954895 |
| Olfactory_Right_MD | SF (Years 0-2) | Pearson | 0.073199462 | 0.476129277 | 0.957648714 |
| ctx-lh-lateralorbitofrontal_MD | HVLDR (Years 2-4) | Pearson | -0.072499718 | 0.487418505 | 0.756929623 |
| ctx-rh-lateralorbitofrontal_MD | UPDRS 1A (Years 0-2) | Pearson | 0.072428171 | 0.592366286 | 0.992597828 |
| ctx-rh-entorhinal_MD | LNS (Years 0-4) | Pearson | -0.072373531 | 0.485811347 | 0.485811347 |
| ctx-rh-medialorbitofrontal_MD | SF (Years 0-2) | Pearson | 0.071252807 | 0.487968437 | 0.957648714 |
| Left-Thalamus-Proper_MD | LNS (Years 0-2) | Pearson | -0.071234822 | 0.488078547 | 0.578352754 |
| Left-Thalamus-Proper_MD | UPDRS 1 (Years 0-4) | Pearson | 0.071164843 | 0.500240227 | 0.875420397 |
| ctx-rh-lateralorbitofrontal_MD | SF (Years 0-2) | Pearson | 0.070562356 | 0.49220486 | 0.957648714 |
| Left-Amygdala_MD | LNS (Years 0-2) | Pearson | -0.069990066 | 0.495730932 | 0.578352754 |
| ctx-rh-lateralorbitofrontal_MD | GDS (Years 2-4) | Pearson | 0.069601414 | 0.498133091 | 0.96312321 |
| ctx-lh-insula_MD | MoCA (Years 0-2) | Pearson | -0.069511748 | 0.500968786 | 0.810613862 |
| Right-Thalamus-Proper_MD | UPDRS 2 (Years 0-4) | Pearson | 0.069270394 | 0.500183836 | 0.940662961 |
| ctx-rh-entorhinal_MD | HVLTR (Years 0-2) | Pearson | -0.069041599 | 0.501603848 | 0.58520449 |
| Left-Thalamus-Proper_MD | MoCA (Years 2-4) | Pearson | -0.068622967 | 0.511053801 | 0.852447016 |
| ctx-lh-medialorbitofrontal_MD | JLO (Years 2-4) | Pearson | -0.06791776 | 0.515415636 | 0.655983536 |
| Right-Thalamus-Proper_MD | SF (Years 2-4) | Pearson | -0.067756162 | 0.514134696 | 0.845399689 |
| Olfactory_Right_MD | GDS (Years 0-2) | Pearson | 0.067474609 | 0.511385508 | 0.98559754 |
| Right-Thalamus-Proper_MD | GDS (Years 0-2) | Pearson | -0.066861137 | 0.515241517 | 0.98559754 |
| Olfactory_Right_MD | HVLDR (Years 2-4) | Pearson | -0.066809705 | 0.52230735 | 0.752546034 |
| ctx-rh-insula_MD | UPDRS 1A (Years 0-4) | Pearson | 0.066501939 | 0.623072879 | 0.995151246 |
| ctx-rh-medialorbitofrontal_MD | MoCA (Years 0-2) | Pearson | -0.066332851 | 0.520794877 | 0.810613862 |
| ctx-rh-medialorbitofrontal_MD | UPDRS 1A (Years 0-4) | Pearson | 0.066214648 | 0.624578377 | 0.995151246 |
| Olfactory_Left_MD | HVLDR (Years 2-4) | Pearson | -0.065128256 | 0.532853614 | 0.756929623 |
| ctx-lh-insula_MD | GDS (Years 2-4) | Pearson | 0.064934345 | 0.527448362 | 0.96312321 |
| ctx-lh-medialorbitofrontal_MD | UPDRS 1B (Years 2-4) | Pearson | 0.06490063 | 0.534289412 | 0.956068694 |
| Olfactory_Right_MD | UPDRS 1B (Years 2-4) | Pearson | 0.064680871 | 0.535677408 | 0.956068694 |
| ctx-lh-lateralorbitofrontal_MD | MoCA (Years 0-2) | Pearson | 0.064582816 | 0.531876678 | 0.810613862 |
| ctx-lh-entorhinal_MD | SF (Years 0-2) | Pearson | 0.064447371 | 0.530556315 | 0.957648714 |
| ctx-rh-lateralorbitofrontal_MD | JLO (Years 0-2) | Pearson | 0.064415517 | 0.530759931 | 0.743063903 |
| ctx-rh-entorhinal_MD | SDM (Years 0-4) | Pearson | -0.063891011 | 0.538477057 | 0.579898369 |
| ctx-lh-insula_MD | SF (Years 0-4) | Pearson | -0.063397284 | 0.54162696 | 0.922234768 |
| Left-Thalamus-Proper_MD | HVLTR (Years 2-4) | Pearson | -0.062804014 | 0.545423861 | 0.984628911 |
| Left-Thalamus-Proper_MD | UPDRS 2 (Years 0-2) | Pearson | -0.06276119 | 0.541388017 | 0.960629925 |
| Olfactory_Right_MD | MoCA (Years 2-4) | Pearson | -0.06274177 | 0.548001653 | 0.852447016 |
| Olfactory_Right_MD | LNS (Years 2-4) | Pearson | -0.062424856 | 0.54785724 | 0.63916678 |
| ctx-lh-entorhinal_MD | UPDRS 2 (Years 0-4) | Pearson | -0.061665779 | 0.548482475 | 0.940662961 |
| ctx-rh-medialorbitofrontal_MD | HVLTR (Years 2-4) | Pearson | -0.061641966 | 0.552898355 | 0.984628911 |
| ctx-lh-insula_MD | LNS (Years 0-2) | Pearson | -0.061253785 | 0.551162406 | 0.593559515 |
| ctx-lh-medialorbitofrontal_MD | MoCA (Years 0-2) | Pearson | -0.060987348 | 0.555007124 | 0.810613862 |
| ctx-rh-insula_MD | UPDRS 2 (Years 0-2) | Pearson | -0.060985709 | 0.552909578 | 0.960629925 |
| ctx-lh-medialorbitofrontal_MD | HVLDR (Years 0-2) | Pearson | -0.060223551 | 0.559982355 | 0.737499004 |
| ctx-rh-entorhinal_MD | HVLDR (Years 2-4) | Pearson | -0.060121744 | 0.564869473 | 0.756929623 |
| Right-Amygdala_MD | GDS (Years 0-4) | Pearson | -0.059892062 | 0.560064937 | 0.987569874 |
| Right-Amygdala_MD | MoCA (Years 0-2) | Pearson | -0.059671685 | 0.563590295 | 0.810613862 |
| Olfactory_Right_MD | UPDRS 1A (Years 2-4) | Pearson | 0.059629114 | 0.634361533 | 0.859170802 |
| ctx-lh-medialorbitofrontal_MD | GDS (Years 0-2) | Pearson | -0.058755122 | 0.567550057 | 0.98559754 |
| ctx-rh-entorhinal_MD | SF (Years 0-4) | Pearson | 0.058504855 | 0.573320077 | 0.922234768 |
| ctx-rh-entorhinal_MD | UPDRS 1 (Years 0-2) | Pearson | -0.057790694 | 0.584249831 | 0.822499724 |
| ctx-lh-medialorbitofrontal_MD | HVLTR (Years 0-2) | Pearson | -0.05739924 | 0.576537744 | 0.620886801 |
| Olfactory_Right_MD | MoCA (Years 0-2) | Pearson | 0.057331663 | 0.579009901 | 0.810613862 |
| ctx-rh-medialorbitofrontal_MD | MoCA (Years 0-4) | Pearson | -0.056576821 | 0.586043099 | 0.783360971 |
| Right-Thalamus-Proper_MD | SDM (Years 0-2) | Pearson | -0.055989834 | 0.585949637 | 0.68360791 |
| Left-Thalamus-Proper_MD | HVLDR (Years 2-4) | Pearson | -0.055904782 | 0.59252288 | 0.752546034 |
| ctx-lh-medialorbitofrontal_MD | HVLDR (Years 2-4) | Pearson | -0.055442285 | 0.595592556 | 0.756929623 |
| ctx-rh-medialorbitofrontal_MD | JLO (Years 2-4) | Pearson | -0.055263091 | 0.596783821 | 0.684207818 |
| Right-Thalamus-Proper_MD | GDS (Years 2-4) | Pearson | 0.055020986 | 0.592459927 | 0.96312321 |
| ctx-rh-entorhinal_MD | SF (Years 2-4) | Pearson | 0.05431217 | 0.601149826 | 0.845399689 |
| ctx-lh-insula_MD | SF (Years 2-4) | Pearson | -0.053747629 | 0.604942447 | 0.845399689 |
| ctx-rh-entorhinal_MD | UPDRS 1 (Years 2-4) | Pearson | 0.053697628 | 0.607235854 | 0.984258963 |
| Right-Amygdala_MD | UPDRS 2 (Years 0-4) | Pearson | -0.053641554 | 0.601785088 | 0.940662961 |
| Left-Thalamus-Proper_MD | SF (Years 0-4) | Pearson | -0.053545676 | 0.606301741 | 0.922234768 |
| ctx-lh-lateralorbitofrontal_MD | SF (Years 2-4) | Pearson | -0.053496401 | 0.606633599 | 0.845399689 |
| Right-Thalamus-Proper_MD | UPDRS 1 (Years 2-4) | Pearson | -0.053157233 | 0.610862451 | 0.984258963 |
| ctx-lh-entorhinal_MD | SDM (Years 0-4) | Pearson | -0.052995135 | 0.610014088 | 0.610014088 |
| ctx-rh-entorhinal_MD | HVLDR (Years 2-4) | Pearson | 0.052901285 | 0.612583412 | 0.752546034 |
| Left-Thalamus-Proper_MD | UPDRS 1B (Years 0-4) | Pearson | 0.052765 | 0.619376579 | 0.959825919 |
| Left-Amygdala_MD | JLO (Years 0-2) | Pearson | 0.052757078 | 0.607798335 | 0.773561518 |
| ctx-rh-entorhinal_MD | MoCA (Years 0-4) | Pearson | 0.052184554 | 0.615497906 | 0.783360971 |
| Right-Thalamus-Proper_MD | SF (Years 0-2) | Pearson | -0.051738959 | 0.614752616 | 0.957648714 |
| ctx-lh-lateralorbitofrontal_MD | SF (Years 0-2) | Pearson | 0.051610673 | 0.615631316 | 0.957648714 |
| ctx-rh-lateralorbitofrontal_MD | UPDRS 1 (Years 2-4) | Pearson | 0.051547544 | 0.621720588 | 0.984258963 |
| ctx-lh-insula_MD | HVLDR (Years 2-4) | Pearson | -0.05137801 | 0.622868964 | 0.756929623 |
| ctx-lh-entorhinal_MD | UPDRS 1A (Years 0-2) | Pearson | 0.051120062 | 0.705694655 | 0.992597828 |
| ctx-lh-entorhinal_MD | HVLDR (Years 0-2) | Pearson | -0.050701008 | 0.623727283 | 0.737499004 |
| ctx-lh-entorhinal_MD | SDM (Years 2-4) | Pearson | -0.050635041 | 0.626039613 | 0.674196506 |
| ctx-lh-insula_MD | GDS (Years 0-4) | Pearson | 0.050257019 | 0.624936092 | 0.987569874 |
| Right-Thalamus-Proper_MD | UPDRS 1B (Years 2-4) | Pearson | -0.049977391 | 0.632390679 | 0.956068694 |
| ctx-lh-entorhinal_MD | JLO (Years 2-4) | Pearson | 0.049545964 | 0.635335831 | 0.684207818 |
| ctx-rh-medialorbitofrontal_MD | HVLDR (Years 0-2) | Pearson | -0.049476738 | 0.632142003 | 0.737499004 |
| Olfactory_Right_MD | UPDRS 1 (Years 0-4) | Pearson | 0.049476189 | 0.639528983 | 0.973915922 |
| Olfactory_Right_MD | SF (Years 0-4) | Pearson | -0.049459989 | 0.63408436 | 0.922234768 |
| ctx-lh-lateralorbitofrontal_MD | UPDRS 1 (Years 0-2) | Pearson | 0.049153188 | 0.641719417 | 0.822499724 |
| Olfactory_Left_MD | UPDRS 1 (Years 0-2) | Pearson | -0.0489563 | 0.643056126 | 0.822499724 |
| ctx-lh-medialorbitofrontal_MD | UPDRS 2 (Years 0-4) | Pearson | 0.0484239 | 0.637630768 | 0.940662961 |
| Left-Thalamus-Proper_MD | UPDRS 2 (Years 0-4) | Pearson | 0.048399197 | 0.637802571 | 0.940662961 |
| Right-Thalamus-Proper_MD | HVLDR (Years 2-4) | Pearson | -0.048130326 | 0.645039458 | 0.752546034 |
| Olfactory_Right_MD | HVLTR (Years 2-4) | Pearson | -0.048071743 | 0.643643743 | 0.984628911 |
| ctx-rh-insula_MD | MoCA (Years 0-2) | Pearson | -0.047927728 | 0.642856183 | 0.818180596 |
| ctx-rh-medialorbitofrontal_MD | HVLDR (Years 2-4) | Pearson | -0.047584516 | 0.64879682 | 0.756929623 |
| ctx-rh-medialorbitofrontal_MD | SF (Years 0-4) | Pearson | -0.047109971 | 0.650300751 | 0.922234768 |
| ctx-rh-entorhinal_MD | UPDRS 2 (Years 0-4) | Pearson | -0.046818531 | 0.648834982 | 0.940662961 |
| ctx-rh-entorhinal_MD | GDS (Years 2-4) | Pearson | -0.04647497 | 0.651243075 | 0.96312321 |
| ctx-lh-entorhinal_MD | UPDRS 1B (Years 0-2) | Pearson | -0.046354017 | 0.662611077 | 0.933181033 |
| Olfactory_Left_MD | SF (Years 0-4) | Pearson | -0.04589651 | 0.65873912 | 0.922234768 |
| ctx-rh-medialorbitofrontal_MD | UPDRS 1B (Years 0-2) | Pearson | -0.0457773 | 0.666557881 | 0.933181033 |
| ctx-rh-entorhinal_MD | MoCA (Years 2-4) | Pearson | -0.045690878 | 0.661900453 | 0.859248263 |
| Olfactory_Left_MD | UPDRS 1A (Years 2-4) | Pearson | 0.04560103 | 0.716175084 | 0.859170802 |
| Olfactory_Left_MD | UPDRS 1A (Years 0-2) | Pearson | -0.045490602 | 0.736862109 | 0.992597828 |
| ctx-lh-insula_MD | UPDRS 1B (Years 2-4) | Pearson | 0.04548851 | 0.663306936 | 0.956068694 |
| Right-Thalamus-Proper_MD | GDS (Years 0-4) | Pearson | -0.045449767 | 0.65845017 | 0.987569874 |
| ctx-lh-entorhinal_MD | SF (Years 2-4) | Pearson | -0.045288184 | 0.662985635 | 0.845399689 |
| Right-Amygdala_MD | SF (Years 2-4) | Pearson | 0.045108411 | 0.664242613 | 0.845399689 |
| Left-Amygdala_MD | UPDRS 1B (Years 2-4) | Pearson | -0.045037351 | 0.666446746 | 0.956068694 |
| Olfactory_Left_MD | UPDRS 2 (Years 0-2) | Pearson | -0.044208605 | 0.667217354 | 0.960629925 |
| ctx-lh-entorhinal_MD | MoCA (Years 2-4) | Pearson | -0.043794826 | 0.675123635 | 0.859248263 |
| Olfactory_Right_MD | UPDRS 2 (Years 0-2) | Pearson | -0.043295125 | 0.673698658 | 0.960629925 |
| Left-Amygdala_MD | GDS (Years 0-2) | Pearson | -0.04324595 | 0.674048251 | 0.98559754 |
| ctx-lh-insula_MD | UPDRS 2 (Years 0-2) | Pearson | -0.043128642 | 0.674882478 | 0.960629925 |
| ctx-lh-insula_MD | UPDRS 1A (Years 0-4) | Pearson | 0.041813558 | 0.757453399 | 0.995151246 |
| Left-Amygdala_MD | UPDRS 1A (Years 0-4) | Pearson | -0.041148079 | 0.761198482 | 0.995151246 |
| Left-Amygdala_MD | HVLTR (Years 2-4) | Pearson | -0.039955808 | 0.700653016 | 0.984628911 |
| ctx-rh-medialorbitofrontal_MD | UPDRS 1 (Years 0-2) | Pearson | -0.039203217 | 0.710618553 | 0.822499724 |
| Olfactory_Right_MD | UPDRS 1B (Years 0-4) | Pearson | 0.03891304 | 0.714205404 | 0.959825919 |
| ctx-rh-lateralorbitofrontal_MD | GDS (Years 0-2) | Pearson | -0.038874079 | 0.70539724 | 0.98559754 |
| Olfactory_Right_MD | HVLDR (Years 0-2) | Pearson | -0.038505835 | 0.709539505 | 0.764119467 |
| ctx-rh-medialorbitofrontal_MD | UPDRS 1A (Years 2-4) | Pearson | 0.03832598 | 0.759966387 | 0.859170802 |
| ctx-lh-medialorbitofrontal_MD | GDS (Years 2-4) | Pearson | 0.03829534 | 0.70958569 | 0.96312321 |
| ctx-lh-medialorbitofrontal_MD | GDS (Years 0-4) | Pearson | -0.038188841 | 0.710357392 | 0.987569874 |
| ctx-lh-medialorbitofrontal_MD | JLO (Years 0-4) | Pearson | 0.03782394 | 0.717397159 | 0.772581556 |
| Right-Thalamus-Proper_MD | UPDRS 1 (Years 0-2) | Pearson | 0.037758999 | 0.720831882 | 0.822499724 |
| Left-Amygdala_MD | UPDRS 1 (Years 2-4) | Pearson | -0.037741818 | 0.71798491 | 0.984258963 |
| Olfactory_Right_MD | JLO (Years 0-2) | Pearson | 0.037660791 | 0.714187991 | 0.825450281 |
| Left-Thalamus-Proper_MD | UPDRS 2 (Years 2-4) | Pearson | 0.037559794 | 0.719288249 | 0.913899161 |
| ctx-rh-insula_MD | GDS (Years 0-4) | Pearson | 0.03731965 | 0.71666649 | 0.987569874 |
| Left-Amygdala_MD | UPDRS 2 (Years 2-4) | Pearson | -0.03709033 | 0.722653445 | 0.913899161 |
| Right-Amygdala_MD | UPDRS 1 (Years 2-4) | Pearson | 0.036911033 | 0.72394007 | 0.984258963 |
| Left-Amygdala_MD | UPDRS 2 (Years 0-2) | Pearson | -0.036380724 | 0.723503208 | 0.960629925 |
| ctx-rh-lateralorbitofrontal_MD | UPDRS 1B (Years 2-4) | Pearson | 0.03599263 | 0.73054246 | 0.956068694 |
| ctx-lh-insula_MD | UPDRS 1A (Years 0-2) | Pearson | 0.035391038 | 0.793815902 | 0.992597828 |
| ctx-lh-lateralorbitofrontal_MD | GDS (Years 0-4) | Pearson | -0.034991715 | 0.733656961 | 0.987569874 |
| ctx-lh-medialorbitofrontal_MD | MoCA (Years 2-4) | Pearson | -0.033593298 | 0.747883028 | 0.872530199 |
| Olfactory_Left_MD | UPDRS 2 (Years 0-4) | Pearson | -0.0331069 | 0.747508434 | 0.940662961 |
| Olfactory_Right_MD | UPDRS 2 (Years 2-4) | Pearson | 0.032685773 | 0.75447532 | 0.913899161 |
| Olfactory_Left_MD | GDS (Years 2-4) | Pearson | 0.032416437 | 0.75260305 | 0.96312321 |
| ctx-lh-entorhinal_MD | UPDRS 1 (Years 0-2) | Pearson | -0.03176195 | 0.763749744 | 0.822499724 |
| Left-Amygdala_MD | SF (Years 2-4) | Pearson | -0.031402993 | 0.762575 | 0.889670833 |
| Olfactory_Right_MD | GDS (Years 2-4) | Pearson | 0.031046043 | 0.762745849 | 0.96312321 |
| ctx-lh-entorhinal_MD | MoCA (Years 0-4) | Pearson | 0.030976715 | 0.765704498 | 0.893321915 |
| ctx-lh-entorhinal_MD | JLO (Years 0-2) | Pearson | 0.030541613 | 0.766489547 | 0.825450281 |
| ctx-rh-medialorbitofrontal_MD | UPDRS 1B (Years 0-4) | Pearson | -0.029671821 | 0.780091542 | 0.959825919 |
| ctx-lh-insula_MD | SF (Years 0-2) | Pearson | -0.029582945 | 0.773619179 | 0.986306573 |
| ctx-rh-entorhinal_MD | UPDRS 1 (Years 0-4) | Pearson | -0.02889392 | 0.784539165 | 0.973915922 |
| Left-Thalamus-Proper_MD | GDS (Years 0-2) | Pearson | -0.028672788 | 0.780405473 | 0.98559754 |
| ctx-lh-entorhinal_MD | LNS (Years 2-4) | Pearson | 0.028455254 | 0.784291093 | 0.791585645 |
| ctx-rh-entorhinal_MD | UPDRS 2 (Years 0-2) | Pearson | -0.028213253 | 0.783838148 | 0.960629925 |
| ctx-rh-lateralorbitofrontal_MD | UPDRS 2 (Years 0-2) | Pearson | -0.028144617 | 0.784351212 | 0.960629925 |
| Left-Amygdala_MD | UPDRS 2 (Years 0-4) | Pearson | -0.02807329 | 0.784884483 | 0.940662961 |
| Left-Amygdala_MD | UPDRS 1A (Years 2-4) | Pearson | -0.027513937 | 0.826420283 | 0.859170802 |
| ctx-lh-lateralorbitofrontal_MD | HVLDR (Years 0-2) | Pearson | -0.027510007 | 0.79019192 | 0.79019192 |
| ctx-rh-entorhinal_MD | LNS (Years 2-4) | Pearson | 0.027470238 | 0.791585645 | 0.791585645 |
| ctx-rh-entorhinal_MD | UPDRS 1A (Years 0-4) | Pearson | 0.02728529 | 0.84032871 | 0.995151246 |
| Olfactory_Right_MD | UPDRS 1 (Years 2-4) | Pearson | 0.02694153 | 0.796592402 | 0.984258963 |
| Right-Amygdala_MD | UPDRS 1B (Years 2-4) | Pearson | -0.026640163 | 0.798819385 | 0.956068694 |
| Right-Amygdala_MD | GDS (Years 2-4) | Pearson | -0.02640751 | 0.797366407 | 0.96312321 |
| Olfactory_Left_MD | MoCA (Years 0-2) | Pearson | -0.026023692 | 0.801287194 | 0.915125839 |
| Right-Thalamus-Proper_MD | UPDRS 2 (Years 2-4) | Pearson | 0.025532288 | 0.807019865 | 0.913899161 |
| Right-Thalamus-Proper_MD | UPDRS 1A (Years 0-2) | Pearson | 0.024934317 | 0.85392879 | 0.992597828 |
| ctx-rh-insula_MD | SF (Years 0-4) | Pearson | -0.024129827 | 0.816453543 | 0.990833022 |
| ctx-lh-lateralorbitofrontal_MD | GDS (Years 0-2) | Pearson | -0.023940638 | 0.815944191 | 0.98559754 |
| ctx-rh-insula_MD | UPDRS 2 (Years 0-4) | Pearson | 0.023924011 | 0.816069763 | 0.940662961 |
| Olfactory_Left_MD | UPDRS 1B (Years 0-4) | Pearson | 0.023656367 | 0.823863262 | 0.959825919 |
| Olfactory_Left_MD | UPDRS 1B (Years 0-2) | Pearson | -0.02347448 | 0.825195935 | 0.987502283 |
| ctx-lh-entorhinal_MD | UPDRS 1A (Years 2-4) | Pearson | 0.022262764 | 0.859170802 | 0.859170802 |
| ctx-rh-medialorbitofrontal_MD | GDS (Years 2-4) | Pearson | -0.02187836 | 0.831554 | 0.96312321 |
| ctx-rh-entorhinal_MD | GDS (Years 0-4) | Pearson | -0.02184318 | 0.831820868 | 0.987569874 |
| ctx-rh-entorhinal_MD | JLO (Years 2-4) | Pearson | -0.021643523 | 0.835964325 | 0.835964325 |
| ctx-rh-entorhinal_MD | UPDRS 1B (Years 0-4) | Pearson | 0.021582959 | 0.83908363 | 0.959825919 |
| ctx-lh-entorhinal_MD | UPDRS 1 (Years 2-4) | Pearson | 0.021333285 | 0.838283438 | 0.984258963 |
| Left-Thalamus-Proper_MD | UPDRS 1B (Years 2-4) | Pearson | -0.020801659 | 0.842260699 | 0.956068694 |
| Olfactory_Left_MD | MoCA (Years 2-4) | Pearson | 0.020468483 | 0.84475531 | 0.909736488 |
| ctx-rh-entorhinal_MD | SDM (Years 2-4) | Pearson | -0.020221438 | 0.845780567 | 0.845780567 |
| Left-Thalamus-Proper_MD | UPDRS 1A (Years 0-2) | Pearson | -0.02020064 | 0.881436998 | 0.992597828 |
| ctx-lh-insula_MD | UPDRS 1 (Years 2-4) | Pearson | 0.020163335 | 0.847041421 | 0.984258963 |
| ctx-rh-lateralorbitofrontal_MD | MoCA (Years 0-2) | Pearson | -0.019587849 | 0.849759708 | 0.915125839 |
| Left-Amygdala_MD | GDS (Years 2-4) | Pearson | 0.019192524 | 0.8519812 | 0.96312321 |
| Left-Thalamus-Proper_MD | GDS (Years 2-4) | Pearson | 0.019090703 | 0.852757618 | 0.96312321 |
| ctx-lh-entorhinal_MD | SDM (Years 0-2) | Pearson | -0.018710507 | 0.85565797 | 0.860750908 |
| Right-Amygdala_MD | HVLTR (Years 2-4) | Pearson | -0.018556579 | 0.858339626 | 0.984628911 |
| ctx-rh-entorhinal_MD | HVLDR (Years 0-4) | Pearson | -0.018432309 | 0.860792408 | 0.860792408 |
| Left-Amygdala_MD | UPDRS 1A (Years 0-2) | Pearson | -0.018373688 | 0.892092881 | 0.992597828 |
| Left-Thalamus-Proper_MD | SDM (Years 0-2) | Pearson | 0.018043498 | 0.860750908 | 0.860750908 |
| Olfactory_Left_MD | UPDRS 2 (Years 2-4) | Pearson | 0.018024275 | 0.863101341 | 0.913899161 |
| ctx-rh-insula_MD | HVLTR (Years 2-4) | Pearson | 0.016709315 | 0.872315946 | 0.984628911 |
| ctx-rh-lateralorbitofrontal_MD | HVLTR (Years 2-4) | Pearson | -0.016327534 | 0.875209527 | 0.984628911 |
| ctx-lh-entorhinal_MD | HVLDR (Years 2-4) | Pearson | 0.016019956 | 0.87820043 | 0.935631415 |
| Right-Amygdala_MD | JLO (Years 0-2) | Pearson | -0.015899766 | 0.87715697 | 0.87715697 |
| ctx-rh-insula_MD | SF (Years 0-2) | Pearson | -0.015838181 | 0.877629085 | 0.986306573 |
| ctx-lh-entorhinal_MD | UPDRS 2 (Years 2-4) | Pearson | 0.015775638 | 0.880044039 | 0.913899161 |
| ctx-rh-insula_MD | HVLDR (Years 2-4) | Pearson | 0.015383316 | 0.883005818 | 0.950929343 |
| ctx-lh-lateralorbitofrontal_MD | UPDRS 2 (Years 0-4) | Pearson | 0.015322953 | 0.881580523 | 0.940662961 |
| ctx-lh-medialorbitofrontal_MD | SF (Years 2-4) | Pearson | -0.015061435 | 0.88481705 | 0.929330034 |
| ctx-rh-insula_MD | UPDRS 1A (Years 0-2) | Pearson | 0.01500388 | 0.911796044 | 0.992597828 |
| ctx-rh-entorhinal_MD | UPDRS 1B (Years 0-2) | Pearson | 0.014895275 | 0.88855304 | 0.987502283 |
| ctx-rh-insula_MD | UPDRS 1B (Years 2-4) | Pearson | -0.014674266 | 0.888362737 | 0.956068694 |
| Right-Thalamus-Proper_MD | UPDRS 1B (Years 0-4) | Pearson | -0.014530297 | 0.891266925 | 0.959825919 |
| ctx-lh-entorhinal_MD | GDS (Years 0-4) | Pearson | 0.014368359 | 0.888909189 | 0.987569874 |
| ctx-rh-medialorbitofrontal_MD | GDS (Years 0-2) | Pearson | -0.013946606 | 0.892150113 | 0.98559754 |
| ctx-rh-lateralorbitofrontal_MD | UPDRS 2 (Years 2-4) | Pearson | -0.013892247 | 0.894276754 | 0.913899161 |
| Right-Amygdala_MD | LNS (Years 0-2) | Pearson | 0.013892078 | 0.892569256 | 0.892569256 |
| ctx-lh-lateralorbitofrontal_MD | UPDRS 2 (Years 0-2) | Pearson | -0.013740175 | 0.893737064 | 0.960629925 |
| ctx-rh-insula_MD | UPDRS 1 (Years 2-4) | Pearson | 0.013699353 | 0.895736422 | 0.984258963 |
| ctx-lh-entorhinal_MD | GDS (Years 0-2) | Pearson | 0.013438128 | 0.896059819 | 0.98559754 |
| Right-Thalamus-Proper_MD | UPDRS 1 (Years 0-4) | Pearson | 0.013360441 | 0.899413455 | 0.973915922 |
| Olfactory_Right_MD | UPDRS 2 (Years 0-4) | Pearson | -0.013161612 | 0.898187009 | 0.940662961 |
| ctx-lh-entorhinal_MD | UPDRS 1B (Years 2-4) | Pearson | 0.012905697 | 0.901745755 | 0.956068694 |
| ctx-lh-lateralorbitofrontal_MD | UPDRS 2 (Years 2-4) | Pearson | -0.012817823 | 0.902411457 | 0.913899161 |
| Right-Thalamus-Proper_MD | UPDRS 2 (Years 0-2) | Pearson | -0.012787692 | 0.901064656 | 0.960629925 |
| ctx-rh-medialorbitofrontal_MD | GDS (Years 0-4) | Pearson | -0.012411675 | 0.903959737 | 0.987569874 |
| ctx-rh-lateralorbitofrontal_MD | GDS (Years 0-4) | Pearson | -0.012255162 | 0.905165152 | 0.987569874 |
| ctx-rh-medialorbitofrontal_MD | UPDRS 2 (Years 2-4) | Pearson | -0.011302777 | 0.913899161 | 0.913899161 |
| Olfactory_Left_MD | HVLTR (Years 2-4) | Pearson | 0.011189213 | 0.914298274 | 0.984628911 |
| Right-Thalamus-Proper_MD | UPDRS 1B (Years 0-2) | Pearson | 0.01108183 | 0.916966406 | 0.987502283 |
| ctx-lh-entorhinal_MD | GDS (Years 2-4) | Pearson | -0.010841189 | 0.916064571 | 0.96312321 |
| ctx-lh-medialorbitofrontal_MD | SF (Years 0-4) | Pearson | -0.010311218 | 0.921000531 | 0.990833022 |
| Right-Amygdala_MD | UPDRS 2 (Years 0-2) | Pearson | -0.010189894 | 0.921090343 | 0.960629925 |
| ctx-lh-entorhinal_MD | SF (Years 0-4) | Pearson | 0.009937641 | 0.923853965 | 0.990833022 |
| Olfactory_Left_MD | UPDRS 1 (Years 0-4) | Pearson | 0.00990249 | 0.9253594 | 0.973915922 |
| ctx-rh-entorhinal_MD | HVLDR (Years 0-2) | Pearson | -0.009899295 | 0.923739255 | 0.923739255 |
| Left-Amygdala_MD | MoCA (Years 0-2) | Pearson | -0.009808515 | 0.92443652 | 0.92443652 |
| ctx-rh-insula_MD | SF (Years 2-4) | Pearson | -0.009221054 | 0.929330034 | 0.929330034 |
| ctx-lh-lateralorbitofrontal_MD | JLO (Years 0-4) | Pearson | 0.008599165 | 0.934441561 | 0.934441561 |
| ctx-rh-insula_MD | HVLDR (Years 2-4) | Pearson | 0.008442757 | 0.935631415 | 0.935631415 |
| Olfactory_Right_MD | UPDRS 1 (Years 0-2) | Pearson | 0.007920669 | 0.940266583 | 0.940266583 |
| ctx-lh-lateralorbitofrontal_MD | UPDRS 1A (Years 0-4) | Pearson | -0.007762798 | 0.954298063 | 0.995151246 |
| ctx-rh-medialorbitofrontal_MD | UPDRS 1B (Years 2-4) | Pearson | 0.007719481 | 0.941135442 | 0.956068694 |
| Left-Thalamus-Proper_MD | UPDRS 1 (Years 2-4) | Pearson | -0.007695626 | 0.941317023 | 0.984258963 |
| ctx-rh-lateralorbitofrontal_MD | UPDRS 2 (Years 0-4) | Pearson | -0.007657049 | 0.940662961 | 0.940662961 |
| ctx-lh-lateralorbitofrontal_MD | UPDRS 1B (Years 2-4) | Pearson | -0.007574167 | 0.942241602 | 0.956068694 |
| ctx-lh-lateralorbitofrontal_MD | UPDRS 1 (Years 2-4) | Pearson | 0.007454324 | 0.943153958 | 0.984258963 |
| Left-Thalamus-Proper_MD | GDS (Years 0-4) | Pearson | -0.007445206 | 0.942301739 | 0.987569874 |
| Left-Thalamus-Proper_MD | SF (Years 0-2) | Pearson | 0.007243888 | 0.943859329 | 0.986306573 |
| Olfactory_Right_MD | UPDRS 1A (Years 0-2) | Pearson | 0.006319688 | 0.962787408 | 0.992597828 |
| ctx-lh-insula_MD | GDS (Years 0-2) | Pearson | 0.006074261 | 0.952912663 | 0.98559754 |
| ctx-lh-insula_MD | HVLDR (Years 2-4) | Pearson | 0.005976933 | 0.954406855 | 0.954406855 |
| ctx-rh-entorhinal_MD | UPDRS 1B (Years 2-4) | Pearson | -0.005758857 | 0.956068694 | 0.956068694 |
| ctx-rh-entorhinal_MD | GDS (Years 0-2) | Pearson | 0.005658135 | 0.956135131 | 0.98559754 |
| ctx-lh-medialorbitofrontal_MD | UPDRS 2 (Years 0-2) | Pearson | 0.005077861 | 0.960629925 | 0.960629925 |
| Olfactory_Left_MD | MoCA (Years 0-4) | Pearson | -0.005075406 | 0.961067233 | 0.992235033 |
| ctx-lh-medialorbitofrontal_MD | SF (Years 0-2) | Pearson | -0.004825225 | 0.962587234 | 0.986306573 |
| ctx-lh-lateralorbitofrontal_MD | GDS (Years 2-4) | Pearson | -0.004756049 | 0.96312321 | 0.96312321 |
| ctx-rh-medialorbitofrontal_MD | MoCA (Years 2-4) | Pearson | 0.003956704 | 0.969808445 | 0.969808445 |
| ctx-lh-entorhinal_MD | HVLTR (Years 0-2) | Pearson | 0.00371276 | 0.971208576 | 0.971208576 |
| ctx-lh-entorhinal_MD | UPDRS 1 (Years 0-4) | Pearson | 0.003587095 | 0.972928279 | 0.973915922 |
| ctx-rh-medialorbitofrontal_MD | UPDRS 1 (Years 0-4) | Pearson | -0.003456182 | 0.973915922 | 0.973915922 |
| ctx-lh-entorhinal_MD | UPDRS 1A (Years 0-4) | Pearson | 0.002456402 | 0.985531494 | 0.995151246 |
| ctx-rh-medialorbitofrontal_MD | UPDRS 1 (Years 2-4) | Pearson | 0.002062563 | 0.984258963 | 0.984258963 |
| ctx-lh-entorhinal_MD | UPDRS 1B (Years 0-4) | Pearson | 0.002054374 | 0.984580579 | 0.984580579 |
| ctx-rh-insula_MD | GDS (Years 0-2) | Pearson | 0.001856953 | 0.98559754 | 0.98559754 |
| ctx-rh-entorhinal_MD | SF (Years 0-2) | Pearson | 0.001765526 | 0.986306573 | 0.986306573 |
| Left-Amygdala_MD | GDS (Years 0-4) | Pearson | 0.001602632 | 0.987569874 | 0.987569874 |
| ctx-lh-lateralorbitofrontal_MD | UPDRS 1A (Years 0-2) | Pearson | 0.00125666 | 0.992597828 | 0.992597828 |
| Olfactory_Right_MD | UPDRS 1B (Years 0-2) | Pearson | 0.001199018 | 0.991000223 | 0.991000223 |
| ctx-lh-lateralorbitofrontal_MD | SF (Years 0-4) | Pearson | -0.001194597 | 0.990833022 | 0.990833022 |
| Olfactory_Right_MD | MoCA (Years 0-4) | Pearson | -0.001011888 | 0.992235033 | 0.992235033 |
| Left-Thalamus-Proper_MD | UPDRS 1A (Years 0-4) | Pearson | -0.000823162 | 0.995151246 | 0.995151246 |
| ctx-lh-insula_MD | HVLTR (Years 2-4) | Pearson | -0.000692947 | 0.994682456 | 0.994682456 |
| lh_lateralorbitofrontal_thickness | SF (Years 0-2) | Pearson | 0.375694295 | 0.000149384 | 0.001195068 |
| rh_entorhinal_thickness | HVLTR (Years 2-4) | Pearson | -0.344636409 | 0.00062612 | 0.005008962 |
| lh_medialorbitofrontal_thickness | UPDRS 1B (Years 0-2) | Pearson | -0.324729198 | 0.001686788 | 0.013494301 |
| lh_medialorbitofrontal_thickness | UPDRS 1 (Years 0-2) | Pearson | -0.28187803 | 0.006485967 | 0.051887736 |
| lh_medialorbitofrontal_thickness | UPDRS 1B (Years 0-4) | Pearson | -0.261160446 | 0.012404314 | 0.099234514 |
| lh_medialorbitofrontal_thickness | SF (Years 0-2) | Pearson | 0.259304311 | 0.010324341 | 0.041297366 |
| rh_insula_thickness | JLO (Years 2-4) | Pearson | 0.250305228 | 0.014966196 | 0.119729568 |
| rh_medialorbitofrontal_thickness | SF (Years 0-2) | Pearson | 0.230275099 | 0.023260276 | 0.062027402 |
| lh_entorhinal_thickness | HVLTR (Years 2-4) | Pearson | -0.230100782 | 0.024880911 | 0.099523644 |
| lh_insula_thickness | HVLDR (Years 0-4) | Pearson | 0.225550416 | 0.029719737 | 0.237757893 |
| lh_entorhinal_thickness | UPDRS 2 (Years 0-2) | Pearson | 0.221953449 | 0.02889029 | 0.231122323 |
| lh_medialorbitofrontal_thickness | UPDRS 1 (Years 0-4) | Pearson | -0.217495503 | 0.037285765 | 0.298286117 |
| rh_insula_thickness | SDM (Years 0-2) | Pearson | 0.212688104 | 0.036476654 | 0.208403087 |
| lh_medialorbitofrontal_thickness | GDS (Years 2-4) | Pearson | 0.204817773 | 0.044171565 | 0.35337252 |
| lh_entorhinal_thickness | SDM (Years 0-2) | Pearson | -0.19782163 | 0.052100772 | 0.208403087 |
| lh_medialorbitofrontal_thickness | LNS (Years 0-2) | Pearson | -0.193597266 | 0.057431647 | 0.315891246 |
| lh_entorhinal_thickness | HVLDR (Years 2-4) | Pearson | 0.191999792 | 0.06375513 | 0.22550935 |
| rh_insula_thickness | LNS (Years 2-4) | Pearson | 0.189798073 | 0.065443522 | 0.523548176 |
| rh_medialorbitofrontal_thickness | UPDRS 1A (Years 0-2) | Pearson | 0.182272574 | 0.174768701 | 0.71805949 |
| lh_lateralorbitofrontal_thickness | UPDRS 1A (Years 0-2) | Pearson | 0.180319032 | 0.179514872 | 0.71805949 |
| rh_insula_thickness | HVLDR (Years 2-4) | Pearson | 0.179766006 | 0.082966024 | 0.22550935 |
| lh_lateralorbitofrontal_thickness | LNS (Years 0-2) | Pearson | -0.179239697 | 0.078972811 | 0.315891246 |
| rh_lateralorbitofrontal_thickness | HVLDR (Years 2-4) | Pearson | 0.178972089 | 0.084357013 | 0.31532298 |
| lh_lateralorbitofrontal_thickness | HVLDR (Years 2-4) | Pearson | 0.178926744 | 0.084437017 | 0.31532298 |
| lh_lateralorbitofrontal_thickness | SF (Years 2-4) | Pearson | -0.178869668 | 0.082857321 | 0.247014901 |
| lh_insula_thickness | HVLDR (Years 2-4) | Pearson | 0.178853707 | 0.084566006 | 0.22550935 |
| rh_entorhinal_thickness | SF (Years 2-4) | Pearson | -0.176960925 | 0.086247066 | 0.247014901 |
| rh_insula_thickness | SDM (Years 0-4) | Pearson | 0.176885797 | 0.086382706 | 0.691061649 |
| rh_lateralorbitofrontal_thickness | SF (Years 0-2) | Pearson | 0.176716301 | 0.083354335 | 0.16670867 |
| lh_lateralorbitofrontal_thickness | HVLTR (Years 0-4) | Pearson | -0.176609689 | 0.086882667 | 0.458834048 |
| lh_medialorbitofrontal_thickness | SF (Years 2-4) | Pearson | -0.173522512 | 0.092630588 | 0.247014901 |
| lh_lateralorbitofrontal_thickness | HVLTR (Years 0-2) | Pearson | -0.170468898 | 0.095037748 | 0.308039424 |
| lh_medialorbitofrontal_thickness | HVLTR (Years 0-2) | Pearson | -0.168477311 | 0.099022152 | 0.308039424 |
| lh_entorhinal_thickness | UPDRS 1B (Years 0-2) | Pearson | 0.166628551 | 0.114423397 | 0.457693588 |
| rh_medialorbitofrontal_thickness | UPDRS 1 (Years 2-4) | Pearson | 0.165798122 | 0.110257038 | 0.822266228 |
| lh_insula_thickness | LNS (Years 0-4) | Pearson | -0.164161805 | 0.111908212 | 0.45746391 |
| lh_entorhinal_thickness | HVLTR (Years 0-4) | Pearson | -0.162909094 | 0.114708512 | 0.458834048 |
| lh_medialorbitofrontal_thickness | HVLDR (Years 2-4) | Pearson | 0.16222761 | 0.118246117 | 0.31532298 |
| rh_entorhinal_thickness | HVLTR (Years 0-2) | Pearson | 0.160846246 | 0.115514784 | 0.308039424 |
| rh_medialorbitofrontal_thickness | GDS (Years 2-4) | Pearson | 0.159630656 | 0.118327659 | 0.473310635 |
| lh_insula_thickness | LNS (Years 0-2) | Pearson | -0.157634804 | 0.123060338 | 0.328160902 |
| rh_insula_thickness | JLO (Years 0-4) | Pearson | 0.155393602 | 0.13477373 | 0.588880132 |
| lh_insula_thickness | SF (Years 0-2) | Pearson | 0.153644265 | 0.132957558 | 0.212732092 |
| rh_insula_thickness | HVLDR (Years 0-4) | Pearson | 0.149569046 | 0.15244552 | 0.609782082 |
| rh_entorhinal_thickness | UPDRS 2 (Years 2-4) | Pearson | 0.149273301 | 0.151015726 | 0.957096231 |
| lh_medialorbitofrontal_thickness | LNS (Years 0-4) | Pearson | -0.146452482 | 0.156718759 | 0.45746391 |
| lh_lateralorbitofrontal_thickness | UPDRS 1 (Years 0-2) | Pearson | -0.14537296 | 0.166768867 | 0.655466985 |
| rh_insula_thickness | UPDRS 2 (Years 0-2) | Pearson | 0.144221797 | 0.158721016 | 0.634884063 |
| lh_insula_thickness | JLO (Years 0-4) | Pearson | -0.144029934 | 0.166061359 | 0.588880132 |
| rh_entorhinal_thickness | MoCA (Years 0-4) | Pearson | 0.143186804 | 0.166272348 | 0.662699261 |
| rh_entorhinal_thickness | HVLDR (Years 2-4) | Pearson | 0.142366217 | 0.171059672 | 0.342119345 |
| lh_medialorbitofrontal_thickness | HVLDR (Years 0-2) | Pearson | -0.140489601 | 0.172170912 | 0.786292394 |
| lh_lateralorbitofrontal_thickness | LNS (Years 0-4) | Pearson | -0.140185543 | 0.175426225 | 0.45746391 |
| lh_medialorbitofrontal_thickness | HVLTR (Years 0-4) | Pearson | -0.137572378 | 0.183693074 | 0.489848198 |
| lh_entorhinal_thickness | SDM (Years 0-4) | Pearson | -0.136630332 | 0.186741815 | 0.746967259 |
| lh_insula_thickness | SF (Years 2-4) | Pearson | -0.135703991 | 0.189775419 | 0.379550839 |
| rh_insula_thickness | SF (Years 0-2) | Pearson | 0.134441503 | 0.189216895 | 0.252289193 |
| rh_medialorbitofrontal_thickness | GDS (Years 0-4) | Pearson | 0.134366823 | 0.189465049 | 0.988878513 |
| rh_insula_thickness | HVLDR (Years 0-2) | Pearson | 0.132956237 | 0.196573098 | 0.786292394 |
| lh_entorhinal_thickness | UPDRS 1B (Years 0-4) | Pearson | 0.132643204 | 0.210063686 | 0.840254745 |
| lh_entorhinal_thickness | UPDRS 1 (Years 2-4) | Pearson | -0.13175539 | 0.205566557 | 0.822266228 |
| rh_entorhinal_thickness | MoCA (Years 2-4) | Pearson | 0.131636556 | 0.205979288 | 0.870805697 |
| rh_entorhinal_thickness | GDS (Years 2-4) | Pearson | -0.130939013 | 0.201110672 | 0.536295125 |
| rh_medialorbitofrontal_thickness | MoCA (Years 2-4) | Pearson | -0.128330112 | 0.217701424 | 0.870805697 |
| lh_lateralorbitofrontal_thickness | SF (Years 0-4) | Pearson | 0.127754995 | 0.217284842 | 0.539030984 |
| rh_entorhinal_thickness | JLO (Years 0-4) | Pearson | -0.127469044 | 0.22083005 | 0.588880132 |
| rh_medialorbitofrontal_thickness | UPDRS 1B (Years 2-4) | Pearson | 0.127172196 | 0.221915938 | 0.968631503 |
| rh_insula_thickness | UPDRS 1A (Years 0-4) | Pearson | -0.126436505 | 0.348658334 | 0.824705378 |
| rh_entorhinal_thickness | SF (Years 0-4) | Pearson | -0.126371331 | 0.222347869 | 0.539030984 |
| rh_insula_thickness | HVLDR (Years 0-4) | Pearson | 0.126258977 | 0.225280124 | 0.88471269 |
| rh_entorhinal_thickness | LNS (Years 0-4) | Pearson | -0.124657908 | 0.228731955 | 0.45746391 |
| rh_medialorbitofrontal_thickness | HVLDR (Years 0-2) | Pearson | 0.124514499 | 0.226771583 | 0.830434844 |
| lh_entorhinal_thickness | HVLDR (Years 2-4) | Pearson | -0.12303136 | 0.237456752 | 0.397896248 |
| lh_medialorbitofrontal_thickness | UPDRS 2 (Years 2-4) | Pearson | -0.122424133 | 0.239797762 | 0.957096231 |
| lh_medialorbitofrontal_thickness | UPDRS 1A (Years 0-2) | Pearson | 0.121984523 | 0.366026433 | 0.850099671 |
| lh_medialorbitofrontal_thickness | HVLDR (Years 2-4) | Pearson | 0.12193911 | 0.241679105 | 0.351674132 |
| lh_entorhinal_thickness | UPDRS 2 (Years 0-4) | Pearson | 0.121445331 | 0.236023795 | 0.805289321 |
| rh_entorhinal_thickness | HVLDR (Years 2-4) | Pearson | -0.120155529 | 0.248685155 | 0.397896248 |
| lh_entorhinal_thickness | UPDRS 1 (Years 0-2) | Pearson | 0.119565594 | 0.256278729 | 0.655466985 |
| rh_medialorbitofrontal_thickness | SDM (Years 2-4) | Pearson | -0.117884659 | 0.25522107 | 0.98892881 |
| rh_entorhinal_thickness | HVLTR (Years 0-4) | Pearson | -0.117363066 | 0.257344531 | 0.514689062 |
| lh_insula_thickness | UPDRS 2 (Years 0-2) | Pearson | 0.117256625 | 0.25269678 | 0.673858079 |
| rh_insula_thickness | HVLTR (Years 2-4) | Pearson | -0.116465971 | 0.261024846 | 0.696066257 |
| rh_medialorbitofrontal_thickness | HVLDR (Years 2-4) | Pearson | -0.116432671 | 0.263755599 | 0.351674132 |
| lh_insula_thickness | JLO (Years 0-2) | Pearson | -0.115429089 | 0.260218393 | 0.591937202 |
| rh_insula_thickness | SF (Years 0-4) | Pearson | 0.115424905 | 0.265340483 | 0.539030984 |
| lh_medialorbitofrontal_thickness | GDS (Years 0-2) | Pearson | -0.112994649 | 0.270472556 | 0.838807744 |
| lh_entorhinal_thickness | JLO (Years 0-2) | Pearson | 0.112215461 | 0.27381141 | 0.591937202 |
| lh_lateralorbitofrontal_thickness | UPDRS 1A (Years 0-4) | Pearson | 0.110019779 | 0.415240455 | 0.824705378 |
| lh_medialorbitofrontal_thickness | UPDRS 2 (Years 0-4) | Pearson | -0.109123523 | 0.287332926 | 0.805289321 |
| rh_entorhinal_thickness | JLO (Years 0-2) | Pearson | -0.108759842 | 0.288952014 | 0.591937202 |
| rh_lateralorbitofrontal_thickness | UPDRS 1A (Years 0-2) | Pearson | 0.107735545 | 0.425049835 | 0.850099671 |
| lh_lateralorbitofrontal_thickness | JLO (Years 0-2) | Pearson | -0.10719917 | 0.295968601 | 0.591937202 |
| rh_lateralorbitofrontal_thickness | SF (Years 0-4) | Pearson | 0.106905689 | 0.302469425 | 0.539030984 |
| lh_entorhinal_thickness | JLO (Years 2-4) | Pearson | -0.104501102 | 0.316166271 | 0.798858472 |
| rh_insula_thickness | MoCA (Years 0-2) | Pearson | 0.103169961 | 0.317172299 | 0.912712544 |
| lh_entorhinal_thickness | UPDRS 1B (Years 2-4) | Pearson | -0.102517368 | 0.325490602 | 0.968631503 |
| rh_medialorbitofrontal_thickness | UPDRS 1A (Years 0-4) | Pearson | 0.100245603 | 0.458122361 | 0.824705378 |
| rh_medialorbitofrontal_thickness | SF (Years 0-4) | Pearson | 0.099599981 | 0.336894365 | 0.539030984 |
| rh_entorhinal_thickness | UPDRS 1B (Years 0-2) | Pearson | 0.099365642 | 0.348703946 | 0.72466019 |
| rh_medialorbitofrontal_thickness | SDM (Years 0-2) | Pearson | 0.099066416 | 0.334334501 | 0.828289478 |
| lh_lateralorbitofrontal_thickness | UPDRS 1B (Years 0-2) | Pearson | -0.096603687 | 0.362330095 | 0.72466019 |
| rh_lateralorbitofrontal_thickness | GDS (Years 2-4) | Pearson | -0.096329742 | 0.34792383 | 0.61073075 |
| rh_lateralorbitofrontal_thickness | GDS (Years 0-2) | Pearson | 0.095637011 | 0.351417736 | 0.838807744 |
| rh_entorhinal_thickness | UPDRS 2 (Years 0-2) | Pearson | 0.094401384 | 0.357703923 | 0.715407846 |
| rh_lateralorbitofrontal_thickness | HVLTR (Years 2-4) | Pearson | -0.093949987 | 0.365150815 | 0.73030163 |
| lh_lateralorbitofrontal_thickness | HVLDR (Years 0-2) | Pearson | -0.092386926 | 0.370646903 | 0.858607364 |
| rh_medialorbitofrontal_thickness | SF (Years 2-4) | Pearson | -0.09229421 | 0.373699729 | 0.597919567 |
| lh_lateralorbitofrontal_thickness | MoCA (Years 2-4) | Pearson | -0.091449436 | 0.380698054 | 0.964779852 |
| rh_entorhinal_thickness | UPDRS 1 (Years 2-4) | Pearson | -0.089958451 | 0.388544531 | 0.849423412 |
| rh_insula_thickness | GDS (Years 2-4) | Pearson | -0.089802542 | 0.381706719 | 0.61073075 |
| lh_insula_thickness | HVLDR (Years 0-2) | Pearson | 0.089751589 | 0.384508248 | 0.830434844 |
| rh_medialorbitofrontal_thickness | MoCA (Years 0-4) | Pearson | -0.089631571 | 0.387700136 | 0.662699261 |
| rh_insula_thickness | HVLTR (Years 0-2) | Pearson | 0.089331362 | 0.384219682 | 0.768439364 |
| lh_entorhinal_thickness | UPDRS 1 (Years 0-4) | Pearson | 0.089281152 | 0.397364211 | 0.946903699 |
| rh_entorhinal_thickness | UPDRS 1A (Years 2-4) | Pearson | 0.087624783 | 0.484168926 | 0.988015389 |
| rh_entorhinal_thickness | HVLDR (Years 0-2) | Pearson | -0.087070535 | 0.398925509 | 0.830434844 |
| lh_medialorbitofrontal_thickness | MoCA (Years 0-4) | Pearson | -0.085516313 | 0.409947162 | 0.662699261 |
| rh_insula_thickness | LNS (Years 0-4) | Pearson | 0.084931297 | 0.413169241 | 0.661070785 |
| lh_lateralorbitofrontal_thickness | LNS (Years 2-4) | Pearson | 0.084787724 | 0.413962243 | 0.985271771 |
| lh_entorhinal_thickness | UPDRS 1A (Years 0-4) | Pearson | -0.08427398 | 0.533107823 | 0.824705378 |
| rh_lateralorbitofrontal_thickness | MoCA (Years 0-2) | Pearson | 0.083788363 | 0.41700431 | 0.912712544 |
| lh_insula_thickness | GDS (Years 0-4) | Pearson | -0.083367131 | 0.416882576 | 0.988878513 |
| rh_entorhinal_thickness | UPDRS 2 (Years 0-4) | Pearson | 0.082406414 | 0.422290533 | 0.805289321 |
| lh_entorhinal_thickness | JLO (Years 0-4) | Pearson | 0.082150896 | 0.431189745 | 0.776298376 |
| rh_entorhinal_thickness | UPDRS 1B (Years 2-4) | Pearson | -0.080869883 | 0.438432915 | 0.968631503 |
| rh_entorhinal_thickness | LNS (Years 2-4) | Pearson | -0.080587927 | 0.43754854 | 0.985271771 |
| rh_medialorbitofrontal_thickness | JLO (Years 2-4) | Pearson | -0.079909224 | 0.443909565 | 0.798858472 |
| rh_entorhinal_thickness | UPDRS 1 (Years 0-2) | Pearson | 0.079724952 | 0.449983167 | 0.655466985 |
| lh_insula_thickness | UPDRS 1A (Years 0-2) | Pearson | -0.079452238 | 0.556873489 | 0.890997582 |
| lh_medialorbitofrontal_thickness | SDM (Years 0-4) | Pearson | 0.079387284 | 0.444428618 | 0.886512666 |
| rh_medialorbitofrontal_thickness | LNS (Years 0-2) | Pearson | -0.078517234 | 0.444592285 | 0.66763161 |
| rh_insula_thickness | UPDRS 1 (Years 2-4) | Pearson | -0.078375578 | 0.452731883 | 0.849423412 |
| lh_insula_thickness | HVLDR (Years 2-4) | Pearson | 0.07750868 | 0.457761493 | 0.610348657 |
| rh_entorhinal_thickness | LNS (Years 0-2) | Pearson | -0.076702287 | 0.455221827 | 0.66763161 |
| rh_medialorbitofrontal_thickness | GDS (Years 0-2) | Pearson | 0.076560897 | 0.456055768 | 0.838807744 |
| rh_lateralorbitofrontal_thickness | HVLDR (Years 0-2) | Pearson | -0.076249217 | 0.460285925 | 0.830434844 |
| rh_insula_thickness | MoCA (Years 0-4) | Pearson | 0.0760956 | 0.463599193 | 0.662699261 |
| lh_entorhinal_thickness | GDS (Years 0-4) | Pearson | 0.075183796 | 0.464222241 | 0.988878513 |
| lh_entorhinal_thickness | SDM (Years 2-4) | Pearson | 0.074817935 | 0.471160518 | 0.98892881 |
| lh_insula_thickness | JLO (Years 2-4) | Pearson | -0.074678286 | 0.474395842 | 0.798858472 |
| lh_entorhinal_thickness | SF (Years 0-4) | Pearson | -0.07373508 | 0.477620979 | 0.636827972 |
| rh_lateralorbitofrontal_thickness | UPDRS 2 (Years 0-2) | Pearson | -0.070279768 | 0.493944322 | 0.790310915 |
| rh_insula_thickness | GDS (Years 0-4) | Pearson | -0.070199458 | 0.494439256 | 0.988878513 |
| rh_medialorbitofrontal_thickness | UPDRS 1B (Years 0-2) | Pearson | -0.069035182 | 0.515546047 | 0.824873675 |
| rh_entorhinal_thickness | UPDRS 1B (Years 0-4) | Pearson | 0.068401285 | 0.519418724 | 0.932140708 |
| lh_medialorbitofrontal_thickness | MoCA (Years 0-2) | Pearson | -0.068126196 | 0.509561503 | 0.912712544 |
| lh_insula_thickness | HVLDR (Years 0-4) | Pearson | 0.067809588 | 0.516086372 | 0.88471269 |
| rh_entorhinal_thickness | MoCA (Years 0-2) | Pearson | 0.067367519 | 0.514298517 | 0.912712544 |
| lh_lateralorbitofrontal_thickness | UPDRS 2 (Years 2-4) | Pearson | 0.067032101 | 0.520920398 | 0.957096231 |
| lh_lateralorbitofrontal_thickness | MoCA (Years 0-4) | Pearson | -0.066819651 | 0.519980638 | 0.662699261 |
| lh_medialorbitofrontal_thickness | HVLDR (Years 0-2) | Pearson | -0.066614596 | 0.519021778 | 0.830434844 |
| rh_lateralorbitofrontal_thickness | UPDRS 2 (Years 2-4) | Pearson | 0.066198016 | 0.526131696 | 0.957096231 |
| lh_lateralorbitofrontal_thickness | UPDRS 1A (Years 2-4) | Pearson | 0.065381096 | 0.601970807 | 0.988015389 |
| rh_entorhinal_thickness | HVLDR (Years 0-4) | Pearson | -0.064697549 | 0.53557201 | 0.88471269 |
| lh_entorhinal_thickness | MoCA (Years 0-4) | Pearson | 0.064123167 | 0.536999065 | 0.662699261 |
| rh_insula_thickness | LNS (Years 0-2) | Pearson | -0.06397104 | 0.533605164 | 0.66763161 |
| rh_lateralorbitofrontal_thickness | UPDRS 1 (Years 2-4) | Pearson | 0.063572318 | 0.542706146 | 0.849423412 |
| lh_lateralorbitofrontal_thickness | JLO (Years 2-4) | Pearson | 0.063126131 | 0.545547879 | 0.798858472 |
| rh_medialorbitofrontal_thickness | LNS (Years 0-4) | Pearson | -0.062811883 | 0.545373415 | 0.727164553 |
| rh_entorhinal_thickness | UPDRS 1 (Years 0-4) | Pearson | 0.062511371 | 0.553871052 | 0.946903699 |
| rh_insula_thickness | UPDRS 1 (Years 0-2) | Pearson | -0.062191185 | 0.555907261 | 0.655466985 |
| lh_medialorbitofrontal_thickness | SDM (Years 2-4) | Pearson | 0.061801615 | 0.551868544 | 0.98892881 |
| rh_medialorbitofrontal_thickness | HVLTR (Years 2-4) | Pearson | -0.061801553 | 0.55186894 | 0.77405223 |
| rh_medialorbitofrontal_thickness | UPDRS 1 (Years 0-2) | Pearson | -0.060928833 | 0.563969956 | 0.655466985 |
| rh_medialorbitofrontal_thickness | JLO (Years 0-4) | Pearson | -0.060095 | 0.56504291 | 0.776298376 |
| lh_insula_thickness | UPDRS 1 (Years 0-2) | Pearson | -0.059442502 | 0.573533612 | 0.655466985 |
| rh_medialorbitofrontal_thickness | HVLDR (Years 0-4) | Pearson | -0.059238773 | 0.570608883 | 0.88471269 |
| lh_lateralorbitofrontal_thickness | HVLDR (Years 0-4) | Pearson | 0.058210109 | 0.579417311 | 0.786210912 |
| lh_insula_thickness | HVLTR (Years 0-4) | Pearson | -0.05795004 | 0.576968053 | 0.864927651 |
| lh_insula_thickness | MoCA (Years 0-4) | Pearson | -0.057511082 | 0.579861853 | 0.662699261 |
| rh_lateralorbitofrontal_thickness | LNS (Years 0-2) | Pearson | -0.056254383 | 0.584177658 | 0.66763161 |
| lh_medialorbitofrontal_thickness | HVLDR (Years 0-4) | Pearson | 0.054933519 | 0.600981213 | 0.786210912 |
| lh_medialorbitofrontal_thickness | UPDRS 1A (Years 0-4) | Pearson | 0.054869099 | 0.685197742 | 0.824705378 |
| lh_lateralorbitofrontal_thickness | HVLDR (Years 2-4) | Pearson | 0.054709519 | 0.600470617 | 0.603449095 |
| rh_lateralorbitofrontal_thickness | SDM (Years 0-2) | Pearson | 0.054298795 | 0.597333918 | 0.828289478 |
| rh_lateralorbitofrontal_thickness | HVLDR (Years 2-4) | Pearson | 0.054263404 | 0.603449095 | 0.603449095 |
| rh_lateralorbitofrontal_thickness | LNS (Years 2-4) | Pearson | 0.053082276 | 0.609425829 | 0.985271771 |
| rh_entorhinal_thickness | SDM (Years 0-2) | Pearson | -0.052759511 | 0.607781757 | 0.828289478 |
| rh_lateralorbitofrontal_thickness | HVLTR (Years 0-2) | Pearson | 0.052375236 | 0.610402467 | 0.973195158 |
| rh_entorhinal_thickness | UPDRS 1A (Years 0-4) | Pearson | 0.052353987 | 0.698924673 | 0.824705378 |
| lh_lateralorbitofrontal_thickness | GDS (Years 2-4) | Pearson | 0.051716073 | 0.61490933 | 0.718678183 |
| lh_medialorbitofrontal_thickness | JLO (Years 0-2) | Pearson | -0.051516479 | 0.616276845 | 0.845868798 |
| rh_medialorbitofrontal_thickness | UPDRS 2 (Years 0-2) | Pearson | -0.051364387 | 0.617319778 | 0.823093037 |
| rh_insula_thickness | UPDRS 1B (Years 0-2) | Pearson | -0.051015519 | 0.631054997 | 0.841406663 |
| lh_insula_thickness | SDM (Years 0-2) | Pearson | 0.050797012 | 0.621217109 | 0.828289478 |
| rh_entorhinal_thickness | SDM (Years 2-4) | Pearson | 0.050097126 | 0.629716979 | 0.98892881 |
| lh_entorhinal_thickness | GDS (Years 2-4) | Pearson | 0.049691148 | 0.62884341 | 0.718678183 |
| rh_entorhinal_thickness | GDS (Years 0-2) | Pearson | 0.049573045 | 0.629660202 | 0.838807744 |
| rh_entorhinal_thickness | HVLDR (Years 0-2) | Pearson | 0.049226139 | 0.633870272 | 0.858607364 |
| rh_lateralorbitofrontal_thickness | JLO (Years 0-4) | Pearson | 0.048981289 | 0.639199186 | 0.776298376 |
| lh_entorhinal_thickness | MoCA (Years 0-2) | Pearson | 0.048981072 | 0.635562297 | 0.912712544 |
| rh_insula_thickness | JLO (Years 0-2) | Pearson | -0.048888735 | 0.634401598 | 0.845868798 |
| rh_lateralorbitofrontal_thickness | JLO (Years 2-4) | Pearson | 0.048519143 | 0.642368252 | 0.798858472 |
| rh_lateralorbitofrontal_thickness | GDS (Years 0-4) | Pearson | 0.048313993 | 0.638395277 | 0.994036661 |
| lh_insula_thickness | UPDRS 1A (Years 0-4) | Pearson | -0.04823398 | 0.721617205 | 0.824705378 |
| rh_medialorbitofrontal_thickness | HVLDR (Years 2-4) | Pearson | -0.048162558 | 0.644817846 | 0.709386732 |
| rh_insula_thickness | UPDRS 1A (Years 2-4) | Pearson | -0.047908888 | 0.702463913 | 0.988015389 |
| rh_medialorbitofrontal_thickness | UPDRS 1A (Years 2-4) | Pearson | 0.047541517 | 0.704640314 | 0.988015389 |
| rh_medialorbitofrontal_thickness | HVLTR (Years 0-4) | Pearson | -0.047341489 | 0.648695738 | 0.864927651 |
| lh_lateralorbitofrontal_thickness | UPDRS 2 (Years 0-4) | Pearson | -0.047118361 | 0.646736358 | 0.805289321 |
| lh_entorhinal_thickness | HVLDR (Years 0-2) | Pearson | 0.046445158 | 0.65317976 | 0.858607364 |
| rh_lateralorbitofrontal_thickness | UPDRS 2 (Years 0-4) | Pearson | -0.04520344 | 0.660186518 | 0.805289321 |
| rh_lateralorbitofrontal_thickness | SDM (Years 2-4) | Pearson | -0.045068197 | 0.664523917 | 0.98892881 |
| rh_medialorbitofrontal_thickness | SDM (Years 0-4) | Pearson | 0.045016528 | 0.664885426 | 0.886512666 |
| rh_entorhinal_thickness | HVLDR (Years 0-4) | Pearson | 0.044982588 | 0.668544661 | 0.786210912 |
| lh_entorhinal_thickness | HVLDR (Years 0-4) | Pearson | -0.043971564 | 0.673886798 | 0.88471269 |
| rh_insula_thickness | UPDRS 2 (Years 0-4) | Pearson | 0.043719526 | 0.670684452 | 0.805289321 |
| rh_medialorbitofrontal_thickness | HVLDR (Years 0-4) | Pearson | 0.043484489 | 0.678966749 | 0.786210912 |
| lh_insula_thickness | UPDRS 1B (Years 2-4) | Pearson | -0.042541299 | 0.683920601 | 0.968631503 |
| lh_lateralorbitofrontal_thickness | SDM (Years 0-4) | Pearson | -0.042420384 | 0.683146626 | 0.886512666 |
| lh_insula_thickness | GDS (Years 0-2) | Pearson | -0.042130726 | 0.681994913 | 0.838807744 |
| lh_lateralorbitofrontal_thickness | HVLTR (Years 2-4) | Pearson | -0.041004781 | 0.693182171 | 0.77405223 |
| lh_entorhinal_thickness | MoCA (Years 2-4) | Pearson | 0.040541157 | 0.698044501 | 0.964779852 |
| lh_insula_thickness | UPDRS 1 (Years 2-4) | Pearson | -0.040221694 | 0.700310088 | 0.849423412 |
| rh_lateralorbitofrontal_thickness | MoCA (Years 0-4) | Pearson | 0.03959139 | 0.703255151 | 0.703255151 |
| lh_insula_thickness | MoCA (Years 0-2) | Pearson | -0.039506987 | 0.702337915 | 0.912712544 |
| lh_insula_thickness | UPDRS 2 (Years 0-4) | Pearson | 0.038980479 | 0.704628155 | 0.805289321 |
| rh_insula_thickness | HVLDR (Years 2-4) | Pearson | -0.038945492 | 0.709386732 | 0.709386732 |
| rh_entorhinal_thickness | SDM (Years 0-4) | Pearson | -0.038788304 | 0.709001647 | 0.886512666 |
| rh_lateralorbitofrontal_thickness | UPDRS 1 (Years 0-2) | Pearson | -0.037392841 | 0.723429191 | 0.723429191 |
| lh_medialorbitofrontal_thickness | JLO (Years 2-4) | Pearson | 0.037287306 | 0.721240844 | 0.798858472 |
| rh_lateralorbitofrontal_thickness | SF (Years 2-4) | Pearson | -0.037242392 | 0.720109179 | 0.877224689 |
| lh_lateralorbitofrontal_thickness | JLO (Years 0-4) | Pearson | -0.036469979 | 0.727108318 | 0.776298376 |
| lh_insula_thickness | HVLTR (Years 2-4) | Pearson | -0.036279941 | 0.727054133 | 0.77405223 |
| rh_insula_thickness | HVLDR (Years 0-2) | Pearson | 0.035788742 | 0.729211857 | 0.835242021 |
| lh_insula_thickness | HVLTR (Years 0-2) | Pearson | -0.035505409 | 0.729896369 | 0.973195158 |
| lh_insula_thickness | UPDRS 1B (Years 0-2) | Pearson | -0.034893671 | 0.742637252 | 0.848728288 |
| lh_lateralorbitofrontal_thickness | HVLDR (Years 0-2) | Pearson | 0.034214103 | 0.740694368 | 0.835242021 |
| rh_lateralorbitofrontal_thickness | HVLDR (Years 0-4) | Pearson | 0.033412682 | 0.749193599 | 0.88471269 |
| lh_medialorbitofrontal_thickness | LNS (Years 2-4) | Pearson | 0.033286099 | 0.748796566 | 0.985271771 |
| lh_insula_thickness | HVLDR (Years 0-2) | Pearson | -0.032396385 | 0.754020324 | 0.858607364 |
| lh_entorhinal_thickness | GDS (Years 0-2) | Pearson | 0.032356543 | 0.75304549 | 0.838807744 |
| lh_medialorbitofrontal_thickness | SF (Years 0-4) | Pearson | 0.031063307 | 0.765068483 | 0.874363981 |
| lh_entorhinal_thickness | SF (Years 2-4) | Pearson | -0.030722635 | 0.767571603 | 0.877224689 |
| lh_medialorbitofrontal_thickness | SDM (Years 0-2) | Pearson | 0.030334557 | 0.768027807 | 0.877746065 |
| lh_entorhinal_thickness | SF (Years 0-2) | Pearson | -0.030066938 | 0.77001732 | 0.782184561 |
| lh_medialorbitofrontal_thickness | HVLTR (Years 2-4) | Pearson | 0.029842128 | 0.77405223 | 0.77405223 |
| rh_medialorbitofrontal_thickness | UPDRS 1 (Years 0-4) | Pearson | 0.029809492 | 0.77788526 | 0.946903699 |
| lh_medialorbitofrontal_thickness | JLO (Years 0-4) | Pearson | -0.029698336 | 0.776298376 | 0.776298376 |
| lh_entorhinal_thickness | HVLDR (Years 0-4) | Pearson | 0.029281544 | 0.780535234 | 0.786210912 |
| rh_lateralorbitofrontal_thickness | HVLDR (Years 0-4) | Pearson | 0.02850549 | 0.786210912 | 0.786210912 |
| lh_entorhinal_thickness | UPDRS 1A (Years 0-2) | Pearson | -0.028492384 | 0.833363526 | 0.930363987 |
| rh_entorhinal_thickness | SF (Years 0-2) | Pearson | 0.028434551 | 0.782184561 | 0.782184561 |
| lh_insula_thickness | SDM (Years 0-4) | Pearson | -0.02677664 | 0.796732938 | 0.886512666 |
| rh_insula_thickness | UPDRS 1B (Years 2-4) | Pearson | -0.026647667 | 0.798763912 | 0.968631503 |
| rh_entorhinal_thickness | JLO (Years 2-4) | Pearson | -0.026634876 | 0.798858472 | 0.798858472 |
| lh_lateralorbitofrontal_thickness | GDS (Years 0-2) | Pearson | -0.026486436 | 0.796773823 | 0.838807744 |
| rh_insula_thickness | UPDRS 1B (Years 0-4) | Pearson | 0.026229624 | 0.80506442 | 0.932140708 |
| lh_insula_thickness | MoCA (Years 2-4) | Pearson | -0.025079018 | 0.810381042 | 0.964779852 |
| lh_insula_thickness | SDM (Years 2-4) | Pearson | -0.024474587 | 0.813878067 | 0.98892881 |
| rh_lateralorbitofrontal_thickness | UPDRS 1A (Years 0-4) | Pearson | 0.024467258 | 0.85663583 | 0.85663583 |
| lh_medialorbitofrontal_thickness | MoCA (Years 2-4) | Pearson | -0.023948116 | 0.818782014 | 0.964779852 |
| lh_medialorbitofrontal_thickness | UPDRS 1B (Years 2-4) | Pearson | 0.022633469 | 0.828573667 | 0.968631503 |
| lh_medialorbitofrontal_thickness | UPDRS 1 (Years 2-4) | Pearson | 0.022389455 | 0.830394048 | 0.849423412 |
| lh_entorhinal_thickness | HVLDR (Years 0-2) | Pearson | 0.021506476 | 0.835242021 | 0.835242021 |
| rh_insula_thickness | GDS (Years 0-2) | Pearson | -0.020923024 | 0.838807744 | 0.838807744 |
| rh_medialorbitofrontal_thickness | LNS (Years 2-4) | Pearson | -0.02082973 | 0.841201268 | 0.985271771 |
| rh_lateralorbitofrontal_thickness | MoCA (Years 2-4) | Pearson | -0.020544986 | 0.84418237 | 0.964779852 |
| rh_lateralorbitofrontal_thickness | UPDRS 1 (Years 0-4) | Pearson | 0.020063902 | 0.849439191 | 0.946903699 |
| lh_lateralorbitofrontal_thickness | UPDRS 1 (Years 2-4) | Pearson | 0.019845568 | 0.849423412 | 0.849423412 |
| rh_medialorbitofrontal_thickness | HVLDR (Years 0-2) | Pearson | -0.019809216 | 0.848082032 | 0.858607364 |
| lh_medialorbitofrontal_thickness | UPDRS 2 (Years 0-2) | Pearson | 0.01887371 | 0.854412722 | 0.899151337 |
| rh_lateralorbitofrontal_thickness | HVLDR (Years 0-2) | Pearson | -0.01842185 | 0.858607364 | 0.858607364 |
| lh_entorhinal_thickness | UPDRS 1A (Years 2-4) | Pearson | 0.018397627 | 0.883432237 | 0.988015389 |
| rh_lateralorbitofrontal_thickness | UPDRS 1B (Years 0-2) | Pearson | -0.017886673 | 0.866360895 | 0.866360895 |
| rh_entorhinal_thickness | UPDRS 1A (Years 0-2) | Pearson | -0.016417226 | 0.903525138 | 0.930363987 |
| lh_lateralorbitofrontal_thickness | HVLDR (Years 0-4) | Pearson | 0.016391052 | 0.8754014 | 0.88471269 |
| lh_insula_thickness | SF (Years 0-4) | Pearson | -0.015224686 | 0.883577286 | 0.883577286 |
| lh_medialorbitofrontal_thickness | HVLDR (Years 0-4) | Pearson | -0.015157317 | 0.88471269 | 0.88471269 |
| lh_insula_thickness | UPDRS 1B (Years 0-4) | Pearson | 0.014974567 | 0.887963611 | 0.932140708 |
| lh_lateralorbitofrontal_thickness | UPDRS 1 (Years 0-4) | Pearson | 0.014949155 | 0.88752658 | 0.946903699 |
| rh_lateralorbitofrontal_thickness | SDM (Years 0-4) | Pearson | 0.014838216 | 0.886512666 | 0.886512666 |
| lh_insula_thickness | UPDRS 2 (Years 2-4) | Pearson | -0.014796024 | 0.887442482 | 0.957096231 |
| rh_insula_thickness | HVLTR (Years 0-4) | Pearson | -0.014783308 | 0.886929834 | 0.940200121 |
| rh_lateralorbitofrontal_thickness | UPDRS 1B (Years 0-4) | Pearson | 0.014488107 | 0.891580728 | 0.932140708 |
| lh_lateralorbitofrontal_thickness | MoCA (Years 0-2) | Pearson | 0.013806273 | 0.893791935 | 0.912712544 |
| lh_lateralorbitofrontal_thickness | SDM (Years 0-2) | Pearson | -0.013053427 | 0.899019457 | 0.899019457 |
| lh_lateralorbitofrontal_thickness | UPDRS 2 (Years 0-2) | Pearson | 0.013036289 | 0.899151337 | 0.899151337 |
| lh_entorhinal_thickness | HVLTR (Years 0-2) | Pearson | 0.01243149 | 0.90380714 | 0.981864514 |
| rh_medialorbitofrontal_thickness | UPDRS 2 (Years 0-4) | Pearson | -0.012247134 | 0.905226985 | 0.905226985 |
| rh_lateralorbitofrontal_thickness | JLO (Years 0-2) | Pearson | 0.012030535 | 0.906895536 | 0.937254049 |
| rh_insula_thickness | UPDRS 1A (Years 0-2) | Pearson | -0.01183651 | 0.930363987 | 0.930363987 |
| rh_medialorbitofrontal_thickness | MoCA (Years 0-2) | Pearson | 0.011335951 | 0.912712544 | 0.912712544 |
| rh_medialorbitofrontal_thickness | UPDRS 1B (Years 0-4) | Pearson | 0.01047289 | 0.921514139 | 0.932140708 |
| lh_lateralorbitofrontal_thickness | UPDRS 1B (Years 0-4) | Pearson | -0.009051277 | 0.932140708 | 0.932140708 |
| lh_entorhinal_thickness | UPDRS 2 (Years 2-4) | Pearson | -0.008940588 | 0.931844728 | 0.957096231 |
| rh_insula_thickness | UPDRS 1 (Years 0-4) | Pearson | -0.008557619 | 0.935472976 | 0.946903699 |
| lh_entorhinal_thickness | LNS (Years 0-2) | Pearson | -0.008437206 | 0.93462997 | 0.93462997 |
| rh_medialorbitofrontal_thickness | JLO (Years 0-2) | Pearson | 0.008097817 | 0.937254049 | 0.937254049 |
| rh_insula_thickness | SF (Years 2-4) | Pearson | 0.008009318 | 0.938597331 | 0.938597331 |
| rh_lateralorbitofrontal_thickness | HVLTR (Years 0-4) | Pearson | -0.00779986 | 0.940200121 | 0.940200121 |
| rh_insula_thickness | SDM (Years 2-4) | Pearson | 0.007405275 | 0.943220153 | 0.98892881 |
| lh_insula_thickness | UPDRS 1 (Years 0-4) | Pearson | 0.007039225 | 0.946903699 | 0.946903699 |
| rh_medialorbitofrontal_thickness | UPDRS 2 (Years 2-4) | Pearson | 0.005981339 | 0.954373282 | 0.957096231 |
| rh_insula_thickness | UPDRS 2 (Years 2-4) | Pearson | -0.00562403 | 0.957096231 | 0.957096231 |
| lh_medialorbitofrontal_thickness | GDS (Years 0-4) | Pearson | -0.004903792 | 0.961978505 | 0.994036661 |
| lh_lateralorbitofrontal_thickness | GDS (Years 0-4) | Pearson | 0.004546084 | 0.964750145 | 0.994036661 |
| rh_lateralorbitofrontal_thickness | UPDRS 1A (Years 2-4) | Pearson | -0.004354928 | 0.972315954 | 0.988015389 |
| rh_lateralorbitofrontal_thickness | UPDRS 1B (Years 2-4) | Pearson | 0.00424463 | 0.967612583 | 0.968631503 |
| rh_lateralorbitofrontal_thickness | LNS (Years 0-4) | Pearson | -0.004184697 | 0.967895733 | 0.97358048 |
| lh_lateralorbitofrontal_thickness | UPDRS 1B (Years 2-4) | Pearson | -0.004111023 | 0.968631503 | 0.968631503 |
| lh_insula_thickness | LNS (Years 2-4) | Pearson | -0.003996014 | 0.969342556 | 0.985271771 |
| lh_insula_thickness | GDS (Years 2-4) | Pearson | 0.003844677 | 0.970186059 | 0.970186059 |
| lh_entorhinal_thickness | LNS (Years 0-4) | Pearson | -0.003443411 | 0.97358048 | 0.97358048 |
| lh_medialorbitofrontal_thickness | UPDRS 1A (Years 2-4) | Pearson | 0.002731751 | 0.98263234 | 0.988015389 |
| rh_medialorbitofrontal_thickness | HVLTR (Years 0-2) | Pearson | -0.002338336 | 0.981864514 | 0.981864514 |
| lh_entorhinal_thickness | LNS (Years 2-4) | Pearson | -0.001919379 | 0.985271771 | 0.985271771 |
| lh_insula_thickness | UPDRS 1A (Years 2-4) | Pearson | 0.001884978 | 0.988015389 | 0.988015389 |
| lh_lateralorbitofrontal_thickness | SDM (Years 2-4) | Pearson | -0.001442759 | 0.98892881 | 0.98892881 |
| rh_insula_thickness | MoCA (Years 2-4) | Pearson | -0.00107415 | 0.99180194 | 0.99180194 |
| rh_entorhinal_thickness | GDS (Years 0-4) | Pearson | -0.000768837 | 0.994036661 | 0.994036661 |
| Left-Thalamus_volume | HVLDR (Years 0-4) | Pearson | 0.312427445 | 0.002171633 | 0.026059596 |
| Left-Amygdala_volume | UPDRS 2 (Years 2-4) | Pearson | 0.30136646 | 0.003160185 | 0.037922222 |
| Right-Amygdala_volume | HVLDR (Years 0-2) | Pearson | 0.297098421 | 0.003287379 | 0.039448547 |
| Right-Thalamus_volume | HVLDR (Years 0-4) | Pearson | 0.289176473 | 0.004701305 | 0.028207827 |
| lh_medialorbitofrontal_volume | HVLTR (Years 2-4) | Pearson | 0.279387104 | 0.006108379 | 0.073300553 |
| Left-Amygdala_volume | LNS (Years 2-4) | Pearson | 0.27575377 | 0.006834782 | 0.082017382 |
| Right-Amygdala_volume | HVLTR (Years 0-4) | Pearson | 0.273200778 | 0.007389878 | 0.047345545 |
| rh_insula_volume | HVLTR (Years 0-4) | Pearson | 0.271037777 | 0.007890924 | 0.047345545 |
| Right-Amygdala_volume | JLO (Years 2-4) | Pearson | 0.264685555 | 0.009936264 | 0.070202415 |
| rh_insula_volume | JLO (Years 2-4) | Pearson | 0.257543422 | 0.012210793 | 0.070202415 |
| rh_insula_volume | GDS (Years 2-4) | Pearson | -0.255048712 | 0.011694924 | 0.140339083 |
| Left-Amygdala_volume | HVLTR (Years 0-4) | Pearson | 0.252259545 | 0.013655644 | 0.054622577 |
| lh_entorhinal_volume | JLO (Years 0-4) | Pearson | 0.244877761 | 0.01737149 | 0.110193217 |
| Left-Amygdala_volume | HVLDR (Years 0-2) | Pearson | 0.244838367 | 0.016207663 | 0.09724598 |
| Right-Thalamus_volume | JLO (Years 2-4) | Pearson | 0.243995809 | 0.017792283 | 0.070202415 |
| rh_insula_volume | LNS (Years 2-4) | Pearson | 0.242828544 | 0.017736525 | 0.106419147 |
| Left-Amygdala_volume | JLO (Years 0-4) | Pearson | 0.242822799 | 0.018365536 | 0.110193217 |
| rh_lateralorbitofrontal_volume | SDM (Years 0-4) | Pearson | 0.242118716 | 0.018082334 | 0.216988002 |
| Right-Amygdala_volume | HVLDR (Years 2-4) | Pearson | 0.241237145 | 0.019165678 | 0.229988132 |
| Right-Thalamus_volume | SF (Years 2-4) | Pearson | 0.239392646 | 0.019464929 | 0.233579145 |
| lh_medialorbitofrontal_volume | HVLDR (Years 2-4) | Pearson | 0.2355525 | 0.022285306 | 0.267423676 |
| lh_insula_volume | HVLTR (Years 0-4) | Pearson | 0.235280222 | 0.02172221 | 0.065166631 |
| lh_entorhinal_volume | JLO (Years 0-2) | Pearson | 0.23429109 | 0.02089729 | 0.25076748 |
| Left-Thalamus_volume | JLO (Years 2-4) | Pearson | 0.233684794 | 0.023400805 | 0.070202415 |
| lh_medialorbitofrontal_volume | SDM (Years 2-4) | Pearson | 0.230458231 | 0.024651003 | 0.146785868 |
| lh_lateralorbitofrontal_volume | SF (Years 0-4) | Pearson | 0.228165816 | 0.026157421 | 0.313889054 |
| lh_medialorbitofrontal_volume | SF (Years 0-2) | Pearson | 0.224994849 | 0.026711564 | 0.19596899 |
| rh_medialorbitofrontal_volume | MoCA (Years 2-4) | Pearson | -0.223311888 | 0.030501155 | 0.366013864 |
| Left-Thalamus_volume | HVLTR (Years 0-4) | Pearson | 0.223269848 | 0.029638341 | 0.071132018 |
| lh_insula_volume | UPDRS 2 (Years 0-2) | Pearson | 0.222058041 | 0.028812939 | 0.345755273 |
| lh_lateralorbitofrontal_volume | HVLDR (Years 0-4) | Pearson | 0.220998075 | 0.03231202 | 0.118591197 |
| Right-Thalamus_volume | HVLTR (Years 0-4) | Pearson | 0.215379932 | 0.036069623 | 0.072139246 |
| Left-Thalamus_volume | SDM (Years 2-4) | Pearson | 0.215348373 | 0.036097528 | 0.146785868 |
| Right-Thalamus_volume | SDM (Years 2-4) | Pearson | 0.214675885 | 0.036696467 | 0.146785868 |
| Right-Amygdala_volume | UPDRS 1B (Years 0-2) | Pearson | -0.214453892 | 0.041221589 | 0.255850252 |
| lh_medialorbitofrontal_volume | UPDRS 1B (Years 0-2) | Pearson | -0.213007699 | 0.042641709 | 0.255850252 |
| Left-Amygdala_volume | HVLDR (Years 0-4) | Pearson | 0.212740052 | 0.039530399 | 0.118591197 |
| Right-Amygdala_volume | UPDRS 1 (Years 0-2) | Pearson | -0.211888696 | 0.042594703 | 0.511136438 |
| lh_lateralorbitofrontal_volume | SF (Years 0-2) | Pearson | 0.21138417 | 0.037667734 | 0.19596899 |
| rh_lateralorbitofrontal_volume | SDM (Years 0-2) | Pearson | 0.20951195 | 0.039434728 | 0.47321674 |
| Right-Thalamus_volume | HVLTR (Years 0-2) | Pearson | 0.20804378 | 0.040868356 | 0.347243814 |
| Left-Amygdala_volume | HVLTR (Years 2-4) | Pearson | 0.20213218 | 0.049485235 | 0.203522417 |
| lh_insula_volume | HVLTR (Years 2-4) | Pearson | 0.200932689 | 0.050880604 | 0.203522417 |
| rh_medialorbitofrontal_volume | SF (Years 0-2) | Pearson | 0.200451916 | 0.048992248 | 0.19596899 |
| rh_medialorbitofrontal_volume | UPDRS 1B (Years 2-4) | Pearson | 0.199909224 | 0.053383356 | 0.632931278 |
| Right-Amygdala_volume | HVLDR (Years 0-4) | Pearson | 0.197397062 | 0.056515649 | 0.135637558 |
| Right-Amygdala_volume | UPDRS 2 (Years 2-4) | Pearson | 0.192397493 | 0.063197167 | 0.379183003 |
| lh_entorhinal_volume | UPDRS 1A (Years 2-4) | Pearson | -0.192214531 | 0.122061373 | 0.650429989 |
| Left-Thalamus_volume | SF (Years 2-4) | Pearson | 0.191426538 | 0.063123563 | 0.378741378 |
| lh_insula_volume | JLO (Years 2-4) | Pearson | 0.190909181 | 0.065305786 | 0.156733886 |
| Left-Thalamus_volume | HVLDR (Years 0-4) | Pearson | 0.188712808 | 0.070050923 | 0.517199704 |
| rh_lateralorbitofrontal_volume | LNS (Years 2-4) | Pearson | 0.188450278 | 0.067415518 | 0.240164115 |
| rh_entorhinal_volume | GDS (Years 2-4) | Pearson | -0.185947255 | 0.068217764 | 0.353510485 |
| rh_lateralorbitofrontal_volume | SF (Years 0-2) | Pearson | 0.18171394 | 0.074857591 | 0.224572772 |
| Right-Amygdala_volume | LNS (Years 2-4) | Pearson | 0.180495393 | 0.080054705 | 0.240164115 |
| rh_insula_volume | HVLTR (Years 2-4) | Pearson | 0.180234968 | 0.080498489 | 0.241495468 |
| rh_insula_volume | SDM (Years 0-4) | Pearson | 0.178706296 | 0.083143233 | 0.452698616 |
| rh_entorhinal_volume | UPDRS 1A (Years 2-4) | Pearson | -0.178384144 | 0.15184853 | 0.650429989 |
| rh_entorhinal_volume | JLO (Years 0-4) | Pearson | 0.175945 | 0.089831731 | 0.25942128 |
| Left-Thalamus_volume | JLO (Years 0-2) | Pearson | -0.173018446 | 0.090122786 | 0.540736719 |
| Left-Amygdala_volume | SDM (Years 2-4) | Pearson | 0.172679183 | 0.094251885 | 0.282755654 |
| Left-Amygdala_volume | JLO (Years 2-4) | Pearson | 0.17222466 | 0.096941385 | 0.19388277 |
| rh_medialorbitofrontal_volume | MoCA (Years 0-4) | Pearson | -0.171245035 | 0.097060332 | 0.806697476 |
| rh_entorhinal_volume | UPDRS 1B (Years 0-4) | Pearson | 0.169425416 | 0.108383113 | 0.906836681 |
| lh_medialorbitofrontal_volume | UPDRS 1 (Years 0-2) | Pearson | -0.169039048 | 0.1072207 | 0.638788684 |
| Right-Amygdala_volume | JLO (Years 0-4) | Pearson | 0.168247603 | 0.105023403 | 0.25942128 |
| rh_lateralorbitofrontal_volume | MoCA (Years 0-2) | Pearson | 0.167125833 | 0.103628794 | 0.637710903 |
| Right-Thalamus_volume | MoCA (Years 0-2) | Pearson | 0.165871056 | 0.10628515 | 0.637710903 |
| Right-Amygdala_volume | HVLTR (Years 0-2) | Pearson | 0.165706573 | 0.104782187 | 0.347243814 |
| lh_insula_volume | GDS (Years 2-4) | Pearson | -0.163960061 | 0.10854538 | 0.353510485 |
| Right-Amygdala_volume | HVLDR (Years 0-4) | Pearson | 0.163843064 | 0.116577921 | 0.517199704 |
| rh_insula_volume | UPDRS 2 (Years 0-2) | Pearson | 0.163602283 | 0.109329112 | 0.528349452 |
| rh_medialorbitofrontal_volume | SDM (Years 0-4) | Pearson | 0.163592289 | 0.113174654 | 0.452698616 |
| lh_lateralorbitofrontal_volume | UPDRS 1A (Years 2-4) | Pearson | 0.163149304 | 0.190569505 | 0.650429989 |
| Left-Thalamus_volume | HVLTR (Years 0-2) | Pearson | 0.162509139 | 0.111750995 | 0.347243814 |
| rh_lateralorbitofrontal_volume | JLO (Years 0-4) | Pearson | 0.162411941 | 0.117823033 | 0.25942128 |
| lh_lateralorbitofrontal_volume | HVLDR (Years 0-2) | Pearson | 0.159558214 | 0.120463246 | 0.322168669 |
| lh_insula_volume | HVLDR (Years 2-4) | Pearson | 0.159256568 | 0.125228203 | 0.624829475 |
| lh_medialorbitofrontal_volume | LNS (Years 2-4) | Pearson | 0.158262206 | 0.125571865 | 0.301372477 |
| lh_lateralorbitofrontal_volume | JLO (Years 0-4) | Pearson | 0.157415564 | 0.12971064 | 0.25942128 |
| rh_insula_volume | UPDRS 1B (Years 0-2) | Pearson | -0.15704178 | 0.137116581 | 0.548466326 |
| rh_insula_volume | HVLDR (Years 0-4) | Pearson | 0.155797678 | 0.133750077 | 0.253861909 |
| lh_medialorbitofrontal_volume | HVLTR (Years 0-2) | Pearson | -0.155286396 | 0.128813791 | 0.347243814 |
| Right-Amygdala_volume | HVLTR (Years 2-4) | Pearson | 0.154919935 | 0.133860814 | 0.321265954 |
| Right-Thalamus_volume | MoCA (Years 0-4) | Pearson | 0.154688601 | 0.134449579 | 0.806697476 |
| Left-Thalamus_volume | SF (Years 0-2) | Pearson | -0.154379715 | 0.131089334 | 0.314614402 |
| rh_insula_volume | HVLDR (Years 0-2) | Pearson | 0.154329658 | 0.133273378 | 0.322168669 |
| Right-Amygdala_volume | UPDRS 1B (Years 2-4) | Pearson | 0.15432746 | 0.137503196 | 0.632931278 |
| rh_lateralorbitofrontal_volume | UPDRS 1A (Years 2-4) | Pearson | 0.15383361 | 0.217490785 | 0.650429989 |
| rh_entorhinal_volume | HVLDR (Years 2-4) | Pearson | -0.152899309 | 0.141224885 | 0.714860413 |
| lh_entorhinal_volume | GDS (Years 2-4) | Pearson | -0.152543187 | 0.135792392 | 0.353510485 |
| rh_medialorbitofrontal_volume | HVLDR (Years 0-2) | Pearson | 0.151937473 | 0.139469265 | 0.939076039 |
| lh_medialorbitofrontal_volume | JLO (Years 2-4) | Pearson | 0.15151158 | 0.144913699 | 0.248423485 |
| lh_insula_volume | HVLDR (Years 0-4) | Pearson | 0.150705491 | 0.149318003 | 0.517199704 |
| rh_lateralorbitofrontal_volume | UPDRS 1A (Years 0-2) | Pearson | -0.150566603 | 0.263585671 | 0.987481291 |
| Right-Amygdala_volume | UPDRS 2 (Years 0-2) | Pearson | 0.150475112 | 0.141240686 | 0.528349452 |
| lh_insula_volume | HVLDR (Years 0-4) | Pearson | 0.150339256 | 0.148086114 | 0.253861909 |
| lh_insula_volume | LNS (Years 0-2) | Pearson | -0.149623371 | 0.143532042 | 0.623771751 |
| rh_insula_volume | HVLTR (Years 0-2) | Pearson | 0.1491987 | 0.144684923 | 0.347243814 |
| lh_insula_volume | GDS (Years 0-4) | Pearson | -0.148622856 | 0.146259337 | 0.704688723 |
| lh_lateralorbitofrontal_volume | GDS (Years 2-4) | Pearson | -0.148246227 | 0.147296036 | 0.353510485 |
| Left-Thalamus_volume | HVLDR (Years 0-2) | Pearson | 0.147374386 | 0.151889325 | 0.322168669 |
| Left-Thalamus_volume | HVLDR (Years 0-2) | Pearson | 0.145747345 | 0.156512673 | 0.939076039 |
| lh_entorhinal_volume | GDS (Years 0-2) | Pearson | 0.145286162 | 0.155637107 | 0.973638175 |
| lh_medialorbitofrontal_volume | SDM (Years 0-4) | Pearson | 0.145118013 | 0.160571977 | 0.481715931 |
| lh_lateralorbitofrontal_volume | HVLTR (Years 2-4) | Pearson | 0.144911507 | 0.161174493 | 0.322348987 |
| lh_insula_volume | HVLDR (Years 0-2) | Pearson | 0.144173388 | 0.161084335 | 0.322168669 |
| Right-Thalamus_volume | HVLDR (Years 0-4) | Pearson | 0.142700155 | 0.172399901 | 0.517199704 |
| rh_entorhinal_volume | SF (Years 2-4) | Pearson | -0.141716511 | 0.170711595 | 0.613924875 |
| Right-Thalamus_volume | JLO (Years 0-2) | Pearson | -0.141067155 | 0.168128548 | 0.672514193 |
| Right-Thalamus_volume | HVLDR (Years 2-4) | Pearson | 0.139886106 | 0.178715103 | 0.714860413 |
| rh_insula_volume | SDM (Years 2-4) | Pearson | 0.139867007 | 0.176419055 | 0.423405731 |
| rh_lateralorbitofrontal_volume | UPDRS 2 (Years 0-2) | Pearson | 0.138489439 | 0.176116484 | 0.528349452 |
| lh_insula_volume | LNS (Years 2-4) | Pearson | 0.138086151 | 0.182045719 | 0.364091438 |
| rh_entorhinal_volume | UPDRS 1 (Years 0-4) | Pearson | 0.137036384 | 0.192714766 | 0.938127689 |
| rh_entorhinal_volume | SDM (Years 0-2) | Pearson | 0.136176099 | 0.183519182 | 0.912798171 |
| rh_insula_volume | LNS (Years 0-2) | Pearson | -0.13580283 | 0.184734588 | 0.623771751 |
| Right-Amygdala_volume | SF (Years 0-2) | Pearson | 0.135643625 | 0.185254757 | 0.370509515 |
| Right-Thalamus_volume | JLO (Years 0-4) | Pearson | 0.135152102 | 0.194018105 | 0.332602465 |
| rh_medialorbitofrontal_volume | LNS (Years 0-2) | Pearson | -0.134896703 | 0.187709408 | 0.623771751 |
| lh_lateralorbitofrontal_volume | JLO (Years 2-4) | Pearson | 0.134434595 | 0.196417649 | 0.294626474 |
| Left-Thalamus_volume | UPDRS 1B (Years 0-2) | Pearson | -0.133647653 | 0.206600856 | 0.590942695 |
| rh_lateralorbitofrontal_volume | HVLDR (Years 2-4) | Pearson | 0.13363634 | 0.199112284 | 0.624829475 |
| Left-Amygdala_volume | UPDRS 1 (Years 0-2) | Pearson | -0.13346122 | 0.20469268 | 0.638788684 |
| lh_entorhinal_volume | SF (Years 2-4) | Pearson | -0.131313 | 0.204641625 | 0.613924875 |
| rh_insula_volume | UPDRS 1 (Years 0-2) | Pearson | -0.131088724 | 0.212929561 | 0.638788684 |
| Left-Amygdala_volume | MoCA (Years 2-4) | Pearson | -0.131053518 | 0.208012852 | 0.882492815 |
| Left-Thalamus_volume | UPDRS 2 (Years 0-4) | Pearson | -0.129991494 | 0.204418562 | 0.857646382 |
| rh_medialorbitofrontal_volume | UPDRS 1 (Years 2-4) | Pearson | 0.129737683 | 0.212654852 | 0.998136771 |
| lh_medialorbitofrontal_volume | LNS (Years 0-2) | Pearson | -0.128999316 | 0.207923917 | 0.623771751 |
| Right-Thalamus_volume | UPDRS 1A (Years 2-4) | Pearson | -0.128245859 | 0.304791348 | 0.650429989 |
| Right-Thalamus_volume | SF (Years 0-4) | Pearson | 0.127916005 | 0.216701026 | 0.642219149 |
| Left-Thalamus_volume | MoCA (Years 0-2) | Pearson | 0.127744672 | 0.214853366 | 0.737363039 |
| Right-Thalamus_volume | HVLDR (Years 0-2) | Pearson | 0.126592019 | 0.219054194 | 0.331535556 |
| lh_medialorbitofrontal_volume | MoCA (Years 2-4) | Pearson | -0.126410061 | 0.224721091 | 0.882492815 |
| rh_entorhinal_volume | HVLDR (Years 0-2) | Pearson | 0.126056988 | 0.221023704 | 0.331535556 |
| Left-Thalamus_volume | LNS (Years 0-4) | Pearson | 0.124323908 | 0.229991221 | 0.71339744 |
| rh_entorhinal_volume | UPDRS 1B (Years 2-4) | Pearson | 0.123819134 | 0.234443418 | 0.632931278 |
| rh_insula_volume | UPDRS 1B (Years 2-4) | Pearson | 0.123640952 | 0.235122643 | 0.632931278 |
| rh_lateralorbitofrontal_volume | HVLDR (Years 0-4) | Pearson | 0.123083672 | 0.237255823 | 0.355883734 |
| Left-Thalamus_volume | UPDRS 1A (Years 2-4) | Pearson | -0.122986471 | 0.325214994 | 0.650429989 |
| lh_lateralorbitofrontal_volume | SDM (Years 0-4) | Pearson | 0.122802223 | 0.235789711 | 0.507123664 |
| lh_entorhinal_volume | SDM (Years 2-4) | Pearson | 0.122560572 | 0.23671982 | 0.473439639 |
| lh_insula_volume | UPDRS 1B (Years 0-2) | Pearson | -0.122342781 | 0.247972445 | 0.590942695 |
| rh_lateralorbitofrontal_volume | LNS (Years 0-4) | Pearson | 0.122053781 | 0.238678723 | 0.71339744 |
| lh_entorhinal_volume | HVLDR (Years 2-4) | Pearson | 0.117664514 | 0.258701909 | 0.624829475 |
| Left-Amygdala_volume | HVLDR (Years 2-4) | Pearson | 0.117262076 | 0.260345615 | 0.624829475 |
| lh_insula_volume | UPDRS 1B (Years 2-4) | Pearson | 0.116440962 | 0.263721366 | 0.632931278 |
| Left-Amygdala_volume | GDS (Years 2-4) | Pearson | -0.11578291 | 0.258750393 | 0.514172284 |
| lh_lateralorbitofrontal_volume | LNS (Years 2-4) | Pearson | 0.115665882 | 0.264337264 | 0.453149595 |
| lh_lateralorbitofrontal_volume | UPDRS 2 (Years 0-2) | Pearson | 0.115617332 | 0.25943667 | 0.592876317 |
| Right-Thalamus_volume | SF (Years 0-2) | Pearson | -0.114860575 | 0.26258901 | 0.450152588 |
| rh_medialorbitofrontal_volume | JLO (Years 2-4) | Pearson | 0.114853248 | 0.270332667 | 0.360443556 |
| rh_entorhinal_volume | SF (Years 0-4) | Pearson | -0.114217742 | 0.270404887 | 0.642219149 |
| rh_lateralorbitofrontal_volume | SF (Years 0-4) | Pearson | 0.114185508 | 0.270541003 | 0.642219149 |
| Left-Amygdala_volume | MoCA (Years 0-2) | Pearson | 0.113369249 | 0.271425122 | 0.737363039 |
| lh_insula_volume | UPDRS 2 (Years 0-4) | Pearson | 0.112787427 | 0.271357817 | 0.857646382 |
| Right-Thalamus_volume | UPDRS 2 (Years 0-4) | Pearson | -0.112558072 | 0.272339906 | 0.857646382 |
| rh_medialorbitofrontal_volume | LNS (Years 0-4) | Pearson | -0.109714677 | 0.289869058 | 0.71339744 |
| Left-Thalamus_volume | UPDRS 1B (Years 0-4) | Pearson | -0.109567861 | 0.301192945 | 0.906836681 |
| Left-Thalamus_volume | HVLDR (Years 2-4) | Pearson | 0.109455757 | 0.293638521 | 0.809980495 |
| Right-Amygdala_volume | UPDRS 2 (Years 0-4) | Pearson | 0.109450562 | 0.285882127 | 0.857646382 |
| Right-Thalamus_volume | SDM (Years 0-4) | Pearson | 0.109396928 | 0.291276693 | 0.507123664 |
| Right-Amygdala_volume | MoCA (Years 2-4) | Pearson | -0.109337337 | 0.294164272 | 0.882492815 |
| Left-Amygdala_volume | LNS (Years 0-4) | Pearson | 0.109324381 | 0.291598708 | 0.71339744 |
| Right-Thalamus_volume | UPDRS 1B (Years 0-2) | Pearson | -0.109001249 | 0.303714773 | 0.590942695 |
| lh_entorhinal_volume | HVLDR (Years 0-2) | Pearson | 0.108858032 | 0.291079721 | 0.388106295 |
| rh_medialorbitofrontal_volume | HVLDR (Years 0-4) | Pearson | 0.108094176 | 0.302376904 | 0.718945082 |
| Right-Thalamus_volume | LNS (Years 0-4) | Pearson | 0.108059964 | 0.297248933 | 0.71339744 |
| lh_medialorbitofrontal_volume | MoCA (Years 0-4) | Pearson | -0.107412658 | 0.300169186 | 0.930333349 |
| Left-Thalamus_volume | UPDRS 1 (Years 0-4) | Pearson | -0.107298153 | 0.308659489 | 0.938127689 |
| Left-Thalamus_volume | SDM (Years 0-4) | Pearson | 0.106846736 | 0.302737653 | 0.507123664 |
| lh_lateralorbitofrontal_volume | HVLTR (Years 0-4) | Pearson | 0.106189144 | 0.305740173 | 0.480418041 |
| Left-Thalamus_volume | UPDRS 1 (Years 0-2) | Pearson | -0.106040883 | 0.314395472 | 0.650436829 |
| lh_medialorbitofrontal_volume | SF (Years 0-4) | Pearson | 0.105823049 | 0.307420127 | 0.642219149 |
| Left-Thalamus_volume | MoCA (Years 0-4) | Pearson | 0.104701673 | 0.312603294 | 0.930333349 |
| lh_medialorbitofrontal_volume | UPDRS 2 (Years 2-4) | Pearson | 0.104560904 | 0.315887884 | 0.834882726 |
| Left-Amygdala_volume | JLO (Years 0-2) | Pearson | 0.104184027 | 0.30983963 | 0.765209663 |
| rh_entorhinal_volume | UPDRS 1 (Years 0-2) | Pearson | 0.103708346 | 0.325218415 | 0.650436829 |
| rh_lateralorbitofrontal_volume | HVLTR (Years 0-4) | Pearson | 0.103063004 | 0.320278694 | 0.480418041 |
| Right-Amygdala_volume | SF (Years 0-4) | Pearson | 0.102887139 | 0.321109575 | 0.642219149 |
| Left-Amygdala_volume | GDS (Years 0-4) | Pearson | -0.102421168 | 0.318142195 | 0.704688723 |
| rh_entorhinal_volume | JLO (Years 0-2) | Pearson | 0.102081456 | 0.319758475 | 0.765209663 |
| rh_medialorbitofrontal_volume | UPDRS 2 (Years 0-2) | Pearson | 0.102070071 | 0.31981273 | 0.592876317 |
| Right-Thalamus_volume | GDS (Years 2-4) | Pearson | -0.101742752 | 0.321375208 | 0.514172284 |
| rh_lateralorbitofrontal_volume | HVLDR (Years 0-2) | Pearson | 0.101197358 | 0.326562959 | 0.391875551 |
| rh_lateralorbitofrontal_volume | JLO (Years 2-4) | Pearson | 0.100908778 | 0.33317943 | 0.399815316 |
| rh_insula_volume | GDS (Years 0-4) | Pearson | -0.100226425 | 0.328677366 | 0.704688723 |
| Left-Amygdala_volume | UPDRS 1B (Years 0-2) | Pearson | -0.100186537 | 0.344716572 | 0.590942695 |
| lh_entorhinal_volume | UPDRS 1A (Years 0-2) | Pearson | 0.100088704 | 0.458829835 | 0.987481291 |
| Left-Amygdala_volume | HVLTR (Years 0-2) | Pearson | 0.099716673 | 0.331155757 | 0.640903895 |
| rh_entorhinal_volume | SDM (Years 0-4) | Pearson | 0.099356563 | 0.338082443 | 0.507123664 |
| Right-Amygdala_volume | HVLDR (Years 2-4) | Pearson | -0.099017909 | 0.342363665 | 0.809980495 |
| Right-Amygdala_volume | GDS (Years 2-4) | Pearson | -0.097357267 | 0.342781523 | 0.514172284 |
| Left-Thalamus_volume | UPDRS 2 (Years 0-2) | Pearson | -0.096744071 | 0.345844518 | 0.592876317 |
| lh_insula_volume | MoCA (Years 0-2) | Pearson | -0.096468517 | 0.349790349 | 0.737363039 |
| lh_entorhinal_volume | LNS (Years 2-4) | Pearson | 0.096236468 | 0.353544899 | 0.530317349 |
| lh_medialorbitofrontal_volume | HVLDR (Years 2-4) | Pearson | 0.096104336 | 0.356823744 | 0.636798585 |
| Right-Thalamus_volume | UPDRS 1A (Years 0-2) | Pearson | -0.095866856 | 0.478088546 | 0.987481291 |
| lh_entorhinal_volume | UPDRS 1B (Years 2-4) | Pearson | 0.095657353 | 0.359075132 | 0.701604466 |
| lh_medialorbitofrontal_volume | UPDRS 1B (Years 0-4) | Pearson | -0.095538659 | 0.367670829 | 0.906836681 |
| rh_lateralorbitofrontal_volume | HVLTR (Years 2-4) | Pearson | 0.094396152 | 0.362867947 | 0.622059338 |
| lh_entorhinal_volume | LNS (Years 0-4) | Pearson | 0.093698149 | 0.366443271 | 0.725472202 |
| Right-Thalamus_volume | HVLDR (Years 0-2) | Pearson | 0.093269116 | 0.366075837 | 0.970213818 |
| rh_insula_volume | HVLDR (Years 2-4) | Pearson | 0.093227722 | 0.371465841 | 0.636798585 |
| Right-Amygdala_volume | MoCA (Years 0-2) | Pearson | 0.092765413 | 0.368681519 | 0.737363039 |
| rh_lateralorbitofrontal_volume | HVLDR (Years 0-4) | Pearson | 0.092653229 | 0.377056396 | 0.718945082 |
| lh_medialorbitofrontal_volume | HVLDR (Years 0-4) | Pearson | 0.091678709 | 0.379500029 | 0.506000039 |
| rh_entorhinal_volume | GDS (Years 0-4) | Pearson | -0.091384409 | 0.373342938 | 0.704688723 |
| lh_insula_volume | HVLTR (Years 0-2) | Pearson | 0.09128588 | 0.373860605 | 0.640903895 |
| rh_insula_volume | JLO (Years 0-2) | Pearson | -0.089633932 | 0.382604832 | 0.765209663 |
| lh_insula_volume | UPDRS 1A (Years 0-4) | Pearson | 0.087862036 | 0.515752691 | 0.916694025 |
| Left-Thalamus_volume | GDS (Years 2-4) | Pearson | -0.087336718 | 0.394967672 | 0.526623563 |
| lh_medialorbitofrontal_volume | UPDRS 1B (Years 2-4) | Pearson | 0.086104694 | 0.409269272 | 0.701604466 |
| rh_medialorbitofrontal_volume | SF (Years 2-4) | Pearson | -0.085801553 | 0.408381499 | 0.877233459 |
| rh_lateralorbitofrontal_volume | MoCA (Years 2-4) | Pearson | -0.085712332 | 0.411414984 | 0.975584792 |
| lh_medialorbitofrontal_volume | JLO (Years 0-4) | Pearson | 0.084383137 | 0.418732595 | 0.545250901 |
| lh_entorhinal_volume | HVLDR (Years 2-4) | Pearson | -0.0842689 | 0.419364999 | 0.809980495 |
| lh_lateralorbitofrontal_volume | MoCA (Years 0-4) | Pearson | 0.083356858 | 0.421913693 | 0.930333349 |
| lh_entorhinal_volume | UPDRS 2 (Years 2-4) | Pearson | 0.083286791 | 0.424824592 | 0.834882726 |
| lh_medialorbitofrontal_volume | UPDRS 2 (Years 0-4) | Pearson | -0.082259147 | 0.423123074 | 0.866192294 |
| lh_medialorbitofrontal_volume | HVLTR (Years 0-4) | Pearson | 0.081353752 | 0.433191799 | 0.577589065 |
| rh_insula_volume | UPDRS 2 (Years 0-4) | Pearson | 0.080507801 | 0.433096147 | 0.866192294 |
| lh_lateralorbitofrontal_volume | SDM (Years 2-4) | Pearson | 0.079405493 | 0.444323823 | 0.55451286 |
| rh_entorhinal_volume | UPDRS 2 (Years 2-4) | Pearson | 0.078214008 | 0.453666951 | 0.834882726 |
| lh_insula_volume | UPDRS 1A (Years 2-4) | Pearson | 0.078083669 | 0.533161681 | 0.847733624 |
| rh_lateralorbitofrontal_volume | UPDRS 1 (Years 0-2) | Pearson | -0.07784628 | 0.460765708 | 0.668743053 |
| rh_insula_volume | SF (Years 0-2) | Pearson | 0.076729384 | 0.455062097 | 0.647218 |
| Left-Amygdala_volume | HVLDR (Years 0-4) | Pearson | 0.076710186 | 0.464877514 | 0.718945082 |
| rh_medialorbitofrontal_volume | SDM (Years 2-4) | Pearson | 0.076434547 | 0.461604499 | 0.55451286 |
| lh_medialorbitofrontal_volume | HVLDR (Years 0-2) | Pearson | -0.076208513 | 0.460526118 | 0.970213818 |
| Right-Amygdala_volume | LNS (Years 0-4) | Pearson | 0.075442126 | 0.467458169 | 0.725472202 |
| lh_entorhinal_volume | UPDRS 1B (Years 0-4) | Pearson | 0.07486474 | 0.480634613 | 0.906836681 |
| Left-Thalamus_volume | GDS (Years 0-4) | Pearson | -0.074637741 | 0.467482488 | 0.704688723 |
| Left-Thalamus_volume | UPDRS 2 (Years 2-4) | Pearson | -0.074300438 | 0.476640909 | 0.834882726 |
| rh_insula_volume | HVLDR (Years 0-4) | Pearson | 0.074260478 | 0.479296721 | 0.718945082 |
| rh_lateralorbitofrontal_volume | SDM (Years 2-4) | Pearson | 0.074215451 | 0.474749157 | 0.55451286 |
| lh_insula_volume | UPDRS 1 (Years 0-2) | Pearson | -0.074006919 | 0.483236744 | 0.668743053 |
| lh_lateralorbitofrontal_volume | SF (Years 2-4) | Pearson | 0.073503806 | 0.479006945 | 0.877233459 |
| rh_lateralorbitofrontal_volume | GDS (Years 0-4) | Pearson | -0.073369109 | 0.475104908 | 0.704688723 |
| lh_lateralorbitofrontal_volume | HVLDR (Years 2-4) | Pearson | 0.073187446 | 0.483287139 | 0.669647163 |
| lh_insula_volume | SDM (Years 2-4) | Pearson | 0.072982833 | 0.482136897 | 0.55451286 |
| rh_medialorbitofrontal_volume | SF (Years 0-4) | Pearson | 0.072079682 | 0.487588747 | 0.747864556 |
| lh_lateralorbitofrontal_volume | SDM (Years 0-2) | Pearson | 0.071792104 | 0.484672979 | 0.912798171 |
| Left-Amygdala_volume | SF (Years 0-2) | Pearson | -0.071670749 | 0.4854135 | 0.647218 |
| Right-Thalamus_volume | UPDRS 2 (Years 2-4) | Pearson | -0.071567962 | 0.493045595 | 0.834882726 |
| Left-Amygdala_volume | SDM (Years 0-2) | Pearson | -0.071547705 | 0.486164947 | 0.912798171 |
| rh_insula_volume | JLO (Years 0-4) | Pearson | 0.071545116 | 0.493184 | 0.545250901 |
| lh_insula_volume | JLO (Years 0-4) | Pearson | 0.071104085 | 0.495859768 | 0.545250901 |
| lh_medialorbitofrontal_volume | UPDRS 1 (Years 0-4) | Pearson | -0.070804811 | 0.502416258 | 0.938127689 |
| Left-Thalamus_volume | JLO (Years 0-4) | Pearson | 0.070454723 | 0.499813326 | 0.545250901 |
| rh_lateralorbitofrontal_volume | GDS (Years 2-4) | Pearson | -0.070345976 | 0.493536491 | 0.592243789 |
| Left-Amygdala_volume | UPDRS 1B (Years 2-4) | Pearson | 0.069980859 | 0.502708734 | 0.754063101 |
| lh_insula_volume | HVLDR (Years 2-4) | Pearson | 0.069757666 | 0.504075509 | 0.809980495 |
| lh_medialorbitofrontal_volume | UPDRS 1A (Years 2-4) | Pearson | 0.06972588 | 0.577998644 | 0.847733624 |
| lh_lateralorbitofrontal_volume | MoCA (Years 0-2) | Pearson | 0.069722315 | 0.499669564 | 0.856576395 |
| Right-Amygdala_volume | SDM (Years 2-4) | Pearson | 0.068695762 | 0.508303455 | 0.55451286 |
| Right-Amygdala_volume | UPDRS 1 (Years 2-4) | Pearson | 0.068530591 | 0.511624081 | 0.998136771 |
| Right-Thalamus_volume | GDS (Years 0-4) | Pearson | -0.067572133 | 0.510773884 | 0.704688723 |
| Right-Thalamus_volume | UPDRS 1B (Years 0-4) | Pearson | -0.066255141 | 0.532638883 | 0.906836681 |
| Left-Thalamus_volume | SDM (Years 0-2) | Pearson | -0.06572069 | 0.522449142 | 0.912798171 |
| Left-Thalamus_volume | HVLTR (Years 2-4) | Pearson | 0.065644578 | 0.527363151 | 0.791044726 |
| lh_lateralorbitofrontal_volume | LNS (Years 0-4) | Pearson | 0.065215404 | 0.530072529 | 0.725472202 |
| Right-Thalamus_volume | UPDRS 1 (Years 0-4) | Pearson | -0.064493962 | 0.541343401 | 0.938127689 |
| Right-Amygdala_volume | SDM (Years 0-4) | Pearson | 0.064207316 | 0.536463836 | 0.596780513 |
| lh_lateralorbitofrontal_volume | HVLDR (Years 2-4) | Pearson | 0.064000331 | 0.539986997 | 0.809980495 |
| rh_entorhinal_volume | JLO (Years 2-4) | Pearson | 0.063803047 | 0.541239499 | 0.590443089 |
| rh_lateralorbitofrontal_volume | MoCA (Years 0-4) | Pearson | 0.063687179 | 0.539776378 | 0.930333349 |
| Right-Thalamus_volume | HVLDR (Years 2-4) | Pearson | 0.063404965 | 0.543771154 | 0.669647163 |
| lh_insula_volume | MoCA (Years 0-4) | Pearson | -0.063230281 | 0.542694454 | 0.930333349 |
| rh_medialorbitofrontal_volume | UPDRS 1B (Years 0-2) | Pearson | -0.06309204 | 0.552423789 | 0.729525975 |
| rh_insula_volume | LNS (Years 0-4) | Pearson | 0.06300999 | 0.544104151 | 0.725472202 |
| Right-Thalamus_volume | UPDRS 1 (Years 0-2) | Pearson | -0.06241413 | 0.554489075 | 0.668743053 |
| lh_entorhinal_volume | MoCA (Years 2-4) | Pearson | -0.062219083 | 0.551347091 | 0.975584792 |
| lh_insula_volume | SF (Years 0-4) | Pearson | 0.062163724 | 0.549536208 | 0.747864556 |
| lh_lateralorbitofrontal_volume | UPDRS 1A (Years 0-2) | Pearson | -0.061458387 | 0.649720417 | 0.987481291 |
| lh_lateralorbitofrontal_volume | UPDRS 2 (Years 2-4) | Pearson | 0.061403245 | 0.556588484 | 0.834882726 |
| lh_entorhinal_volume | UPDRS 1A (Years 0-4) | Pearson | 0.061325814 | 0.65042697 | 0.916694025 |
| rh_medialorbitofrontal_volume | HVLDR (Years 2-4) | Pearson | -0.061178074 | 0.558039302 | 0.669647163 |
| lh_entorhinal_volume | SF (Years 0-2) | Pearson | 0.060163195 | 0.558286899 | 0.669944279 |
| rh_lateralorbitofrontal_volume | UPDRS 1 (Years 2-4) | Pearson | 0.059922331 | 0.566163293 | 0.998136771 |
| lh_lateralorbitofrontal_volume | UPDRS 1 (Years 0-2) | Pearson | -0.059625543 | 0.572351782 | 0.668743053 |
| rh_medialorbitofrontal_volume | SDM (Years 0-2) | Pearson | 0.059108902 | 0.565215876 | 0.912798171 |
| lh_lateralorbitofrontal_volume | HVLDR (Years 0-4) | Pearson | 0.058968891 | 0.574475014 | 0.765966685 |
| rh_medialorbitofrontal_volume | UPDRS 1 (Years 0-4) | Pearson | 0.058944106 | 0.576757328 | 0.938127689 |
| lh_entorhinal_volume | SF (Years 0-4) | Pearson | -0.058724615 | 0.571878109 | 0.747864556 |
| rh_lateralorbitofrontal_volume | UPDRS 1A (Years 0-4) | Pearson | -0.058705532 | 0.664453874 | 0.916694025 |
| Left-Amygdala_volume | LNS (Years 0-2) | Pearson | -0.058313069 | 0.570473017 | 0.995105932 |
| rh_insula_volume | MoCA (Years 0-2) | Pearson | -0.058015738 | 0.57448206 | 0.861723089 |
| lh_insula_volume | UPDRS 1 (Years 2-4) | Pearson | 0.057968266 | 0.578914939 | 0.998136771 |
| lh_lateralorbitofrontal_volume | GDS (Years 0-4) | Pearson | -0.057908065 | 0.573157174 | 0.704688723 |
| lh_entorhinal_volume | GDS (Years 0-4) | Pearson | 0.057208853 | 0.577805019 | 0.704688723 |
| Left-Amygdala_volume | SDM (Years 0-4) | Pearson | 0.056822411 | 0.584415315 | 0.596780513 |
| lh_entorhinal_volume | SDM (Years 0-4) | Pearson | 0.056235555 | 0.588308449 | 0.596780513 |
| rh_lateralorbitofrontal_volume | UPDRS 1B (Years 0-2) | Pearson | -0.056004832 | 0.597998643 | 0.729525975 |
| lh_medialorbitofrontal_volume | GDS (Years 0-4) | Pearson | 0.055797328 | 0.587240602 | 0.704688723 |
| Right-Thalamus_volume | LNS (Years 2-4) | Pearson | 0.055695549 | 0.591901165 | 0.789201554 |
| lh_insula_volume | SDM (Years 0-4) | Pearson | 0.054964518 | 0.596780513 | 0.596780513 |
| lh_entorhinal_volume | HVLTR (Years 0-4) | Pearson | 0.054871425 | 0.597403165 | 0.716883798 |
| lh_lateralorbitofrontal_volume | UPDRS 1B (Years 0-2) | Pearson | -0.054492271 | 0.607938312 | 0.729525975 |
| rh_entorhinal_volume | HVLDR (Years 0-4) | Pearson | -0.054256978 | 0.603492049 | 0.724190459 |
| lh_entorhinal_volume | UPDRS 2 (Years 0-2) | Pearson | 0.054158285 | 0.598284283 | 0.897426425 |
| rh_medialorbitofrontal_volume | UPDRS 1A (Years 2-4) | Pearson | 0.05333169 | 0.670622889 | 0.847733624 |
| rh_insula_volume | UPDRS 1 (Years 2-4) | Pearson | 0.053161499 | 0.610833783 | 0.998136771 |
| lh_medialorbitofrontal_volume | GDS (Years 2-4) | Pearson | 0.05306173 | 0.605724132 | 0.637685597 |
| lh_entorhinal_volume | UPDRS 1 (Years 0-2) | Pearson | 0.052654272 | 0.618143798 | 0.668743053 |
| rh_medialorbitofrontal_volume | HVLTR (Years 2-4) | Pearson | 0.050329412 | 0.628127881 | 0.837503842 |
| lh_medialorbitofrontal_volume | HVLDR (Years 0-2) | Pearson | -0.049701266 | 0.63059521 | 0.684471986 |
| rh_medialorbitofrontal_volume | UPDRS 2 (Years 0-4) | Pearson | 0.048871975 | 0.634517909 | 0.984909257 |
| lh_lateralorbitofrontal_volume | UPDRS 1 (Years 2-4) | Pearson | -0.048505424 | 0.642462425 | 0.998136771 |
| lh_medialorbitofrontal_volume | JLO (Years 0-2) | Pearson | -0.048488005 | 0.637185034 | 0.884559363 |
| rh_lateralorbitofrontal_volume | HVLDR (Years 2-4) | Pearson | 0.048485202 | 0.642601254 | 0.856801672 |
| rh_medialorbitofrontal_volume | GDS (Years 2-4) | Pearson | 0.048416016 | 0.637685597 | 0.637685597 |
| rh_entorhinal_volume | LNS (Years 0-4) | Pearson | 0.047591139 | 0.646966817 | 0.739083392 |
| Right-Amygdala_volume | UPDRS 1A (Years 2-4) | Pearson | 0.047237245 | 0.706444687 | 0.847733624 |
| rh_insula_volume | HVLDR (Years 0-2) | Pearson | -0.046860508 | 0.65028085 | 0.970213818 |
| Left-Amygdala_volume | HVLDR (Years 0-2) | Pearson | 0.045810539 | 0.657619 | 0.970213818 |
| rh_medialorbitofrontal_volume | UPDRS 1 (Years 0-2) | Pearson | -0.045204166 | 0.668743053 | 0.668743053 |
| lh_entorhinal_volume | HVLTR (Years 0-2) | Pearson | 0.044913561 | 0.662232184 | 0.940998094 |
| lh_lateralorbitofrontal_volume | HVLDR (Years 0-2) | Pearson | 0.044515281 | 0.666716319 | 0.970213818 |
| Left-Amygdala_volume | SF (Years 2-4) | Pearson | 0.044508308 | 0.668445278 | 0.877233459 |
| rh_insula_volume | SDM (Years 0-2) | Pearson | 0.044046595 | 0.668365075 | 0.912798171 |
| lh_medialorbitofrontal_volume | SF (Years 2-4) | Pearson | -0.043332795 | 0.676707272 | 0.877233459 |
| Left-Thalamus_volume | SF (Years 0-4) | Pearson | 0.043181668 | 0.67777226 | 0.747864556 |
| rh_insula_volume | GDS (Years 0-2) | Pearson | 0.043152291 | 0.674714267 | 0.973638175 |
| Right-Thalamus_volume | UPDRS 2 (Years 0-2) | Pearson | -0.042945017 | 0.676189105 | 0.901585474 |
| Left-Thalamus_volume | LNS (Years 0-2) | Pearson | 0.042937716 | 0.676241074 | 0.995105932 |
| lh_lateralorbitofrontal_volume | UPDRS 1B (Years 2-4) | Pearson | -0.042490737 | 0.684276326 | 0.912368434 |
| Right-Thalamus_volume | SDM (Years 0-2) | Pearson | -0.042139526 | 0.681932073 | 0.912798171 |
| rh_insula_volume | SF (Years 0-4) | Pearson | 0.042081728 | 0.68554251 | 0.747864556 |
| rh_medialorbitofrontal_volume | HVLDR (Years 0-2) | Pearson | 0.042006747 | 0.684471986 | 0.684471986 |
| lh_insula_volume | SF (Years 0-2) | Pearson | 0.041804203 | 0.684328231 | 0.746539888 |
| rh_medialorbitofrontal_volume | HVLTR (Years 0-4) | Pearson | 0.041775825 | 0.68770936 | 0.728429665 |
| rh_entorhinal_volume | UPDRS 1B (Years 0-2) | Pearson | 0.041394382 | 0.69684303 | 0.760192396 |
| Left-Thalamus_volume | UPDRS 1A (Years 0-2) | Pearson | -0.040948553 | 0.762322409 | 0.987481291 |
| rh_medialorbitofrontal_volume | UPDRS 1B (Years 0-4) | Pearson | 0.040431962 | 0.703559318 | 0.906836681 |
| rh_lateralorbitofrontal_volume | HVLTR (Years 0-2) | Pearson | 0.038825488 | 0.705748571 | 0.940998094 |
| rh_insula_volume | UPDRS 1A (Years 0-4) | Pearson | 0.038668027 | 0.775202693 | 0.916694025 |
| lh_entorhinal_volume | HVLTR (Years 2-4) | Pearson | 0.038328769 | 0.712297221 | 0.854756666 |
| rh_entorhinal_volume | UPDRS 2 (Years 0-4) | Pearson | 0.036718337 | 0.721042374 | 0.984909257 |
| rh_entorhinal_volume | HVLTR (Years 0-4) | Pearson | -0.036089682 | 0.728429665 | 0.728429665 |
| rh_medialorbitofrontal_volume | JLO (Years 0-4) | Pearson | 0.036056984 | 0.730079166 | 0.730079166 |
| Right-Amygdala_volume | UPDRS 1 (Years 0-4) | Pearson | 0.036023352 | 0.733171105 | 0.938127689 |
| lh_insula_volume | GDS (Years 0-2) | Pearson | -0.035999443 | 0.726285716 | 0.973638175 |
| lh_insula_volume | LNS (Years 0-4) | Pearson | -0.035904096 | 0.729772233 | 0.739083392 |
| Left-Thalamus_volume | UPDRS 1A (Years 0-4) | Pearson | 0.035568237 | 0.792806389 | 0.916694025 |
| lh_insula_volume | JLO (Years 0-2) | Pearson | -0.035496756 | 0.729959661 | 0.884559363 |
| lh_medialorbitofrontal_volume | UPDRS 1A (Years 0-2) | Pearson | 0.035110313 | 0.795415899 | 0.987481291 |
| lh_medialorbitofrontal_volume | LNS (Years 0-4) | Pearson | -0.034620059 | 0.739083392 | 0.739083392 |
| lh_entorhinal_volume | UPDRS 2 (Years 0-4) | Pearson | 0.034306644 | 0.738681943 | 0.984909257 |
| Left-Amygdala_volume | GDS (Years 0-2) | Pearson | -0.033295786 | 0.74611661 | 0.973638175 |
| Left-Amygdala_volume | UPDRS 1 (Years 0-4) | Pearson | 0.032867299 | 0.755781029 | 0.938127689 |
| lh_entorhinal_volume | UPDRS 1 (Years 2-4) | Pearson | -0.032814505 | 0.753539118 | 0.998136771 |
| rh_medialorbitofrontal_volume | JLO (Years 0-2) | Pearson | -0.032624621 | 0.751065827 | 0.884559363 |
| lh_insula_volume | SDM (Years 0-2) | Pearson | -0.03261591 | 0.751130127 | 0.912798171 |
| Right-Amygdala_volume | UPDRS 1B (Years 0-4) | Pearson | -0.032388914 | 0.760534207 | 0.906836681 |
| rh_lateralorbitofrontal_volume | JLO (Years 0-2) | Pearson | 0.032256521 | 0.753784521 | 0.884559363 |
| lh_entorhinal_volume | JLO (Years 2-4) | Pearson | -0.032099199 | 0.75874563 | 0.75874563 |
| lh_entorhinal_volume | SDM (Years 0-2) | Pearson | -0.031326718 | 0.760665143 | 0.912798171 |
| lh_insula_volume | UPDRS 1B (Years 0-4) | Pearson | -0.031281463 | 0.76848814 | 0.906836681 |
| lh_lateralorbitofrontal_volume | MoCA (Years 2-4) | Pearson | 0.031181587 | 0.765440413 | 0.975584792 |
| rh_medialorbitofrontal_volume | UPDRS 2 (Years 2-4) | Pearson | 0.031119351 | 0.765895116 | 0.961562933 |
| Left-Thalamus_volume | UPDRS 1B (Years 2-4) | Pearson | 0.030690506 | 0.769030414 | 0.922836497 |
| rh_insula_volume | SF (Years 2-4) | Pearson | -0.030387891 | 0.770033497 | 0.877233459 |
| Left-Amygdala_volume | UPDRS 1A (Years 2-4) | Pearson | -0.030259769 | 0.809408083 | 0.882990636 |
| rh_insula_volume | MoCA (Years 2-4) | Pearson | 0.02990657 | 0.774771375 | 0.975584792 |
| lh_medialorbitofrontal_volume | UPDRS 1 (Years 2-4) | Pearson | 0.029857531 | 0.775130908 | 0.998136771 |
| rh_lateralorbitofrontal_volume | GDS (Years 0-2) | Pearson | -0.028766888 | 0.779703075 | 0.973638175 |
| Right-Amygdala_volume | GDS (Years 0-2) | Pearson | 0.028667155 | 0.780447525 | 0.973638175 |
| rh_entorhinal_volume | UPDRS 1A (Years 0-2) | Pearson | -0.028545423 | 0.833057768 | 0.987481291 |
| lh_insula_volume | SF (Years 2-4) | Pearson | 0.028156051 | 0.786504903 | 0.877233459 |
| rh_medialorbitofrontal_volume | UPDRS 1A (Years 0-4) | Pearson | -0.02807955 | 0.83574426 | 0.916694025 |
| Left-Amygdala_volume | UPDRS 2 (Years 0-2) | Pearson | -0.02735759 | 0.790240857 | 0.948289028 |
| Right-Amygdala_volume | LNS (Years 0-2) | Pearson | -0.026634889 | 0.795659539 | 0.995105932 |
| lh_entorhinal_volume | HVLDR (Years 0-4) | Pearson | 0.025667464 | 0.806018153 | 0.86513631 |
| Right-Thalamus_volume | UPDRS 1A (Years 0-4) | Pearson | -0.02485052 | 0.854414345 | 0.916694025 |
| Right-Amygdala_volume | JLO (Years 0-2) | Pearson | -0.024616188 | 0.810846083 | 0.884559363 |
| lh_entorhinal_volume | MoCA (Years 0-2) | Pearson | 0.024458432 | 0.81301454 | 0.981148407 |
| lh_entorhinal_volume | MoCA (Years 0-4) | Pearson | -0.024355218 | 0.814769572 | 0.997989383 |
| Right-Amygdala_volume | GDS (Years 0-4) | Pearson | -0.023656543 | 0.818090449 | 0.892462308 |
| lh_insula_volume | MoCA (Years 2-4) | Pearson | 0.023620865 | 0.821216885 | 0.975584792 |
| rh_entorhinal_volume | MoCA (Years 0-2) | Pearson | 0.023543935 | 0.819885513 | 0.981148407 |
| lh_insula_volume | HVLDR (Years 0-2) | Pearson | -0.023435717 | 0.820699511 | 0.970213818 |
| rh_lateralorbitofrontal_volume | SF (Years 2-4) | Pearson | -0.023385873 | 0.822017741 | 0.877233459 |
| lh_lateralorbitofrontal_volume | UPDRS 1A (Years 0-4) | Pearson | -0.022952595 | 0.865425846 | 0.916694025 |
| rh_lateralorbitofrontal_volume | UPDRS 1B (Years 0-4) | Pearson | 0.02258594 | 0.831713245 | 0.906836681 |
| Right-Thalamus_volume | MoCA (Years 2-4) | Pearson | 0.022280619 | 0.831206267 | 0.975584792 |
| rh_insula_volume | UPDRS 1B (Years 0-4) | Pearson | -0.022219189 | 0.834406654 | 0.906836681 |
| rh_entorhinal_volume | HVLTR (Years 0-2) | Pearson | -0.021686485 | 0.833009771 | 0.946781316 |
| rh_medialorbitofrontal_volume | UPDRS 1A (Years 0-2) | Pearson | 0.021603071 | 0.873271213 | 0.987481291 |
| lh_medialorbitofrontal_volume | SDM (Years 0-2) | Pearson | -0.020787452 | 0.839838238 | 0.916187168 |
| rh_entorhinal_volume | UPDRS 1A (Years 0-4) | Pearson | -0.020481348 | 0.879801537 | 0.916694025 |
| rh_lateralorbitofrontal_volume | UPDRS 2 (Years 2-4) | Pearson | 0.020376226 | 0.845446349 | 0.961562933 |
| lh_medialorbitofrontal_volume | HVLDR (Years 0-4) | Pearson | 0.01996654 | 0.849336504 | 0.997365706 |
| Left-Amygdala_volume | UPDRS 1A (Years 0-4) | Pearson | 0.018437683 | 0.891719293 | 0.916694025 |
| rh_insula_volume | HVLDR (Years 2-4) | Pearson | 0.018291553 | 0.861091449 | 0.946814862 |
| rh_lateralorbitofrontal_volume | LNS (Years 0-2) | Pearson | -0.018221848 | 0.859388555 | 0.995105932 |
| lh_entorhinal_volume | UPDRS 1 (Years 0-4) | Pearson | 0.017975229 | 0.864956085 | 0.938127689 |
| Left-Thalamus_volume | GDS (Years 0-2) | Pearson | -0.017972098 | 0.861296422 | 0.973638175 |
| rh_medialorbitofrontal_volume | HVLDR (Years 0-4) | Pearson | 0.01775378 | 0.86513631 | 0.86513631 |
| rh_entorhinal_volume | SDM (Years 2-4) | Pearson | 0.017689597 | 0.864894009 | 0.864894009 |
| Left-Amygdala_volume | HVLDR (Years 2-4) | Pearson | 0.017384798 | 0.867913624 | 0.946814862 |
| rh_entorhinal_volume | LNS (Years 0-2) | Pearson | 0.016696986 | 0.871049362 | 0.995105932 |
| Right-Amygdala_volume | SF (Years 2-4) | Pearson | -0.016060624 | 0.877233459 | 0.877233459 |
| lh_medialorbitofrontal_volume | UPDRS 1A (Years 0-4) | Pearson | 0.015777259 | 0.907269021 | 0.916694025 |
| Right-Thalamus_volume | LNS (Years 0-2) | Pearson | 0.015777051 | 0.878097754 | 0.995105932 |
| rh_entorhinal_volume | SF (Years 0-2) | Pearson | -0.015389769 | 0.881067927 | 0.881067927 |
| Left-Thalamus_volume | HVLDR (Years 2-4) | Pearson | 0.015162594 | 0.884672835 | 0.949951556 |
| rh_insula_volume | UPDRS 1 (Years 0-4) | Pearson | -0.014728942 | 0.889172801 | 0.938127689 |
| rh_insula_volume | MoCA (Years 0-4) | Pearson | -0.014713513 | 0.887460158 | 0.997989383 |
| lh_lateralorbitofrontal_volume | UPDRS 1B (Years 0-4) | Pearson | 0.014643786 | 0.890422914 | 0.906836681 |
| Right-Amygdala_volume | UPDRS 1A (Years 0-4) | Pearson | 0.014167663 | 0.916694025 | 0.916694025 |
| rh_entorhinal_volume | MoCA (Years 0-4) | Pearson | 0.013882736 | 0.893776383 | 0.997989383 |
| rh_entorhinal_volume | UPDRS 1 (Years 2-4) | Pearson | 0.01368153 | 0.895871304 | 0.998136771 |
| rh_entorhinal_volume | HVLDR (Years 0-2) | Pearson | 0.013555638 | 0.895709063 | 0.970213818 |
| Left-Thalamus_volume | UPDRS 1 (Years 2-4) | Pearson | 0.0134959 | 0.897276357 | 0.998136771 |
| rh_medialorbitofrontal_volume | MoCA (Years 0-2) | Pearson | 0.013075147 | 0.89938604 | 0.981148407 |
| Left-Thalamus_volume | LNS (Years 2-4) | Pearson | 0.012675157 | 0.90296907 | 0.999824978 |
| Left-Amygdala_volume | UPDRS 1B (Years 0-4) | Pearson | 0.012439514 | 0.906836681 | 0.906836681 |
| Right-Thalamus_volume | GDS (Years 0-2) | Pearson | 0.011976514 | 0.907311749 | 0.973638175 |
| lh_insula_volume | UPDRS 1A (Years 0-2) | Pearson | -0.011933789 | 0.929793111 | 0.987481291 |
| rh_entorhinal_volume | HVLDR (Years 0-4) | Pearson | 0.010057828 | 0.923770942 | 0.997365706 |
| rh_lateralorbitofrontal_volume | UPDRS 1 (Years 0-4) | Pearson | -0.009905583 | 0.925336153 | 0.938127689 |
| Right-Amygdala_volume | SDM (Years 0-2) | Pearson | -0.009139551 | 0.92920186 | 0.92920186 |
| rh_entorhinal_volume | GDS (Years 0-2) | Pearson | 0.008998515 | 0.930291616 | 0.973638175 |
| Left-Thalamus_volume | MoCA (Years 2-4) | Pearson | -0.008616658 | 0.934308494 | 0.975584792 |
| lh_lateralorbitofrontal_volume | UPDRS 1 (Years 0-4) | Pearson | 0.008275285 | 0.937597516 | 0.938127689 |
| lh_insula_volume | UPDRS 1 (Years 0-4) | Pearson | 0.008204838 | 0.938127689 | 0.938127689 |
| lh_lateralorbitofrontal_volume | HVLTR (Years 0-2) | Pearson | -0.007378855 | 0.942815073 | 0.946781316 |
| lh_entorhinal_volume | HVLDR (Years 0-2) | Pearson | 0.007292326 | 0.943783855 | 0.970213818 |
| Right-Amygdala_volume | UPDRS 1A (Years 0-2) | Pearson | 0.007219949 | 0.957490925 | 0.987481291 |
| lh_lateralorbitofrontal_volume | LNS (Years 0-2) | Pearson | -0.007188809 | 0.944285507 | 0.995105932 |
| lh_medialorbitofrontal_volume | UPDRS 2 (Years 0-2) | Pearson | 0.007049819 | 0.945361026 | 0.994737328 |
| rh_medialorbitofrontal_volume | HVLTR (Years 0-2) | Pearson | 0.006866294 | 0.946781316 | 0.946781316 |
| Right-Thalamus_volume | UPDRS 1B (Years 2-4) | Pearson | -0.006851338 | 0.94774552 | 0.965526303 |
| rh_insula_volume | UPDRS 2 (Years 2-4) | Pearson | -0.006691874 | 0.948960083 | 0.961562933 |
| rh_entorhinal_volume | HVLDR (Years 2-4) | Pearson | 0.006561712 | 0.949951556 | 0.949951556 |
| lh_entorhinal_volume | UPDRS 1B (Years 0-2) | Pearson | 0.006413791 | 0.951886053 | 0.951886053 |
| rh_entorhinal_volume | HVLTR (Years 2-4) | Pearson | -0.00616885 | 0.95268839 | 0.963994693 |
| Left-Amygdala_volume | UPDRS 1A (Years 0-2) | Pearson | 0.006126005 | 0.963927118 | 0.987481291 |
| lh_lateralorbitofrontal_volume | GDS (Years 0-2) | Pearson | -0.005827896 | 0.954820419 | 0.973638175 |
| lh_insula_volume | UPDRS 2 (Years 2-4) | Pearson | -0.005038041 | 0.961562933 | 0.961562933 |
| rh_medialorbitofrontal_volume | GDS (Years 0-4) | Pearson | 0.004959394 | 0.961547725 | 0.961547725 |
| Right-Amygdala_volume | HVLDR (Years 0-2) | Pearson | 0.004878655 | 0.962373775 | 0.970213818 |
| Right-Thalamus_volume | HVLTR (Years 2-4) | Pearson | 0.004693507 | 0.963994693 | 0.963994693 |
| Left-Amygdala_volume | UPDRS 1 (Years 2-4) | Pearson | -0.004571268 | 0.965121771 | 0.998136771 |
| rh_lateralorbitofrontal_volume | UPDRS 1B (Years 2-4) | Pearson | -0.004518216 | 0.965526303 | 0.965526303 |
| Right-Amygdala_volume | MoCA (Years 0-4) | Pearson | -0.004516413 | 0.96535238 | 0.997989383 |
| Left-Amygdala_volume | UPDRS 2 (Years 0-4) | Pearson | -0.003917978 | 0.969617906 | 0.987346646 |
| rh_lateralorbitofrontal_volume | HVLDR (Years 0-2) | Pearson | 0.003861581 | 0.970213818 | 0.970213818 |
| rh_medialorbitofrontal_volume | GDS (Years 0-2) | Pearson | 0.003505559 | 0.972814736 | 0.973638175 |
| lh_medialorbitofrontal_volume | GDS (Years 0-2) | Pearson | 0.003399337 | 0.973638175 | 0.973638175 |
| rh_entorhinal_volume | MoCA (Years 2-4) | Pearson | 0.003199435 | 0.975584792 | 0.975584792 |
| lh_lateralorbitofrontal_volume | UPDRS 2 (Years 0-4) | Pearson | 0.002350407 | 0.981770917 | 0.987346646 |
| rh_insula_volume | UPDRS 1A (Years 0-2) | Pearson | -0.002125345 | 0.987481291 | 0.987481291 |
| rh_medialorbitofrontal_volume | LNS (Years 2-4) | Pearson | 0.002042487 | 0.984327225 | 0.999824978 |
| rh_lateralorbitofrontal_volume | UPDRS 2 (Years 0-4) | Pearson | 0.001631415 | 0.987346646 | 0.987346646 |
| rh_medialorbitofrontal_volume | HVLDR (Years 2-4) | Pearson | -0.001393817 | 0.98936232 | 0.98936232 |
| Left-Amygdala_volume | SF (Years 0-4) | Pearson | 0.001261252 | 0.990321557 | 0.990321557 |
| rh_insula_volume | UPDRS 1A (Years 2-4) | Pearson | -0.00120623 | 0.992330671 | 0.992330671 |
| rh_entorhinal_volume | UPDRS 2 (Years 0-2) | Pearson | -0.000678501 | 0.994737328 | 0.994737328 |
| lh_entorhinal_volume | LNS (Years 0-2) | Pearson | -0.000630977 | 0.995105932 | 0.995105932 |
| lh_entorhinal_volume | HVLDR (Years 0-4) | Pearson | -0.000347054 | 0.997365706 | 0.997365706 |
| Left-Amygdala_volume | MoCA (Years 0-4) | Pearson | 0.000262009 | 0.997989383 | 0.997989383 |
| Right-Thalamus_volume | UPDRS 1 (Years 2-4) | Pearson | 0.000244125 | 0.998136771 | 0.998136771 |
| lh_medialorbitofrontal_volume | MoCA (Years 0-2) | Pearson | 6.21E-05 | 0.999520808 | 0.999520808 |
| lh_lateralorbitofrontal_volume | JLO (Years 0-2) | Pearson | 4.27E-05 | 0.999668548 | 0.999668548 |
| rh_entorhinal_volume | LNS (Years 2-4) | Pearson | -2.28E-05 | 0.999824978 | 0.999824978 |

NfL: neurofilament light chain, CSF: cerebrospinal fluid, Aβ1-42: Beta-Amyloid-(1-42), pTau: phosphorylated Tau, tTau: total Tau, ctx: cortex, rh: right hemisphere, lh: left hemisphere, MD: mean diffusivity, FA: fractional anisotropy, UPDRS: the Unified Parkinson's Disease Rating Scale, JLO: the Benton Judgment of Line Orientation test, HVLTR: the Hopkins Verbal Learning Total Recall test, HVLDR: the Hopkins Verbal Learning Delayed Recall test, MoCA: the Montreal Cognitive Assessment test, LNS: the Letter-Number Sequencing test, SF: the Semantic Fluency test, SDM: the Symbol Digit Modalities test, GDS: Geriatric Depression Scale (short form), FDR: false discovery rate.

**eTable 2.** The regressions between baseline imaging/fluid biomarkers and changes in clinical measures

| **Feature** | **Target** | **R-Squared** | **Beta** | **Beta Adjusted** | **Overall P-Value** | **Overall P-Value (FDR-corrected)** | **Main Variable P-Value** |
| --- | --- | --- | --- | --- | --- | --- | --- |
| ctx-lh-insula_FA | JLO (Years 2-4) | 0.165841982 | 68.14460248 | 0.113664491 | 0.006234449 | 0.013715787 | 0.31027686 |
| ctx-lh-lateralorbitofrontal_FA | HVLDR (Years 0-4) | 0.156751458 | 591.7887597 | 0.261452893 | 0.009330912 | 0.018661824 | 0.017384286 |
| ctx-lh-lateralorbitofrontal_FA | JLO (Years 2-4) | 0.188379474 | 191.0952011 | 0.19748897 | 0.002210962 | 0.009168493 | 0.064168996 |
| ctx-lh-lateralorbitofrontal_FA | LNS (Years 0-4) | 0.167368786 | 567.9757886 | 0.33831591 | 0.005390854 | 0.013177644 | 0.002014522 |
| ctx-lh-medialorbitofrontal_FA | LNS (Years 0-2) | 0.194663318 | 546.7562841 | 0.323357557 | 0.001240117 | 0.006820644 | 0.001268481 |
| ctx-rh-insula_FA | JLO (Years 2-4) | 0.17739285 | 108.9630529 | 0.167560923 | 0.003688623 | 0.010143712 | 0.1336448 |
| ctx-rh-lateralorbitofrontal_FA | HVLDR (Years 0-4) | 0.118835456 | 384.1896304 | 0.153618502 | 0.045400791 | 0.04994087 | 0.178693441 |
| ctx-rh-lateralorbitofrontal_FA | JLO (Years 2-4) | 0.224142535 | 315.3247189 | 0.294356522 | 0.000384611 | 0.004273319 | 0.006633503 |
| ctx-rh-lateralorbitofrontal_FA | LNS (Years 0-4) | 0.132554857 | 513.4181032 | 0.276902692 | 0.02467261 | 0.033924838 | 0.015048231 |
| ctx-rh-medialorbitofrontal_FA | LNS (Years 0-2) | 0.204689605 | 574.2973749 | 0.343901608 | 0.000750357 | 0.005502616 | 0.000687862 |
| ctx-rh-medialorbitofrontal_FA | LNS (Years 0-4) | 0.151692287 | 503.6995554 | 0.29394362 | 0.010872572 | 0.019933049 | 0.004983764 |
| Left-Amygdala_FA | JLO (Years 2-4) | 0.18246142 | 136.9029943 | 0.172193072 | 0.002917248 | 0.009168493 | 0.094825888 |
| Left-Thalamus-Proper_FA | JLO (Years 0-2) | 0.107224157 | -280.4163939 | -0.272186773 | 0.062516053 | 0.062516053 | 0.009303711 |
| Olfactory_Left_FA | HVLDR (Years 0-4) | 0.11649901 | 302.5968566 | 0.152832131 | 0.049766676 | 0.052136518 | 0.209230052 |
| Olfactory_Left_FA | LNS (Years 0-4) | 0.144611901 | 475.8272685 | 0.32339091 | 0.014795093 | 0.025037849 | 0.007497854 |
| Olfactory_Left_FA | SDM (Years 2-4) | 0.14143848 | 176.2768283 | 0.217776953 | 0.01695444 | 0.026642692 | 0.069198126 |
| Olfactory_Right_FA | HVLDR (Years 0-4) | 0.133987871 | 319.9737663 | 0.194043593 | 0.0246166 | 0.033924838 | 0.068182077 |
| Olfactory_Right_FA | LNS (Years 0-4) | 0.12609088 | 297.6852817 | 0.244732889 | 0.032225151 | 0.039365767 | 0.021899793 |
| Olfactory_Right_FA | SDM (Years 2-4) | 0.18106728 | 190.4244036 | 0.284574373 | 0.002859016 | 0.009168493 | 0.006223448 |
| Right-Amygdala_FA | HVLDR (Years 0-4) | 0.126082769 | 320.4502476 | 0.169372759 | 0.033997708 | 0.039365767 | 0.111620765 |
| Right-Amygdala_FA | JLO (Years 2-4) | 0.223944696 | 222.6248877 | 0.275079257 | 0.000388484 | 0.004273319 | 0.006716331 |
| Right-Thalamus-Proper_FA | HVLDR (Years 0-4) | 0.126742367 | 316.8958442 | 0.171112805 | 0.033103628 | 0.039365767 | 0.107053229 |
| ctx-lh-insula_MD | HVLDR (Years 0-4) | 0.154042316 | -111824.7878 | -0.276295675 | 0.010504452 | 0.027836798 | 0.020410128 |
| ctx-lh-insula_MD | HVLTR (Years 0-2) | 0.129035432 | -69347.48591 | -0.359273605 | 0.025575382 | 0.043725654 | 0.002977132 |
| ctx-lh-insula_MD | HVLTR (Years 0-4) | 0.109492703 | -70084.01064 | -0.311342696 | 0.062437604 | 0.076957978 | 0.010637923 |
| ctx-lh-insula_MD | JLO (Years 2-4) | 0.176416635 | -29522.55349 | -0.170114684 | 0.003857966 | 0.020447219 | 0.142954333 |
| ctx-lh-insula_MD | SDM (Years 0-4) | 0.183506412 | -85233.15175 | -0.399402285 | 0.002548552 | 0.020447219 | 0.000738922 |
| ctx-lh-lateralorbitofrontal_MD | HVLDR (Years 0-4) | 0.13521061 | -75211.74291 | -0.192090912 | 0.0234018 | 0.042090676 | 0.063256017 |
| ctx-lh-lateralorbitofrontal_MD | LNS (Years 0-4) | 0.137539739 | -76283.94225 | -0.262576752 | 0.020011679 | 0.039282185 | 0.011278517 |
| ctx-lh-lateralorbitofrontal_MD | SDM (Years 0-4) | 0.118874253 | -46337.9968 | -0.224619891 | 0.043152867 | 0.058643639 | 0.031126469 |
| ctx-lh-medialorbitofrontal_MD | LNS (Years 0-4) | 0.143568141 | -75583.86022 | -0.282714972 | 0.015475075 | 0.034581933 | 0.007963258 |
| ctx-rh-insula_MD | HVLDR (Years 0-2) | 0.13706987 | -119120.9075 | -0.27136587 | 0.019234292 | 0.039208365 | 0.029984818 |
| ctx-rh-insula_MD | HVLDR (Years 0-4) | 0.146231375 | -114688.4918 | -0.267831035 | 0.014715093 | 0.034581933 | 0.032494595 |
| ctx-rh-insula_MD | HVLTR (Years 0-2) | 0.144114631 | -84038.01844 | -0.405385177 | 0.013311632 | 0.033596023 | 0.001258668 |
| ctx-rh-insula_MD | HVLTR (Years 0-4) | 0.102562861 | -73680.17735 | -0.309328708 | 0.081387762 | 0.095856697 | 0.015677113 |
| ctx-rh-insula_MD | JLO (Years 2-4) | 0.181948191 | -37166.24734 | -0.201227987 | 0.002987734 | 0.020447219 | 0.098136891 |
| ctx-rh-insula_MD | LNS (Years 0-4) | 0.096991687 | -62045.02655 | -0.195106163 | 0.10020342 | 0.112995346 | 0.12490472 |
| ctx-rh-insula_MD | SDM (Years 0-4) | 0.158942955 | -83548.10517 | -0.369988675 | 0.007885716 | 0.024266013 | 0.003070325 |
| ctx-rh-lateralorbitofrontal_MD | HVLDR (Years 0-4) | 0.120582858 | -91235.88819 | -0.155766747 | 0.042368287 | 0.058643639 | 0.15916374 |
| ctx-rh-lateralorbitofrontal_MD | LNS (Years 0-4) | 0.168278946 | -147715.0738 | -0.339478186 | 0.005171532 | 0.022319141 | 0.001911058 |
| ctx-rh-lateralorbitofrontal_MD | SDM (Years 0-4) | 0.101162331 | -58529.73308 | -0.189431158 | 0.085793853 | 0.098849439 | 0.089433586 |
| ctx-rh-medialorbitofrontal_MD | LNS (Years 0-4) | 0.143292112 | -77895.11979 | -0.276289047 | 0.015659743 | 0.034581933 | 0.008091108 |
| Left-Amygdala_MD | HVLDR (Years 0-2) | 0.121387079 | -83061.3818 | -0.184788314 | 0.037079732 | 0.055965116 | 0.078383097 |
| Left-Amygdala_MD | HVLDR (Years 0-4) | 0.158346292 | -110608.7809 | -0.253219199 | 0.008699137 | 0.024266013 | 0.015819609 |
| Left-Amygdala_MD | JLO (Years 2-4) | 0.2390302 | -56320.35512 | -0.30356225 | 0.000178985 | 0.009486227 | 0.00260531 |
| Left-Thalamus-Proper_MD | HVLDR (Years 0-2) | 0.1320343 | -66792.50903 | -0.256907973 | 0.023824911 | 0.042090676 | 0.040678045 |
| Left-Thalamus-Proper_MD | HVLDR (Years 0-4) | 0.16536256 | -81451.55494 | -0.319061469 | 0.006369842 | 0.022506774 | 0.010456876 |
| Left-Thalamus-Proper_MD | HVLTR (Years 0-2) | 0.079118076 | -30429.21901 | -0.248811634 | 0.178301778 | 0.178301778 | 0.051948772 |
| Left-Thalamus-Proper_MD | HVLTR (Years 0-4) | 0.112153245 | -46990.41248 | -0.33219479 | 0.056295248 | 0.071039242 | 0.009168793 |
| Left-Thalamus-Proper_MD | JLO (Years 2-4) | 0.180248125 | -21124.89079 | -0.194382054 | 0.003232996 | 0.020447219 | 0.110022205 |
| Left-Thalamus-Proper_MD | SDM (Years 0-4) | 0.094445957 | -25426.17362 | -0.189603709 | 0.110018693 | 0.121478973 | 0.135733599 |
| Left-Thalamus-Proper_MD | SDM (Years 2-4) | 0.175745044 | -33561.28634 | -0.323038893 | 0.003666342 | 0.020447219 | 0.008566297 |
| Olfactory_Left_MD | HVLDR (Years 0-2) | 0.12056538 | -54809.08563 | -0.20711532 | 0.038344566 | 0.055965116 | 0.082525659 |
| Olfactory_Left_MD | HVLDR (Years 0-4) | 0.131189268 | -54614.77224 | -0.21142585 | 0.027621268 | 0.045747725 | 0.081039559 |
| Olfactory_Left_MD | JLO (Years 2-4) | 0.160758338 | -9205.181781 | -0.083610169 | 0.007819915 | 0.024266013 | 0.480447528 |
| Olfactory_Left_MD | LNS (Years 0-4) | 0.122752891 | -51504.29363 | -0.269487257 | 0.036915519 | 0.055965116 | 0.026604222 |
| Olfactory_Left_MD | SDM (Years 0-4) | 0.090796313 | -22763.18234 | -0.16773187 | 0.125564187 | 0.130488272 | 0.171510043 |
| Olfactory_Right_MD | HVLDR (Years 0-4) | 0.135493859 | -35043.29923 | -0.214151359 | 0.023128473 | 0.042090676 | 0.062169021 |
| Olfactory_Right_MD | JLO (Years 2-4) | 0.167221661 | -8392.673614 | -0.121498365 | 0.005859874 | 0.022319141 | 0.27851047 |
| Olfactory_Right_MD | LNS (Years 0-4) | 0.121349184 | -30329.1655 | -0.252455072 | 0.039069987 | 0.055965116 | 0.028879096 |
| Olfactory_Right_MD | SDM (Years 0-4) | 0.12682826 | -22977.92625 | -0.26935329 | 0.031266333 | 0.050215625 | 0.019584513 |
| Right-Amygdala_MD | HVLDR (Years 0-2) | 0.138067285 | -115662.6425 | -0.234075284 | 0.018429303 | 0.039070123 | 0.028234734 |
| Right-Amygdala_MD | HVLDR (Years 0-4) | 0.211573581 | -175034.2896 | -0.360579597 | 0.000721404 | 0.019117215 | 0.000681225 |
| Right-Amygdala_MD | HVLTR (Years 0-2) | 0.090892634 | -56476.80811 | -0.242560385 | 0.116781513 | 0.123788403 | 0.026210211 |
| Right-Amygdala_MD | HVLTR (Years 0-4) | 0.086312607 | -61862.85469 | -0.229651868 | 0.147255688 | 0.150087528 | 0.039239861 |
| Right-Amygdala_MD | JLO (Years 2-4) | 0.195107497 | -44299.93002 | -0.214089065 | 0.001606443 | 0.020447219 | 0.041521046 |
| Right-Amygdala_MD | SDM (Years 0-4) | 0.103465311 | -49545.10919 | -0.194009536 | 0.078657208 | 0.094746183 | 0.077736862 |
| Right-Thalamus-Proper_MD | HVLDR (Years 0-2) | 0.163748941 | -81188.65255 | -0.337094959 | 0.005895622 | 0.022319141 | 0.006097979 |
| Right-Thalamus-Proper_MD | HVLDR (Years 0-4) | 0.18479309 | -85006.82916 | -0.360600664 | 0.002616509 | 0.020447219 | 0.00333101 |
| Right-Thalamus-Proper_MD | HVLTR (Years 0-2) | 0.111936795 | -38033.70653 | -0.334205975 | 0.051817741 | 0.06698391 | 0.007874506 |
| Right-Thalamus-Proper_MD | HVLTR (Years 0-4) | 0.157048805 | -55396.77417 | -0.422233792 | 0.008580666 | 0.024266013 | 0.000741417 |
| Right-Thalamus-Proper_MD | JLO (Years 2-4) | 0.167495411 | -13462.43179 | -0.133156783 | 0.005788136 | 0.022319141 | 0.272700488 |
| Right-Thalamus-Proper_MD | LNS (Years 0-4) | 0.092824693 | -30890.06865 | -0.176352586 | 0.116702516 | 0.123788403 | 0.1628836 |
| Right-Thalamus-Proper_MD | SDM (Years 0-4) | 0.118136125 | -33385.55268 | -0.268416583 | 0.044444393 | 0.058888821 | 0.032501205 |
| Right-Thalamus-Proper_MD | SDM (Years 2-4) | 0.182665052 | -32510.48031 | -0.337384055 | 0.002651818 | 0.020447219 | 0.005654335 |
| Left-Thalamus_volume | HVLDR (Years 0-4) | 0.120014006 | 13826.25099 | 0.184908714 | 0.043334437 | 0.081091409 | 0.165243921 |
| Left-Amygdala_volume | UPDRS 2 (Years 2-4) | 0.184927641 | 190697.7844 | 0.38154608 | 0.002600089 | 0.015600535 | 0.000525466 |
| Right-Amygdala_volume | HVLDR (Years 0-2) | 0.148689269 | 89239.96073 | 0.264146851 | 0.011605675 | 0.034817025 | 0.014939762 |
| Right-Thalamus_volume | HVLDR (Years 0-4) | 0.114377072 | 11822.21179 | 0.152489658 | 0.054060939 | 0.081091409 | 0.242355734 |
| Right-Amygdala_volume | HVLTR (Years 0-4) | 0.087379841 | 43125.41743 | 0.233760514 | 0.141818748 | 0.141818748 | 0.036924463 |
| rh_insula_volume | HVLTR (Years 0-4) | 0.088318617 | 14586.51942 | 0.231921177 | 0.137180302 | 0.141818748 | 0.035003972 |
| lh_lateralorbitofrontal_thickness | SF (Years 0-2) | 0.24230179 | 60.24135422 | 0.40178219 | 0.000104146 | 0.000416584 | 3.47E-05 |
| rh_entorhinal_thickness | HVLTR (Years 2-4) | 0.140943967 | -25.08439199 | -0.347904526 | 0.017316422 | 0.017316422 | 0.000761318 |
| lh_medialorbitofrontal_thickness | UPDRS 1B (Years 0-2) | 0.174445126 | -450.8762268 | -0.321702531 | 0.005379419 | 0.007172559 | 0.002721086 |
| lh_medialorbitofrontal_thickness | SF (Years 0-2) | 0.185731221 | 49.14837725 | 0.333219531 | 0.00192356 | 0.003847121 | 0.001115131 |
| Serum - NfL | HVLDR (Years 0-4) | 0.204375148 | -2.808252986 | -0.429949182 | 0.001682482 | 0.004486619 | 0.002980412 |
| Serum - NfL | HVLTR (Years 0-2) | 0.215643768 | -1.88663676 | -0.602850422 | 0.00072866 | 0.004486619 | 3.13E-05 |
| Serum - NfL | JLO (Years 2-4) | 0.211758245 | -0.959751117 | -0.33112618 | 0.001198274 | 0.004486619 | 0.019499359 |
| Cerebrospinal Fluid - NFL | HVLTR (Years 0-2) | 0.20948227 | -0.138745837 | -0.4030265 | 0.069399264 | 0.079313445 | 0.014343411 |
| Cerebrospinal Fluid - NFL | HVLDR (Years 0-4) | 0.284984409 | -0.148490865 | -0.216970416 | 0.012359987 | 0.024719974 | 0.155571872 |
| Serum - NfL | HVLTR (Years 0-4) | 0.129992233 | -1.574458114 | -0.425472527 | 0.03615137 | 0.048201826 | 0.004564161 |
| Serum - NfL | HVLDR (Years 0-2) | 0.147392389 | -3.711829328 | -0.541238881 | 0.017012683 | 0.027220292 | 0.000292287 |
| Serum - NfL | JLO (Years 0-2) | 0.094322634 | 1.215451873 | 0.339565399 | 0.123124613 | 0.123124613 | 0.023595228 |

NfL: neurofilament light chain, CSF: cerebrospinal fluid, Aβ1-42: Beta-Amyloid-(1-42), pTau: phosphorylated Tau, tTau: total Tau, ctx: cortex, rh: right hemisphere, lh: left hemisphere, MD: mean diffusivity, FA: fractional anisotropy, UPDRS: the Unified Parkinson's Disease Rating Scale, JLO: the Benton Judgment of Line Orientation test, HVLTR: the Hopkins Verbal Learning Total Recall test, HVLDR: the Hopkins Verbal Learning Delayed Recall test, MoCA: the Montreal Cognitive Assessment test, LNS: the Letter-Number Sequencing test, SF: the Semantic Fluency test, SDM: the Symbol Digit Modalities test, GDS: Geriatric Depression Scale (short form), FDR: false discovery rate.

**eTable 3.** The correlations between baseline imaging/fluid biomarkers and changes in UPDRS 3 scores

| **Feature** | **Target** | **Correlation Type** | **Correlation Coefficient** | **Raw P-value** | **P-value (FDR-corrected)** |
| --- | --- | --- | --- | --- | --- |
| ctx-lh-medialorbitofrontal_FA | UPDRS 3, Off (Years 0-4) | Pearson | 0.99695712 | 0.00304288 | 0.042600321 |
| rh_entorhinal_volume | UPDRS 3, On (Years 0-4) | Pearson | -0.903996982 | 0.005205429 | 0.062465149 |
| ctx-lh-insula_FA | UPDRS 3, On (Years 0-2) | Pearson | 0.866415916 | 0.01164211 | 0.080982519 |
| ctx-lh-lateralorbitofrontal_FA | UPDRS 3, On (Years 0-2) | Pearson | 0.832708043 | 0.020047804 | 0.080982519 |
| ctx-rh-lateralorbitofrontal_FA | UPDRS 3, On (Years 0-2) | Pearson | 0.81579668 | 0.025260271 | 0.080982519 |
| ctx-rh-medialorbitofrontal_FA | UPDRS 3, On (Years 0-2) | Pearson | 0.797125447 | 0.031813628 | 0.080982519 |
| ctx-rh-entorhinal_FA | UPDRS 3, On (Years 0-2) | Pearson | 0.796289784 | 0.032126797 | 0.080982519 |
| ctx-rh-insula_FA | UPDRS 3, On (Years 0-2) | Pearson | 0.785368276 | 0.036377653 | 0.080982519 |
| ctx-lh-entorhinal_FA | UPDRS 3, On (Years 0-2) | Pearson | 0.775456009 | 0.040491259 | 0.080982519 |
| rh_lateralorbitofrontal_thickness | UPDRS 3, On (Years 2-4) | Pearson | -0.272079044 | 0.024796933 | 0.098538539 |
| rh_medialorbitofrontal_thickness | UPDRS 3, On (Years 2-4) | Pearson | -0.254336646 | 0.036350775 | 0.098538539 |
| rh_entorhinal_thickness | UPDRS 3, On (Years 2-4) | Pearson | -0.247762086 | 0.041639946 | 0.098538539 |
| lh_medialorbitofrontal_thickness | UPDRS 3, On (Years 2-4) | Pearson | -0.23940205 | 0.04926927 | 0.098538539 |
| rh_insula_thickness | UPDRS 3, On (Years 2-4) | Pearson | -0.218164633 | 0.07389316 | 0.118229057 |
| lh_medialorbitofrontal_volume | UPDRS 3, Off (Years 0-4) | Pearson | -0.989144004 | 0.010855996 | 0.13027195 |
| ctx-lh-medialorbitofrontal_FA | UPDRS 3, On (Years 0-2) | Pearson | 0.680436396 | 0.092499921 | 0.161874862 |
| lh_entorhinal_thickness | UPDRS 3, On (Years 2-4) | Pearson | -0.18382549 | 0.133464289 | 0.171919023 |
| lh_insula_thickness | UPDRS 3, On (Years 2-4) | Pearson | -0.176284527 | 0.150429145 | 0.171919023 |
| Olfactory_Left_FA | UPDRS 3, Off (Years 0-4) | Pearson | 0.974706833 | 0.025293167 | 0.177052171 |
| Right-Thalamus_volume | UPDRS 3, Off (Years 0-4) | Pearson | -0.968900147 | 0.031099853 | 0.186599117 |
| Right-Amygdala_FA | UPDRS 3, Off (Years 0-2) | Pearson | 0.985729261 | 0.014270739 | 0.199790347 |
| ctx-lh-lateralorbitofrontal_FA | UPDRS 3, Off (Years 0-4) | Pearson | 0.921448626 | 0.078551374 | 0.208259577 |
| ctx-lh-insula_FA | UPDRS 3, Off (Years 0-4) | Pearson | 0.916867898 | 0.083132102 | 0.208259577 |
| ctx-rh-medialorbitofrontal_FA | UPDRS 3, Off (Years 0-4) | Pearson | 0.905452929 | 0.094547071 | 0.208259577 |
| ctx-rh-lateralorbitofrontal_FA | UPDRS 3, Off (Years 0-4) | Pearson | 0.896319041 | 0.103680959 | 0.208259577 |
| ctx-rh-insula_FA | UPDRS 3, Off (Years 0-4) | Pearson | 0.895870212 | 0.104129788 | 0.208259577 |
| Left-Amygdala_FA | UPDRS 3, On (Years 0-2) | Pearson | 0.602571668 | 0.15215005 | 0.236677855 |
| Cerebrospinal Fluid - CSF Alpha-synuclein | UPDRS 3, On (Years 0-2) | Pearson | 0.813796064 | 0.093712822 | 0.242665645 |
| Serum - NfL | UPDRS 3, On (Years 0-2) | Pearson | -0.634514746 | 0.175958543 | 0.242665645 |
| Cerebrospinal Fluid - tTau | UPDRS 3, On (Years 0-2) | Spearman | 0.7 | 0.188120404 | 0.242665645 |
| Cerebrospinal Fluid - ABeta 1-42 | UPDRS 3, On (Years 0-2) | Pearson | 0.693417569 | 0.194132516 | 0.242665645 |
| ctx-rh-entorhinal_FA | UPDRS 3, Off (Years 0-4) | Pearson | 0.85183672 | 0.14816328 | 0.25928574 |
| Left-Amygdala_FA | UPDRS 3, Off (Years 0-2) | Pearson | 0.959133673 | 0.040866327 | 0.28606429 |
| Right-Amygdala_FA | UPDRS 3, On (Years 2-4) | Pearson | 0.276980041 | 0.022217633 | 0.311046863 |
| Cerebrospinal Fluid - ABeta 1-42 | UPDRS 3, On (Years 0-4) | Pearson | 0.729927879 | 0.161482774 | 0.313534007 |
| Cerebrospinal Fluid - pTau | UPDRS 3, On (Years 0-4) | Spearman | 0.7 | 0.188120404 | 0.313534007 |
| Cerebrospinal Fluid - tTau | UPDRS 3, On (Years 0-4) | Spearman | 0.7 | 0.188120404 | 0.313534007 |
| ctx-lh-entorhinal_FA | UPDRS 3, Off (Years 0-2) | Pearson | 0.882863009 | 0.117136991 | 0.323302532 |
| ctx-lh-lateralorbitofrontal_FA | UPDRS 3, Off (Years 0-2) | Pearson | 0.852004494 | 0.147995506 | 0.323302532 |
| Olfactory_Left_FA | UPDRS 3, Off (Years 0-2) | Pearson | 0.820151988 | 0.179848012 | 0.323302532 |
| ctx-rh-lateralorbitofrontal_FA | UPDRS 3, Off (Years 0-2) | Pearson | 0.806330601 | 0.193669399 | 0.323302532 |
| ctx-rh-medialorbitofrontal_FA | UPDRS 3, Off (Years 0-2) | Pearson | 0.799310491 | 0.200689509 | 0.323302532 |
| ctx-rh-entorhinal_FA | UPDRS 3, Off (Years 0-2) | Pearson | 0.784209725 | 0.215790275 | 0.323302532 |
| ctx-rh-insula_FA | UPDRS 3, Off (Years 0-2) | Pearson | 0.778132411 | 0.221867589 | 0.323302532 |
| ctx-lh-insula_FA | UPDRS 3, Off (Years 0-2) | Pearson | 0.76906962 | 0.23093038 | 0.323302532 |
| Right-Amygdala_FA | UPDRS 3, Off (Years 0-4) | Pearson | 0.755876336 | 0.244123664 | 0.379747922 |
| ctx-rh-entorhinal_MD | UPDRS 3, On (Years 0-2) | Pearson | -0.809043403 | 0.02753295 | 0.385461307 |
| Right-Thalamus-Proper_FA | UPDRS 3, On (Years 0-2) | Pearson | 0.45711372 | 0.30244693 | 0.389997524 |
| Olfactory_Left_FA | UPDRS 3, On (Years 0-2) | Pearson | 0.453791328 | 0.306426626 | 0.389997524 |
| rh_entorhinal_thickness | UPDRS 3, On (Years 0-4) | Pearson | -0.692105887 | 0.084876989 | 0.392398824 |
| lh_lateralorbitofrontal_thickness | UPDRS 3, On (Years 0-4) | Pearson | -0.672183656 | 0.098099706 | 0.392398824 |
| Cerebrospinal Fluid - CSF Alpha-synuclein | UPDRS 3, Off (Years 0-4) | Pearson | 0.992287015 | 0.079119933 | 0.395599667 |
| ctx-lh-entorhinal_FA | UPDRS 3, Off (Years 2-4) | Pearson | 0.314642916 | 0.057867849 | 0.400481035 |
| Right-Amygdala_FA | UPDRS 3, Off (Years 2-4) | Pearson | 0.292444725 | 0.079012728 | 0.400481035 |
| Left-Thalamus-Proper_FA | UPDRS 3, Off (Years 2-4) | Pearson | 0.286299349 | 0.085817365 | 0.400481035 |
| lh_insula_volume | UPDRS 3, On (Years 0-4) | Pearson | -0.649897326 | 0.114083091 | 0.407657913 |
| lh_medialorbitofrontal_volume | UPDRS 3, On (Years 0-4) | Pearson | -0.641818751 | 0.120185994 | 0.407657913 |
| Right-Amygdala_volume | UPDRS 3, On (Years 0-4) | Pearson | -0.621955274 | 0.135885971 | 0.407657913 |
| Left-Amygdala_volume | UPDRS 3, On (Years 2-4) | Pearson | 0.257172243 | 0.034248856 | 0.410986269 |
| rh_medialorbitofrontal_volume | UPDRS 3, Off (Years 0-4) | Pearson | -0.864473958 | 0.135526042 | 0.422594456 |
| lh_insula_volume | UPDRS 3, Off (Years 0-4) | Pearson | -0.859135181 | 0.140864819 | 0.422594456 |
| Cerebrospinal Fluid - ABeta 1-42 | UPDRS 3, Off (Years 0-4) | Pearson | 0.961104065 | 0.178141483 | 0.445353709 |
| Serum - NfL | UPDRS 3, On (Years 0-4) | Pearson | 0.459872001 | 0.358819382 | 0.448524228 |
| ctx-lh-medialorbitofrontal_FA | UPDRS 3, Off (Years 0-2) | Pearson | 0.616745781 | 0.383254219 | 0.487778097 |
| Right-Thalamus-Proper_FA | UPDRS 3, Off (Years 2-4) | Pearson | 0.245753545 | 0.142608722 | 0.499130528 |
| ctx-rh-entorhinal_FA | UPDRS 3, Off (Years 2-4) | Pearson | 0.221199936 | 0.188277142 | 0.527175998 |
| ctx-lh-lateralorbitofrontal_FA | UPDRS 3, Off (Years 2-4) | Pearson | 0.201333543 | 0.23212072 | 0.541615014 |
| Left-Amygdala_volume | UPDRS 3, On (Years 0-4) | Pearson | -0.520180539 | 0.231370788 | 0.55528989 |
| rh_entorhinal_volume | UPDRS 3, Off (Years 0-4) | Pearson | -0.768547461 | 0.231452539 | 0.555486093 |
| Olfactory_Left_FA | UPDRS 3, Off (Years 2-4) | Pearson | -0.182901463 | 0.278571833 | 0.557143666 |
| Left-Thalamus-Proper_FA | UPDRS 3, On (Years 0-2) | Pearson | 0.308577121 | 0.500723606 | 0.58417754 |
| Olfactory_Right_FA | UPDRS 3, On (Years 2-4) | Pearson | 0.191294404 | 0.118128411 | 0.616012073 |
| ctx-lh-insula_FA | UPDRS 3, On (Years 2-4) | Pearson | -0.17555809 | 0.152144555 | 0.616012073 |
| ctx-rh-insula_FA | UPDRS 3, On (Years 2-4) | Pearson | -0.143378215 | 0.24343448 | 0.616012073 |
| Olfactory_Left_FA | UPDRS 3, On (Years 2-4) | Pearson | -0.131545104 | 0.284937875 | 0.616012073 |
| Right-Thalamus-Proper_FA | UPDRS 3, On (Years 2-4) | Pearson | -0.114925005 | 0.350704978 | 0.616012073 |
| ctx-rh-medialorbitofrontal_FA | UPDRS 3, On (Years 2-4) | Pearson | -0.114646547 | 0.351881229 | 0.616012073 |
| ctx-lh-lateralorbitofrontal_FA | UPDRS 3, On (Years 2-4) | Pearson | 0.114616831 | 0.352006899 | 0.616012073 |
| Cerebrospinal Fluid - pTau | UPDRS 3, Off (Years 2-4) | Spearman | 0.292122335 | 0.088601206 | 0.620208442 |
| lh_lateralorbitofrontal_thickness | UPDRS 3, On (Years 2-4) | Pearson | -0.060553661 | 0.623758078 | 0.623758078 |
| Cerebrospinal Fluid - pTau | UPDRS 3, On (Years 0-2) | Spearman | -0.3 | 0.623837665 | 0.623837665 |
| Right-Amygdala_FA | UPDRS 3, On (Years 0-2) | Pearson | 0.252375198 | 0.58506773 | 0.63007294 |
| Right-Thalamus-Proper_FA | UPDRS 3, Off (Years 0-2) | Pearson | 0.455086619 | 0.544913381 | 0.635732277 |
| rh_insula_volume | UPDRS 3, Off (Years 0-4) | Pearson | -0.669664747 | 0.330335253 | 0.660670505 |
| Left-Amygdala_MD | UPDRS 3, On (Years 2-4) | Pearson | -0.207470042 | 0.089574016 | 0.669469171 |
| Right-Thalamus-Proper_MD | UPDRS 3, On (Years 2-4) | Pearson | 0.164379343 | 0.180405368 | 0.669469171 |
| ctx-lh-insula_MD | UPDRS 3, On (Years 2-4) | Pearson | 0.153360832 | 0.211811017 | 0.669469171 |
| ctx-rh-insula_MD | UPDRS 3, On (Years 2-4) | Pearson | 0.124865571 | 0.310317803 | 0.669469171 |
| ctx-lh-entorhinal_MD | UPDRS 3, On (Years 2-4) | Pearson | -0.116167791 | 0.345484991 | 0.669469171 |
| ctx-rh-entorhinal_MD | UPDRS 3, On (Years 2-4) | Pearson | -0.111377224 | 0.365873416 | 0.669469171 |
| Olfactory_Right_MD | UPDRS 3, On (Years 2-4) | Pearson | -0.105657295 | 0.391155942 | 0.669469171 |
| ctx-rh-lateralorbitofrontal_MD | UPDRS 3, On (Years 2-4) | Pearson | -0.094004152 | 0.445763339 | 0.669469171 |
| Right-Amygdala_MD | UPDRS 3, On (Years 2-4) | Pearson | -0.089578539 | 0.467562444 | 0.669469171 |
| ctx-rh-medialorbitofrontal_MD | UPDRS 3, On (Years 2-4) | Pearson | 0.08372239 | 0.497273506 | 0.669469171 |
| ctx-lh-lateralorbitofrontal_MD | UPDRS 3, On (Years 2-4) | Pearson | -0.076123022 | 0.53724474 | 0.669469171 |
| ctx-lh-medialorbitofrontal_MD | UPDRS 3, On (Years 2-4) | Pearson | -0.069407768 | 0.573830718 | 0.669469171 |
| Olfactory_Right_FA | UPDRS 3, On (Years 0-2) | Pearson | 0.197685779 | 0.67091718 | 0.67091718 |
| Left-Amygdala_FA | UPDRS 3, Off (Years 0-4) | Pearson | 0.508351656 | 0.491648344 | 0.688307682 |
| rh_lateralorbitofrontal_thickness | UPDRS 3, On (Years 0-4) | Pearson | -0.452304691 | 0.30821475 | 0.695634393 |
| rh_medialorbitofrontal_thickness | UPDRS 3, On (Years 0-4) | Pearson | -0.420280313 | 0.347817196 | 0.695634393 |
| ctx-rh-entorhinal_FA | UPDRS 3, On (Years 2-4) | Pearson | 0.089825917 | 0.466328916 | 0.696143713 |
| ctx-lh-entorhinal_FA | UPDRS 3, On (Years 2-4) | Pearson | 0.075328057 | 0.541515375 | 0.696143713 |
| Left-Amygdala_FA | UPDRS 3, On (Years 2-4) | Pearson | 0.074317116 | 0.54697006 | 0.696143713 |
| Left-Thalamus-Proper_MD | UPDRS 3, On (Years 2-4) | Pearson | 0.054490335 | 0.658969557 | 0.709659523 |
| lh_lateralorbitofrontal_thickness | UPDRS 3, Off (Years 0-4) | Pearson | -0.800857957 | 0.199142043 | 0.72286327 |
| rh_lateralorbitofrontal_thickness | UPDRS 3, Off (Years 0-4) | Pearson | -0.766792271 | 0.233207729 | 0.72286327 |
| lh_insula_thickness | UPDRS 3, Off (Years 0-4) | Pearson | 0.524633127 | 0.475366873 | 0.72286327 |
| lh_entorhinal_thickness | UPDRS 3, Off (Years 0-4) | Pearson | 0.362153105 | 0.637846895 | 0.72286327 |
| rh_entorhinal_thickness | UPDRS 3, Off (Years 0-4) | Pearson | -0.318407819 | 0.681592181 | 0.72286327 |
| rh_insula_thickness | UPDRS 3, Off (Years 0-4) | Pearson | 0.299006436 | 0.700993564 | 0.72286327 |
| rh_medialorbitofrontal_thickness | UPDRS 3, Off (Years 0-4) | Pearson | -0.280552706 | 0.719447294 | 0.72286327 |
| lh_medialorbitofrontal_thickness | UPDRS 3, Off (Years 0-4) | Pearson | -0.27713673 | 0.72286327 | 0.72286327 |
| ctx-lh-medialorbitofrontal_FA | UPDRS 3, On (Years 0-4) | Pearson | 0.741272067 | 0.056565461 | 0.728015205 |
| ctx-rh-insula_FA | UPDRS 3, On (Years 0-4) | Pearson | 0.471112911 | 0.285930604 | 0.728015205 |
| ctx-rh-lateralorbitofrontal_FA | UPDRS 3, On (Years 0-4) | Pearson | 0.457999058 | 0.301390288 | 0.728015205 |
| ctx-lh-insula_FA | UPDRS 3, On (Years 0-4) | Pearson | 0.439651083 | 0.323617142 | 0.728015205 |
| ctx-rh-medialorbitofrontal_FA | UPDRS 3, On (Years 0-4) | Pearson | 0.425745098 | 0.340915069 | 0.728015205 |
| ctx-lh-lateralorbitofrontal_FA | UPDRS 3, On (Years 0-4) | Pearson | 0.424404702 | 0.342602614 | 0.728015205 |
| ctx-rh-entorhinal_FA | UPDRS 3, On (Years 0-4) | Pearson | 0.407636744 | 0.364007602 | 0.728015205 |
| rh_insula_thickness | UPDRS 3, On (Years 0-4) | Pearson | 0.339082458 | 0.456869816 | 0.730991706 |
| Left-Thalamus-Proper_FA | UPDRS 3, Off (Years 0-2) | Pearson | 0.320408617 | 0.679591383 | 0.731867643 |
| Left-Thalamus_volume | UPDRS 3, Off (Years 0-4) | Pearson | -0.569041938 | 0.430958062 | 0.735064591 |
| Right-Amygdala_volume | UPDRS 3, Off (Years 0-4) | Pearson | -0.509956939 | 0.490043061 | 0.735064591 |
| ctx-lh-entorhinal_MD | UPDRS 3, Off (Years 0-2) | Pearson | -0.9471053 | 0.0528947 | 0.740525804 |
| lh_medialorbitofrontal_thickness | UPDRS 3, On (Years 0-4) | Pearson | -0.268250702 | 0.560809874 | 0.747746499 |
| Left-Amygdala_volume | UPDRS 3, Off (Years 2-4) | Pearson | 0.255136329 | 0.127504851 | 0.75149872 |
| rh_medialorbitofrontal_volume | UPDRS 3, Off (Years 2-4) | Pearson | -0.166789606 | 0.323812478 | 0.75149872 |
| Left-Thalamus_volume | UPDRS 3, Off (Years 2-4) | Pearson | -0.135439297 | 0.424138759 | 0.75149872 |
| lh_entorhinal_volume | UPDRS 3, Off (Years 2-4) | Pearson | -0.121120971 | 0.475166207 | 0.75149872 |
| lh_medialorbitofrontal_volume | UPDRS 3, Off (Years 2-4) | Pearson | -0.120344094 | 0.478023821 | 0.75149872 |
| lh_lateralorbitofrontal_volume | UPDRS 3, Off (Years 2-4) | Pearson | -0.11913285 | 0.482497046 | 0.75149872 |
| rh_lateralorbitofrontal_volume | UPDRS 3, Off (Years 2-4) | Pearson | -0.118514006 | 0.484790856 | 0.75149872 |
| Right-Thalamus_volume | UPDRS 3, Off (Years 2-4) | Pearson | -0.095147099 | 0.575366066 | 0.75149872 |
| lh_insula_volume | UPDRS 3, Off (Years 2-4) | Pearson | -0.069748328 | 0.681658527 | 0.75149872 |
| Right-Amygdala_volume | UPDRS 3, Off (Years 2-4) | Pearson | 0.069628422 | 0.682177121 | 0.75149872 |
| rh_entorhinal_volume | UPDRS 3, Off (Years 2-4) | Pearson | -0.068083008 | 0.688873827 | 0.75149872 |
| Serum - NfL | UPDRS 3, Off (Years 0-2) | Pearson | -0.747664505 | 0.462350299 | 0.759445206 |
| Cerebrospinal Fluid - CSF Alpha-synuclein | UPDRS 3, Off (Years 0-2) | Pearson | 0.508468499 | 0.660423656 | 0.759445206 |
| Cerebrospinal Fluid - pTau | UPDRS 3, Off (Years 0-2) | Spearman | -0.5 | 0.666666667 | 0.759445206 |
| Cerebrospinal Fluid - tTau | UPDRS 3, Off (Years 0-2) | Spearman | -0.5 | 0.666666667 | 0.759445206 |
| Cerebrospinal Fluid - ABeta 1-42 | UPDRS 3, Off (Years 0-2) | Pearson | 0.368934684 | 0.759445206 | 0.759445206 |
| rh_insula_volume | UPDRS 3, On (Years 0-4) | Pearson | -0.390972768 | 0.385807915 | 0.771615829 |
| rh_medialorbitofrontal_volume | UPDRS 3, On (Years 0-4) | Pearson | -0.3365317 | 0.46048046 | 0.789395075 |
| Olfactory_Right_FA | UPDRS 3, Off (Years 2-4) | Pearson | 0.116029604 | 0.494056137 | 0.801820628 |
| ctx-rh-insula_FA | UPDRS 3, Off (Years 2-4) | Pearson | -0.093350783 | 0.582632408 | 0.801820628 |
| ctx-lh-medialorbitofrontal_FA | UPDRS 3, Off (Years 2-4) | Pearson | 0.087469298 | 0.606705375 | 0.801820628 |
| ctx-rh-lateralorbitofrontal_FA | UPDRS 3, Off (Years 2-4) | Pearson | 0.067753864 | 0.690303133 | 0.801820628 |
| Left-Amygdala_FA | UPDRS 3, Off (Years 2-4) | Pearson | 0.059499752 | 0.726480598 | 0.801820628 |
| ctx-lh-insula_FA | UPDRS 3, Off (Years 2-4) | Pearson | -0.055428506 | 0.744547726 | 0.801820628 |
| ctx-lh-entorhinal_FA | UPDRS 3, Off (Years 0-4) | Pearson | 0.368621772 | 0.631378228 | 0.80357229 |
| Cerebrospinal Fluid - tTau | UPDRS 3, Off (Years 2-4) | Spearman | 0.20745732 | 0.231765271 | 0.811178447 |
| Left-Thalamus-Proper_FA | UPDRS 3, On (Years 0-4) | Pearson | 0.32856576 | 0.471823684 | 0.811911271 |
| Olfactory_Left_FA | UPDRS 3, On (Years 0-4) | Pearson | 0.294158573 | 0.52194296 | 0.811911271 |
| lh_insula_thickness | UPDRS 3, Off (Years 0-2) | Pearson | 0.610793754 | 0.389206246 | 0.822887295 |
| rh_medialorbitofrontal_thickness | UPDRS 3, Off (Years 0-2) | Pearson | 0.46038711 | 0.53961289 | 0.822887295 |
| rh_entorhinal_thickness | UPDRS 3, Off (Years 0-2) | Pearson | 0.427569013 | 0.572430987 | 0.822887295 |
| rh_insula_thickness | UPDRS 3, Off (Years 0-2) | Pearson | -0.328802119 | 0.671197881 | 0.822887295 |
| lh_medialorbitofrontal_thickness | UPDRS 3, Off (Years 0-2) | Pearson | 0.259449291 | 0.740550709 | 0.822887295 |
| rh_lateralorbitofrontal_thickness | UPDRS 3, Off (Years 0-2) | Pearson | -0.252108593 | 0.747891407 | 0.822887295 |
| lh_lateralorbitofrontal_thickness | UPDRS 3, Off (Years 0-2) | Pearson | -0.187200854 | 0.812799146 | 0.822887295 |
| lh_entorhinal_thickness | UPDRS 3, Off (Years 0-2) | Pearson | -0.177112705 | 0.822887295 | 0.822887295 |
| Right-Thalamus_volume | UPDRS 3, On (Years 0-4) | Pearson | -0.24105258 | 0.602561318 | 0.832433934 |
| Left-Thalamus_volume | UPDRS 3, On (Years 0-4) | Pearson | -0.192914218 | 0.678558607 | 0.832433934 |
| lh_entorhinal_volume | UPDRS 3, On (Years 0-4) | Pearson | -0.183502455 | 0.693694945 | 0.832433934 |
| lh_insula_thickness | UPDRS 3, Off (Years 2-4) | Pearson | -0.228711107 | 0.173332765 | 0.833152857 |
| rh_insula_thickness | UPDRS 3, Off (Years 2-4) | Pearson | -0.188434812 | 0.264034726 | 0.833152857 |
| lh_medialorbitofrontal_thickness | UPDRS 3, Off (Years 2-4) | Pearson | -0.170700584 | 0.312432321 | 0.833152857 |
| Cerebrospinal Fluid - pTau | UPDRS 3, Off (Years 0-4) | Spearman | 0.5 | 0.666666667 | 0.833333333 |
| Cerebrospinal Fluid - tTau | UPDRS 3, Off (Years 0-4) | Spearman | 0.5 | 0.666666667 | 0.833333333 |
| lh_entorhinal_thickness | UPDRS 3, Off (Years 2-4) | Pearson | -0.124750027 | 0.461936913 | 0.833568308 |
| rh_entorhinal_thickness | UPDRS 3, Off (Years 2-4) | Pearson | -0.108941428 | 0.520980193 | 0.833568308 |
| rh_lateralorbitofrontal_volume | UPDRS 3, On (Years 0-4) | Pearson | 0.125734631 | 0.788229518 | 0.841708164 |
| lh_lateralorbitofrontal_volume | UPDRS 3, On (Years 0-4) | Pearson | -0.09365174 | 0.841708164 | 0.841708164 |
| Left-Thalamus-Proper_FA | UPDRS 3, Off (Years 0-4) | Pearson | -0.27790071 | 0.72209929 | 0.842449172 |
| rh_lateralorbitofrontal_volume | UPDRS 3, Off (Years 0-4) | Pearson | -0.281658036 | 0.718341964 | 0.843484524 |
| lh_entorhinal_volume | UPDRS 3, Off (Years 0-4) | Pearson | 0.18968217 | 0.81031783 | 0.843484524 |
| Left-Amygdala_volume | UPDRS 3, Off (Years 0-4) | Pearson | -0.172546 | 0.827454 | 0.843484524 |
| lh_lateralorbitofrontal_volume | UPDRS 3, Off (Years 0-4) | Pearson | -0.156515476 | 0.843484524 | 0.843484524 |
| Serum - NfL | UPDRS 3, Off (Years 0-4) | Pearson | -0.234807459 | 0.849108073 | 0.849108073 |
| Right-Amygdala_FA | UPDRS 3, On (Years 0-4) | Pearson | 0.194099406 | 0.676658516 | 0.852380325 |
| Left-Amygdala_FA | UPDRS 3, On (Years 0-4) | Pearson | 0.191451428 | 0.680905616 | 0.852380325 |
| Olfactory_Right_FA | UPDRS 3, On (Years 0-4) | Pearson | -0.160751729 | 0.730611707 | 0.852380325 |
| Left-Amygdala_MD | UPDRS 3, Off (Years 2-4) | Pearson | -0.304479337 | 0.066908852 | 0.860303586 |
| ctx-lh-entorhinal_MD | UPDRS 3, Off (Years 2-4) | Pearson | -0.219808438 | 0.191142514 | 0.860303586 |
| ctx-rh-lateralorbitofrontal_MD | UPDRS 3, Off (Years 2-4) | Pearson | -0.214181359 | 0.203042494 | 0.860303586 |
| Olfactory_Right_MD | UPDRS 3, Off (Years 2-4) | Pearson | -0.175741406 | 0.29814121 | 0.860303586 |
| ctx-rh-entorhinal_MD | UPDRS 3, Off (Years 2-4) | Pearson | -0.169008081 | 0.317325852 | 0.860303586 |
| ctx-lh-medialorbitofrontal_MD | UPDRS 3, Off (Years 2-4) | Pearson | -0.152138541 | 0.368701537 | 0.860303586 |
| ctx-rh-entorhinal_MD | UPDRS 3, Off (Years 0-2) | Pearson | -0.874831042 | 0.125168958 | 0.876182708 |
| Cerebrospinal Fluid - a-Synuclein | UPDRS 3, On (Years 2-4) | Pearson | 0.182740419 | 0.308730109 | 0.893150076 |
| Cerebrospinal Fluid - tTau | UPDRS 3, On (Years 2-4) | Spearman | 0.124355415 | 0.319817199 | 0.893150076 |
| Cerebrospinal Fluid - ABeta 1-42 | UPDRS 3, On (Years 2-4) | Pearson | 0.099146677 | 0.431999288 | 0.893150076 |
| Serum - NfL | UPDRS 3, On (Years 2-4) | Pearson | 0.067408964 | 0.596635008 | 0.893150076 |
| Cerebrospinal Fluid - pTau | UPDRS 3, On (Years 2-4) | Spearman | 0.043370676 | 0.729511302 | 0.893150076 |
| Cerebrospinal Fluid - NFL | UPDRS 3, On (Years 2-4) | Pearson | -0.046904619 | 0.795481192 | 0.893150076 |
| Cerebrospinal Fluid - CSF Alpha-synuclein | UPDRS 3, On (Years 2-4) | Pearson | 0.016854428 | 0.893150076 | 0.893150076 |
| Cerebrospinal Fluid - CSF Alpha-synuclein | UPDRS 3, On (Years 0-4) | Pearson | -0.074573887 | 0.905137659 | 0.905137659 |
| lh_insula_thickness | UPDRS 3, On (Years 0-2) | Pearson | 0.54195711 | 0.208881676 | 0.907665335 |
| rh_lateralorbitofrontal_thickness | UPDRS 3, On (Years 0-2) | Pearson | -0.437121535 | 0.326735036 | 0.907665335 |
| lh_lateralorbitofrontal_thickness | UPDRS 3, On (Years 0-2) | Pearson | -0.385957967 | 0.392468853 | 0.907665335 |
| rh_medialorbitofrontal_thickness | UPDRS 3, On (Years 0-2) | Pearson | -0.260550039 | 0.572536228 | 0.907665335 |
| rh_insula_thickness | UPDRS 3, On (Years 0-2) | Pearson | -0.172404695 | 0.711647005 | 0.907665335 |
| lh_medialorbitofrontal_thickness | UPDRS 3, On (Years 0-2) | Pearson | -0.138051039 | 0.767864132 | 0.907665335 |
| rh_entorhinal_thickness | UPDRS 3, On (Years 0-2) | Pearson | 0.120229786 | 0.797363586 | 0.907665335 |
| lh_entorhinal_thickness | UPDRS 3, On (Years 0-2) | Pearson | 0.054470379 | 0.907665335 | 0.907665335 |
| Right-Thalamus-Proper_FA | UPDRS 3, On (Years 0-4) | Pearson | -0.092442406 | 0.843734588 | 0.908637249 |
| Right-Thalamus_volume | UPDRS 3, On (Years 2-4) | Pearson | -0.157604378 | 0.199287922 | 0.917159279 |
| Right-Amygdala_volume | UPDRS 3, On (Years 2-4) | Pearson | 0.131690635 | 0.284400639 | 0.917159279 |
| rh_lateralorbitofrontal_volume | UPDRS 3, On (Years 2-4) | Pearson | -0.109539226 | 0.373886607 | 0.917159279 |
| Left-Thalamus_volume | UPDRS 3, On (Years 2-4) | Pearson | -0.095406228 | 0.438977115 | 0.917159279 |
| rh_medialorbitofrontal_volume | UPDRS 3, On (Years 2-4) | Pearson | -0.091388198 | 0.45857964 | 0.917159279 |
| ctx-rh-medialorbitofrontal_FA | UPDRS 3, Off (Years 2-4) | Pearson | -0.016633814 | 0.922159844 | 0.922159844 |
| ctx-lh-entorhinal_FA | UPDRS 3, On (Years 0-4) | Pearson | 0.040848702 | 0.930710932 | 0.930710932 |
| rh_medialorbitofrontal_volume | UPDRS 3, Off (Years 0-2) | Pearson | -0.875100653 | 0.124899347 | 0.931664773 |
| Right-Thalamus_volume | UPDRS 3, Off (Years 0-2) | Pearson | -0.830774704 | 0.169225296 | 0.931664773 |
| rh_lateralorbitofrontal_volume | UPDRS 3, Off (Years 0-2) | Pearson | -0.696065449 | 0.303934551 | 0.931664773 |
| lh_medialorbitofrontal_volume | UPDRS 3, Off (Years 0-2) | Pearson | -0.652563496 | 0.347436504 | 0.931664773 |
| lh_lateralorbitofrontal_volume | UPDRS 3, Off (Years 0-2) | Pearson | -0.474192494 | 0.525807506 | 0.931664773 |
| Left-Amygdala_volume | UPDRS 3, Off (Years 0-2) | Pearson | 0.421865941 | 0.578134059 | 0.931664773 |
| lh_insula_volume | UPDRS 3, Off (Years 0-2) | Pearson | -0.299353282 | 0.700646718 | 0.931664773 |
| rh_insula_volume | UPDRS 3, Off (Years 0-2) | Pearson | -0.244482647 | 0.755517353 | 0.931664773 |
| Right-Amygdala_volume | UPDRS 3, Off (Years 0-2) | Pearson | 0.223668209 | 0.776331791 | 0.931664773 |
| lh_entorhinal_volume | UPDRS 3, Off (Years 0-2) | Pearson | 0.208232782 | 0.791767218 | 0.931664773 |
| Left-Thalamus_volume | UPDRS 3, Off (Years 0-2) | Pearson | -0.074808787 | 0.925191213 | 0.931664773 |
| rh_entorhinal_volume | UPDRS 3, Off (Years 0-2) | Pearson | -0.068335227 | 0.931664773 | 0.931664773 |
| lh_entorhinal_thickness | UPDRS 3, On (Years 0-4) | Pearson | 0.03457479 | 0.94133909 | 0.946220671 |
| lh_insula_thickness | UPDRS 3, On (Years 0-4) | Pearson | 0.031694556 | 0.946220671 | 0.946220671 |
| ctx-lh-lateralorbitofrontal_MD | UPDRS 3, Off (Years 2-4) | Pearson | -0.112869628 | 0.505970447 | 0.946657917 |
| Right-Amygdala_MD | UPDRS 3, Off (Years 2-4) | Pearson | -0.078781686 | 0.643017395 | 0.946657917 |
| ctx-rh-insula_MD | UPDRS 3, Off (Years 2-4) | Pearson | 0.074231019 | 0.66237585 | 0.946657917 |
| ctx-rh-medialorbitofrontal_MD | UPDRS 3, Off (Years 2-4) | Pearson | -0.069338151 | 0.683433139 | 0.946657917 |
| Left-Thalamus-Proper_MD | UPDRS 3, Off (Years 2-4) | Pearson | -0.034908033 | 0.837484864 | 0.946657917 |
| Right-Thalamus-Proper_MD | UPDRS 3, Off (Years 2-4) | Pearson | -0.01427263 | 0.93318253 | 0.946657917 |
| Olfactory_Left_MD | UPDRS 3, Off (Years 2-4) | Pearson | -0.012743854 | 0.94032597 | 0.946657917 |
| ctx-lh-insula_MD | UPDRS 3, Off (Years 2-4) | Pearson | -0.01138957 | 0.946657917 | 0.946657917 |
| rh_insula_volume | UPDRS 3, Off (Years 2-4) | Pearson | -0.011188436 | 0.947598607 | 0.947598607 |
| ctx-lh-lateralorbitofrontal_MD | UPDRS 3, On (Years 0-2) | Pearson | -0.56892766 | 0.182555971 | 0.951280855 |
| ctx-lh-entorhinal_MD | UPDRS 3, On (Years 0-2) | Pearson | -0.490052915 | 0.264246841 | 0.951280855 |
| ctx-lh-insula_MD | UPDRS 3, On (Years 0-2) | Pearson | -0.457335172 | 0.302182477 | 0.951280855 |
| ctx-lh-medialorbitofrontal_MD | UPDRS 3, On (Years 0-2) | Pearson | -0.42667758 | 0.339743162 | 0.951280855 |
| Olfactory_Left_MD | UPDRS 3, On (Years 2-4) | Pearson | 0.006073043 | 0.96079851 | 0.96079851 |
| Right-Thalamus-Proper_FA | UPDRS 3, Off (Years 0-4) | Pearson | -0.105258319 | 0.894741681 | 0.963567964 |
| rh_medialorbitofrontal_volume | UPDRS 3, On (Years 0-2) | Pearson | -0.530604977 | 0.220469683 | 0.964644737 |
| rh_lateralorbitofrontal_volume | UPDRS 3, On (Years 0-2) | Pearson | -0.38877314 | 0.388723938 | 0.964644737 |
| lh_medialorbitofrontal_volume | UPDRS 3, On (Years 0-2) | Pearson | -0.322051942 | 0.481173766 | 0.964644737 |
| lh_insula_volume | UPDRS 3, On (Years 0-2) | Pearson | -0.269337984 | 0.559160432 | 0.964644737 |
| Right-Thalamus_volume | UPDRS 3, On (Years 0-2) | Pearson | 0.255900599 | 0.579653178 | 0.964644737 |
| Right-Amygdala_volume | UPDRS 3, On (Years 0-2) | Pearson | -0.220425737 | 0.634817879 | 0.964644737 |
| Left-Thalamus_volume | UPDRS 3, On (Years 0-2) | Pearson | 0.216508945 | 0.640996906 | 0.964644737 |
| lh_lateralorbitofrontal_volume | UPDRS 3, On (Years 0-2) | Pearson | 0.191882645 | 0.680213527 | 0.964644737 |
| rh_entorhinal_volume | UPDRS 3, On (Years 0-2) | Pearson | -0.125452574 | 0.788697069 | 0.964644737 |
| Left-Amygdala_volume | UPDRS 3, On (Years 0-2) | Pearson | 0.116314968 | 0.803870614 | 0.964644737 |
| ctx-lh-lateralorbitofrontal_MD | UPDRS 3, Off (Years 0-2) | Pearson | -0.720883337 | 0.279116663 | 0.965770635 |
| ctx-lh-medialorbitofrontal_MD | UPDRS 3, Off (Years 0-2) | Pearson | -0.421685565 | 0.578314435 | 0.965770635 |
| Right-Amygdala_MD | UPDRS 3, Off (Years 0-2) | Pearson | -0.395024779 | 0.604975221 | 0.965770635 |
| Olfactory_Left_MD | UPDRS 3, Off (Years 0-2) | Pearson | -0.241735028 | 0.758264972 | 0.965770635 |
| Left-Thalamus-Proper_MD | UPDRS 3, Off (Years 0-2) | Pearson | 0.204995067 | 0.795004933 | 0.965770635 |
| ctx-lh-insula_MD | UPDRS 3, Off (Years 0-2) | Pearson | -0.196364423 | 0.803635577 | 0.965770635 |
| ctx-rh-medialorbitofrontal_MD | UPDRS 3, Off (Years 0-2) | Pearson | -0.147936762 | 0.852063238 | 0.965770635 |
| Left-Amygdala_MD | UPDRS 3, Off (Years 0-2) | Pearson | 0.138347742 | 0.861652258 | 0.965770635 |
| ctx-rh-lateralorbitofrontal_MD | UPDRS 3, Off (Years 0-2) | Pearson | -0.112022265 | 0.887977735 | 0.965770635 |
| Right-Thalamus-Proper_MD | UPDRS 3, Off (Years 0-2) | Pearson | -0.052990692 | 0.947009308 | 0.965770635 |
| ctx-rh-insula_MD | UPDRS 3, Off (Years 0-2) | Pearson | -0.041238555 | 0.958761445 | 0.965770635 |
| Olfactory_Right_MD | UPDRS 3, Off (Years 0-2) | Pearson | -0.034229365 | 0.965770635 | 0.965770635 |
| Left-Thalamus-Proper_FA | UPDRS 3, On (Years 2-4) | Pearson | -0.023461006 | 0.84938458 | 0.966167449 |
| ctx-lh-medialorbitofrontal_FA | UPDRS 3, On (Years 2-4) | Pearson | 0.01596961 | 0.897155488 | 0.966167449 |
| ctx-rh-lateralorbitofrontal_FA | UPDRS 3, On (Years 2-4) | Pearson | 0.005061262 | 0.967325635 | 0.967325635 |
| Olfactory_Right_FA | UPDRS 3, Off (Years 0-4) | Pearson | -0.02634202 | 0.97365798 | 0.97365798 |
| lh_entorhinal_volume | UPDRS 3, On (Years 0-2) | Pearson | -0.049226057 | 0.916532466 | 0.976462958 |
| rh_insula_volume | UPDRS 3, On (Years 0-2) | Pearson | 0.013865795 | 0.976462958 | 0.976462958 |
| lh_entorhinal_volume | UPDRS 3, On (Years 2-4) | Pearson | -0.067596961 | 0.583890032 | 0.981007943 |
| rh_entorhinal_volume | UPDRS 3, On (Years 2-4) | Pearson | -0.039275312 | 0.750491988 | 0.981007943 |
| lh_insula_volume | UPDRS 3, On (Years 2-4) | Pearson | -0.035673211 | 0.772729133 | 0.981007943 |
| lh_medialorbitofrontal_volume | UPDRS 3, On (Years 2-4) | Pearson | 0.007918927 | 0.948897405 | 0.981007943 |
| lh_lateralorbitofrontal_volume | UPDRS 3, On (Years 2-4) | Pearson | 0.004669206 | 0.969855429 | 0.981007943 |
| rh_insula_volume | UPDRS 3, On (Years 2-4) | Pearson | -0.002941339 | 0.981007943 | 0.981007943 |
| Cerebrospinal Fluid - NFL | UPDRS 3, Off (Years 2-4) | Pearson | -0.157517819 | 0.495293764 | 0.98299606 |
| Cerebrospinal Fluid - CSF Alpha-synuclein | UPDRS 3, Off (Years 2-4) | Pearson | 0.090973764 | 0.603243644 | 0.98299606 |
| Serum - NfL | UPDRS 3, Off (Years 2-4) | Pearson | 0.06236044 | 0.721932307 | 0.98299606 |
| Cerebrospinal Fluid - a-Synuclein | UPDRS 3, Off (Years 2-4) | Pearson | 0.026026932 | 0.91083488 | 0.98299606 |
| Cerebrospinal Fluid - ABeta 1-42 | UPDRS 3, Off (Years 2-4) | Pearson | -0.003797154 | 0.98299606 | 0.98299606 |
| Right-Thalamus-Proper_MD | UPDRS 3, On (Years 0-4) | Pearson | 0.590192592 | 0.163019115 | 0.983843599 |
| Left-Thalamus-Proper_MD | UPDRS 3, On (Years 0-4) | Pearson | 0.555478084 | 0.195470103 | 0.983843599 |
| ctx-lh-lateralorbitofrontal_MD | UPDRS 3, On (Years 0-4) | Pearson | 0.500062715 | 0.253100846 | 0.983843599 |
| ctx-rh-medialorbitofrontal_MD | UPDRS 3, On (Years 0-4) | Pearson | 0.430515569 | 0.334937752 | 0.983843599 |
| Olfactory_Right_MD | UPDRS 3, On (Years 0-4) | Pearson | 0.417483018 | 0.351372714 | 0.983843599 |
| rh_lateralorbitofrontal_thickness | UPDRS 3, Off (Years 2-4) | Pearson | -0.032776179 | 0.847289142 | 0.985133141 |
| rh_medialorbitofrontal_thickness | UPDRS 3, Off (Years 2-4) | Pearson | 0.013029765 | 0.938989639 | 0.985133141 |
| lh_lateralorbitofrontal_thickness | UPDRS 3, Off (Years 2-4) | Pearson | -0.003172275 | 0.985133141 | 0.985133141 |
| Olfactory_Right_FA | UPDRS 3, Off (Years 0-2) | Pearson | 0.01330704 | 0.98669296 | 0.98669296 |
| ctx-rh-medialorbitofrontal_MD | UPDRS 3, On (Years 0-2) | Pearson | -0.297426183 | 0.517107557 | 0.992753903 |
| Left-Amygdala_MD | UPDRS 3, On (Years 0-2) | Pearson | 0.268964799 | 0.55972639 | 0.992753903 |
| ctx-rh-insula_MD | UPDRS 3, On (Years 0-2) | Pearson | -0.257823618 | 0.576706261 | 0.992753903 |
| ctx-rh-lateralorbitofrontal_MD | UPDRS 3, On (Years 0-2) | Pearson | -0.123123293 | 0.792560105 | 0.992753903 |
| Right-Thalamus-Proper_MD | UPDRS 3, On (Years 0-2) | Pearson | -0.119673025 | 0.798288446 | 0.992753903 |
| Right-Amygdala_MD | UPDRS 3, On (Years 0-2) | Pearson | 0.064940403 | 0.889986069 | 0.992753903 |
| Olfactory_Right_MD | UPDRS 3, On (Years 0-2) | Pearson | -0.05981807 | 0.898631277 | 0.992753903 |
| Left-Thalamus-Proper_MD | UPDRS 3, On (Years 0-2) | Pearson | -0.046087256 | 0.921842909 | 0.992753903 |
| ctx-rh-entorhinal_MD | UPDRS 3, Off (Years 0-4) | Pearson | -0.849974274 | 0.150025726 | 0.995049359 |
| Left-Thalamus-Proper_MD | UPDRS 3, Off (Years 0-4) | Pearson | 0.745043656 | 0.254956344 | 0.995049359 |
| ctx-lh-entorhinal_MD | UPDRS 3, Off (Years 0-4) | Pearson | -0.637690782 | 0.362309218 | 0.995049359 |
| Right-Thalamus-Proper_MD | UPDRS 3, Off (Years 0-4) | Pearson | 0.546832884 | 0.453167116 | 0.995049359 |
| ctx-rh-insula_MD | UPDRS 3, Off (Years 0-4) | Pearson | 0.522336863 | 0.477663137 | 0.995049359 |
| Left-Amygdala_MD | UPDRS 3, Off (Years 0-4) | Pearson | 0.520468129 | 0.479531871 | 0.995049359 |
| ctx-rh-medialorbitofrontal_MD | UPDRS 3, Off (Years 0-4) | Pearson | 0.36930142 | 0.63069858 | 0.995049359 |
| ctx-lh-insula_MD | UPDRS 3, Off (Years 0-4) | Pearson | 0.322822821 | 0.677177179 | 0.995049359 |
| Olfactory_Right_MD | UPDRS 3, Off (Years 0-4) | Pearson | 0.283871444 | 0.716128556 | 0.995049359 |
| ctx-lh-medialorbitofrontal_MD | UPDRS 3, Off (Years 0-4) | Pearson | -0.145730311 | 0.854269689 | 0.995049359 |
| ctx-rh-lateralorbitofrontal_MD | UPDRS 3, Off (Years 0-4) | Pearson | 0.120227734 | 0.879772266 | 0.995049359 |
| Right-Amygdala_MD | UPDRS 3, Off (Years 0-4) | Pearson | -0.049597949 | 0.950402051 | 0.995049359 |
| Olfactory_Left_MD | UPDRS 3, Off (Years 0-4) | Pearson | -0.022379047 | 0.977620953 | 0.995049359 |
| ctx-lh-lateralorbitofrontal_MD | UPDRS 3, Off (Years 0-4) | Pearson | -0.004950641 | 0.995049359 | 0.995049359 |
| ctx-rh-insula_MD | UPDRS 3, On (Years 0-4) | Pearson | 0.358507679 | 0.429724793 | 0.997575042 |
| ctx-rh-lateralorbitofrontal_MD | UPDRS 3, On (Years 0-4) | Pearson | 0.257154662 | 0.577730868 | 0.997575042 |
| ctx-rh-entorhinal_MD | UPDRS 3, On (Years 0-4) | Pearson | -0.247824933 | 0.59207916 | 0.997575042 |
| ctx-lh-insula_MD | UPDRS 3, On (Years 0-4) | Pearson | 0.189408559 | 0.68418677 | 0.997575042 |
| ctx-lh-medialorbitofrontal_MD | UPDRS 3, On (Years 0-4) | Pearson | -0.149335114 | 0.749298218 | 0.997575042 |
| ctx-lh-entorhinal_MD | UPDRS 3, On (Years 0-4) | Pearson | 0.092411658 | 0.843786122 | 0.997575042 |
| Left-Amygdala_MD | UPDRS 3, On (Years 0-4) | Pearson | -0.023544645 | 0.960040447 | 0.997575042 |
| Olfactory_Left_MD | UPDRS 3, On (Years 0-4) | Pearson | 0.016495809 | 0.971999655 | 0.997575042 |
| Right-Amygdala_MD | UPDRS 3, On (Years 0-4) | Pearson | 0.001428419 | 0.997575042 | 0.997575042 |
| Olfactory_Left_MD | UPDRS 3, On (Years 0-2) | Pearson | -0.000210141 | 0.999643254 | 0.999643254 |

NfL: neurofilament light chain, CSF: cerebrospinal fluid, Aβ1-42: Beta-Amyloid-(1-42), pTau: phosphorylated Tau, tTau: total Tau, ctx: cortex, rh: right hemisphere, lh: left hemisphere, MD: mean diffusivity, FA: fractional anisotropy, UPDRS 3: the Unified Parkinson's Disease Rating Scale Part-III, On: on-state, Off: off-state, FDR: false discovery rate.

**eTable 4.** The longitudinal micro- and macrostructural changes in olfactory-related regions

| **Target** | **Test Type** | **Main Statistic** | **Main P-Value** | **Main P-Value (FDR-corrected)** | **Post-Hoc (Years 0-2) Statistic** | **Post-Hoc (Years 0-2) P-Value** | **Post-Hoc (Years 0-4) Statistic** | **Post-Hoc (Years 0-4) P-Value** | **Post-Hoc (Years 2-4) Statistic** | **Post-Hoc (Years 2-4) P-Value** |
| --- | --- | --- | --- | --- | --- | --- | --- | --- | --- | --- |
| Olfactory_Left_MD | Friedman | 1.072164948 | 0.585035661 | 0.668612184 | NA | NA | NA | NA | NA | NA |
| Olfactory_Right_MD | Friedman | 0.577319588 | 0.749267067 | 0.749267067 | NA | NA | NA | NA | NA | NA |
| Left-Amygdala_MD | Friedman | 5.587628866 | 0.061187373 | 0.258641303 | NA | NA | NA | NA | NA | NA |
| Right-Amygdala_MD | Friedman | 5.030927835 | 0.080825407 | 0.258641303 | NA | NA | NA | NA | NA | NA |
| ctx-lh-entorhinal_MD | Friedman | 3.051546392 | 0.217452858 | 0.406110337 | NA | NA | NA | NA | NA | NA |
| ctx-lh-lateralorbitofrontal_MD | Friedman | 2.742268041 | 0.25381896 | 0.406110337 | NA | NA | NA | NA | NA | NA |
| ctx-lh-medialorbitofrontal_MD | Friedman | 4.597938144 | 0.100362257 | 0.267632684 | NA | NA | NA | NA | NA | NA |
| ctx-lh-insula_MD | Friedman | 5.195876289 | 0.074426878 | 0.258641303 | NA | NA | NA | NA | NA | NA |
| ctx-rh-entorhinal_MD | Friedman | 3.979381443 | 0.136737709 | 0.312543335 | NA | NA | NA | NA | NA | NA |
| ctx-rh-lateralorbitofrontal_MD | Friedman | 2.865979381 | 0.238594531 | 0.406110337 | NA | NA | NA | NA | NA | NA |
| ctx-rh-medialorbitofrontal_MD | Friedman | 1.381443299 | 0.501214238 | 0.668612184 | NA | NA | NA | NA | NA | NA |
| ctx-rh-insula_MD | Friedman | 1.175257732 | 0.555643229 | 0.668612184 | NA | NA | NA | NA | NA | NA |
| Left-Thalamus-Proper_MD | Friedman | 30.94845361 | 1.90E-07 | 3.05E-06 | 1308 | 0.000120694 | 1047 | 1.72E-06 | 1727 | 0.019436427 |
| Right-Thalamus-Proper_MD | Friedman | 12.86597938 | 0.001607637 | 0.012861098 | 1523 | 0.002132769 | 1268 | 6.65E-05 | 1881 | 0.074598517 |
| Olfactory_Left_MD | Friedman | 1.072164948 | 0.585035661 | 0.668612184 | NA | NA | NA | NA | NA | NA |
| Olfactory_Right_MD | Friedman | 0.577319588 | 0.749267067 | 0.749267067 | NA | NA | NA | NA | NA | NA |
| Left-Amygdala_FA | Friedman | 6.927835052 | 0.031306876 | 0.146098755 | 1934 | 0.111335602 | 1719 | 0.017989271 | 2081 | 0.287655824 |
| Right-Amygdala_FA | Friedman | 4.350515464 | 0.113578877 | 0.26501738 | NA | NA | NA | NA | NA | NA |
| ctx-lh-entorhinal_FA | Friedman | 0.762886598 | 0.682875105 | 0.86911377 | NA | NA | NA | NA | NA | NA |
| ctx-lh-lateralorbitofrontal_FA | Friedman | 5.587628866 | 0.061187373 | 0.214155806 | NA | NA | NA | NA | NA | NA |
| ctx-lh-medialorbitofrontal_FA | ANOVA | 0.759819834 | 0.468685996 | 0.729067105 | NA | NA | NA | NA | NA | NA |
| ctx-lh-insula_FA | Friedman | 8.309278351 | 0.015691452 | 0.109840164 | 1719 | 0.017989271 | 1557 | 0.003190591 | 2014 | 0.192110919 |
| ctx-rh-entorhinal_FA | Friedman | 0.515463918 | 0.772802347 | 0.901602738 | NA | NA | NA | NA | NA | NA |
| ctx-rh-lateralorbitofrontal_FA | ANOVA | 0.154388549 | 0.857009869 | 0.922933705 | NA | NA | NA | NA | NA | NA |
| ctx-rh-medialorbitofrontal_FA | ANOVA | 0.46224076 | 0.630337149 | 0.86911377 | NA | NA | NA | NA | NA | NA |
| ctx-rh-insula_FA | Friedman | 13.54639175 | 0.001144033 | 0.016016457 | 1919 | 0.099724219 | 1425 | 0.000617703 | 1824 | 0.046809001 |
| Left-Thalamus-Proper_FA | Friedman | 4.721649485 | 0.094342383 | 0.264158672 | NA | NA | NA | NA | NA | NA |
| Right-Thalamus-Proper_FA | ANOVA | 1.902747163 | 0.151028636 | 0.302057272 | NA | NA | NA | NA | NA | NA |
| Olfactory_Left_FA | Friedman | 2.556701031 | 0.278496297 | 0.48736852 | NA | NA | NA | NA | NA | NA |
| Olfactory_Right_FA | Friedman | 0.144329897 | 0.930377419 | 0.930377419 | NA | NA | NA | NA | NA | NA |
| lh_entorhinal_volume | Friedman | 6.515463918 | 0.038475563 | 0.055575814 | 2204 | 0.534800073 | 1627 | 0.006999324 | 1956 | 0.130265074 |
| lh_lateralorbitofrontal_volume | Friedman | 7.072164948 | 0.02912721 | 0.054093391 | 2156 | 0.427538631 | 1755 | 0.025331965 | 2080 | 0.286027654 |
| lh_medialorbitofrontal_volume | Friedman | 9.917525773 | 0.007021609 | 0.015213486 | 2111 | 0.339410263 | 1579 | 0.004110022 | 1973 | 0.146532905 |
| lh_insula_volume | Friedman | 12.12371134 | 0.002330073 | 0.007572737 | 2003 | 0.178967654 | 1503 | 0.00167193 | 1549 | 0.00290573 |
| rh_entorhinal_volume | Friedman | 6.742268041 | 0.034350661 | 0.055575814 | 2102 | 0.323292124 | 1474 | 0.00116462 | 1864 | 0.065169303 |
| rh_lateralorbitofrontal_volume | ANOVA | 0.989725697 | 0.372939168 | 0.440746289 | NA | NA | NA | NA | NA | NA |
| rh_medialorbitofrontal_volume | Friedman | 0.762886598 | 0.682875105 | 0.739781363 | NA | NA | NA | NA | NA | NA |
| rh_insula_volume | ANOVA | 0.27585417 | 0.759123891 | 0.759123891 | NA | NA | NA | NA | NA | NA |
| Left-Amygdala_volume | Friedman | 10.57731959 | 0.005048522 | 0.013126157 | 1601 | 0.0052638 | 1360 | 0.000254583 | 1738 | 0.021591833 |
| Right-Amygdala_volume | ANOVA | 2.756368408 | 0.065198433 | 0.084757963 | NA | NA | NA | NA | NA | NA |
| Left-Thalamus_volume | Friedman | 39.40206186 | 2.78E-09 | 3.61E-08 | 1899 | 0.085768025 | 1019 | 1.04E-06 | 1266 | 6.45E-05 |
| Right-Thalamus_volume | Friedman | 27.60824742 | 1.01E-06 | 4.38E-06 | 1973 | 0.146532905 | 1120 | 6.15E-06 | 1172 | 1.46E-05 |
| Right-Thalamus_volume | Friedman | 27.60824742 | 1.01E-06 | 4.38E-06 | 1973 | 0.146532905 | 1120 | 6.15E-06 | 1172 | 1.46E-05 |
| lh_entorhinal_thickness | Friedman | 1.917525773 | 0.383366861 | 0.766733722 | NA | NA | NA | NA | NA | NA |
| lh_lateralorbitofrontal_thickness | Friedman | 7.855670103 | 0.019686246 | 0.078744985 | 2074 | 0.276376311 | 1579 | 0.004109267 | 2013 | 0.190873885 |
| lh_medialorbitofrontal_thickness | Friedman | 0.976744186 | 0.613624505 | 0.905753642 | NA | NA | NA | NA | NA | NA |
| lh_insula_thickness | ANOVA | 0.099021959 | 0.905753642 | 0.905753642 | NA | NA | NA | NA | NA | NA |
| rh_entorhinal_thickness | Friedman | 14.37209302 | 0.000757076 | 0.00605661 | 1425.5 | 0.000621773 | 1321 | 0.000145897 | 2053 | 0.314924691 |
| rh_lateralorbitofrontal_thickness | ANOVA | 0.12338821 | 0.88396715 | 0.905753642 | NA | NA | NA | NA | NA | NA |
| rh_medialorbitofrontal_thickness | Friedman | 3.105943152 | 0.211618199 | 0.564315197 | NA | NA | NA | NA | NA | NA |
| rh_insula_thickness | Friedman | 0.687338501 | 0.709163445 | 0.905753642 | NA | NA | NA | NA | NA | NA |

MD: mean diffusivity, FA: fractional anisotropy, ctx: cortex, rh: right hemisphere, lh: left hemisphere, FDR: false discovery rate, NA: not applicable.
